# Supplementary material for: Deciphering Tumour Microenvironment of Liver Cancer through Deconvolution of Bulk RNA-Seq Data with Single-Cell Atlas
Source: Cancers (Basel). 2022 Dec 27;15(1):153. doi: 10.3390/cancers15010153 (PMC9818189; doi:10.3390/cancers15010153)

Deconvolution Output – Other Cells

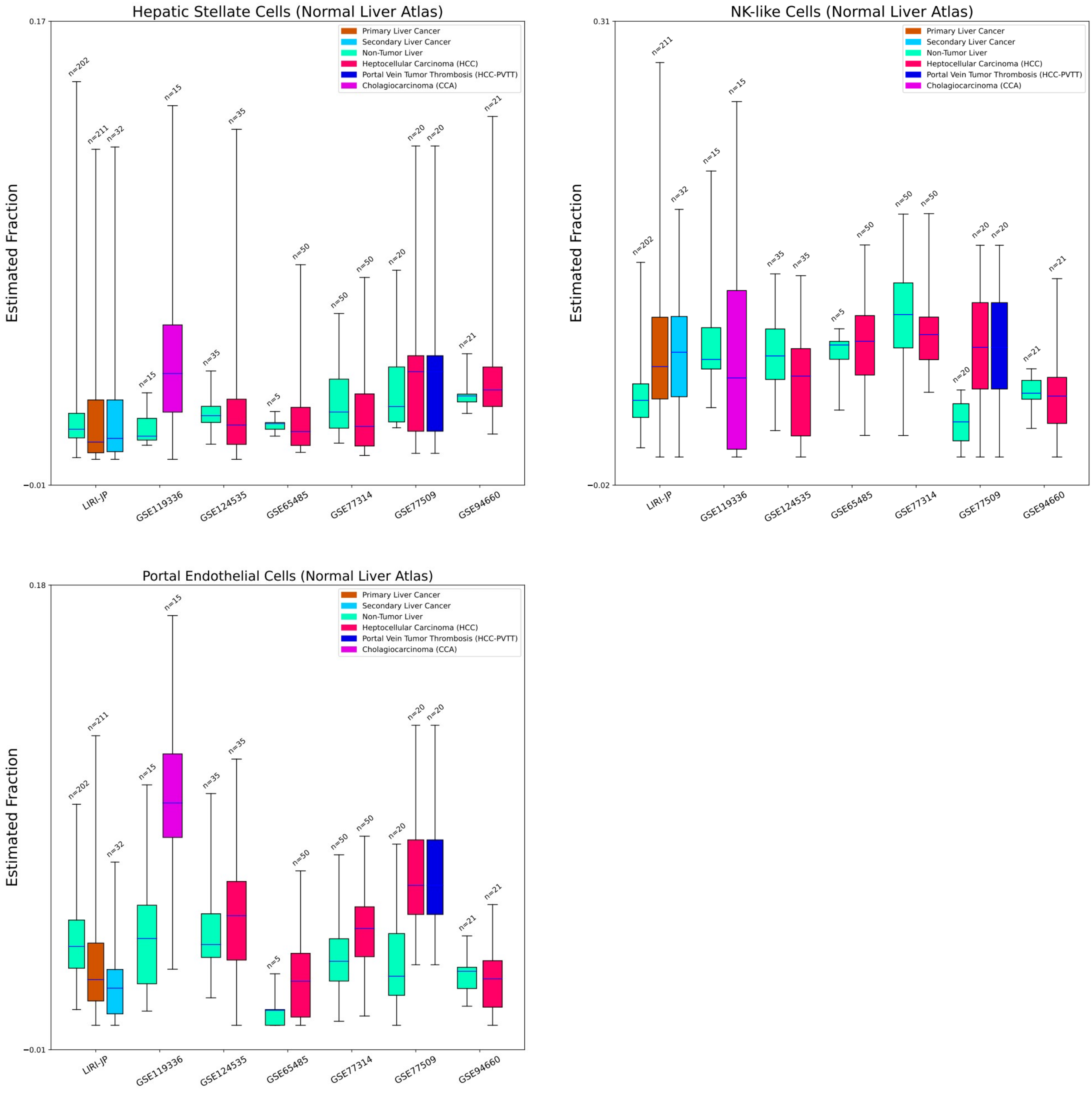

Deconvolution Output – Other Cells

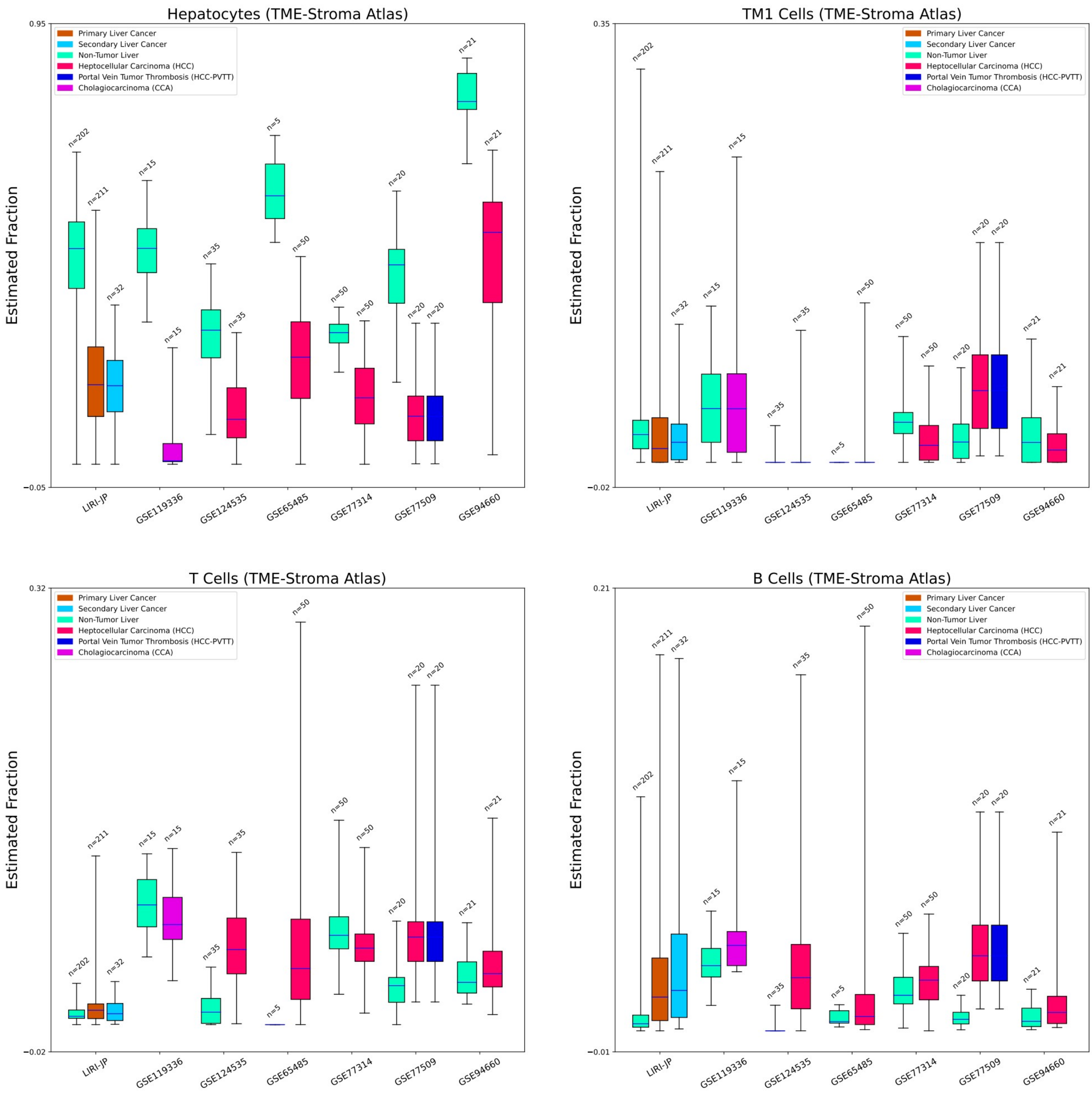

Deconvolution Output – Other Cells

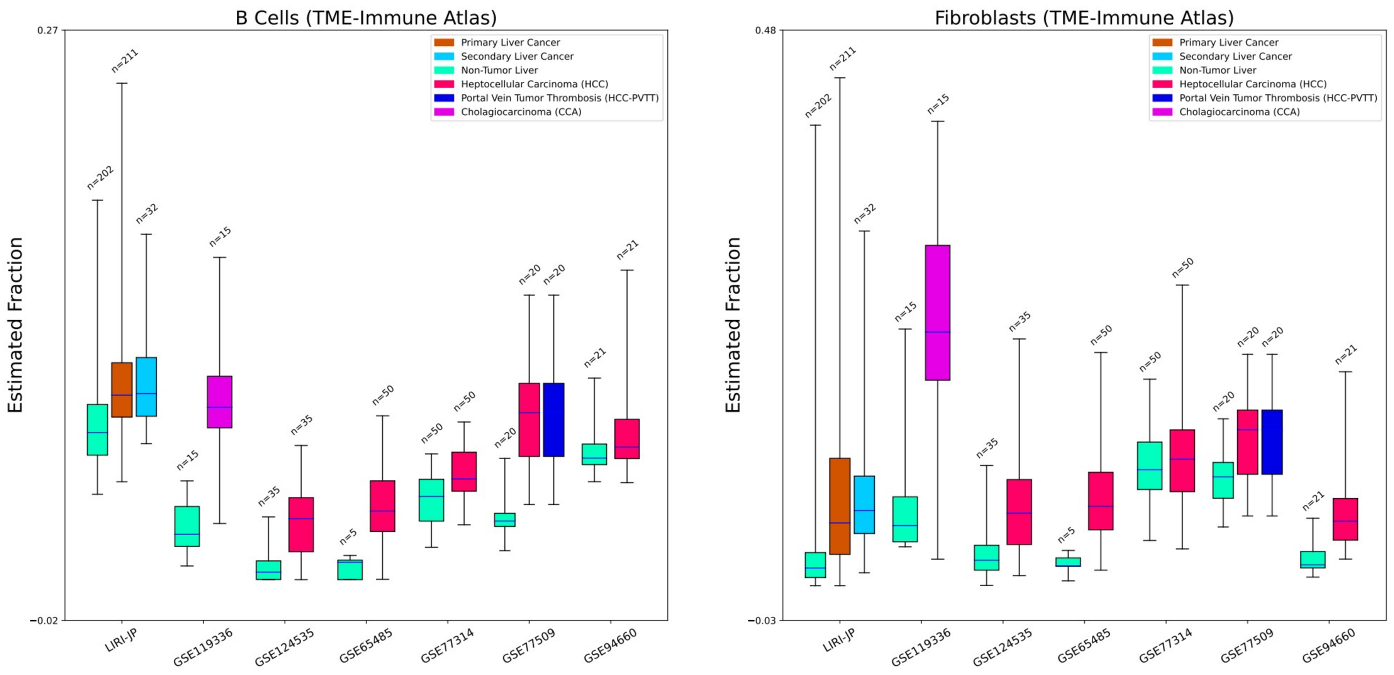

Estimation by Support Vector Regression – Other Cell Types (Normal Atlas)

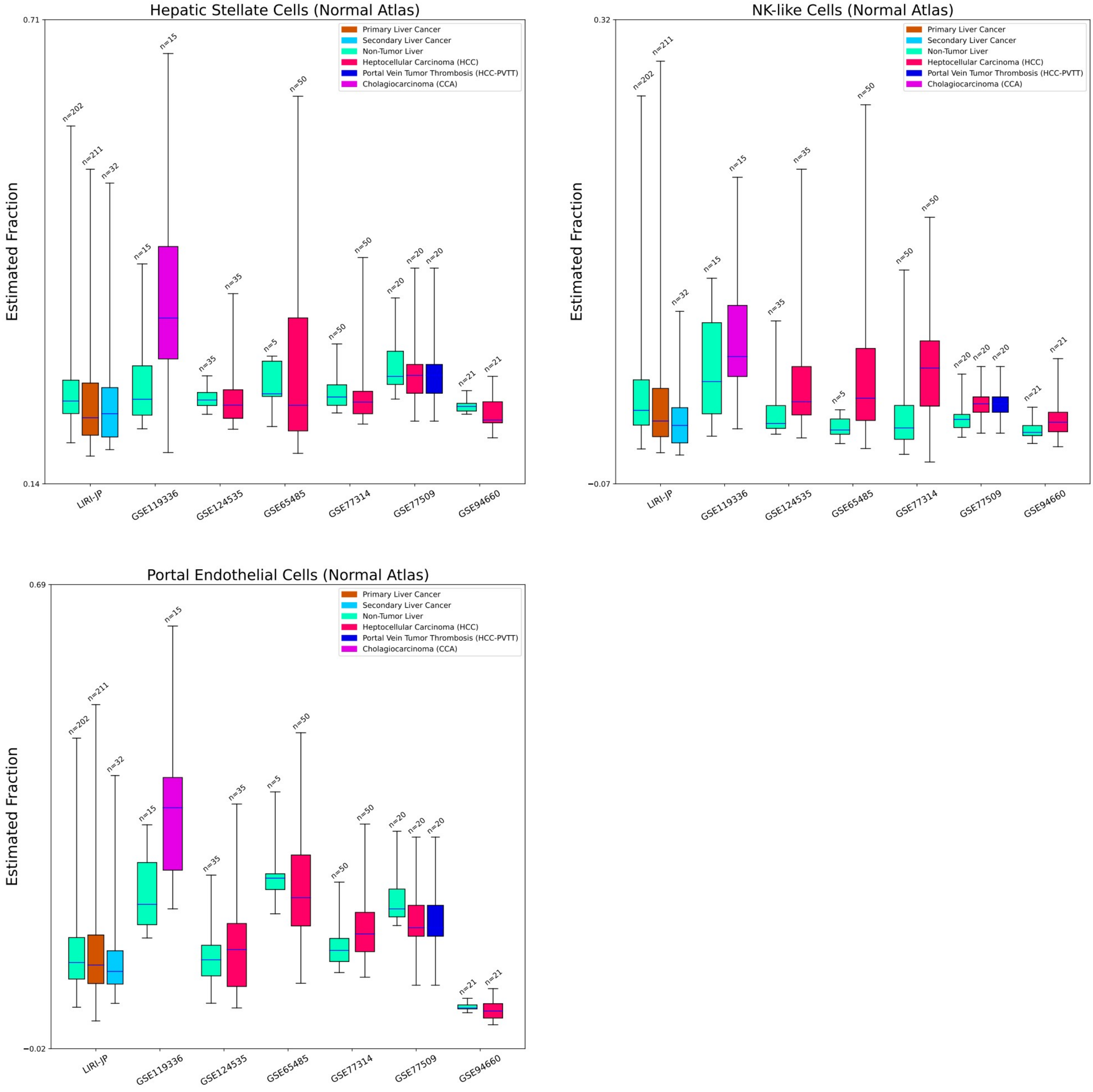

Estimation by Support Vector Regression – Other Cell Types (TME-Stroma Atlas)

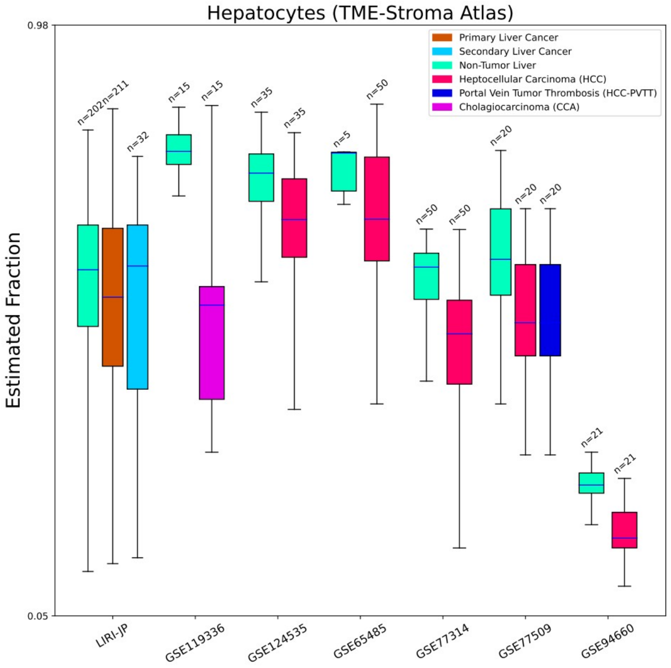

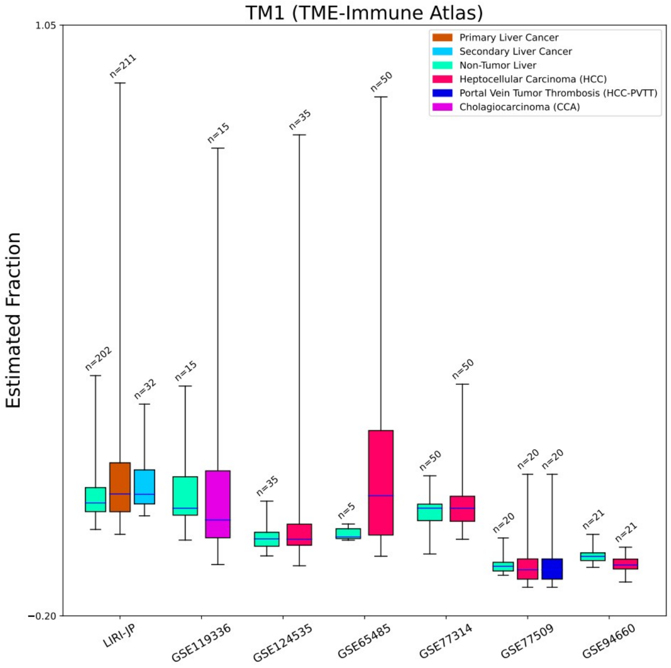

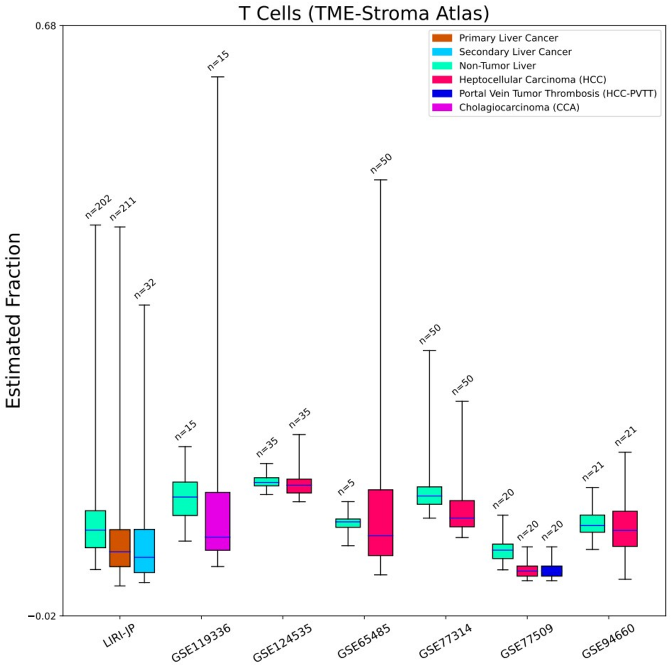

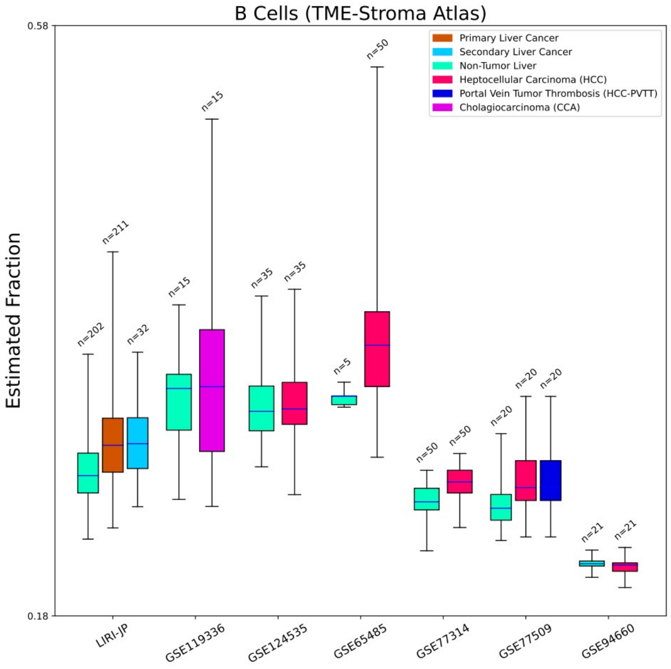

Estimation by Support Vector Regression – Other Cell Types (TME-Immune Atlas)

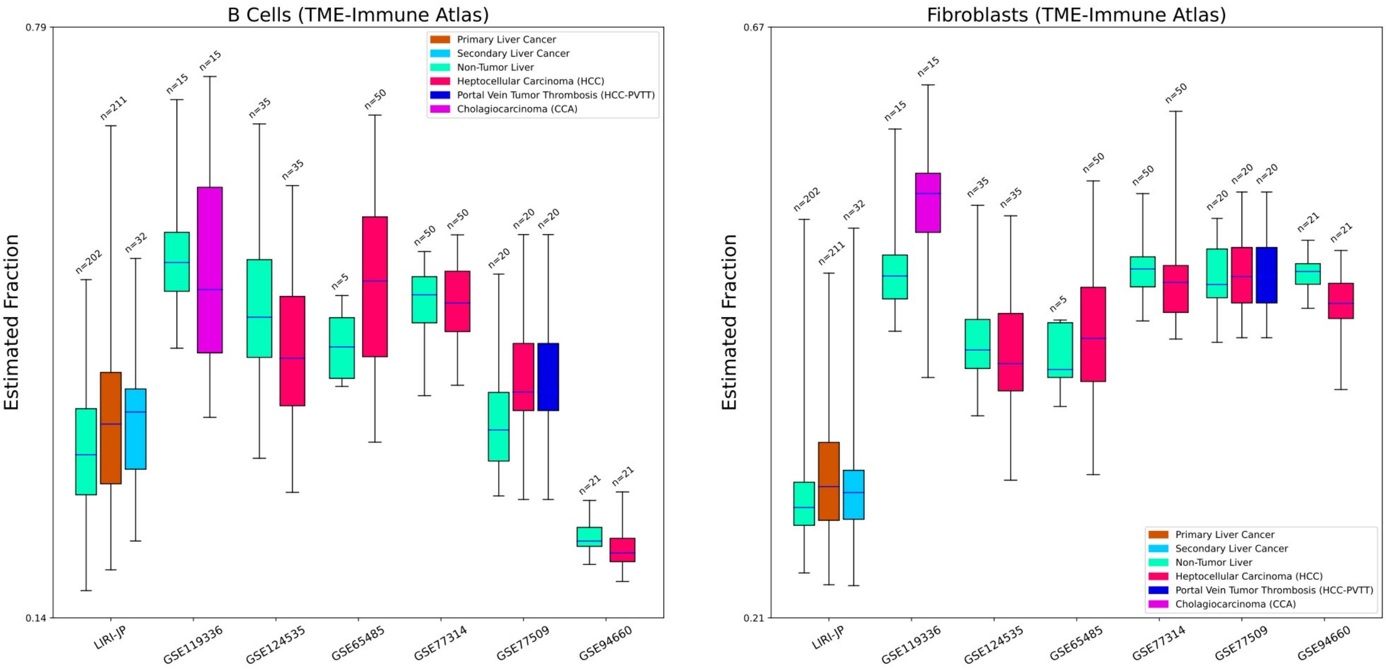

Supplement: Supplementary file 1 [file cancers-15-00153-s001.zip › cancers-2059594-supplementary/Supplements/S3_Estimation_by_Cibersortx_and_Support_Vector_Regression_Other_Cell_Types.pdf]
