# Supplementary material for: Deciphering Tumour Microenvironment of Liver Cancer through Deconvolution of Bulk RNA-Seq Data with Single-Cell Atlas
Source: Cancers (Basel). 2022 Dec 27;15(1):153. doi: 10.3390/cancers15010153 (PMC9818189; doi:10.3390/cancers15010153)

Summary of *In Silico* Validation – Normal Atlas

	Pearson Correlation Coefficient	Mean Absolute Error	Error Propensity
alpha-beta T Cells	0.9936	0.0304	⇧
gamma-delta T Cells	0.9852	0.1227	⇩
Central Venous LSECs	0.9937	0.0841	⇩
Periportal LSECs	0.9931	0.0764	⇩
Cholangiocytes	0.9915	0.1198	⇩
Erythroid Cells	0.9586	0.4574	⇩
Hepatic Stellate Cells	0.9901	0.1379	⇩
Hepatocyte	0.979	0.1481	⇧
Inflammatory Macrophage	0.9981	0.0202	⇧
Non-inflammatory Macrophage	0.9817	0.2695	⇩
Mature B Cells	0.9968	0.0327	⇩
Plasma Cells	0.9328	0.4348	⇩
NK-like Cells	0.9945	0.0611	⇧
Portal Endothelial Cells	0.9928	0.0648	⇩

In Silico Validation of Cibersortx – Normal Atlas

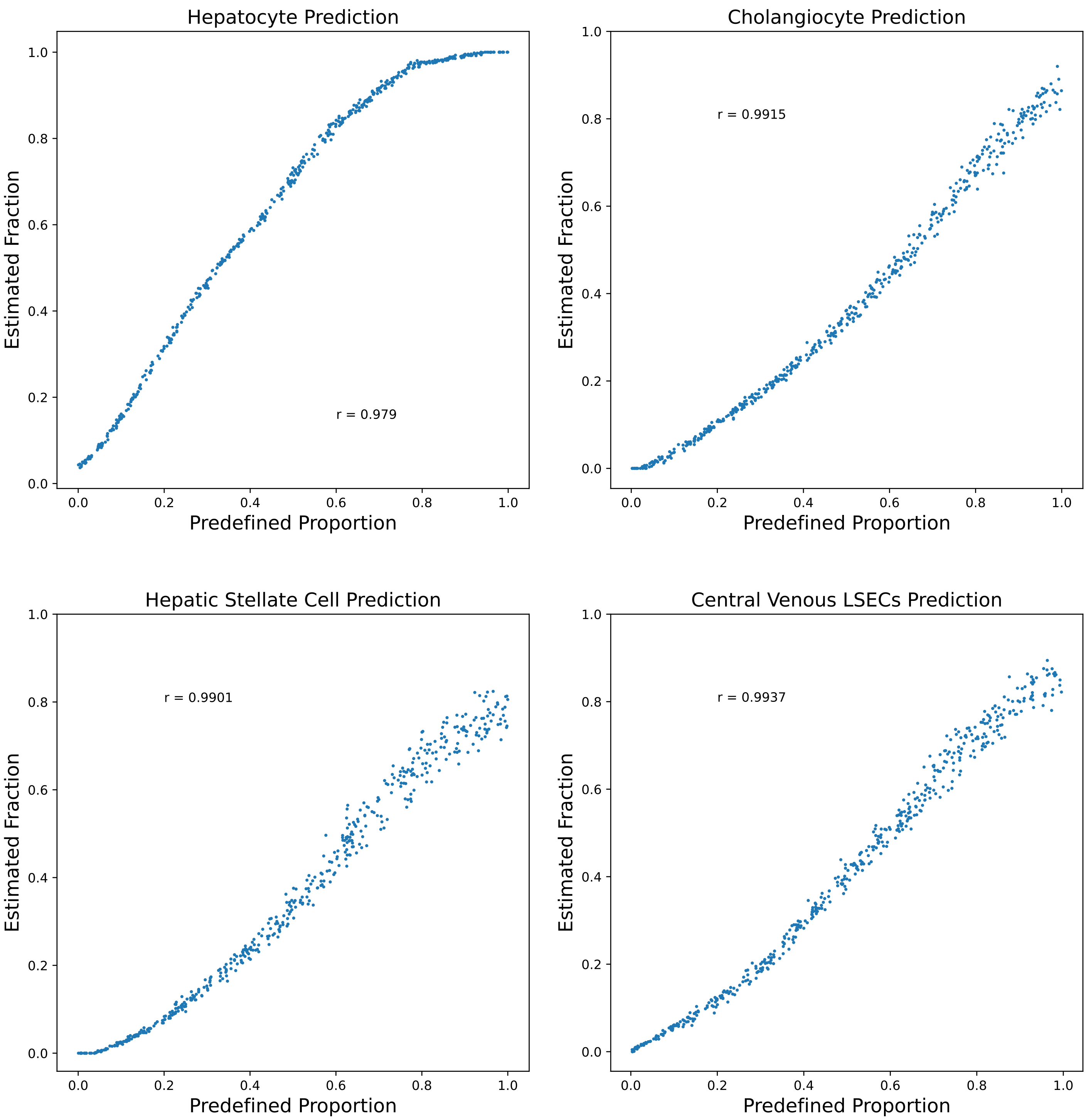

In Silico Validation of Cibersortx – Normal Atlas

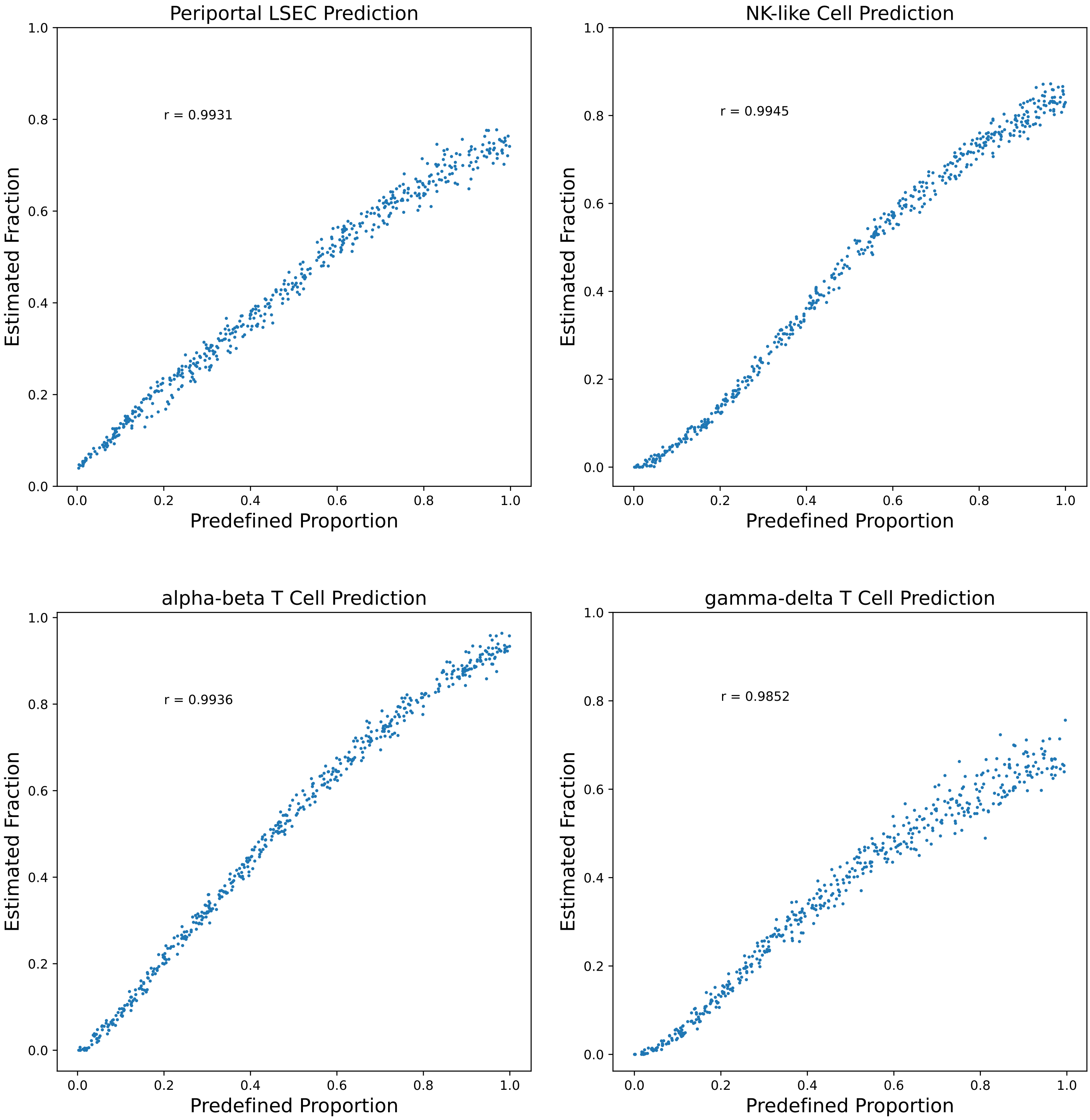

In Silico Validation of Cibersortx – Normal Atlas

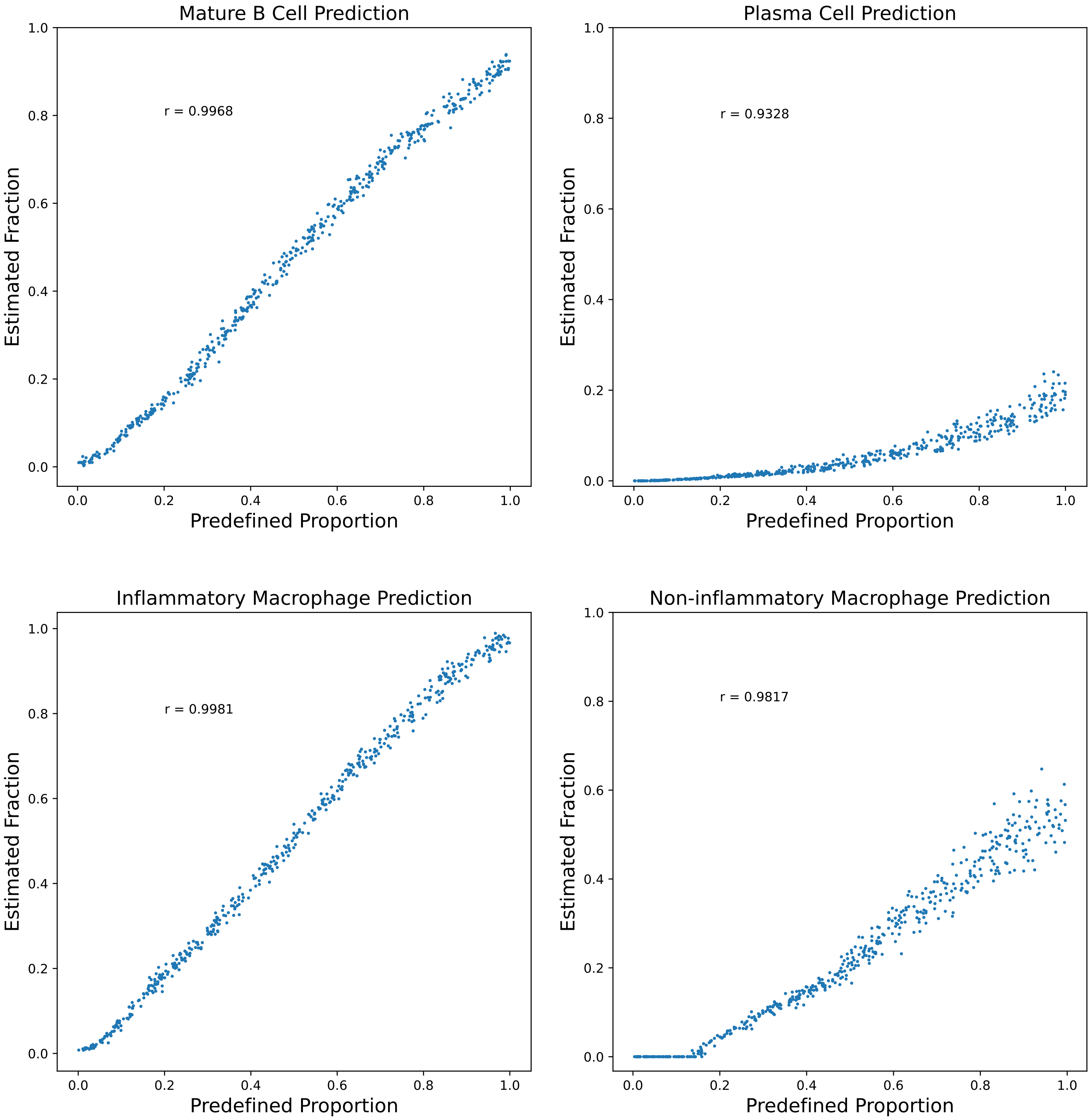

In Silico Validation of Cibersortx – Normal Atlas

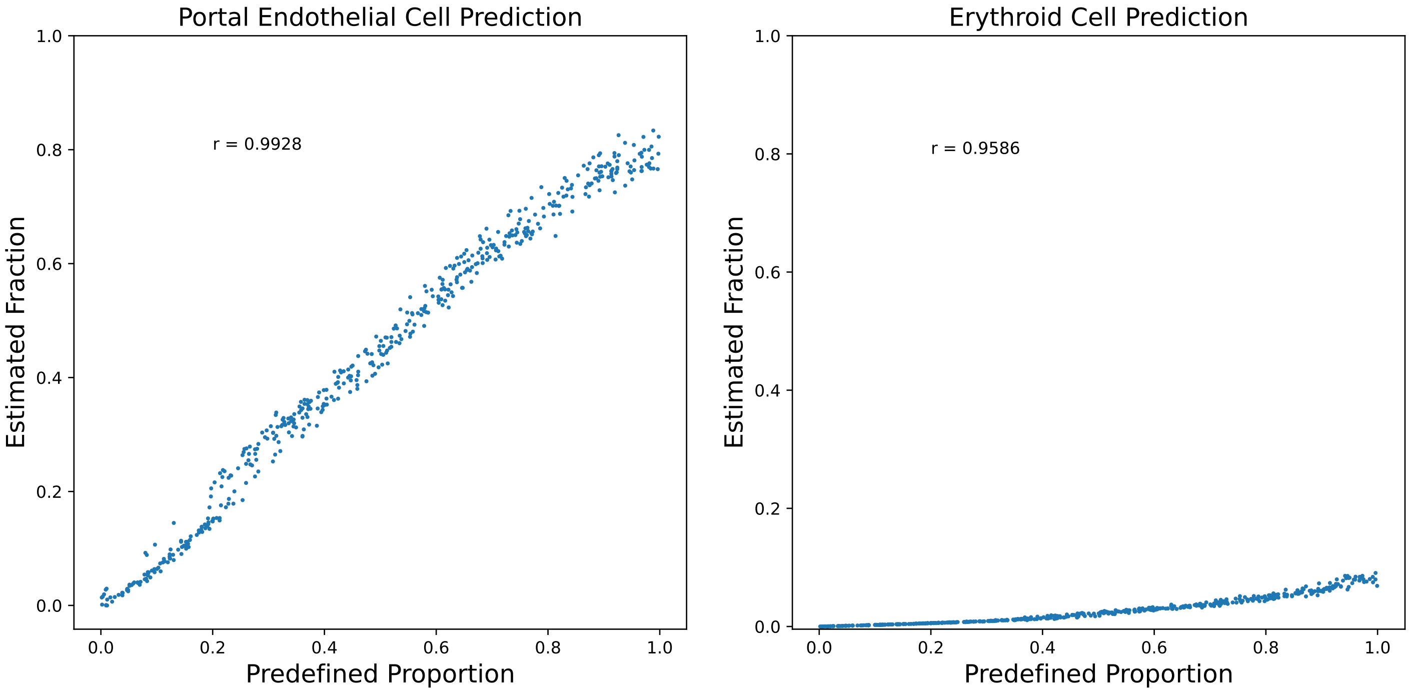

Summary of *In Silico* Validation – Normal Atlas

	Pearson Correlation Coefficient	Mean Absolute Error	Error Propensity
T Cells	0.9863	0.0929	⇩
LSECs	0.993	0.0882	⇩
Cholangiocytes	0.9844	0.1731	⇩
Erythroid Cells	0.9586	0.4574	⇩
Hepatic Stellate Cells	0.986	0.2161	⇩
Hepatocytes	0.992	0.0931	⇧
Macrophage	0.9891	0.0979	⇩
B Cell	0.9544	0.4347	⇩
NK-like Cells	0.9925	0.1177	⇩
Portal Endothelial Cells	0.998	0.1086	⇩

In Silico Validation of Cibersortx – Normal Atlas (Collapsed Subtypes)

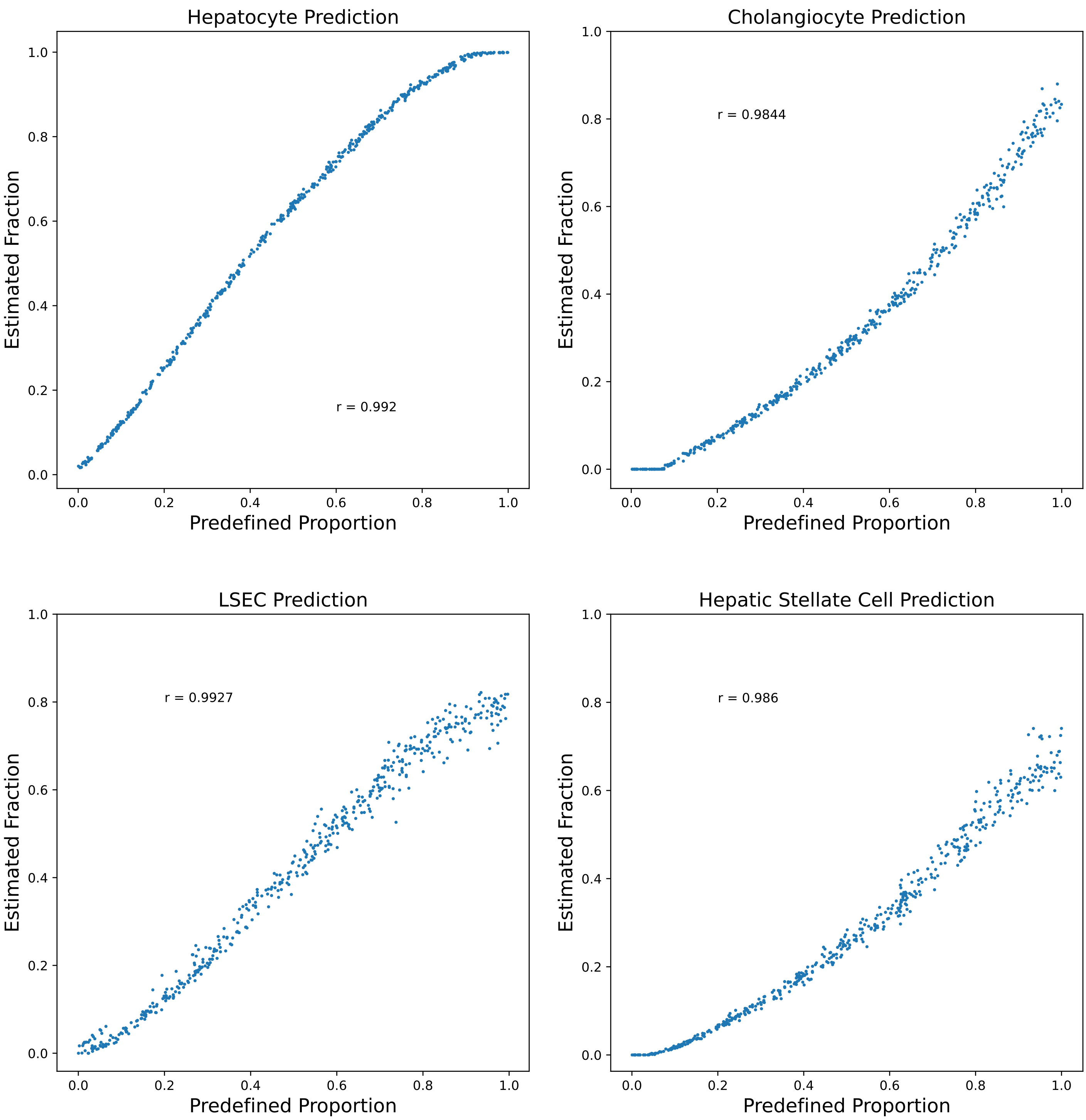

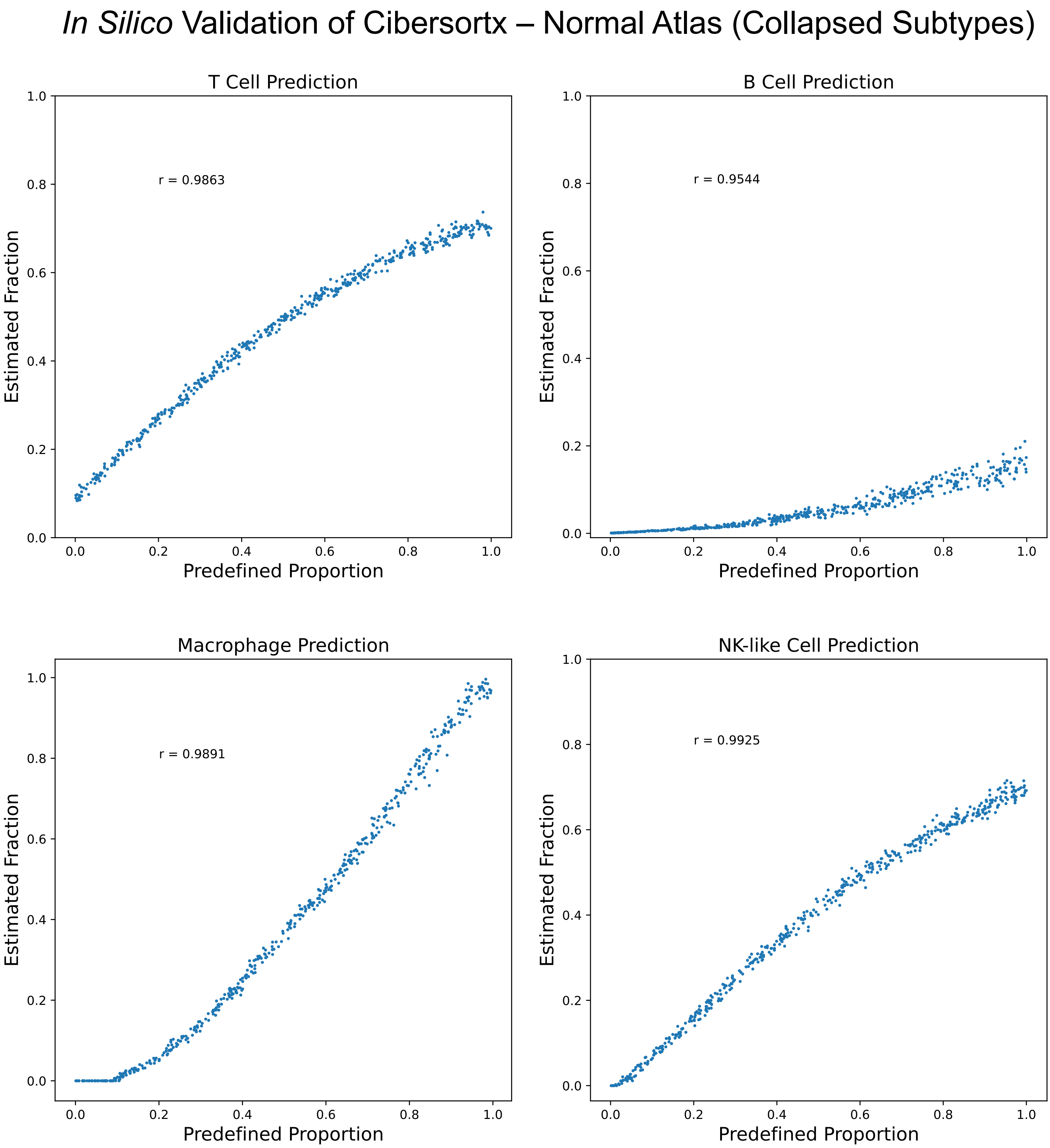
In Silico Validation of Cibersortx – Normal Atlas (Collapsed Subtypes)
T Cell Prediction
r = 0.9863
B Cell Prediction
r = 0.9544
Macrophage Prediction
r = 0.9891
NK-like Cell Prediction
r = 0.9925
Estimated Fraction
Predefined Proportion
0.0
0.2
0.4
0.6
0.8
1.0

In Silico Validation of Cibersortx – Normal Atlas (Collapsed Subtypes)

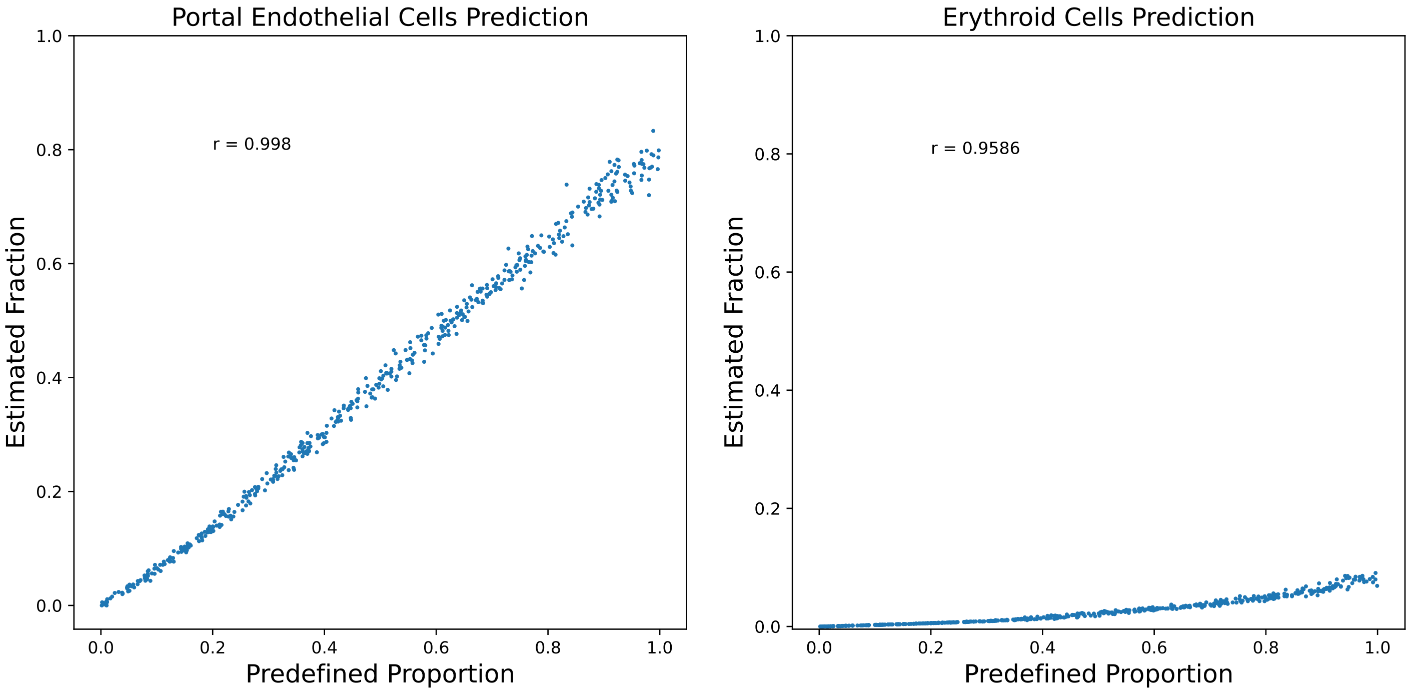

Summary of *In Silico* Validation – TME-Stroma Atlas

	Pearson Correlation Coefficient	Mean Absolute Error	Error Propensity
B cells	0.9884	0.042	⇧
CAFs	0.9798	0.1047	⇩
cDC1	0.9948	0.0361	⇧
cDC2	0.9847	0.1151	⇩
Hepatocytes	0.887	0.2756	⇧
Kupffer cells	0.9922	0.0738	⇩
LSEC	0.977	0.1262	⇩
LVEC	0.9855	0.1278	⇩
LVECt	0.9868	0.1268	⇩
Pericytes	0.972	0.1816	⇩
Proliferation	0.9775	0.0741	⇩
SAMs	0.9885	0.0611	⇧
Stellate cells	0.9715	0.1444	⇩
T cells	0.9793	0.1422	⇩
TM1	0.9834	0.1473	⇩
vSMC	0.984	0.1059	⇩

In Silico Validation of Cibersortx – TME-Stroma Atlas

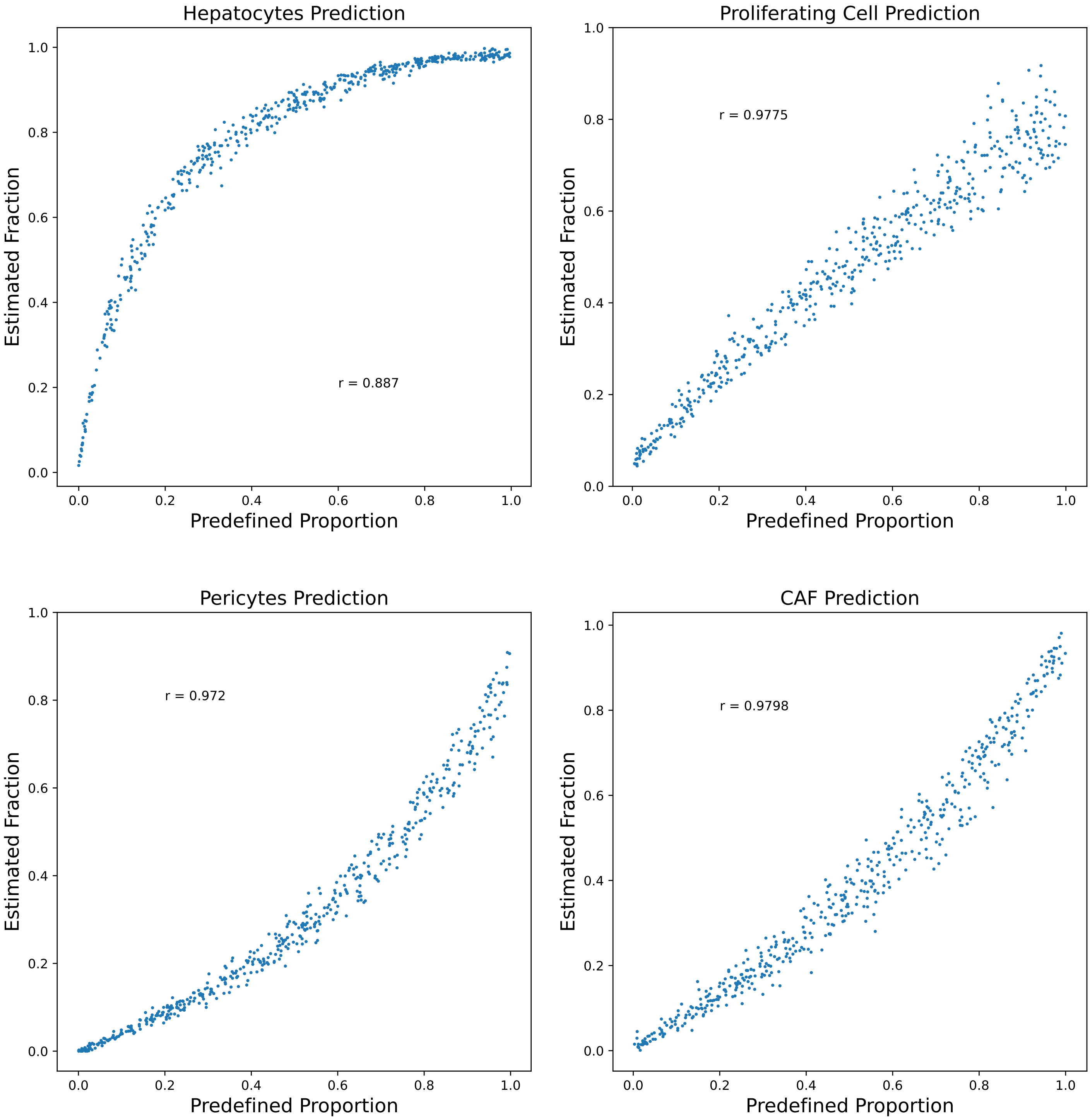

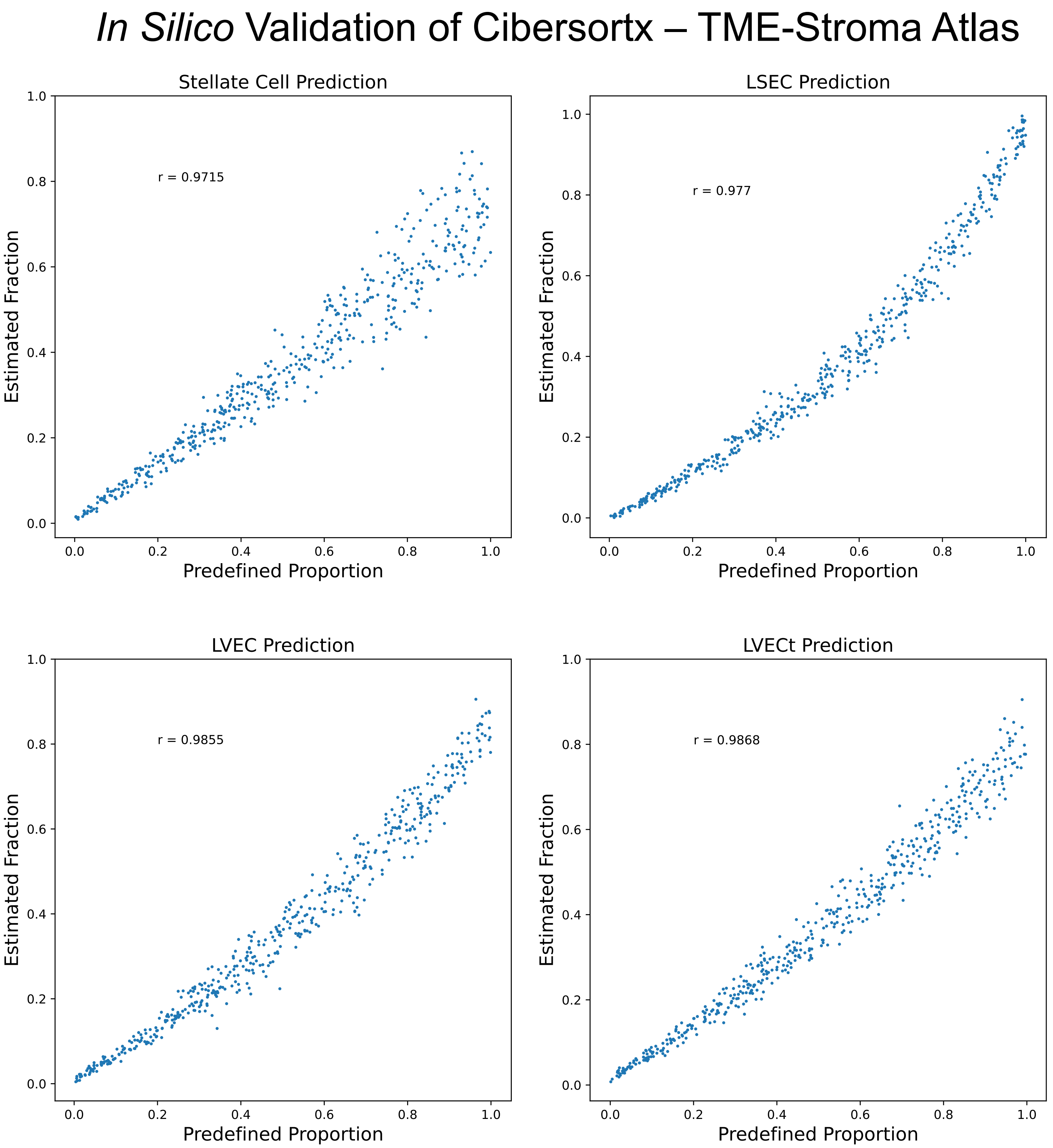
In Silico Validation of Cibersortx – TME-Stroma Atlas
Stellate Cell Prediction
r = 0.9715
LSEC Prediction
r = 0.977
LVEC Prediction
r = 0.9855
LVECt Prediction
r = 0.9868
Estimated Fraction
Predefined Proportion
0.0
0.2
0.4
0.6
0.8
1.0

In Silico Validation of Cibersortx – TME-Stroma Atlas

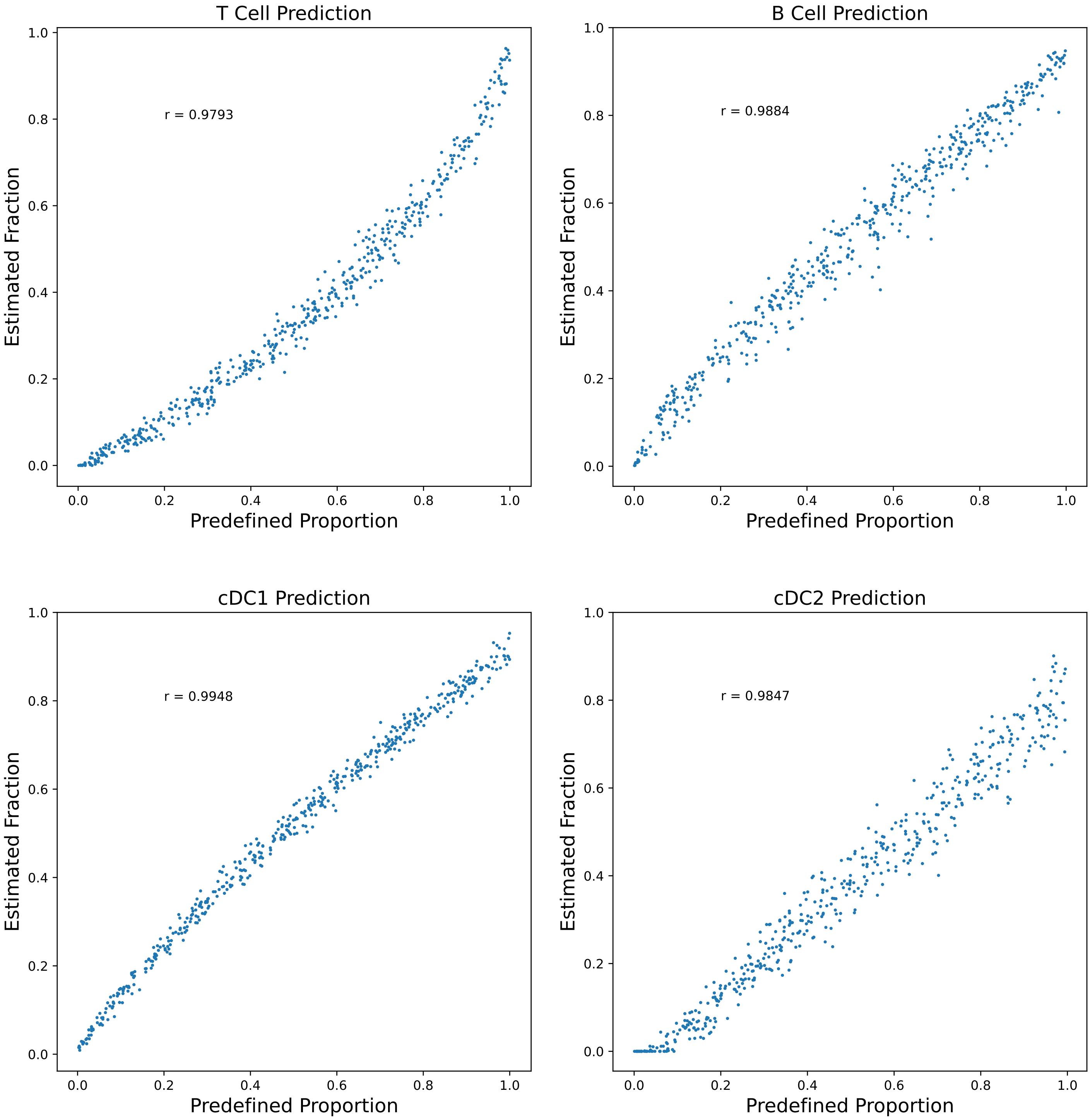

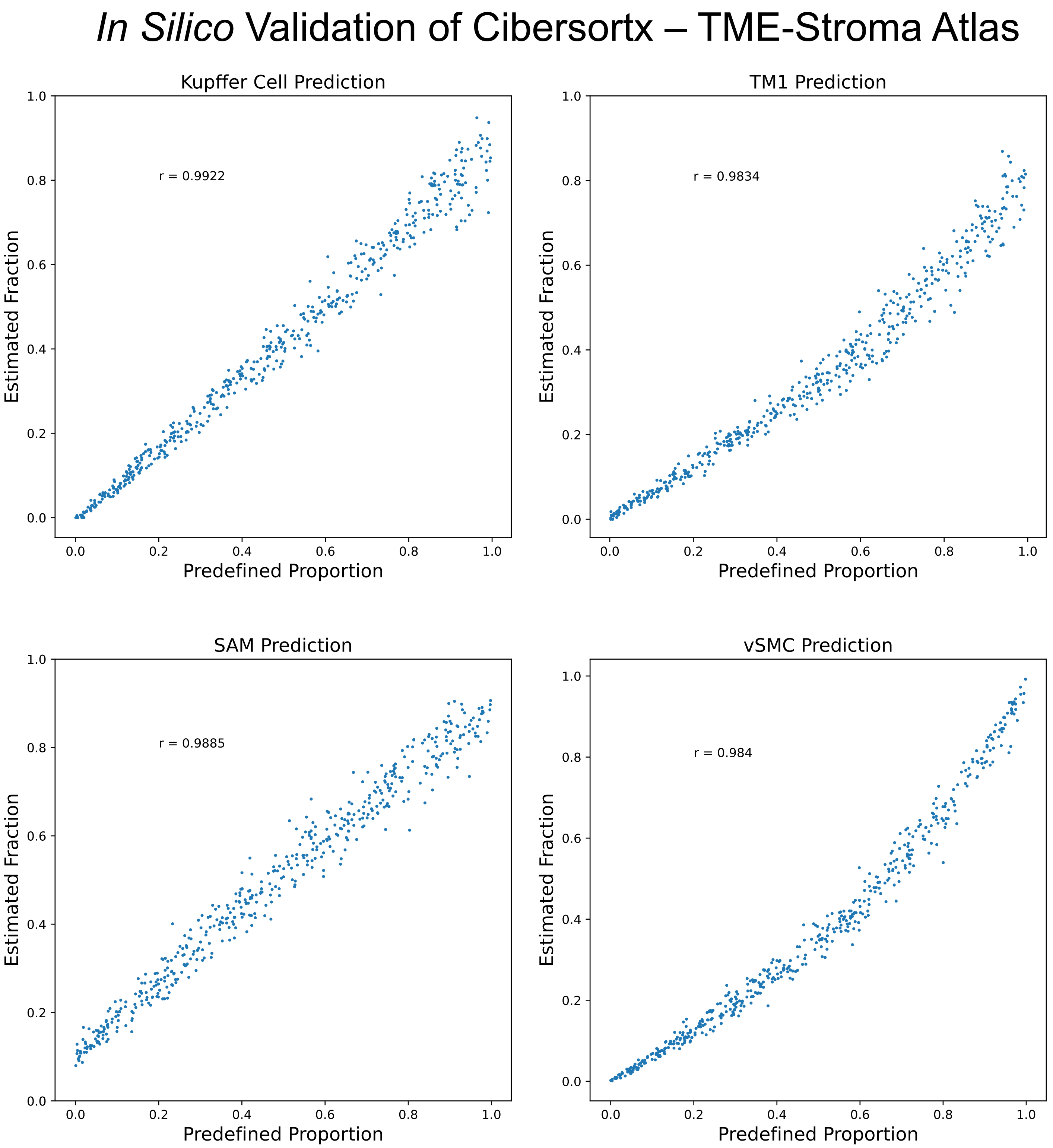
In Silico Validation of Cibersortx – TME-Stroma Atlas
Kupffer Cell Prediction
r = 0.9922
TM1 Prediction
r = 0.9834
SAM Prediction
r = 0.9885
vSMC Prediction
r = 0.984
Estimated Fraction
Predefined Proportion

Summary of *In Silico* Validation – TME-Immune Atlas

	Pearson Correlation Coefficient	Mean Absolute Error	Error Propensity
B Cells	0.99	0.0356	⇧
Bi-Potent Cells	0.9962	0.019	⇩
$CD4^+$ Cells	0.9953	0.1283	⇩
$CD8^+$ Cells	0.9952	0.1261	⇩
T_{regs}	0.9914	0.0847	⇧
Endothelial Cells	0.9996	0.0564	⇩
Fibroblasts	0.9839	0.1091	⇧
Hepatocytes	0.999	0.0872	⇩
Mast Cells	0.9979	0.0928	⇩
Myeloid	0.9956	0.0296	⇧
Nk Cells	0.9986	0.0933	⇩

In Silico Validation of Cibersortx – TME-Immune Atlas

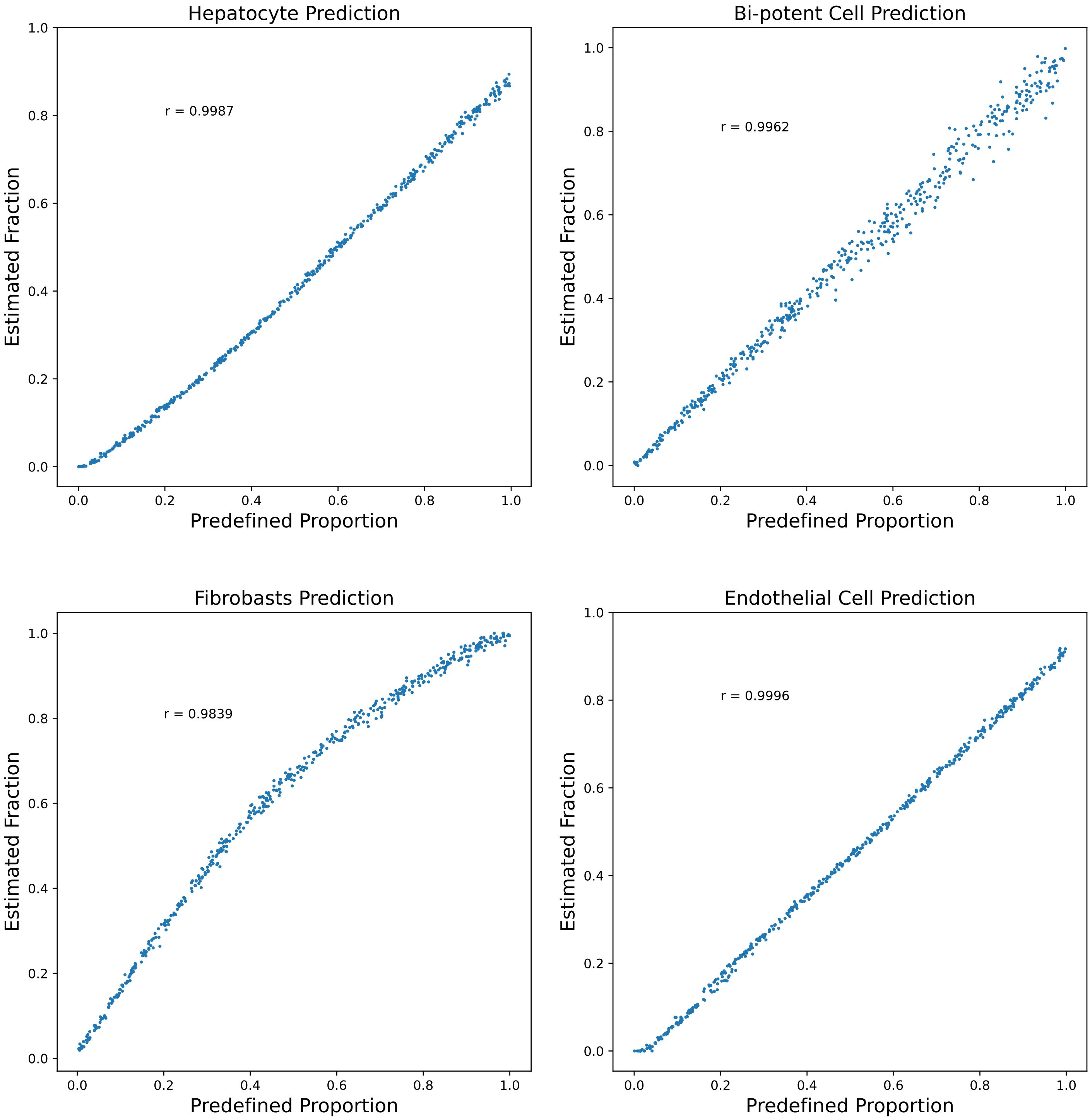

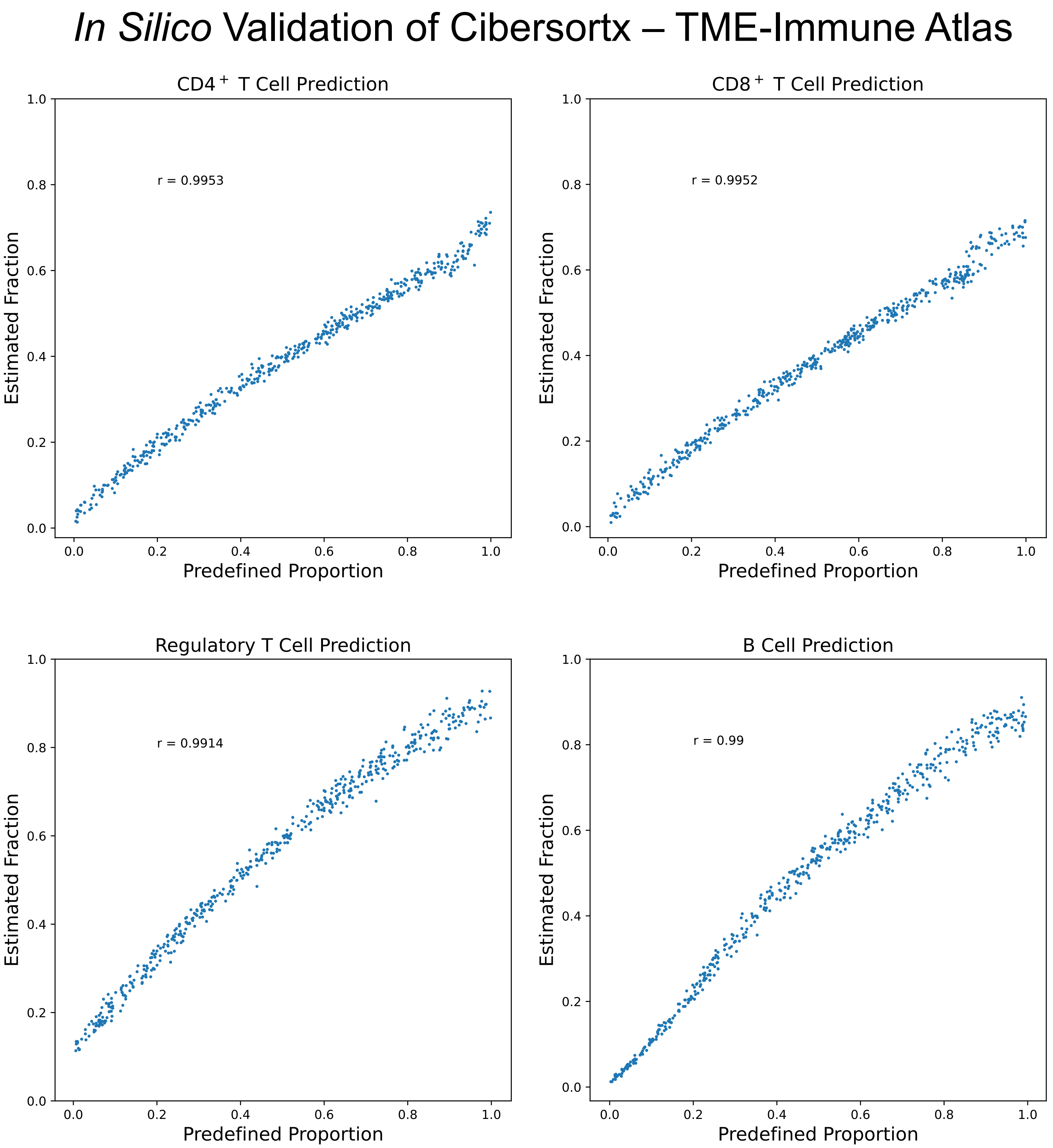
In Silico Validation of Cibersortx – TME-Immune Atlas
CD4$^+$ T Cell Prediction
r = 0.9953
CD8$^+$ T Cell Prediction
r = 0.9952
Regulatory T Cell Prediction
r = 0.9914
B Cell Prediction
r = 0.99
Estimated Fraction
Predefined Proportion

In Silico Validation of Cibersortx – TME-Immune Atlas

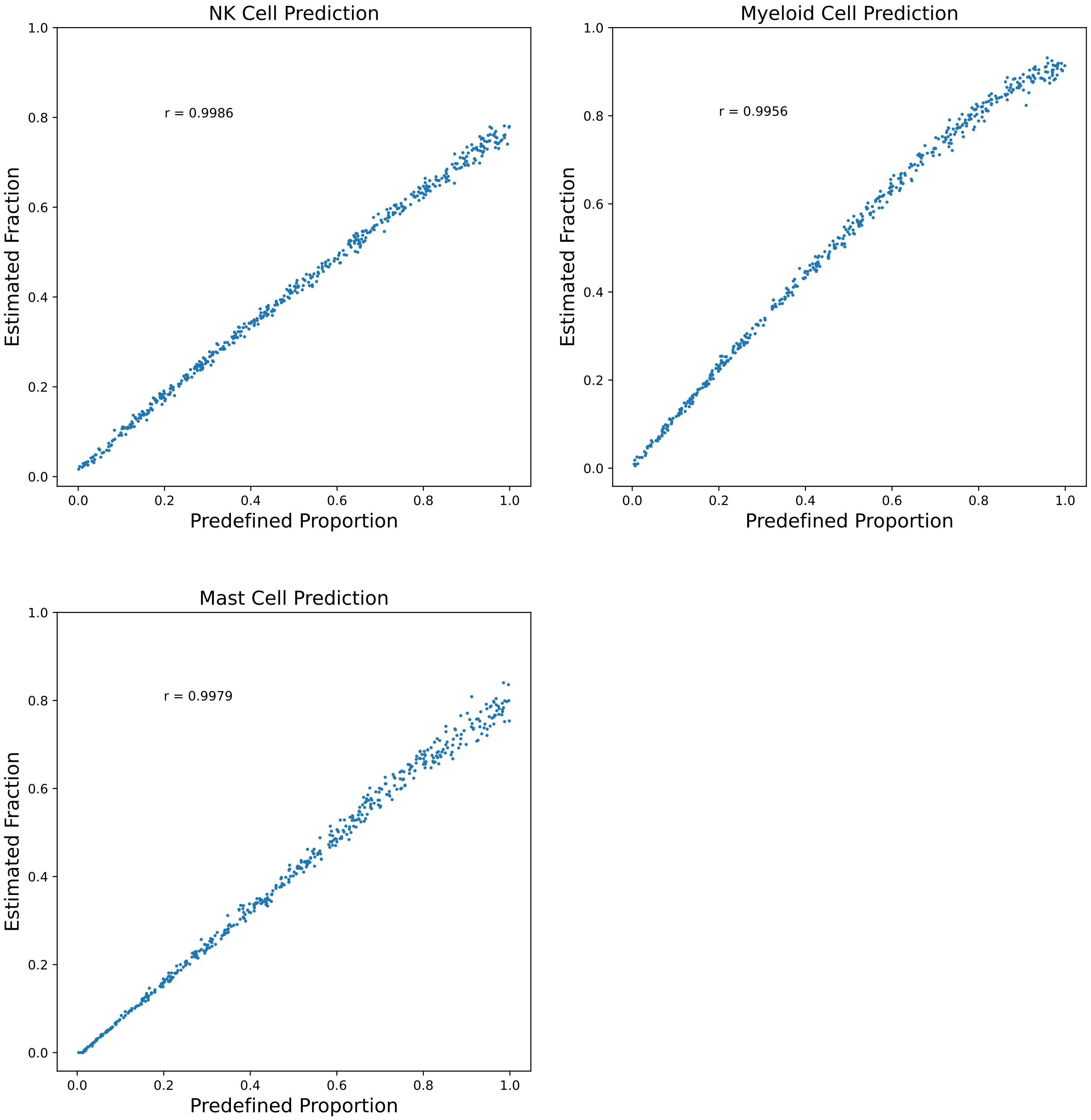

Summary of Cross-study Validation

Cell type (Reference atlas)	Pseudobulk (Atlas)	Pearson Correlation Coefficient	Mean Absolute Error	Error Propensity
Hepatocytes (GSE115469)	Hepatocytes (GSE146409)	0.963	0.0748	⇧
Hepatocytes (GSE115469)	Hepatocytes (GSE156337)	0.9964	0.208	⇩
Hepatocytes (GSE146409)	Hepatocytes (GSE115469)	0.9993	0.2514	⇩
Hepatocytes (GSE146409)	Hepatocytes (GSE156337)	0.9903	0.4135	⇩
Hepatocytes (GSE156337)	Hepatocytes (GSE115469)	0.9981	0.0601	⇩
Hepatocytes (GSE156337)	Hepatocytes (GSE146409)	0.9486	0.1071	⇧

Cell type (Reference atlas)	Pseudobulk (Atlas)	Pearson Correlation Coefficient	Mean Absolute Error	Error Propensity
B Cells (GSE115469, Mature B Cells + Plasma B Cells)	B Cells (GSE146409)	0.9838	0.1907	⇩
B Cells (GSE115469, Mature B Cells + Plasma B Cells)	B Cells (GSE156337)	0.9975	0.2249	⇩
B Cells (GSE146409)	B Cells (GSE115469, Mature B Cells + Plasma B Cells)	0.9799	0.4078	⇩
B Cells (GSE146409)	B Cells (GSE156337)	0.9953	0.3854	⇩
B Cells (GSE156337)	B Cells (GSE115469, Mature B Cells + Plasma B Cells)	0.9803	0.4065	⇩
B Cells (GSE156337)	B Cells (GSE146409)	0.9498	0.3766	⇩

Cell type (Reference atlas)	Pseudobulk (Atlas)	Pearson Correlation Coefficient	Mean Absolute Error	Error Propensity
T Cells (GSE115469 alpha-beta + gamma-delta T cells)	T Cells (GSE146409)	0.9985	0.0884	⇩
T Cells (GSE115469 alpha-beta + gamma-delta T cells)	T Cells (GSE156337, $CD4^+$ + $CD8^+$ + T_{reg})	0.9986	0.0879	⇩
T Cells (GSE146409)	T Cells (GSE115469 alpha-beta + gamma-delta T cells)	0.9936	0.3092	⇩
T Cells (GSE146409)	T Cells (GSE156337, $CD4^+$ + $CD8^+$ + T_{reg})	0.9977	0.2352	⇩
T Cells (GSE156337, $CD4^+$ + $CD8^+$ + T_{reg})	T Cells (GSE115469 alpha-beta + gamma-delta T cells)	0.9799	0.3099	⇩
T Cells (GSE156337, $CD4^+$ + $CD8^+$ + T_{reg})	T Cells (GSE146409)	0.946	0.2875	⇩

Cell type (Reference atlas)	Pseudobulk (Atlas)	Pearson Correlation Coefficient	Mean Absolute Error	Error Propensity
Hepatic Stellate Cells (GSE115469)	Stellate Cells (GSE146409)	0.9685	0.3595	⇩
Stellate Cells (GSE146409)	Hepatic Stellate Cells (GSE115469)	0.9741	0.3896	⇩
LSEC (GSE115469)	LSEC (GSE146409)	0.9698	0.2339	⇩
LSEC (GSE146409)	LSEC (GSE115469)	0.9458	0.3989	⇩
Nk-like Cells (GSE115469)	NK Cells (GSE156337)	0.9619	0.4516	⇩
NK Cells (GSE156337)	Nk-like Cells (GSE115469)	0.9908	0.2223	⇩
CAFs (GSE146409)	Fibroblasts (GSE156337)	0.7238	0.4811	⇩
Fibroblasts (GSE156337)	CAFs (GSE146409)	0.9636	0.2564	⇩

In Silico Validation of Cibersortx – Cross-study Validation

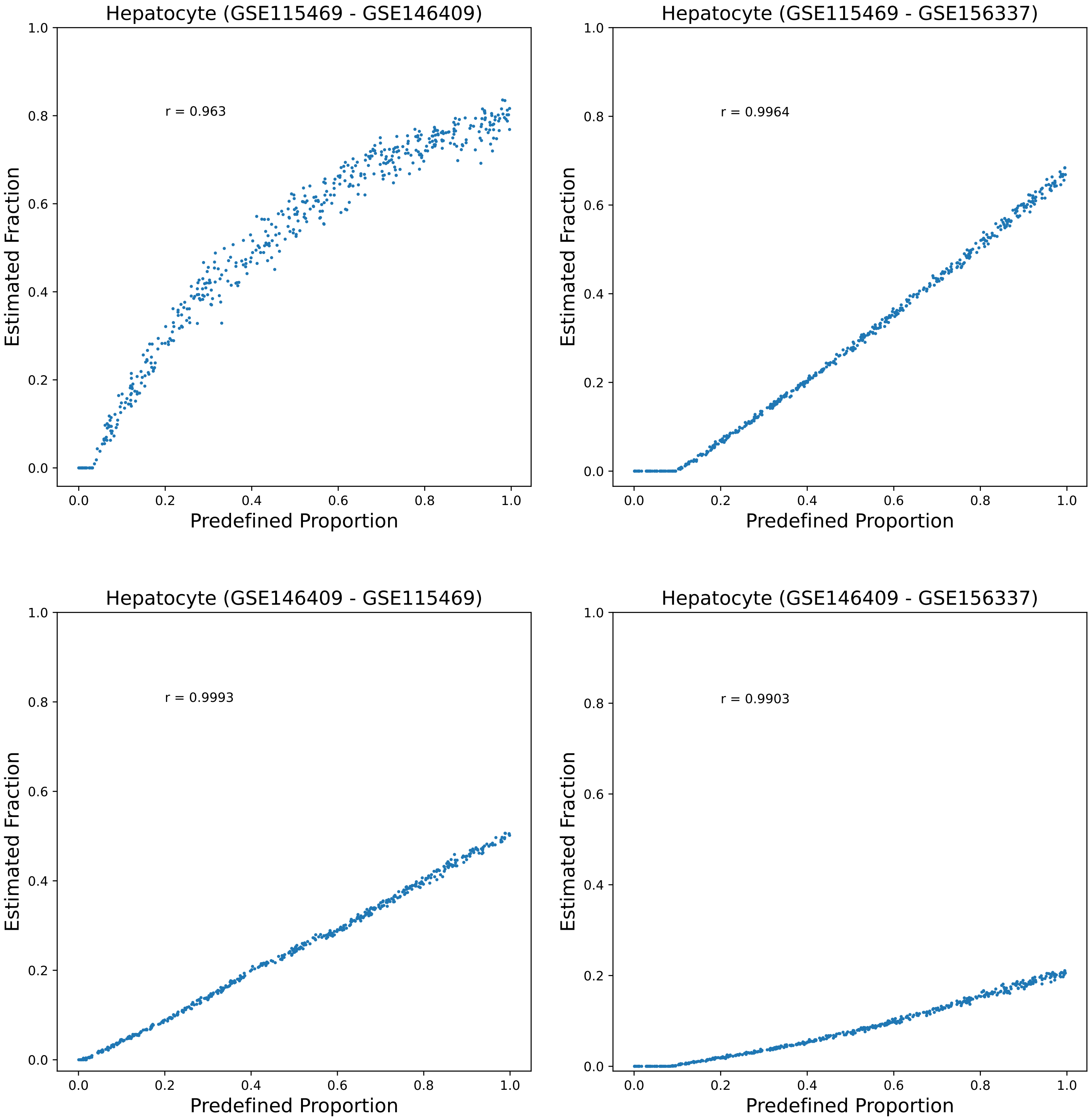

In Silico Validation of Cibersortx – Cross-study Validation

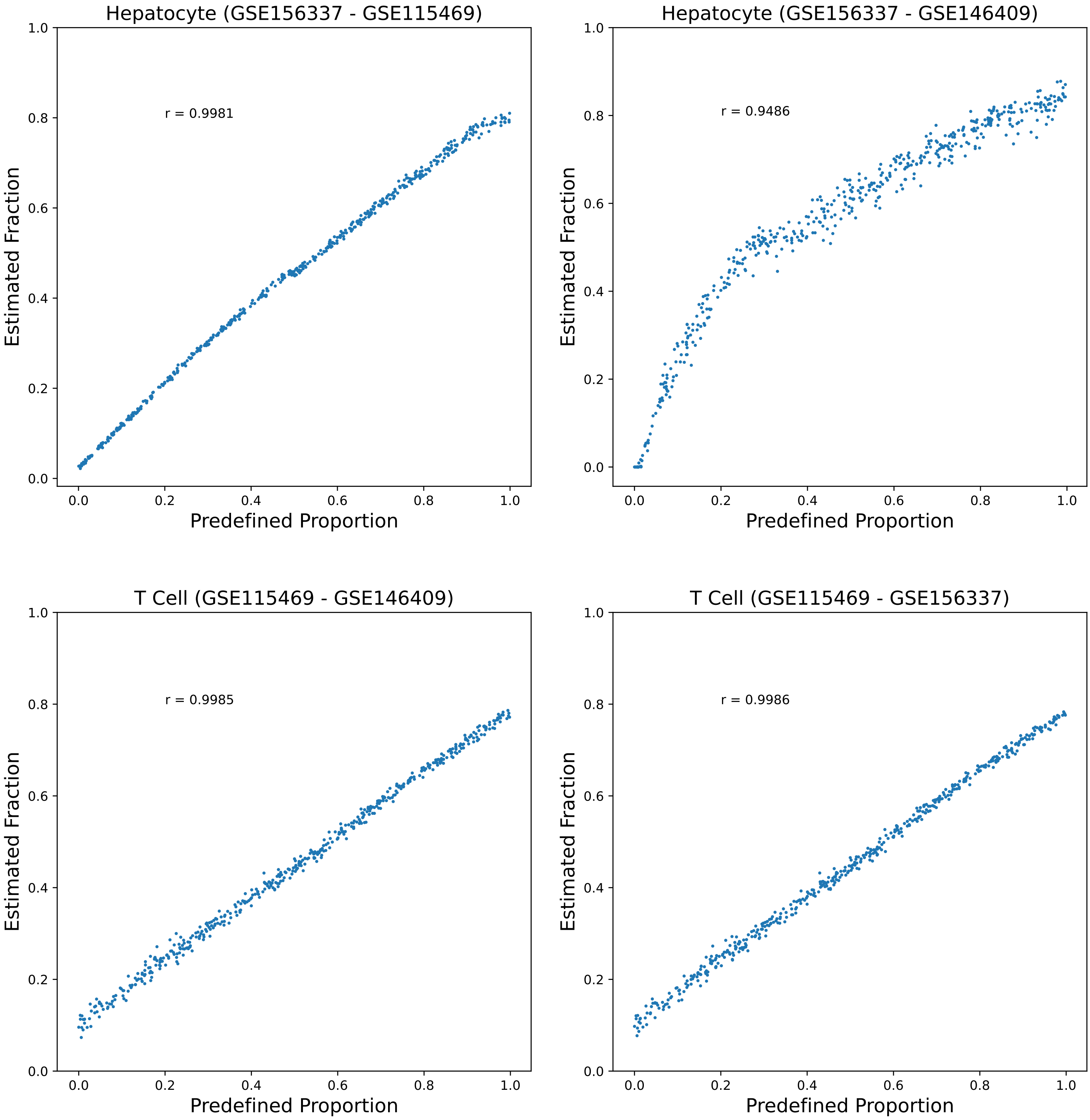

In Silico Validation of Cibersortx – Cross-study Validation

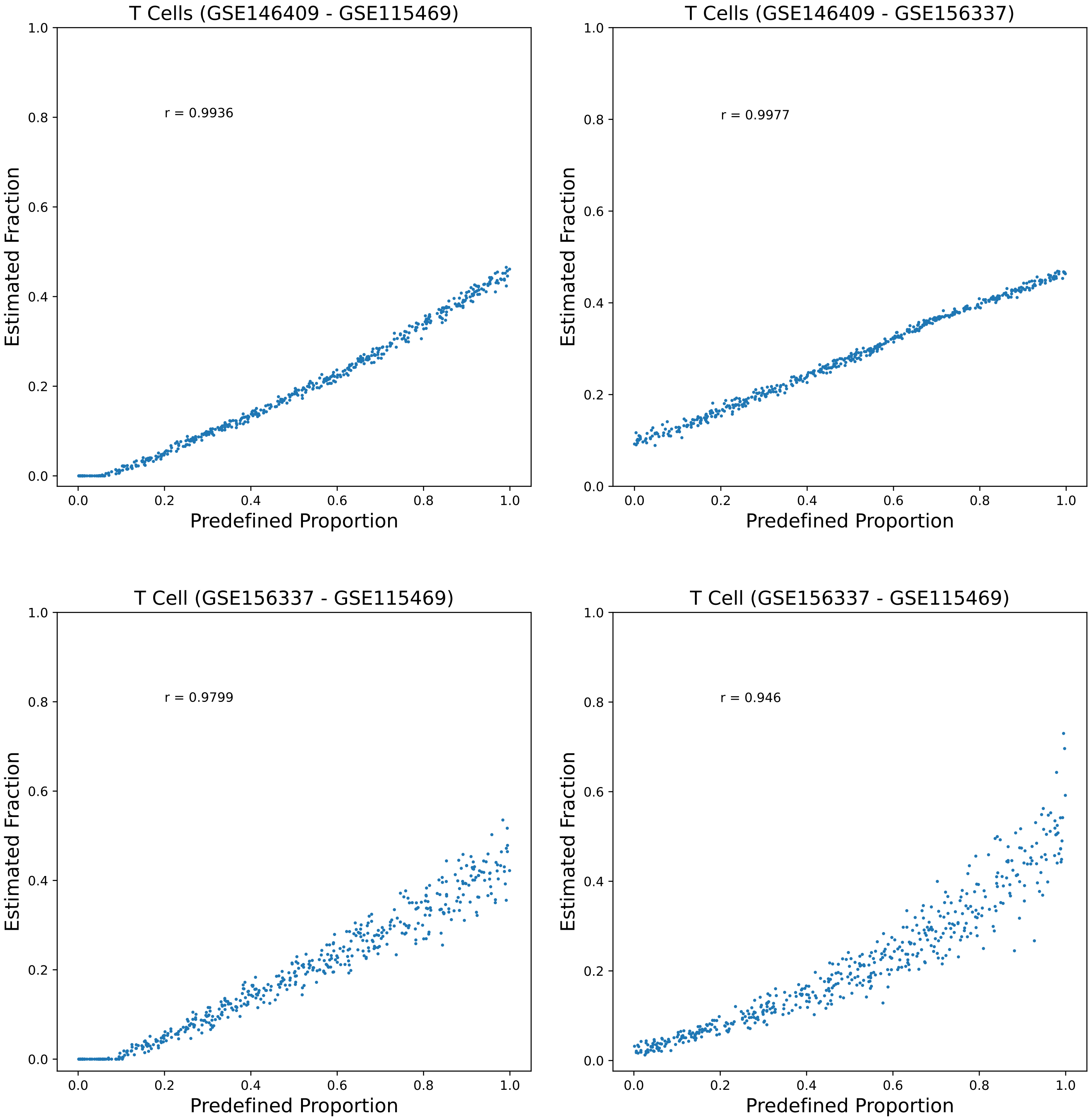

In Silico Validation of Cibersortx – Cross-study Validation

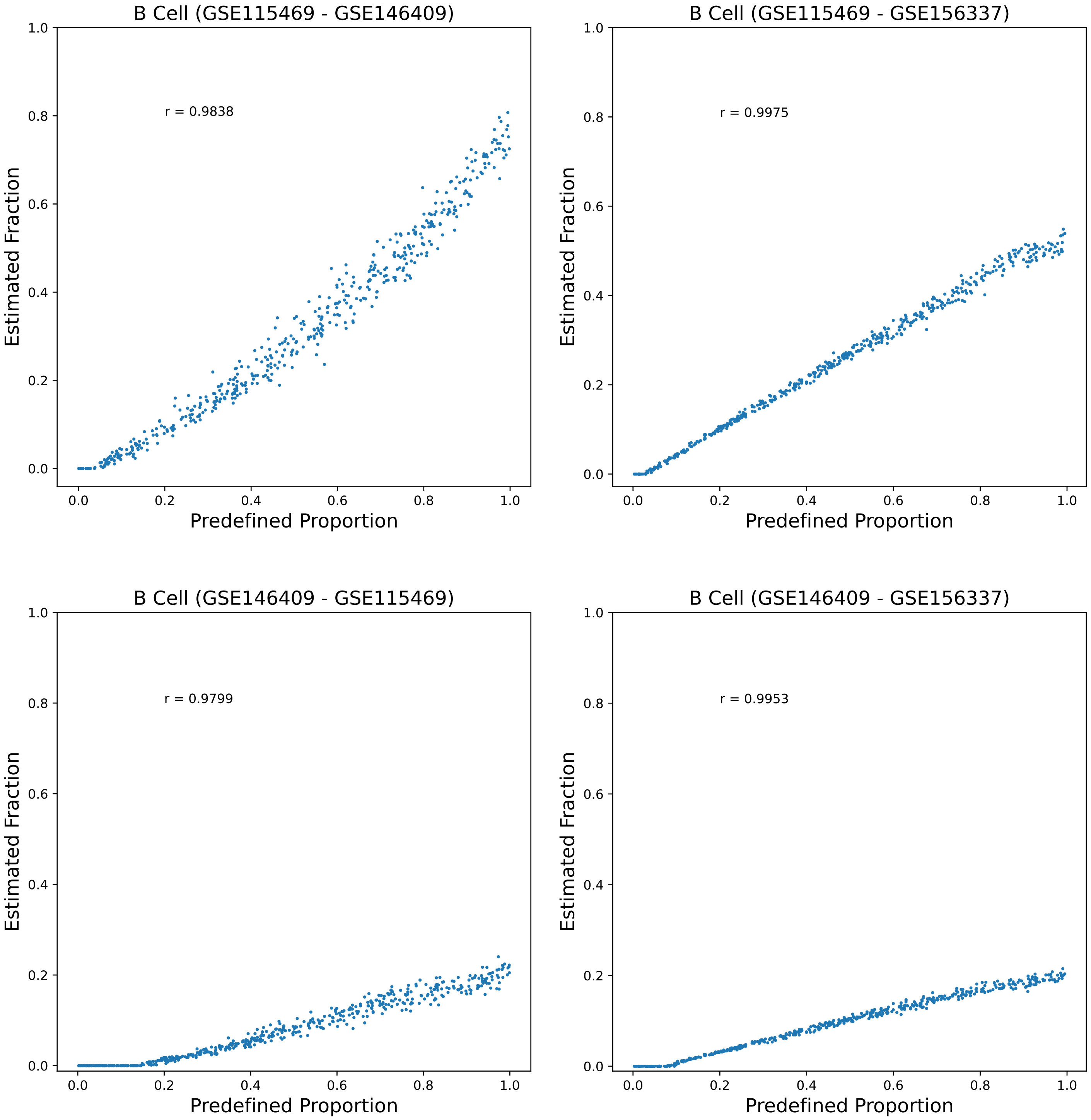

In Silico Validation of Cibersortx – Cross-study Validation

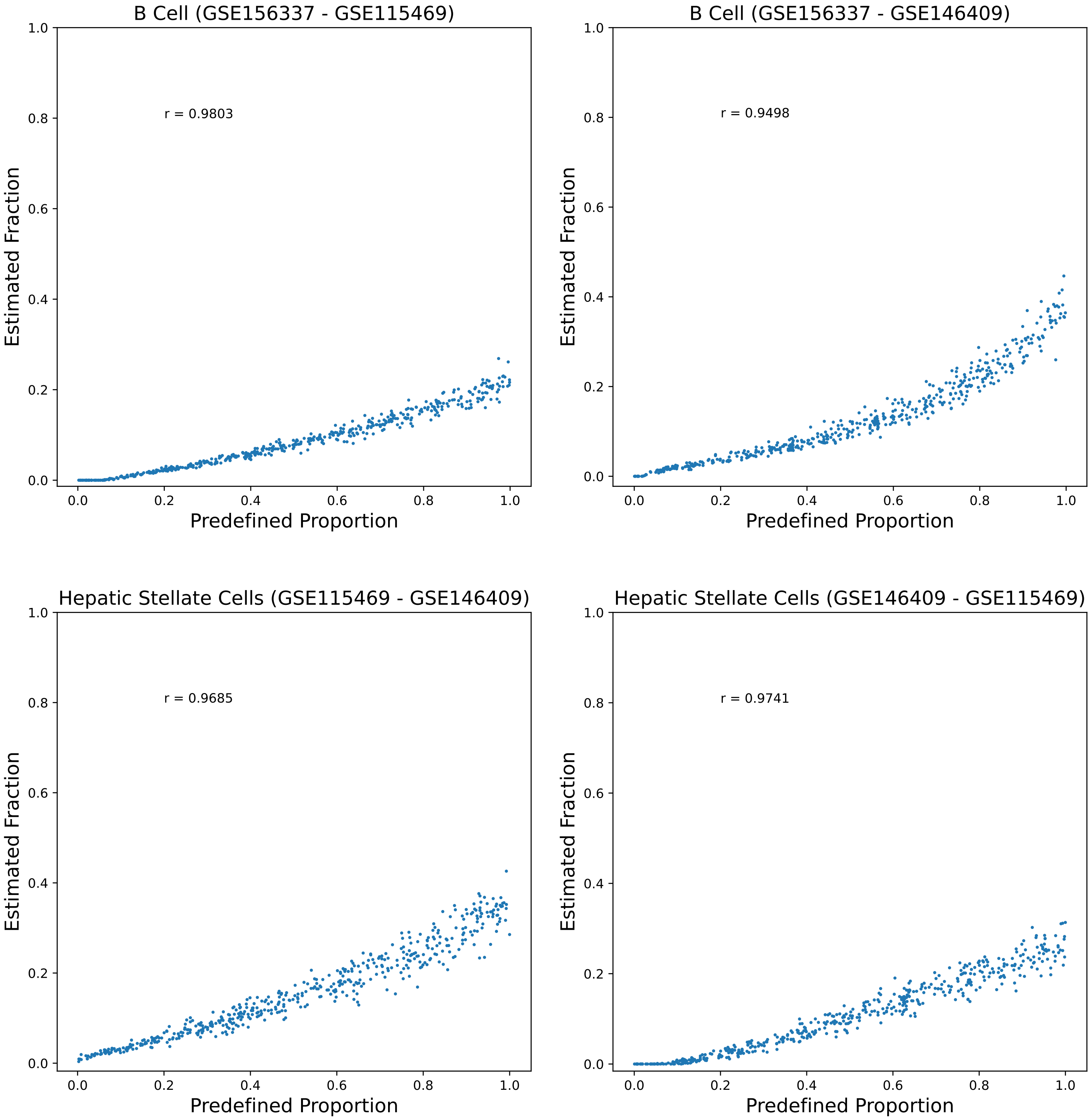

In Silico Validation of Cibersortx – Cross-study Validation

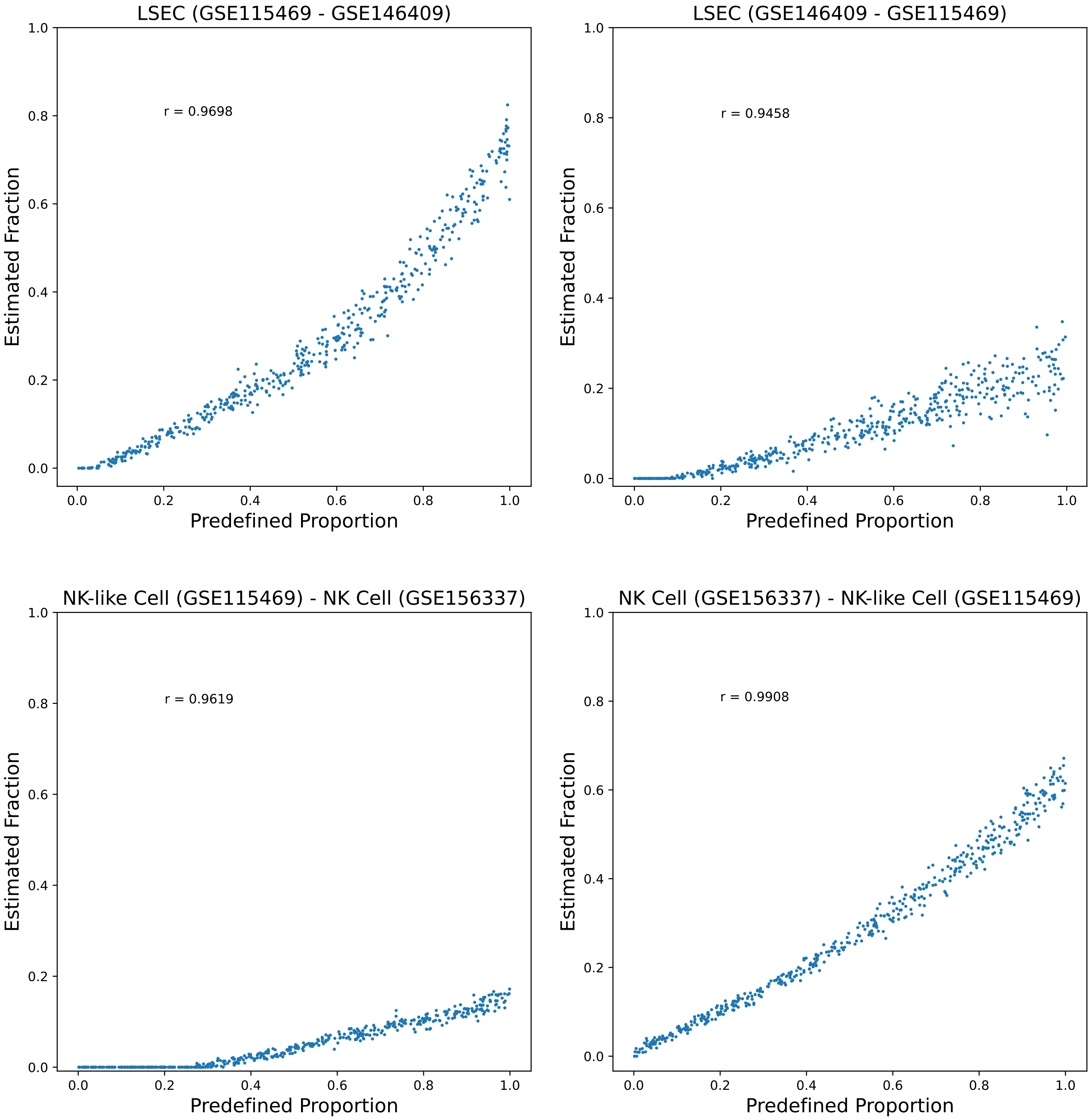

In Silico Validation of Cibersortx – Cross-study Validation

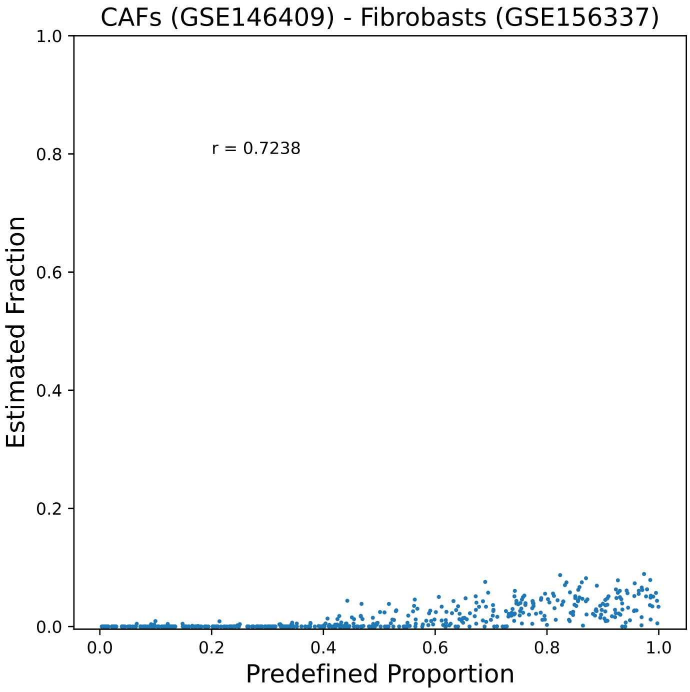

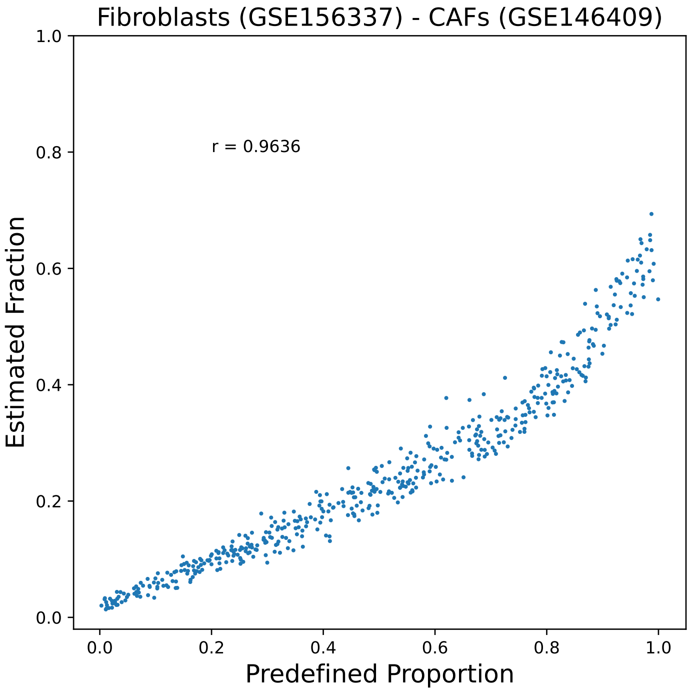

In Silico Validation of Support Vector Regression – Normal Atlas

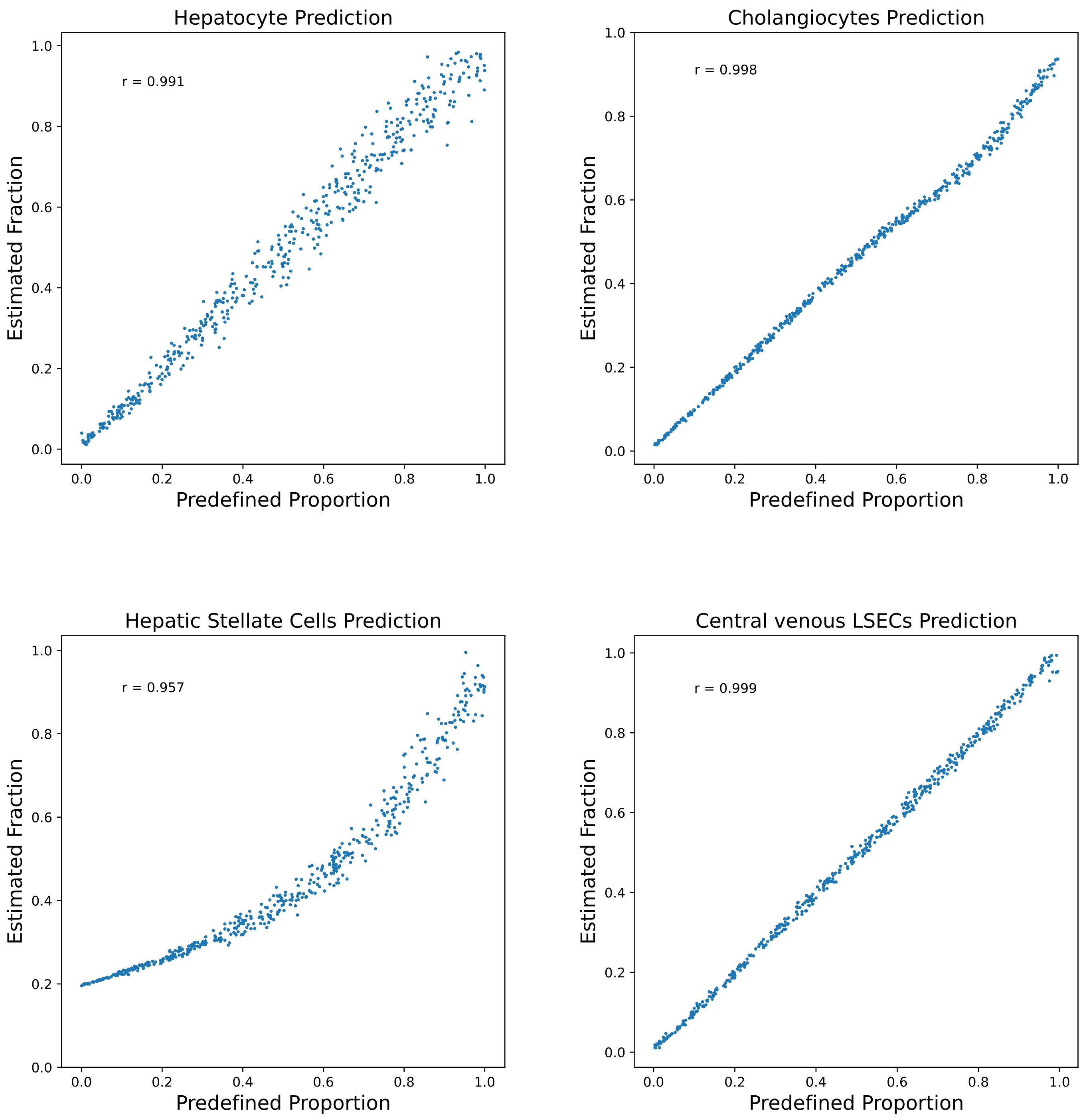

In Silico Validation of Support Vector Regression – Normal Atlas

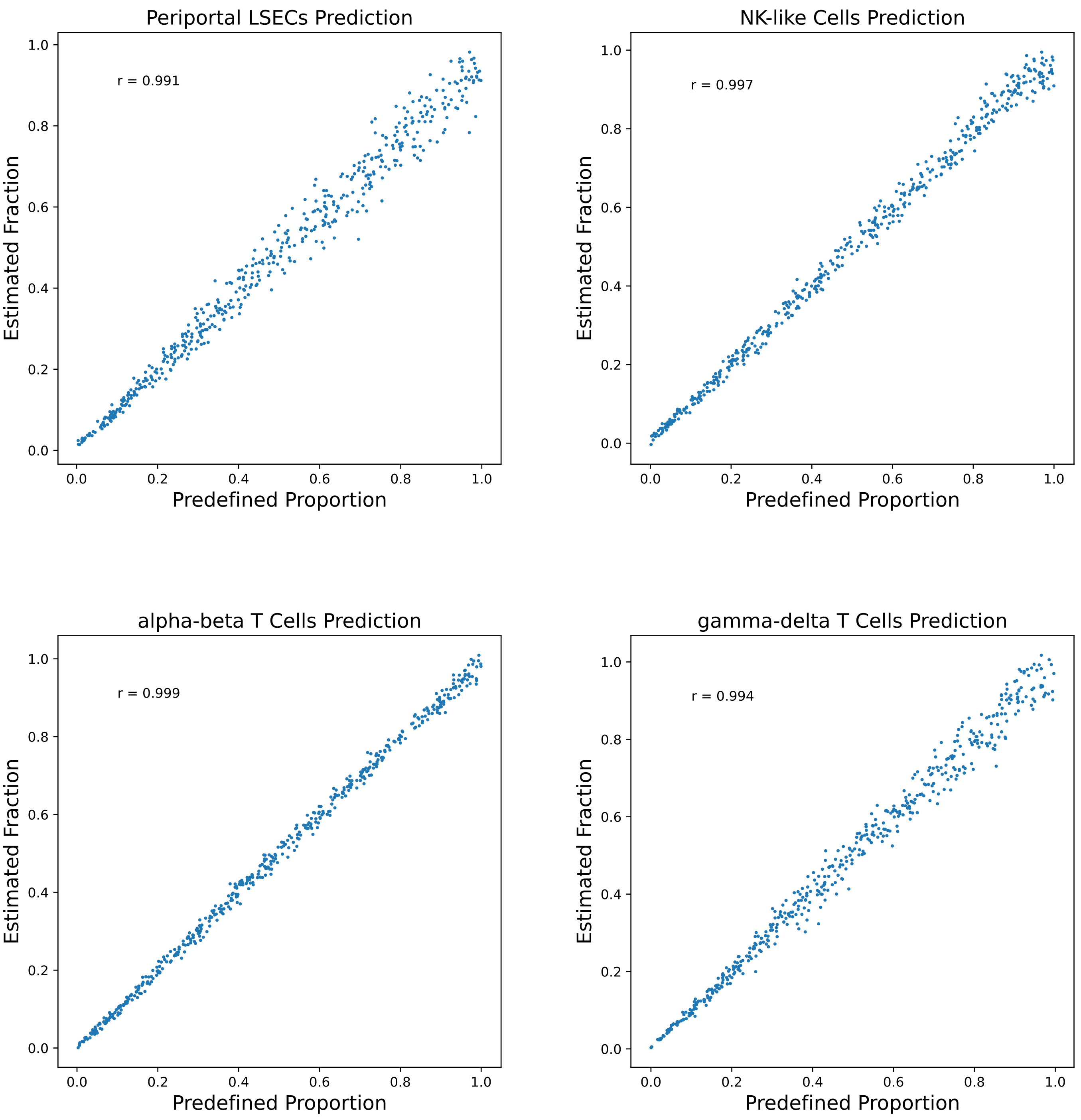

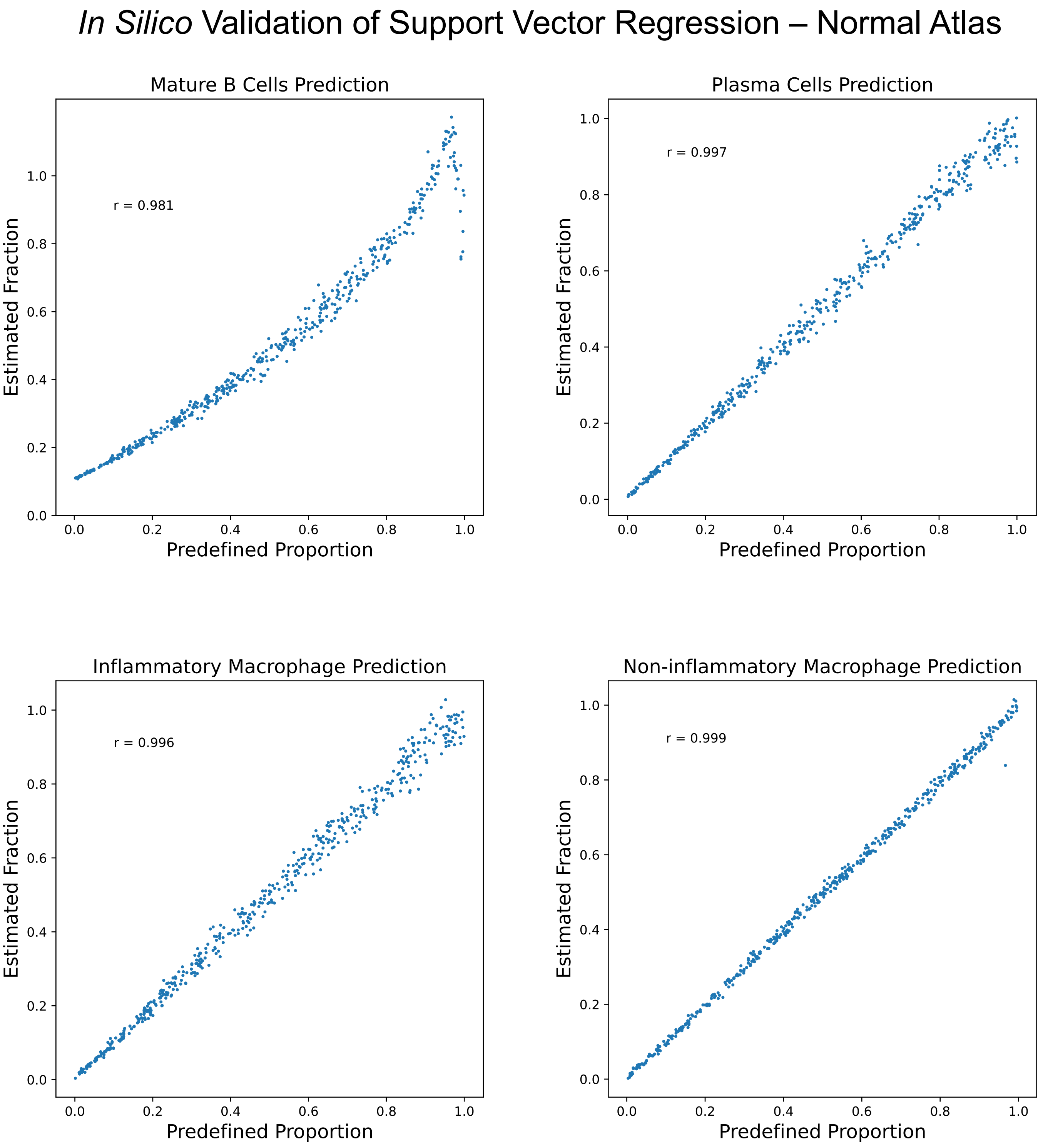
In Silico Validation of Support Vector Regression – Normal Atlas
Mature B Cells Prediction
r = 0.981
Plasma Cells Prediction
r = 0.997
Inflammatory Macrophage Prediction
r = 0.996
Non-inflammatory Macrophage Prediction
r = 0.999
Estimated Fraction
Predefined Proportion
0.0
0.2
0.4
0.6
0.8
1.0

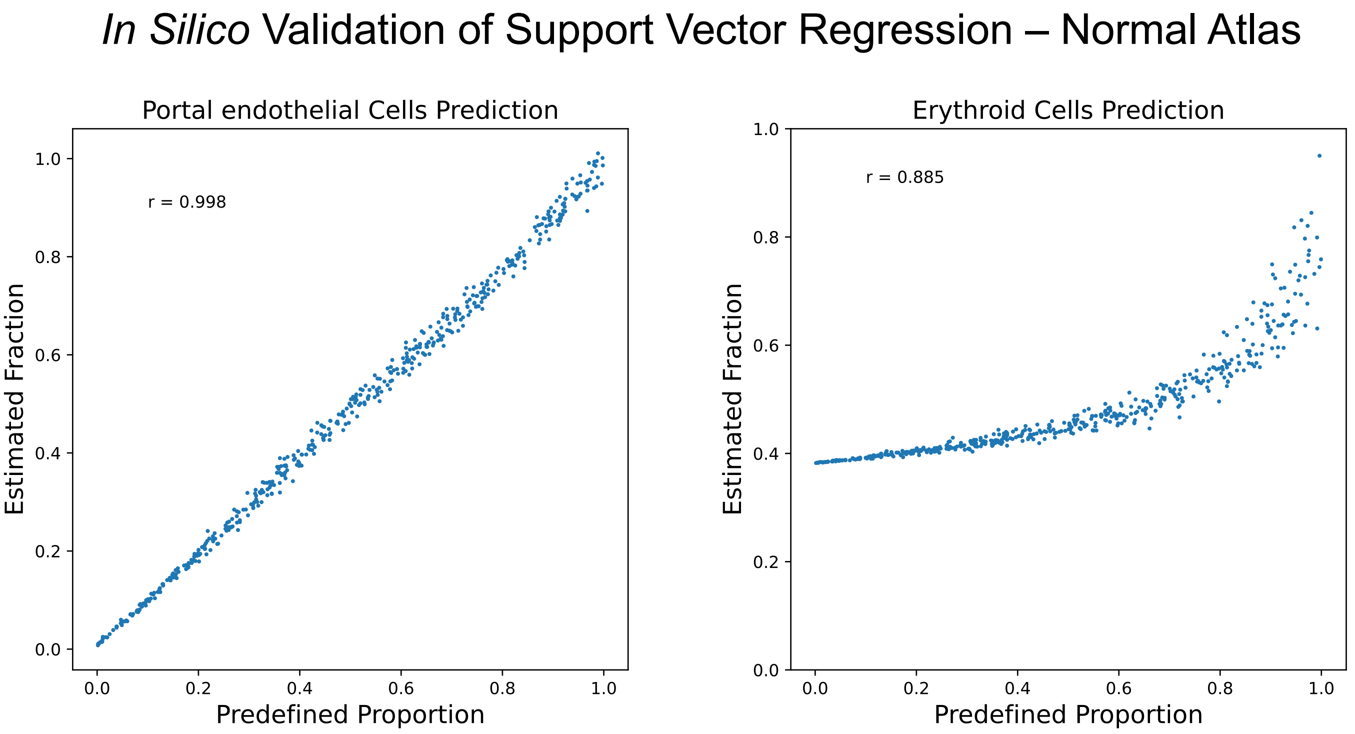
In Silico Validation of Support Vector Regression – Normal Atlas
Portal endothelial Cells Prediction
r = 0.998
Estimated Fraction
Predefined Proportion
0.0
0.2
0.4
0.6
0.8
1.0
Erythroid Cells Prediction
r = 0.885
Estimated Fraction
Predefined Proportion

In Silico Validation of Support Vector Regression – TME-Stroma Atlas

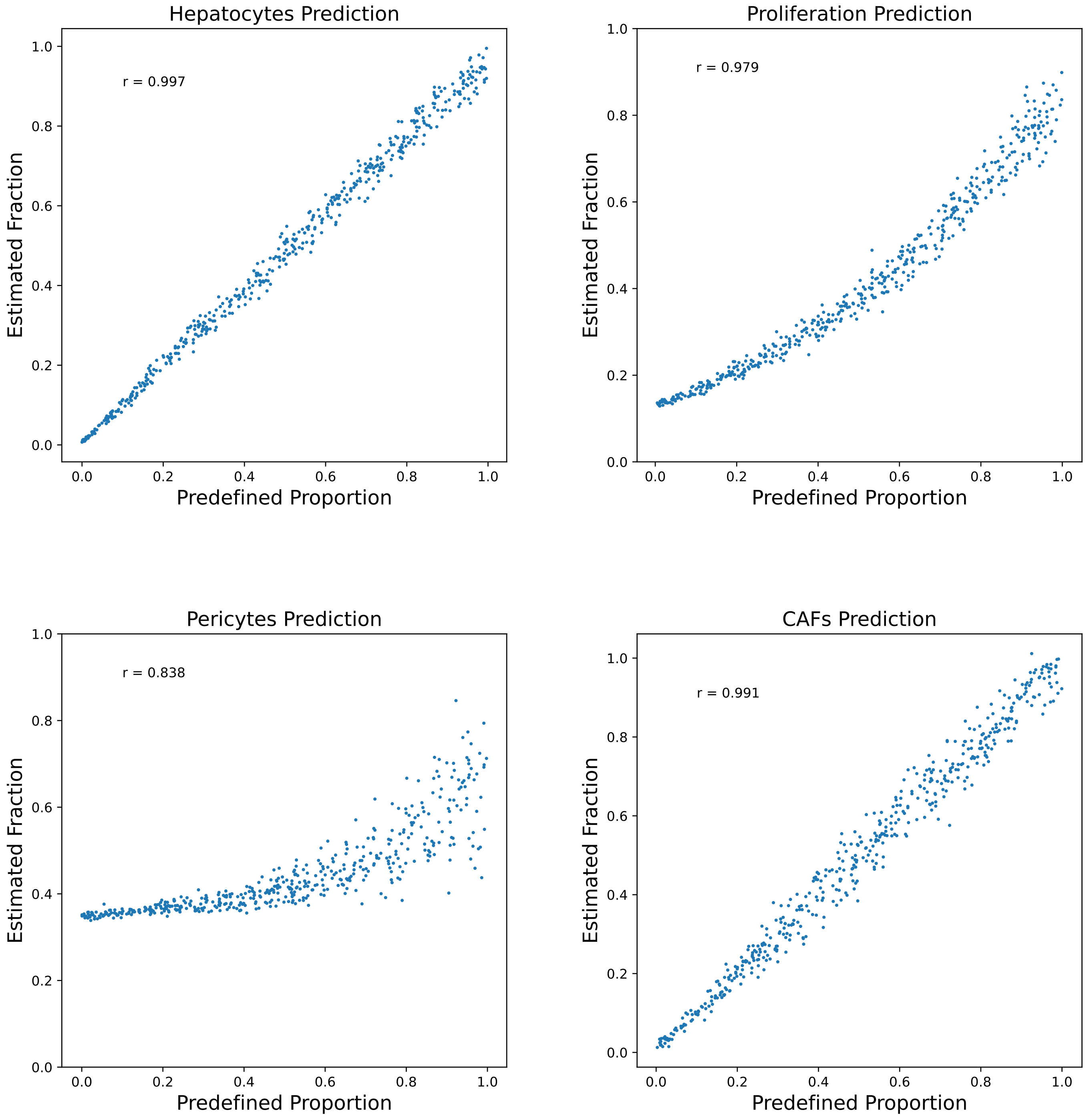

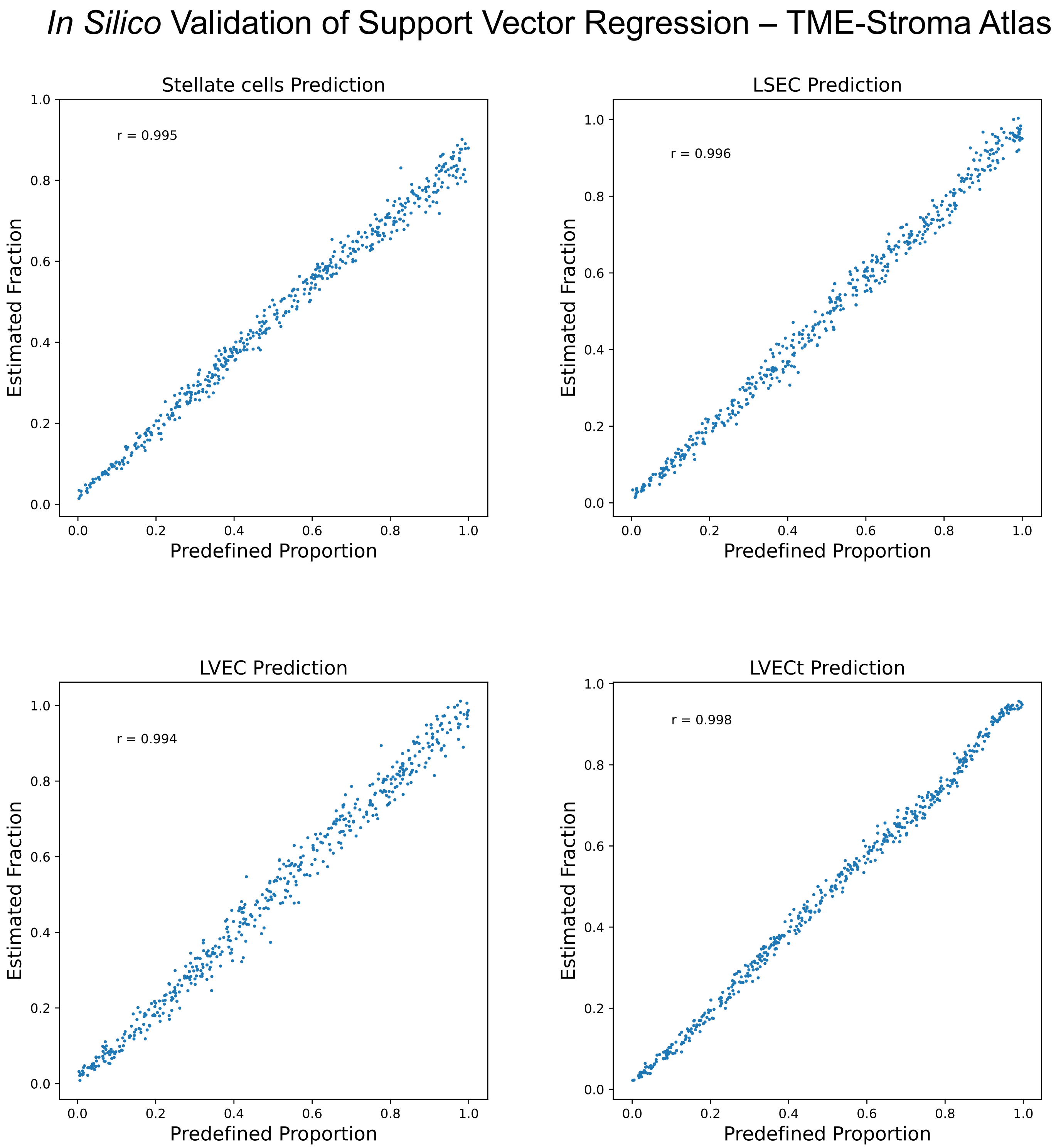
In Silico Validation of Support Vector Regression – TME-Stroma Atlas
Stellate cells Prediction
r = 0.995
LSEC Prediction
r = 0.996
LVEC Prediction
r = 0.994
LVECt Prediction
r = 0.998
Estimated Fraction
Predefined Proportion
0.0
0.2
0.4
0.6
0.8
1.0

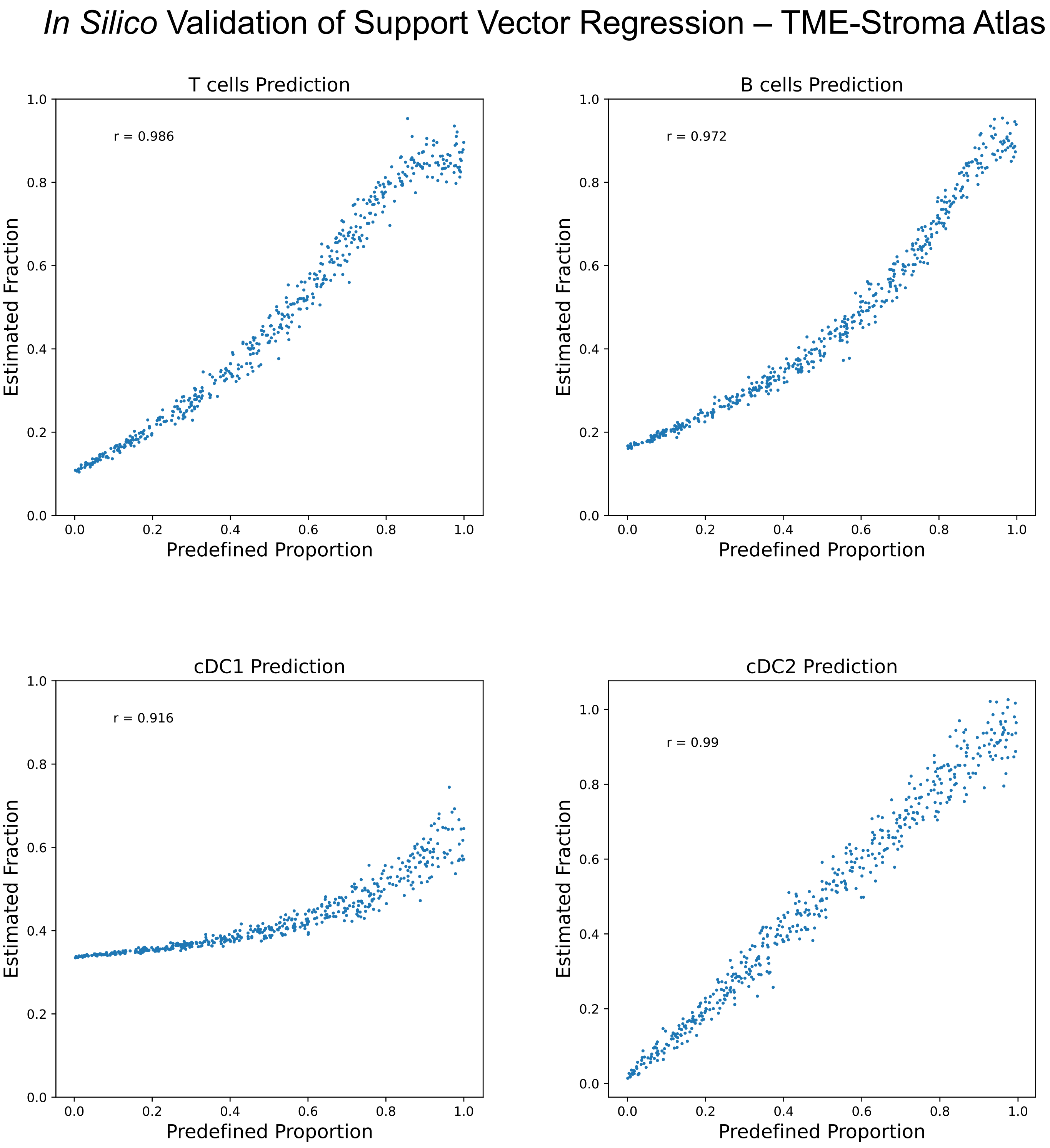
In Silico Validation of Support Vector Regression – TME-Stroma Atlas
T cells Prediction
r = 0.986
B cells Prediction
r = 0.972
cDC1 Prediction
r = 0.916
cDC2 Prediction
r = 0.99
Estimated Fraction
Predefined Proportion
0.0
0.2
0.4
0.6
0.8
1.0

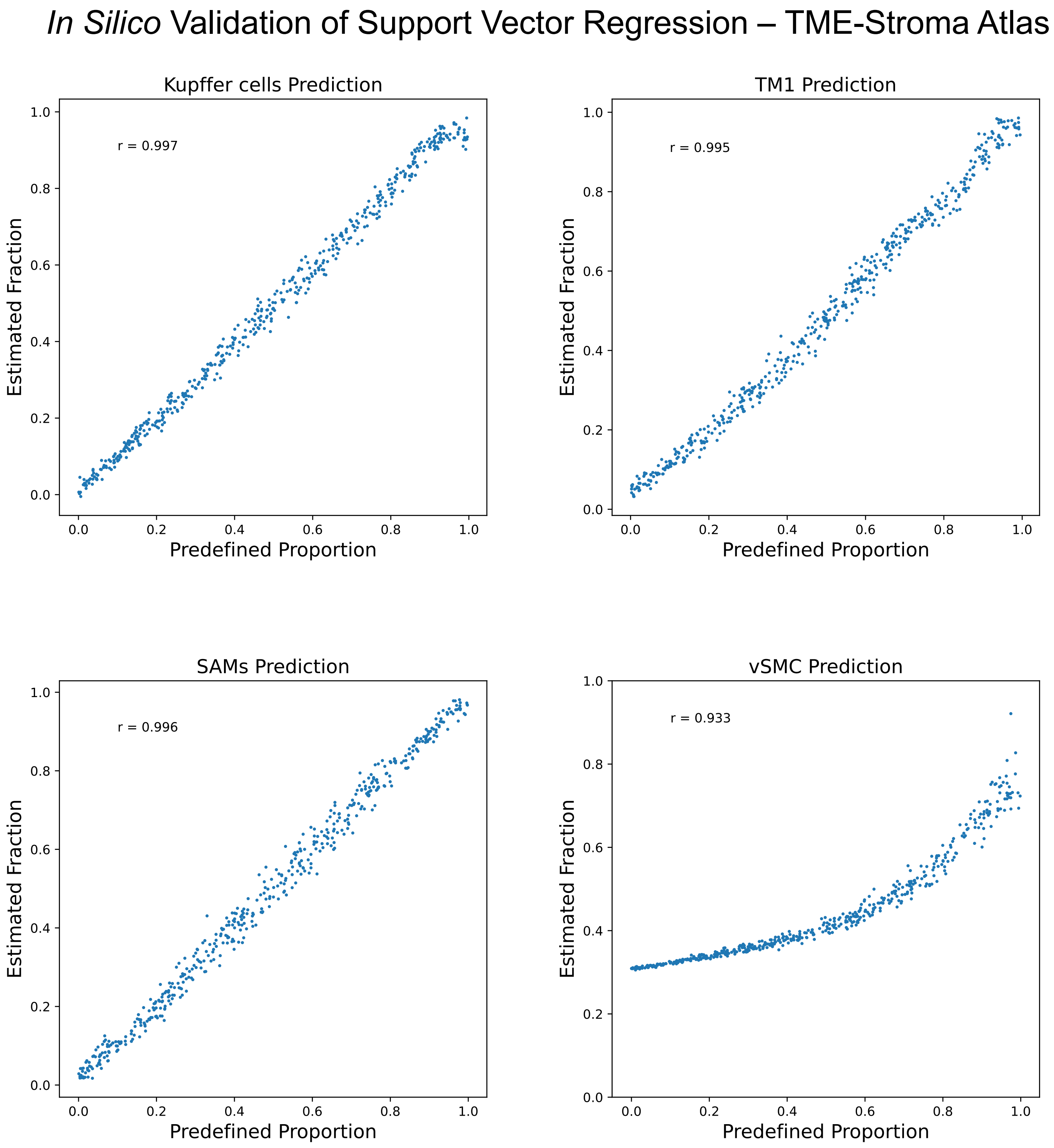
In Silico Validation of Support Vector Regression – TME-Stroma Atlas
Kupffer cells Prediction
r = 0.997
TM1 Prediction
r = 0.995
SAMs Prediction
r = 0.996
vSMC Prediction
r = 0.933
Estimated Fraction
Predefined Proportion

In Silico Validation of Support Vector Regression – TME-Immune Atlas

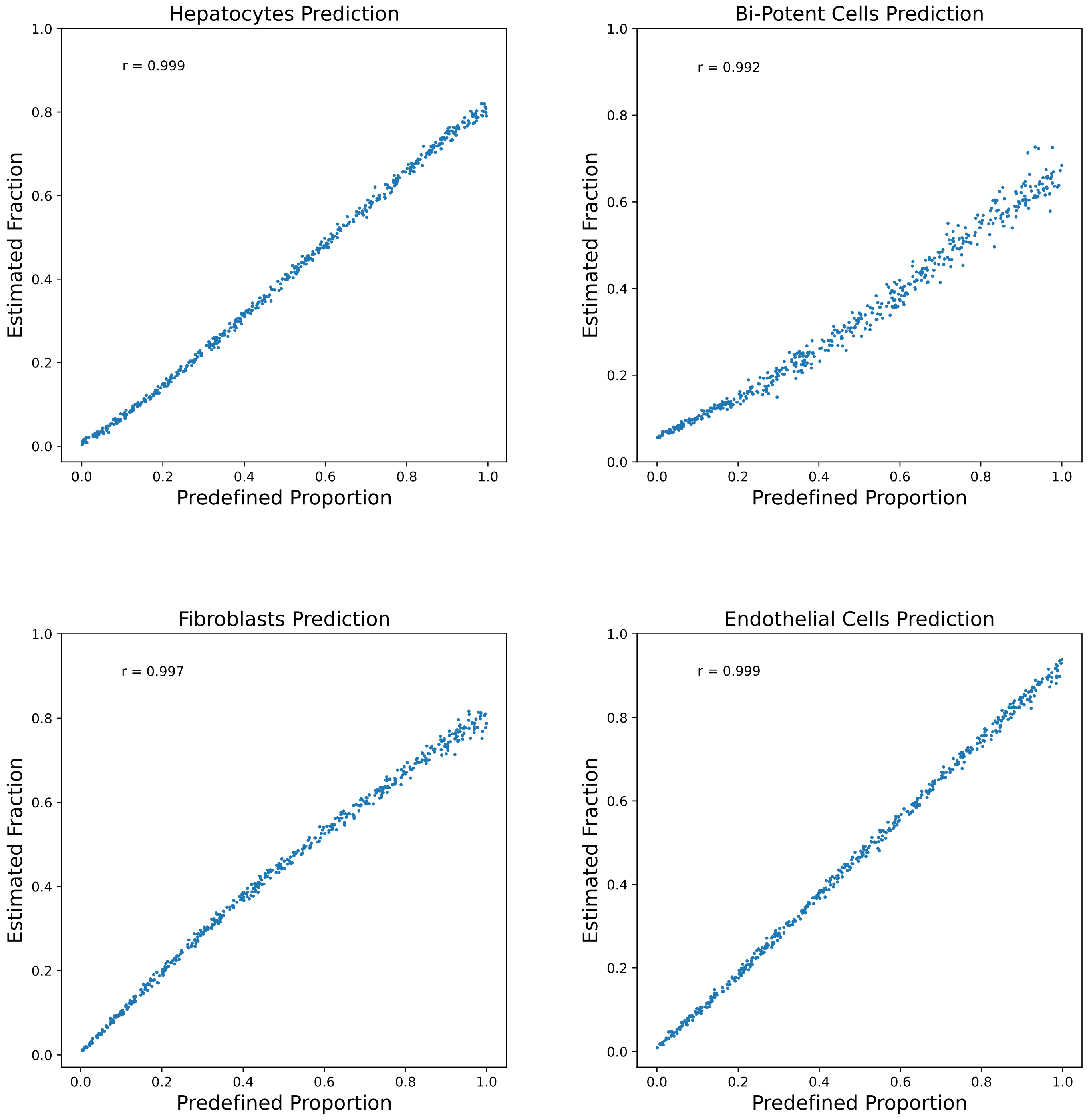

In Silico Validation of Support Vector Regression – TME-Immune Atlas

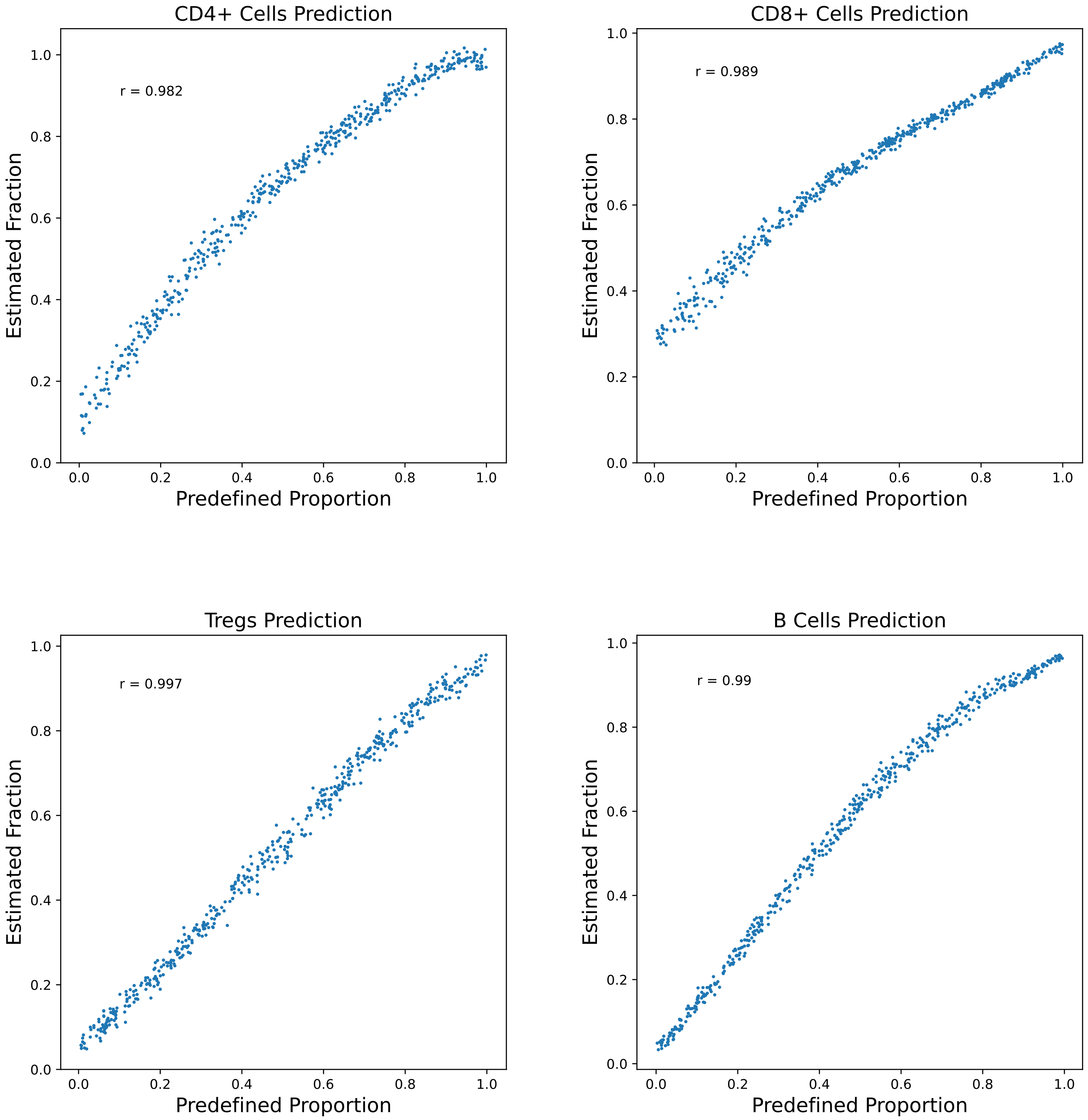

In Silico Validation of Support Vector Regression – TME-Immune Atlas

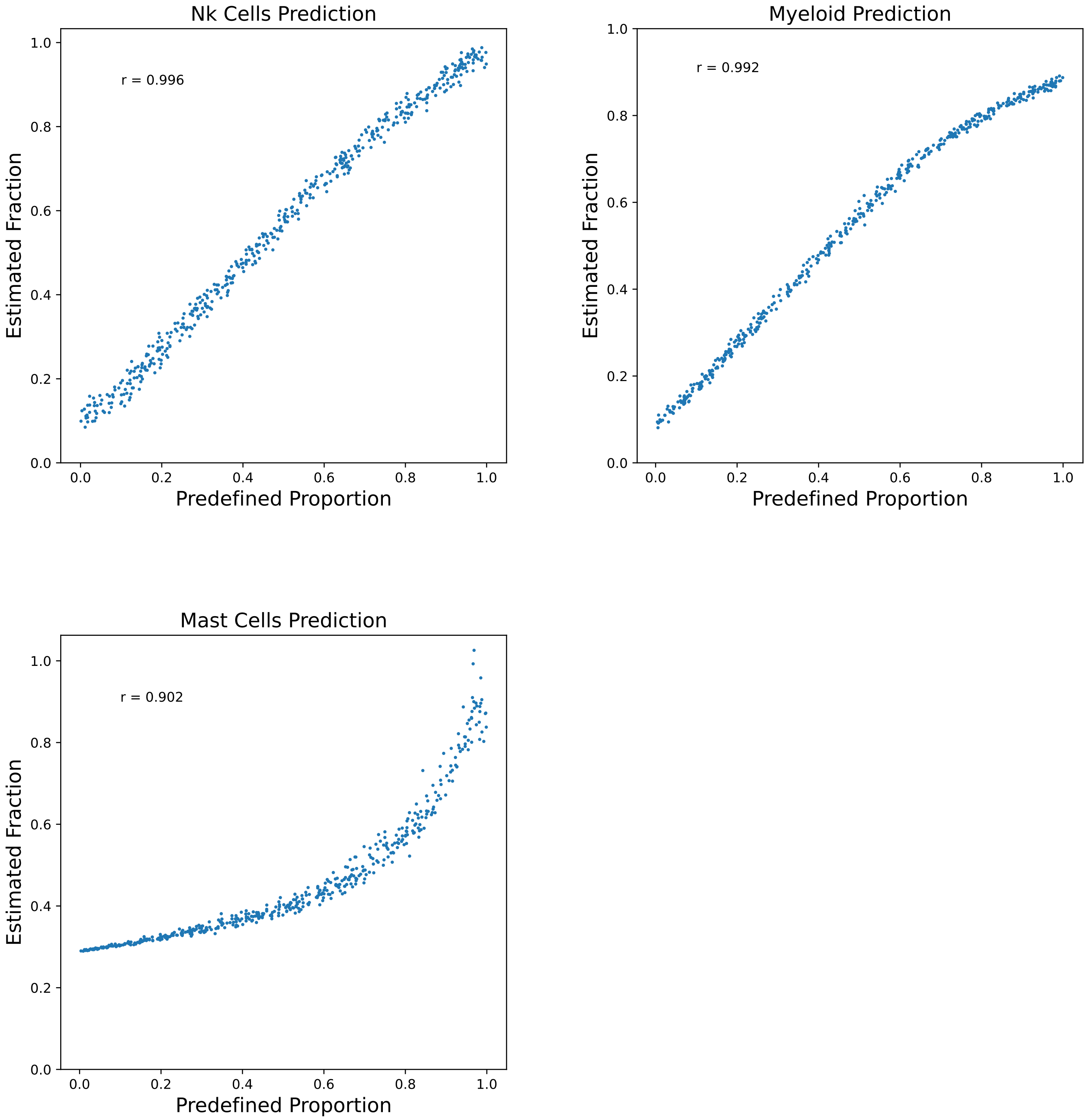

In Silico Validation of Support Vector Regression – Other Cells

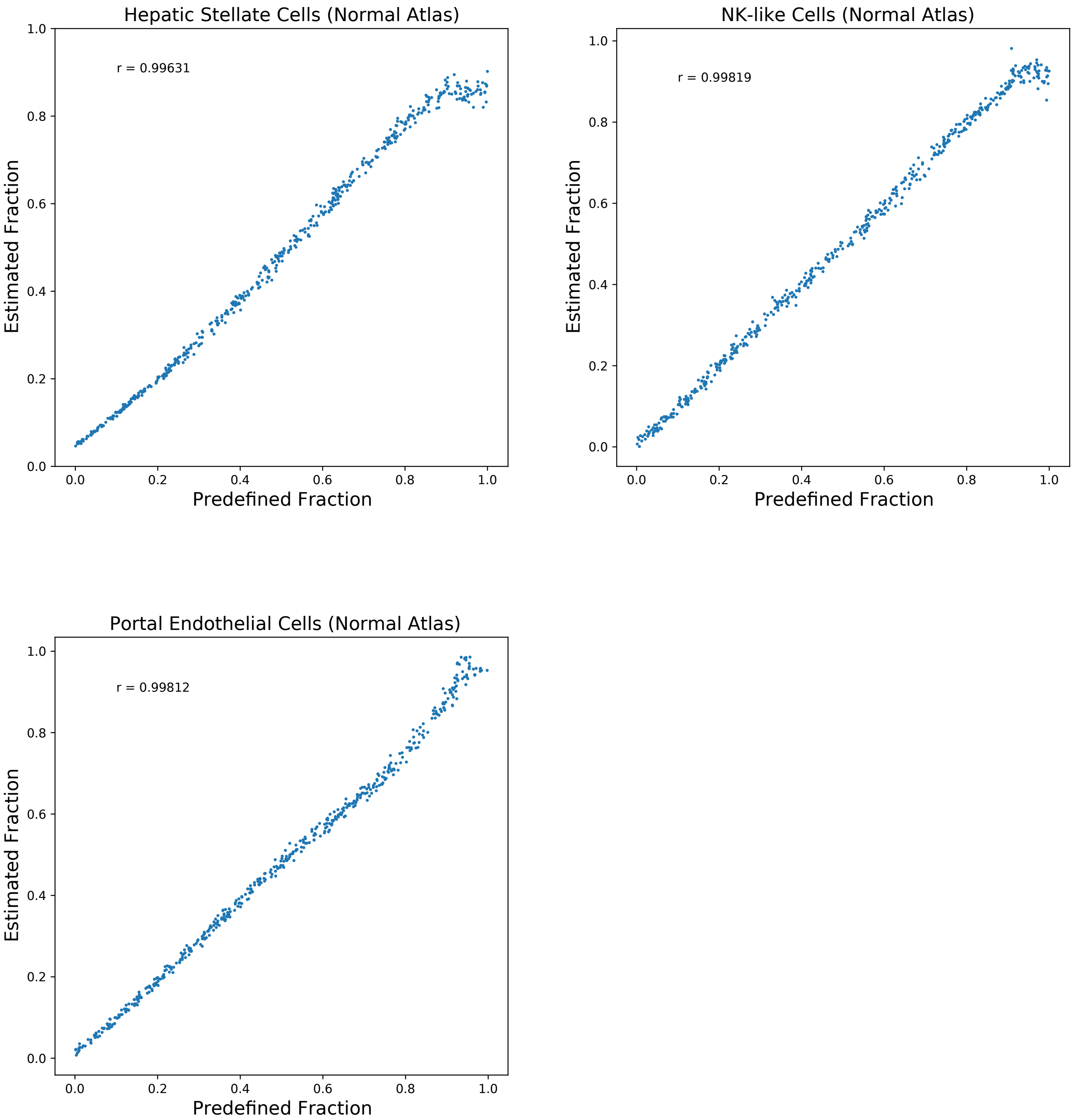

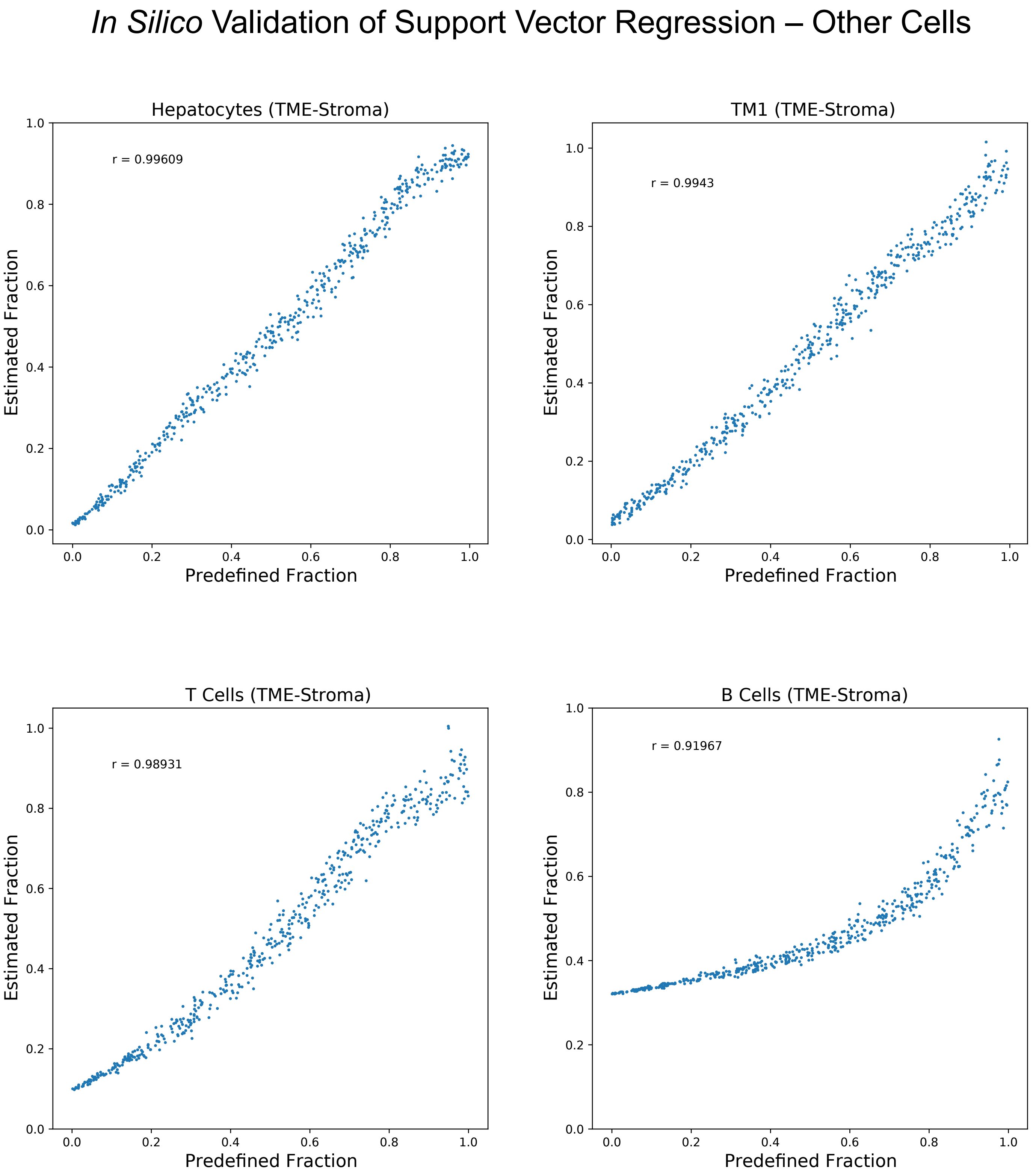
In Silico Validation of Support Vector Regression – Other Cells
Hepatocytes (TME-Stroma)
r = 0.99609
TM1 (TME-Stroma)
r = 0.9943
T Cells (TME-Stroma)
r = 0.98931
B Cells (TME-Stroma)
r = 0.91967
Estimated Fraction
Predefined Fraction
0.0
0.2
0.4
0.6
0.8
1.0

In Silico Validation of Support Vector Regression – Other Cells

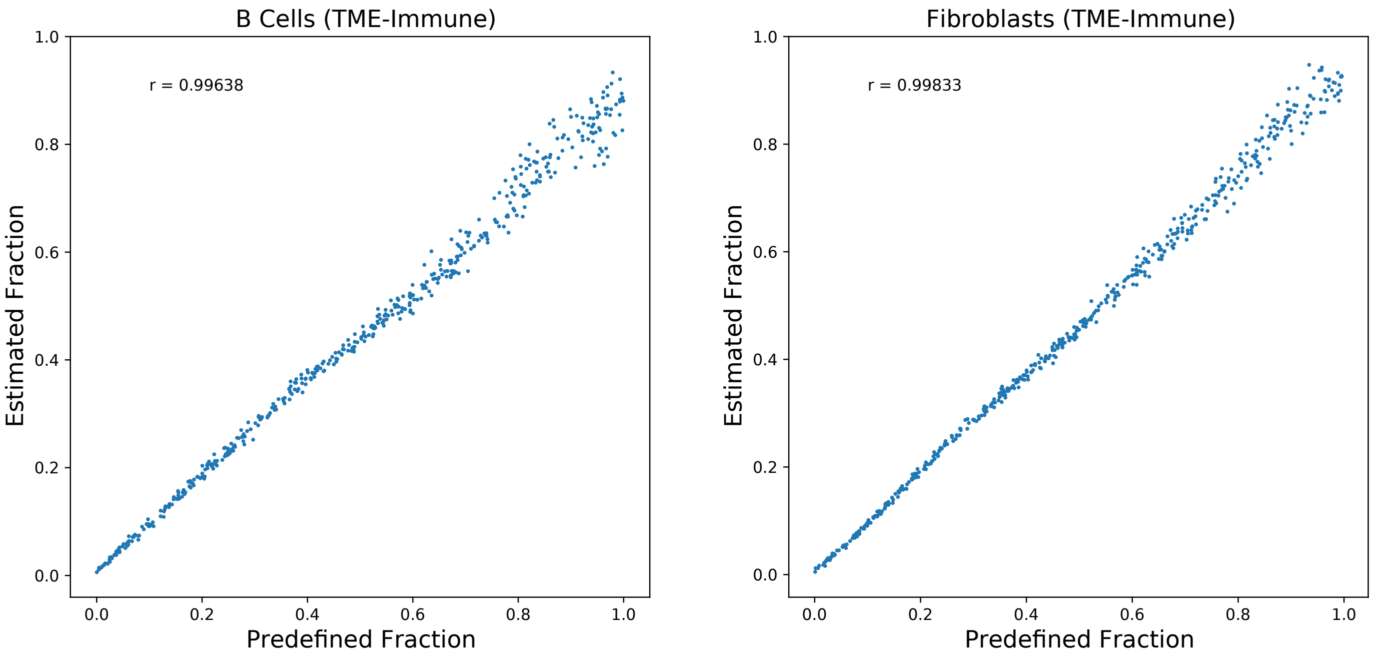

Supplement: Supplementary file 1 [file cancers-15-00153-s001.zip › cancers-2059594-supplementary/Supplements/S4_In_Silico_Validation_of_Cibersortx_and_Support_Vector_Regression.pdf]
