# Supplementary material for: Deciphering Tumour Microenvironment of Liver Cancer through Deconvolution of Bulk RNA-Seq Data with Single-Cell Atlas
Source: Cancers (Basel). 2022 Dec 27;15(1):153. doi: 10.3390/cancers15010153 (PMC9818189; doi:10.3390/cancers15010153)
Supplement: Supplementary file 1 [file cancers-15-00153-s001.zip › cancers-2059594-supplementary/Supplements/S5_Survival_Impacts_of_Cell_Fractions_Estimated_by_Cibersortx_TCGA-LIHC.pdf]

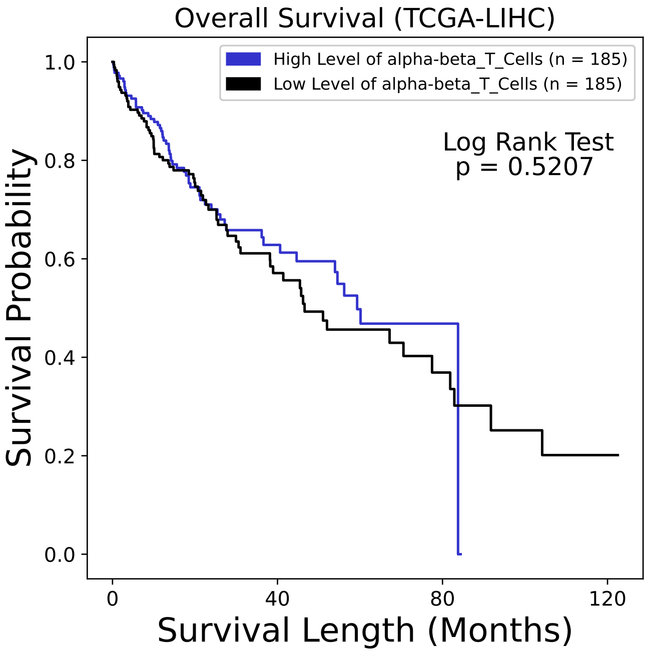
Overall Survival (TCGA-LIHC)
High Level of alpha-beta_T_Cells (n = 185)
Low Level of alpha-beta_T_Cells (n = 185)
Log Rank Test
p = 0.5207
Survival Probability
Survival Length (Months)
1.0
0.8
0.6
0.4
0.2
0.0
0
40
80
120

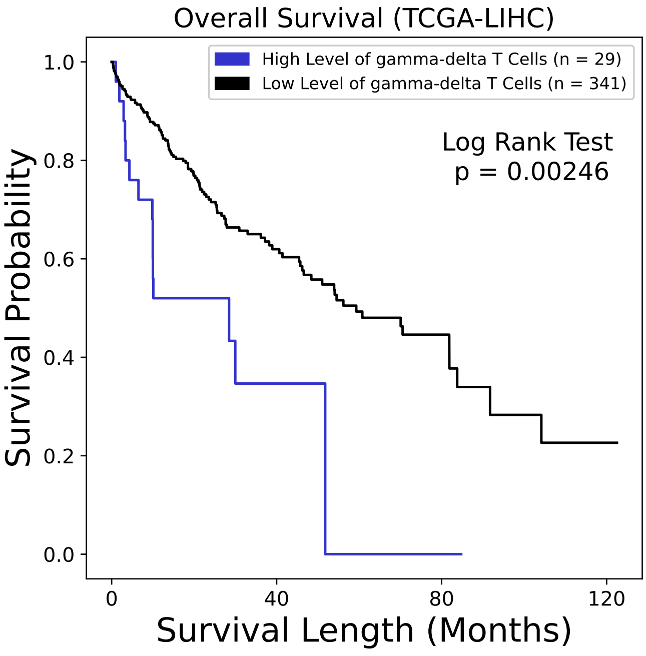
Overall Survival (TCGA-LIHC)
High Level of gamma-delta T Cells (n = 29)
Low Level of gamma-delta T Cells (n = 341)
Log Rank Test
p = 0.00246
Survival Probability
Survival Length (Months)
1.0
0.8
0.6
0.4
0.2
0.0
0
40
80
120

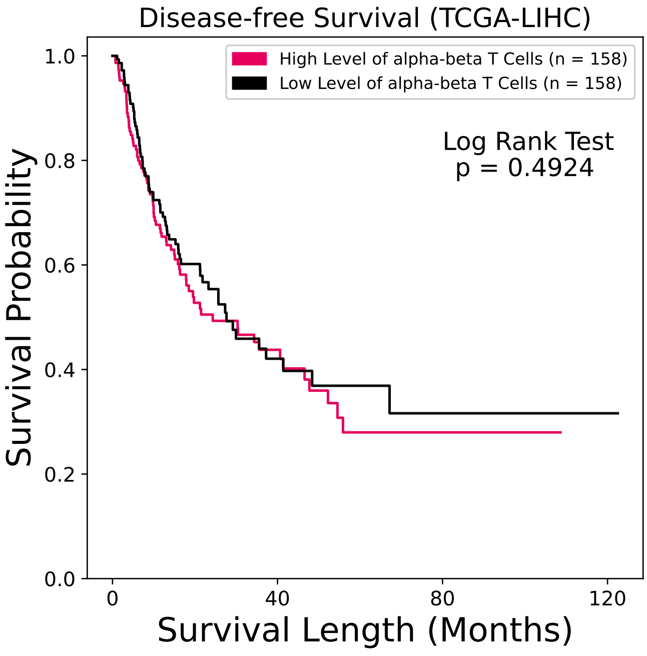
Disease-free Survival (TCGA-LIHC)
High Level of alpha-beta T Cells (n = 158)
Low Level of alpha-beta T Cells (n = 158)
Log Rank Test
p = 0.4924
Survival Probability
Survival Length (Months)
1.0
0.8
0.6
0.4
0.2
0.0
0
40
80
120

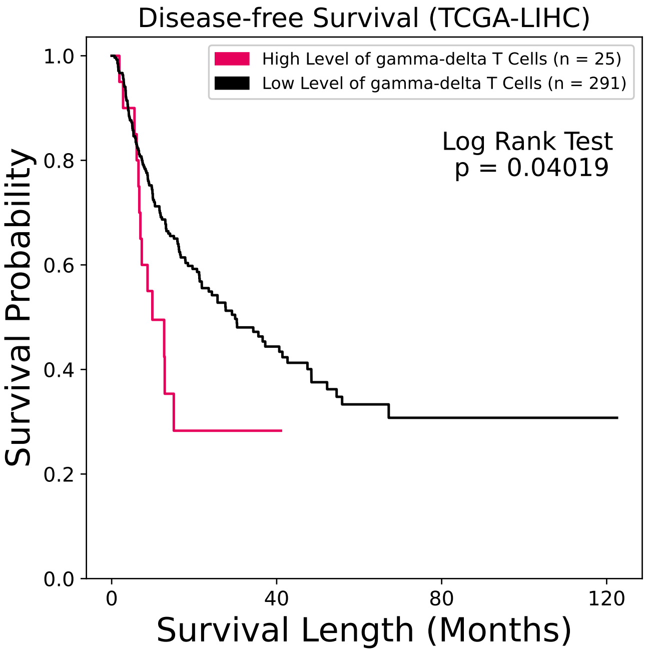
Disease-free Survival (TCGA-LIHC)
High Level of gamma-delta T Cells (n = 25)
Low Level of gamma-delta T Cells (n = 291)
Log Rank Test
p = 0.04019
Survival Probability
Survival Length (Months)
1.0
0.8
0.6
0.4
0.2
0.0
0
40
80
120

B3

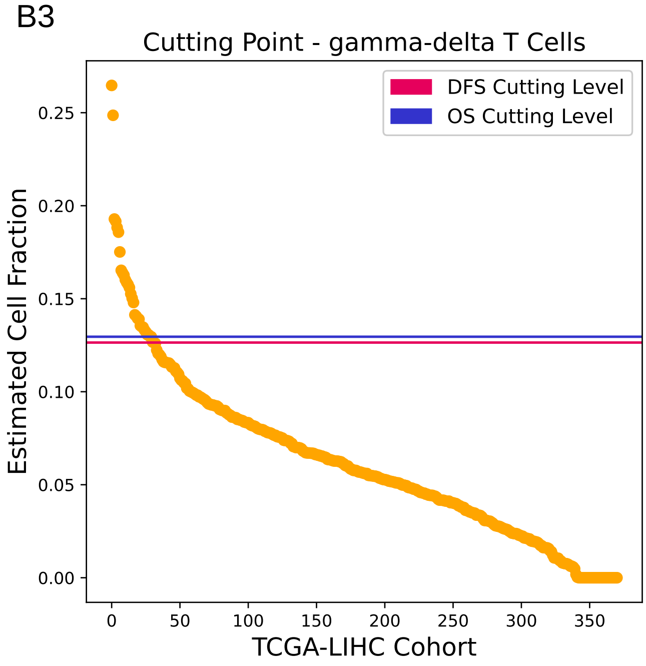
Cutting Point - gamma-delta T Cells
DFS Cutting Level
OS Cutting Level
Estimated Cell Fraction
TCGA-LIHC Cohort
0.25
0.20
0.15
0.10
0.05
0.00
0
50
100
150
200
250
300
350

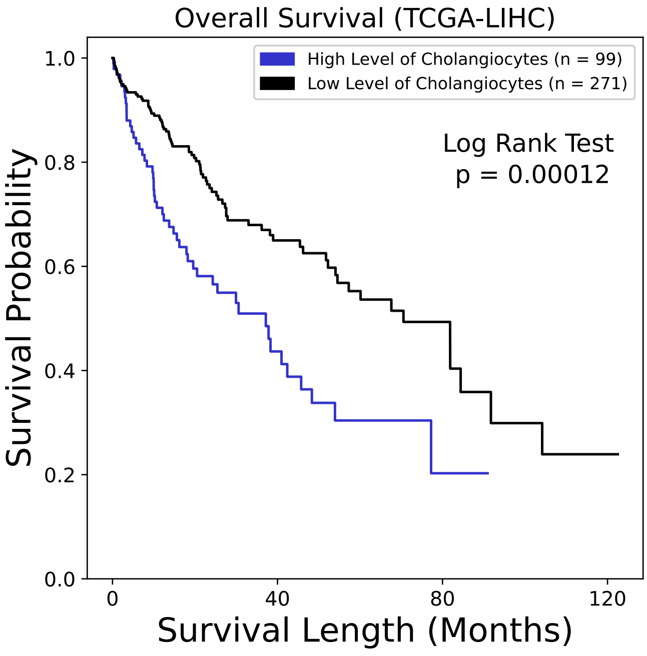
Overall Survival (TCGA-LIHC)
High Level of Cholangiocytes (n = 99)
Low Level of Cholangiocytes (n = 271)
Log Rank Test
p = 0.00012
Survival Probability
Survival Length (Months)

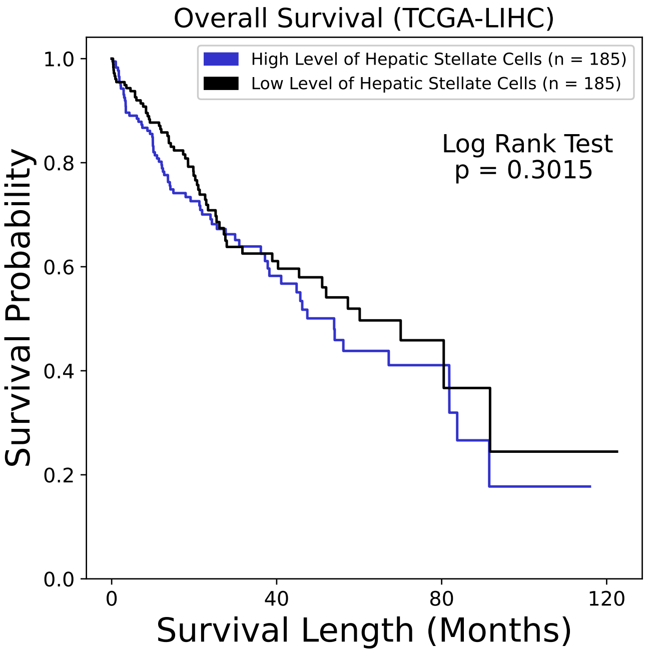
Overall Survival (TCGA-LIHC)
High Level of Hepatic Stellate Cells (n = 185)
Low Level of Hepatic Stellate Cells (n = 185)
Log Rank Test
p = 0.3015
Survival Probability
Survival Length (Months)

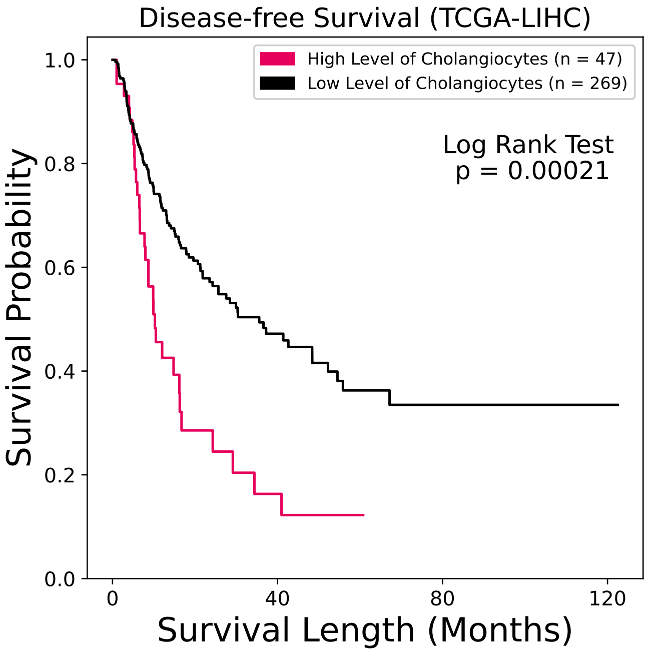
Disease-free Survival (TCGA-LIHC)
High Level of Cholangiocytes (n = 47)
Low Level of Cholangiocytes (n = 269)
Log Rank Test
p = 0.00021
Survival Probability
Survival Length (Months)

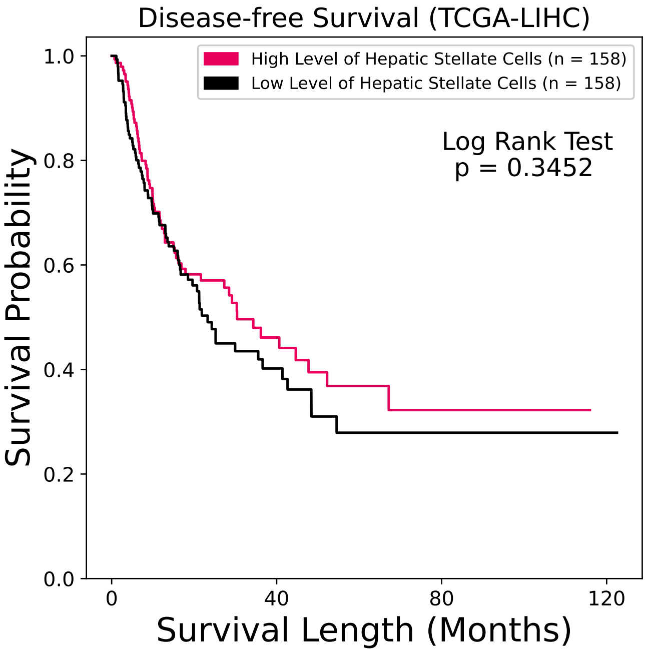
Disease-free Survival (TCGA-LIHC)
High Level of Hepatic Stellate Cells (n = 158)
Low Level of Hepatic Stellate Cells (n = 158)
Log Rank Test
p = 0.3452
Survival Probability
Survival Length (Months)

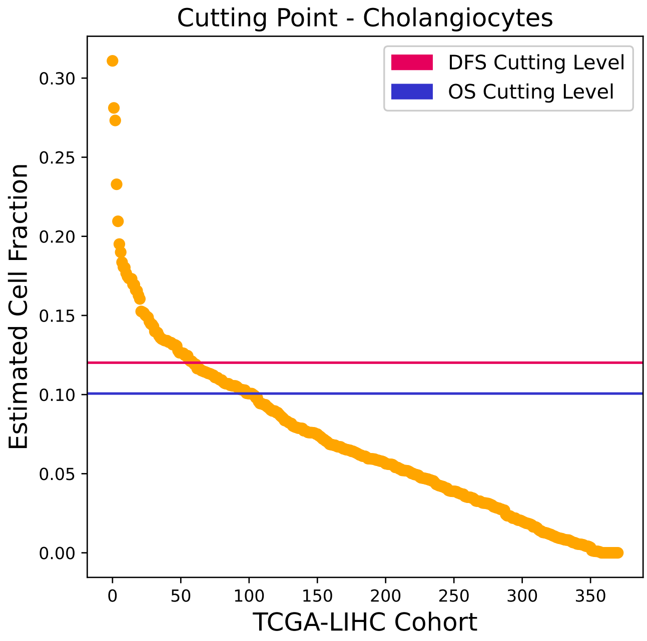
Cutting Point - Cholangiocytes
DFS Cutting Level
OS Cutting Level
Estimated Cell Fraction
TCGA-LIHC Cohort

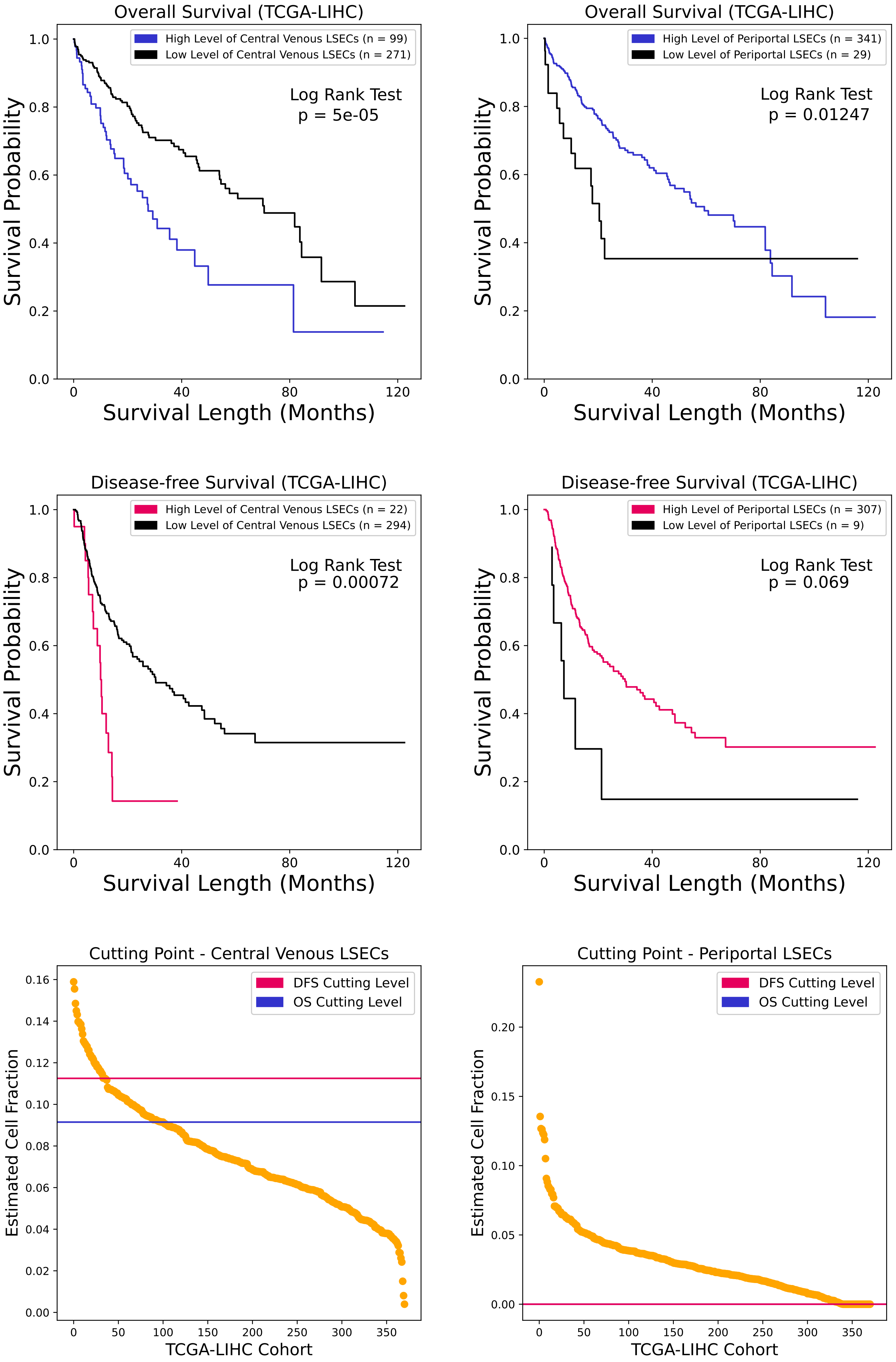

Overall Survival (TCGA-LIHC)
High Level of Central Venous LSECs (n = 99)
Low Level of Central Venous LSECs (n = 271)
Log Rank Test
p = 5e-05
Survival Probability
Survival Length (Months)
Overall Survival (TCGA-LIHC)
High Level of Periportal LSECs (n = 341)
Low Level of Periportal LSECs (n = 29)
Log Rank Test
p = 0.01247
Survival Probability
Survival Length (Months)
Disease-free Survival (TCGA-LIHC)
High Level of Central Venous LSECs (n = 22)
Low Level of Central Venous LSECs (n = 294)
Log Rank Test
p = 0.00072
Survival Probability
Survival Length (Months)
Disease-free Survival (TCGA-LIHC)
High Level of Periportal LSECs (n = 307)
Low Level of Periportal LSECs (n = 9)
Log Rank Test
p = 0.069
Survival Probability
Survival Length (Months)
Cutting Point - Central Venous LSECs
DFS Cutting Level
OS Cutting Level
Estimated Cell Fraction
TCGA-LIHC Cohort
Cutting Point - Periportal LSECs
DFS Cutting Level
OS Cutting Level
Estimated Cell Fraction
TCGA-LIHC Cohort

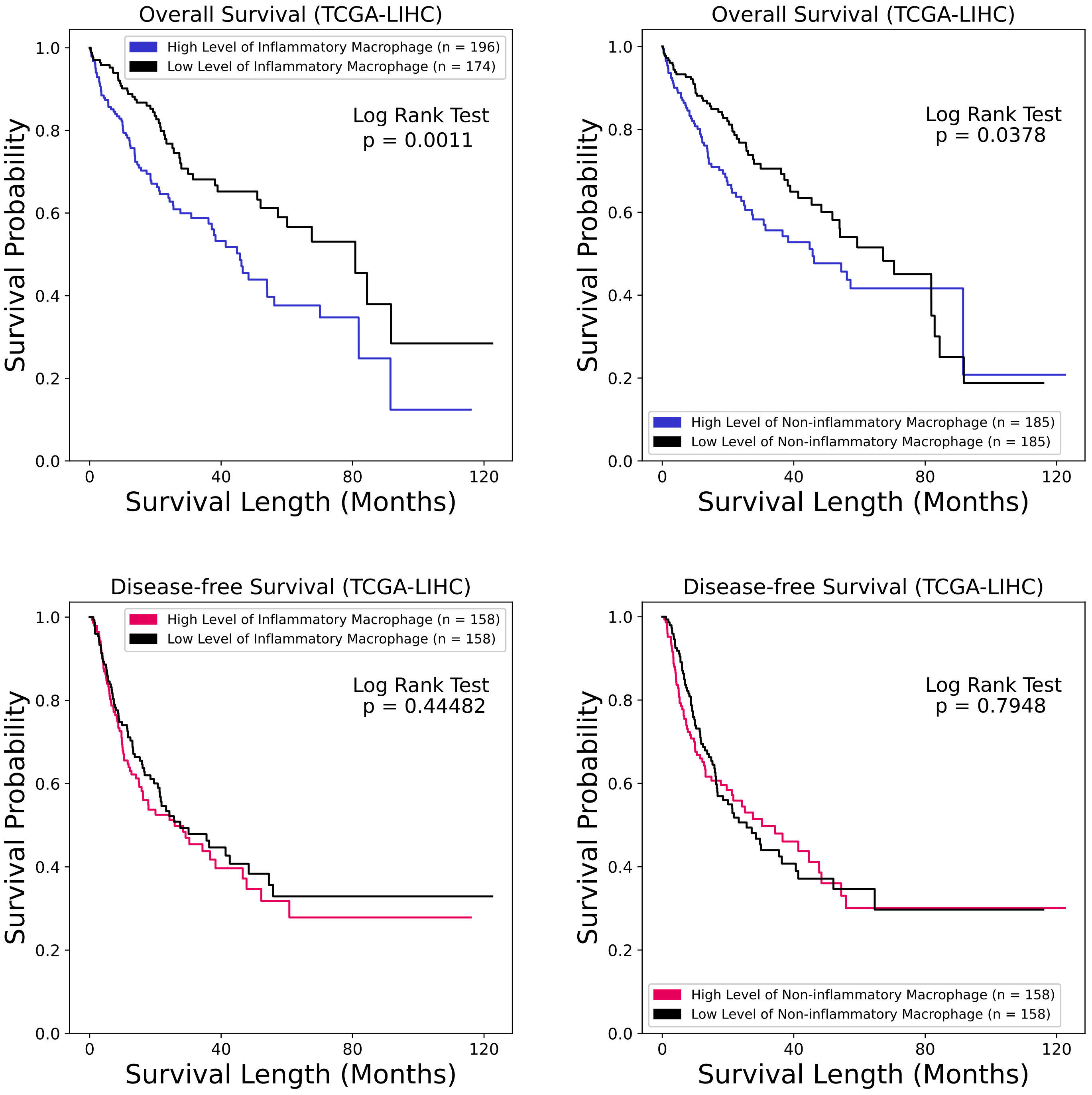
Overall Survival (TCGA-LIHC)
High Level of Inflammatory Macrophage (n = 196)
Low Level of Inflammatory Macrophage (n = 174)
Log Rank Test
p = 0.0011
Survival Probability
Survival Length (Months)
Overall Survival (TCGA-LIHC)
Log Rank Test
p = 0.0378
High Level of Non-inflammatory Macrophage (n = 185)
Low Level of Non-inflammatory Macrophage (n = 185)
Survival Probability
Survival Length (Months)
Disease-free Survival (TCGA-LIHC)
High Level of Inflammatory Macrophage (n = 158)
Low Level of Inflammatory Macrophage (n = 158)
Log Rank Test
p = 0.44482
Survival Probability
Survival Length (Months)
Disease-free Survival (TCGA-LIHC)
Log Rank Test
p = 0.7948
High Level of Non-inflammatory Macrophage (n = 158)
Low Level of Non-inflammatory Macrophage (n = 158)
Survival Probability
Survival Length (Months)

G3

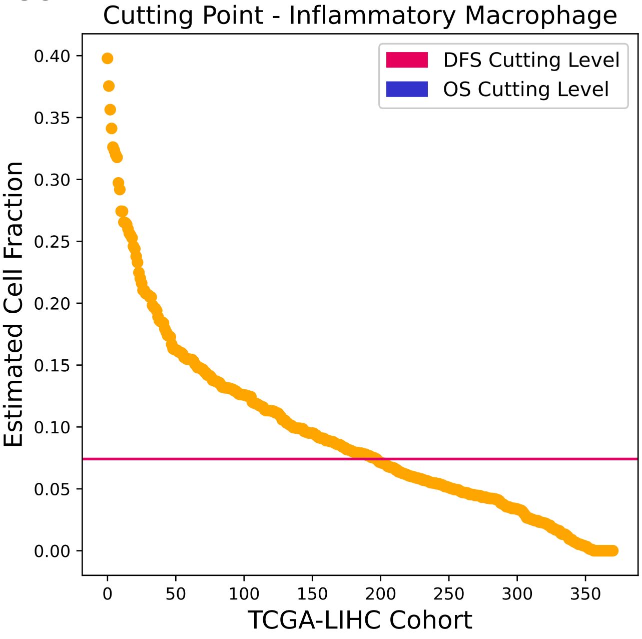
Cutting Point - Inflammatory Macrophage
DFS Cutting Level
OS Cutting Level
Estimated Cell Fraction
TCGA-LIHC Cohort

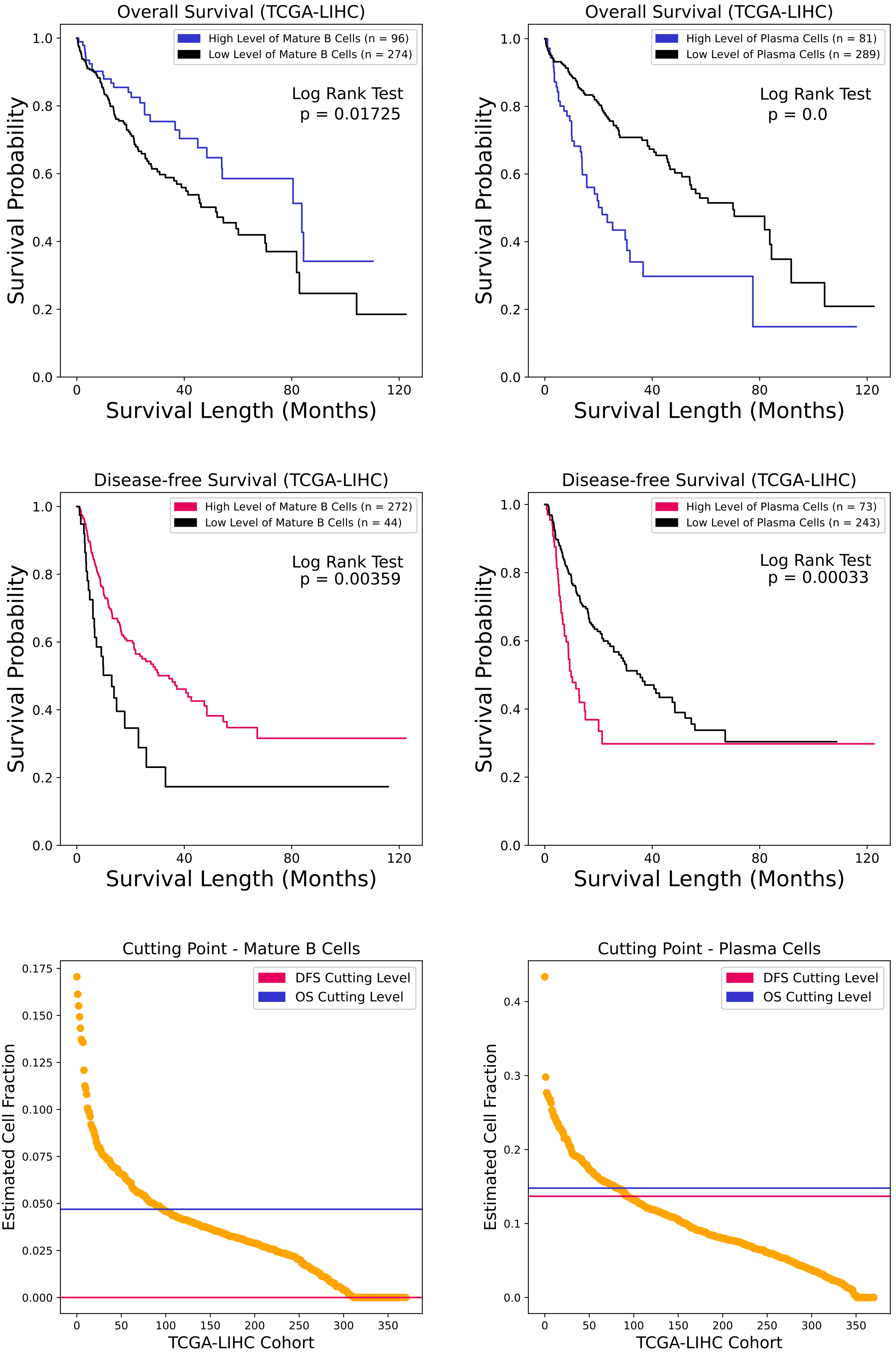
Overall Survival (TCGA-LIHC)
High Level of Mature B Cells (n = 96)
Low Level of Mature B Cells (n = 274)
Log Rank Test
p = 0.01725
Survival Probability
Survival Length (Months)
Overall Survival (TCGA-LIHC)
High Level of Plasma Cells (n = 81)
Low Level of Plasma Cells (n = 289)
Log Rank Test
p = 0.0
Survival Probability
Survival Length (Months)
Disease-free Survival (TCGA-LIHC)
High Level of Mature B Cells (n = 272)
Low Level of Mature B Cells (n = 44)
Log Rank Test
p = 0.00359
Survival Probability
Survival Length (Months)
Disease-free Survival (TCGA-LIHC)
High Level of Plasma Cells (n = 73)
Low Level of Plasma Cells (n = 243)
Log Rank Test
p = 0.00033
Survival Probability
Survival Length (Months)
Cutting Point - Mature B Cells
DFS Cutting Level
OS Cutting Level
Estimated Cell Fraction
TCGA-LIHC Cohort
Cutting Point - Plasma Cells
DFS Cutting Level
OS Cutting Level
Estimated Cell Fraction
TCGA-LIHC Cohort

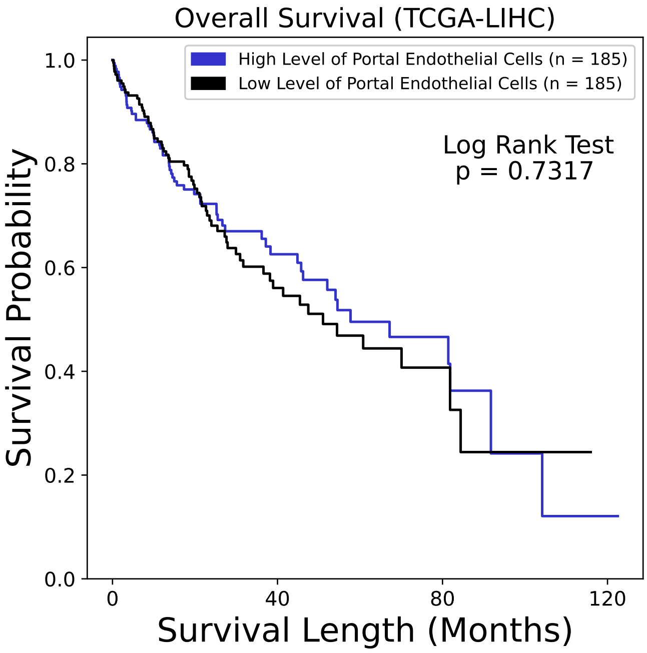
Overall Survival (TCGA-LIHC)
High Level of Portal Endothelial Cells (n = 185)
Low Level of Portal Endothelial Cells (n = 185)
Log Rank Test
p = 0.7317
Survival Probability
Survival Length (Months)

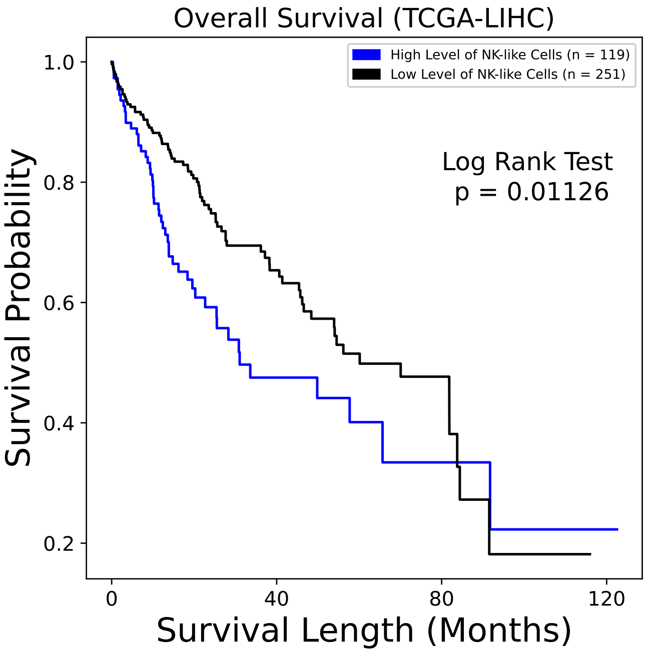
Overall Survival (TCGA-LIHC)
High Level of NK-like Cells (n = 119)
Low Level of NK-like Cells (n = 251)
Log Rank Test
p = 0.01126
Survival Probability
Survival Length (Months)

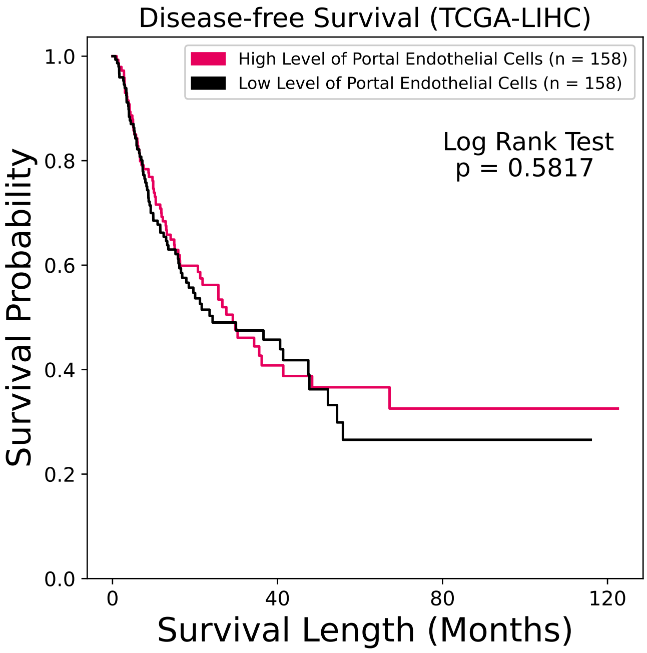
Disease-free Survival (TCGA-LIHC)
High Level of Portal Endothelial Cells (n = 158)
Low Level of Portal Endothelial Cells (n = 158)
Log Rank Test
p = 0.5817
Survival Probability
Survival Length (Months)

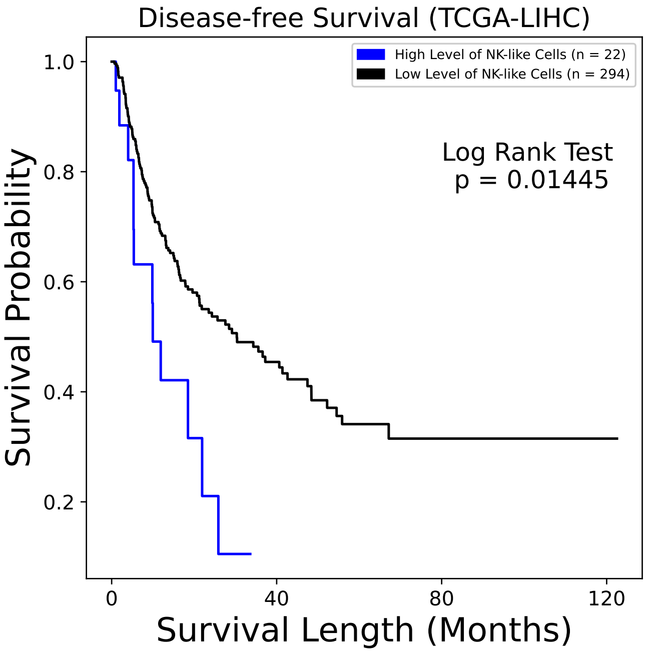
Disease-free Survival (TCGA-LIHC)
High Level of NK-like Cells (n = 22)
Low Level of NK-like Cells (n = 294)
Log Rank Test
p = 0.01445
Survival Probability
Survival Length (Months)

L3

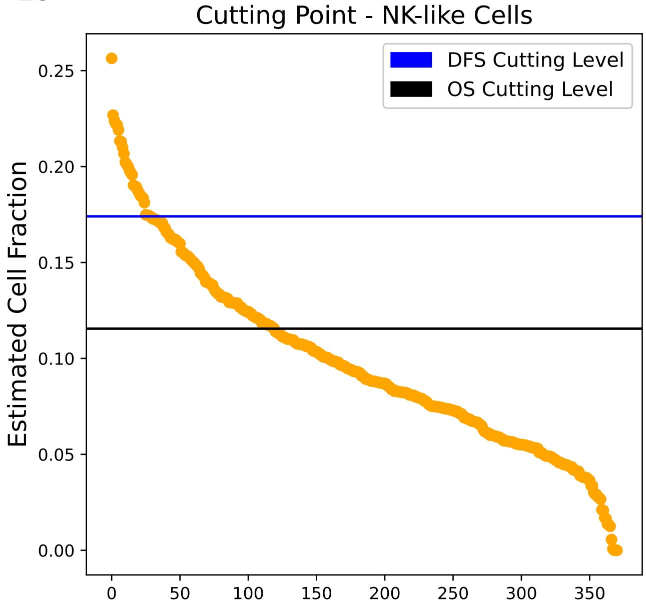
Cutting Point - NK-like Cells
DFS Cutting Level
OS Cutting Level
Estimated Cell Fraction

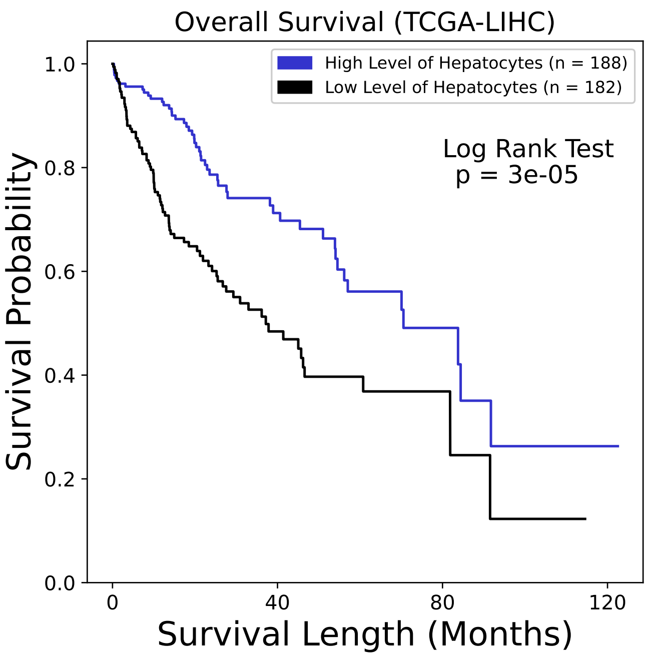
Overall Survival (TCGA-LIHC)
High Level of Hepatocytes (n = 188)
Low Level of Hepatocytes (n = 182)
Log Rank Test
p = 3e-05
Survival Probability
Survival Length (Months)
1.0
0.8
0.6
0.4
0.2
0.0
0
40
80
120

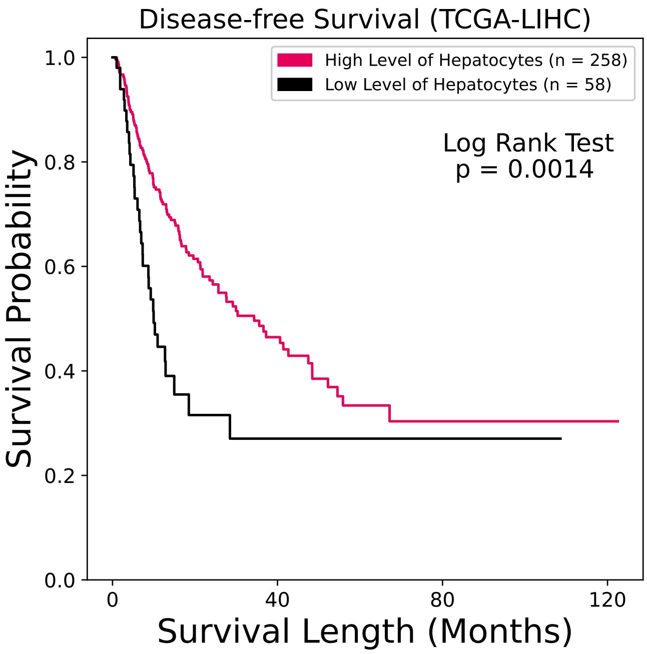
Disease-free Survival (TCGA-LIHC)
High Level of Hepatocytes (n = 258)
Low Level of Hepatocytes (n = 58)
Log Rank Test
p = 0.0014
Survival Probability
Survival Length (Months)
1.0
0.8
0.6
0.4
0.2
0.0
0
40
80
120

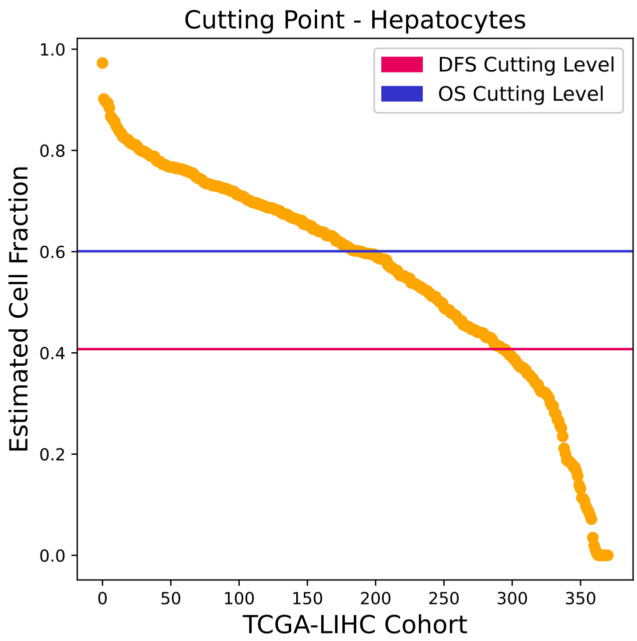
Cutting Point - Hepatocytes
DFS Cutting Level
OS Cutting Level
Estimated Cell Fraction
TCGA-LIHC Cohort
1.0
0.8
0.6
0.4
0.2
0.0
0
50
100
150
200
250
300
350

Figure S8

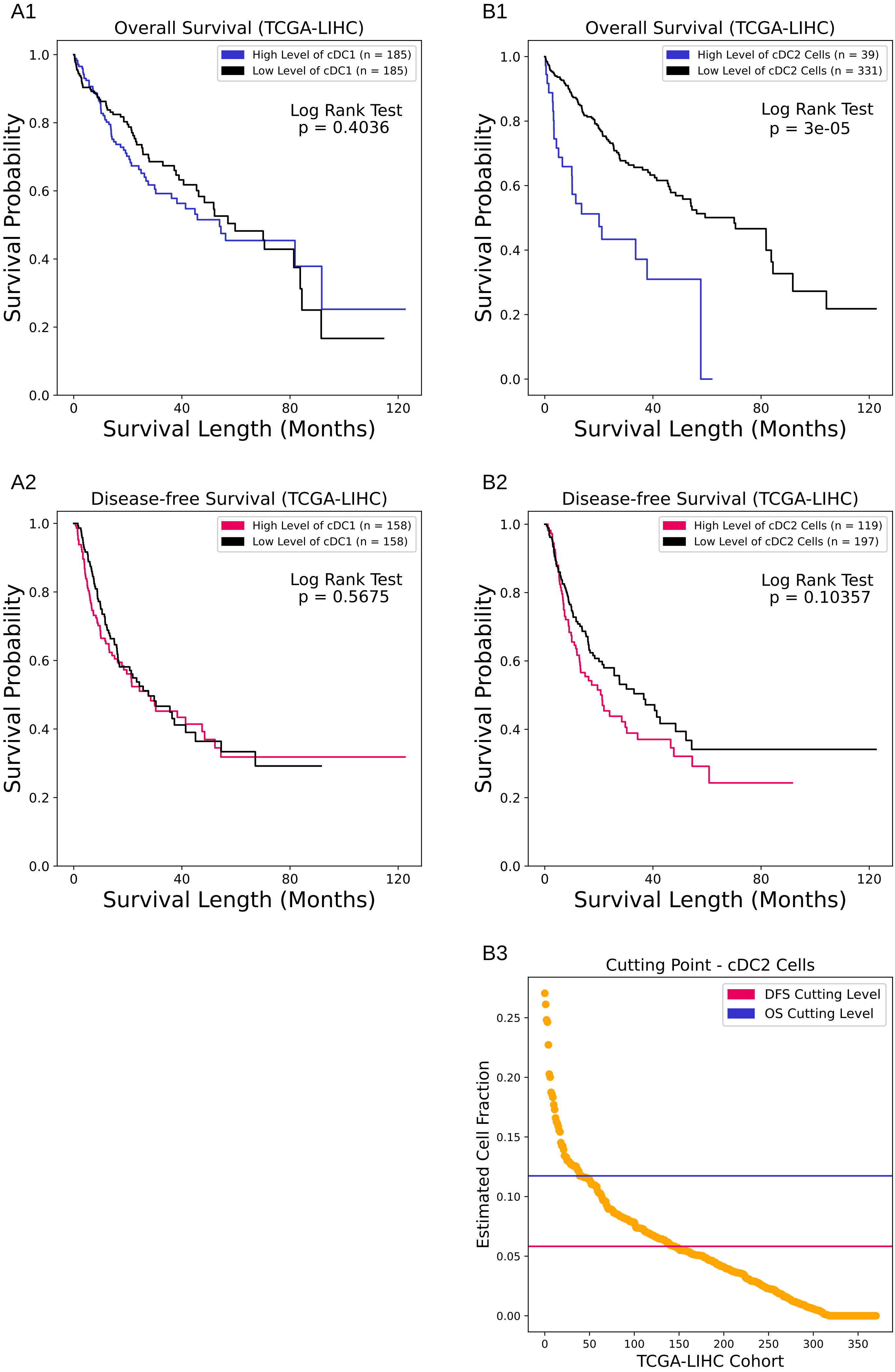

A1
Overall Survival (TCGA-LIHC)
High Level of cDC1 (n = 185)
Low Level of cDC1 (n = 185)
Log Rank Test
p = 0.4036
Survival Probability
Survival Length (Months)
B1
Overall Survival (TCGA-LIHC)
High Level of cDC2 Cells (n = 39)
Low Level of cDC2 Cells (n = 331)
Log Rank Test
p = 3e-05
Survival Probability
Survival Length (Months)
A2
Disease-free Survival (TCGA-LIHC)
High Level of cDC1 (n = 158)
Low Level of cDC1 (n = 158)
Log Rank Test
p = 0.5675
Survival Probability
Survival Length (Months)
B2
Disease-free Survival (TCGA-LIHC)
High Level of cDC2 Cells (n = 119)
Low Level of cDC2 Cells (n = 197)
Log Rank Test
p = 0.10357
Survival Probability
Survival Length (Months)
B3
Cutting Point - cDC2 Cells
DFS Cutting Level
OS Cutting Level
Estimated Cell Fraction
TCGA-LIHC Cohort

Figure S8

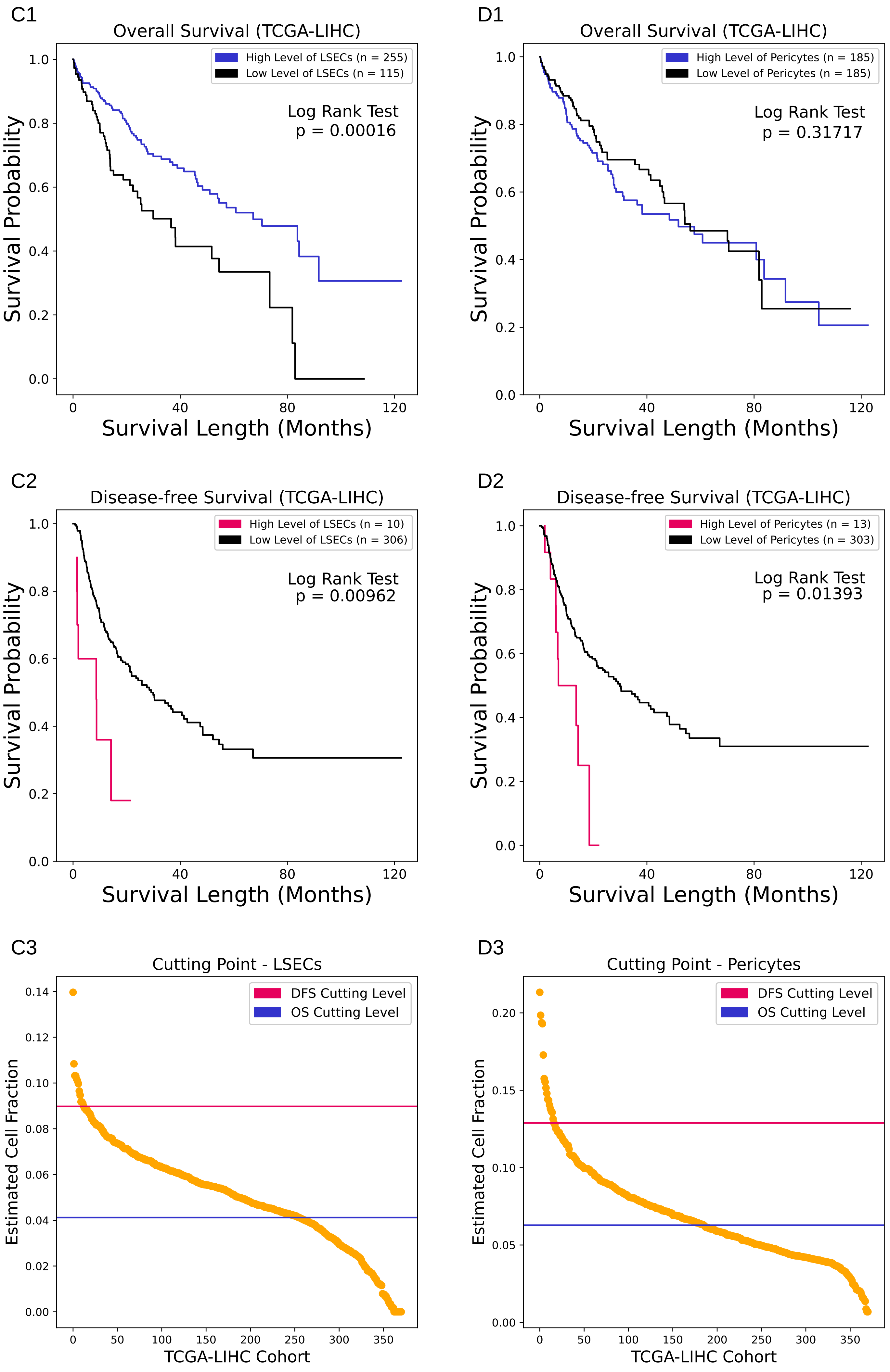
C1
Overall Survival (TCGA-LIHC)
High Level of LSECs (n = 255)
Low Level of LSECs (n = 115)
Log Rank Test
p = 0.00016
Survival Probability
Survival Length (Months)
D1
Overall Survival (TCGA-LIHC)
High Level of Pericytes (n = 185)
Low Level of Pericytes (n = 185)
Log Rank Test
p = 0.31717
Survival Probability
Survival Length (Months)
C2
Disease-free Survival (TCGA-LIHC)
High Level of LSECs (n = 10)
Low Level of LSECs (n = 306)
Log Rank Test
p = 0.00962
Survival Probability
Survival Length (Months)
D2
Disease-free Survival (TCGA-LIHC)
High Level of Pericytes (n = 13)
Low Level of Pericytes (n = 303)
Log Rank Test
p = 0.01393
Survival Probability
Survival Length (Months)
C3
Cutting Point - LSECs
DFS Cutting Level
OS Cutting Level
Estimated Cell Fraction
TCGA-LIHC Cohort
D3
Cutting Point - Pericytes
DFS Cutting Level
OS Cutting Level
Estimated Cell Fraction
TCGA-LIHC Cohort

Figure S8

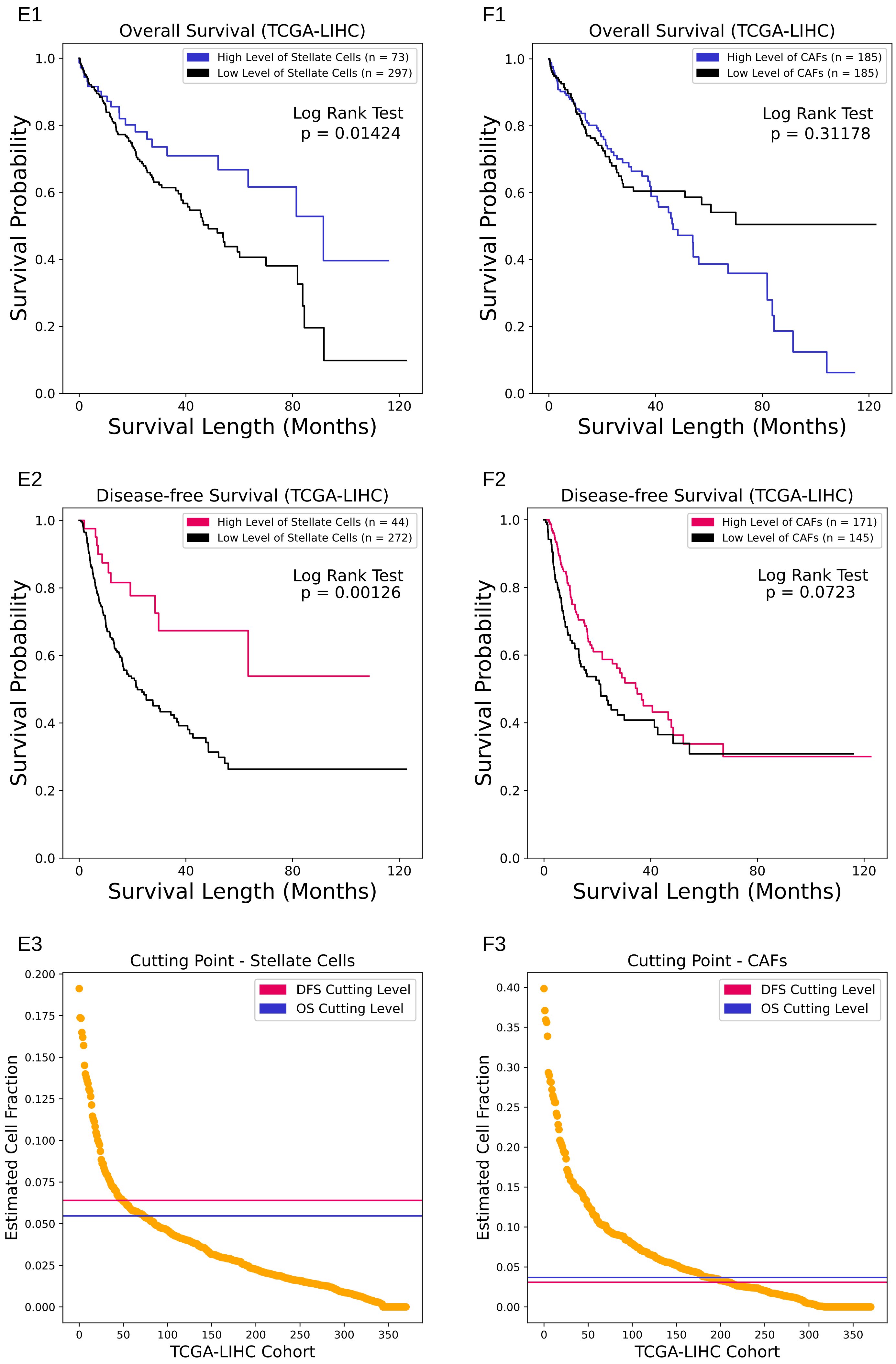
E1
Overall Survival (TCGA-LIHC)
High Level of Stellate Cells (n = 73)
Low Level of Stellate Cells (n = 297)
Log Rank Test
p = 0.01424
Survival Probability
Survival Length (Months)
F1
Overall Survival (TCGA-LIHC)
High Level of CAFs (n = 185)
Low Level of CAFs (n = 185)
Log Rank Test
p = 0.31178
Survival Probability
Survival Length (Months)
E2
Disease-free Survival (TCGA-LIHC)
High Level of Stellate Cells (n = 44)
Low Level of Stellate Cells (n = 272)
Log Rank Test
p = 0.00126
Survival Probability
Survival Length (Months)
F2
Disease-free Survival (TCGA-LIHC)
High Level of CAFs (n = 171)
Low Level of CAFs (n = 145)
Log Rank Test
p = 0.0723
Survival Probability
Survival Length (Months)
E3
Cutting Point - Stellate Cells
DFS Cutting Level
OS Cutting Level
Estimated Cell Fraction
TCGA-LIHC Cohort
F3
Cutting Point - CAFs
DFS Cutting Level
OS Cutting Level
Estimated Cell Fraction
TCGA-LIHC Cohort

Figure S8

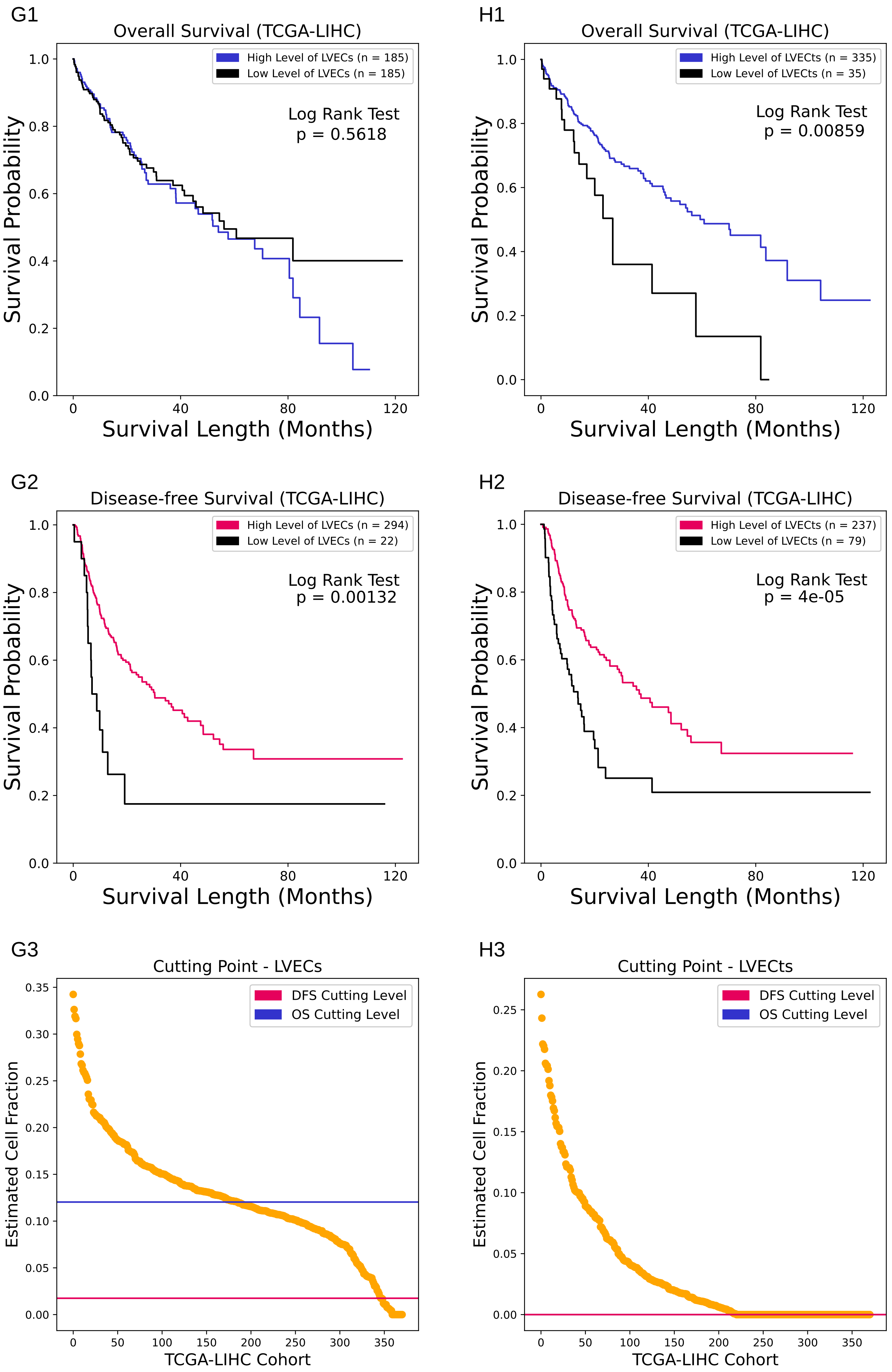

G1
Overall Survival (TCGA-LIHC)
High Level of LVECs (n = 185)
Low Level of LVECs (n = 185)
Log Rank Test
p = 0.5618
Survival Probability
Survival Length (Months)
H1
Overall Survival (TCGA-LIHC)
High Level of LVECts (n = 335)
Low Level of LVECts (n = 35)
Log Rank Test
p = 0.00859
Survival Probability
Survival Length (Months)
G2
Disease-free Survival (TCGA-LIHC)
High Level of LVECs (n = 294)
Low Level of LVECs (n = 22)
Log Rank Test
p = 0.00132
Survival Probability
Survival Length (Months)
H2
Disease-free Survival (TCGA-LIHC)
High Level of LVECts (n = 237)
Low Level of LVECts (n = 79)
Log Rank Test
p = 4e-05
Survival Probability
Survival Length (Months)
G3
Cutting Point - LVECs
DFS Cutting Level
OS Cutting Level
Estimated Cell Fraction
TCGA-LIHC Cohort
H3
Cutting Point - LVECts
DFS Cutting Level
OS Cutting Level
Estimated Cell Fraction
TCGA-LIHC Cohort

Figure S8

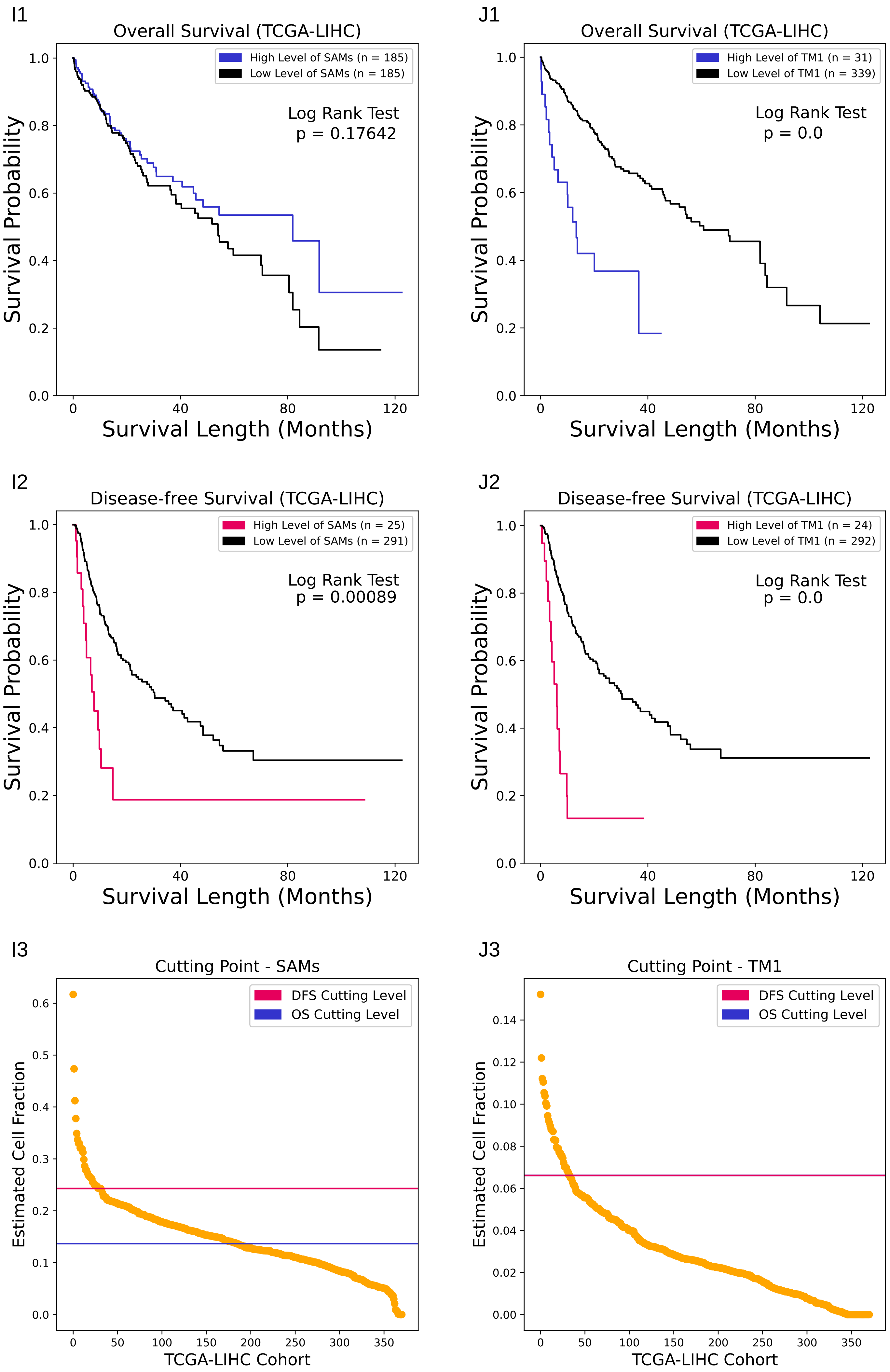
I1
Overall Survival (TCGA-LIHC)
High Level of SAMs (n = 185)
Low Level of SAMs (n = 185)
Log Rank Test
p = 0.17642
Survival Probability
Survival Length (Months)
J1
Overall Survival (TCGA-LIHC)
High Level of TM1 (n = 31)
Low Level of TM1 (n = 339)
Log Rank Test
p = 0.0
Survival Probability
Survival Length (Months)
I2
Disease-free Survival (TCGA-LIHC)
High Level of SAMs (n = 25)
Low Level of SAMs (n = 291)
Log Rank Test
p = 0.00089
Survival Probability
Survival Length (Months)
J2
Disease-free Survival (TCGA-LIHC)
High Level of TM1 (n = 24)
Low Level of TM1 (n = 292)
Log Rank Test
p = 0.0
Survival Probability
Survival Length (Months)
I3
Cutting Point - SAMs
DFS Cutting Level
OS Cutting Level
Estimated Cell Fraction
TCGA-LIHC Cohort
J3
Cutting Point - TM1
DFS Cutting Level
OS Cutting Level
Estimated Cell Fraction
TCGA-LIHC Cohort

Figure S8

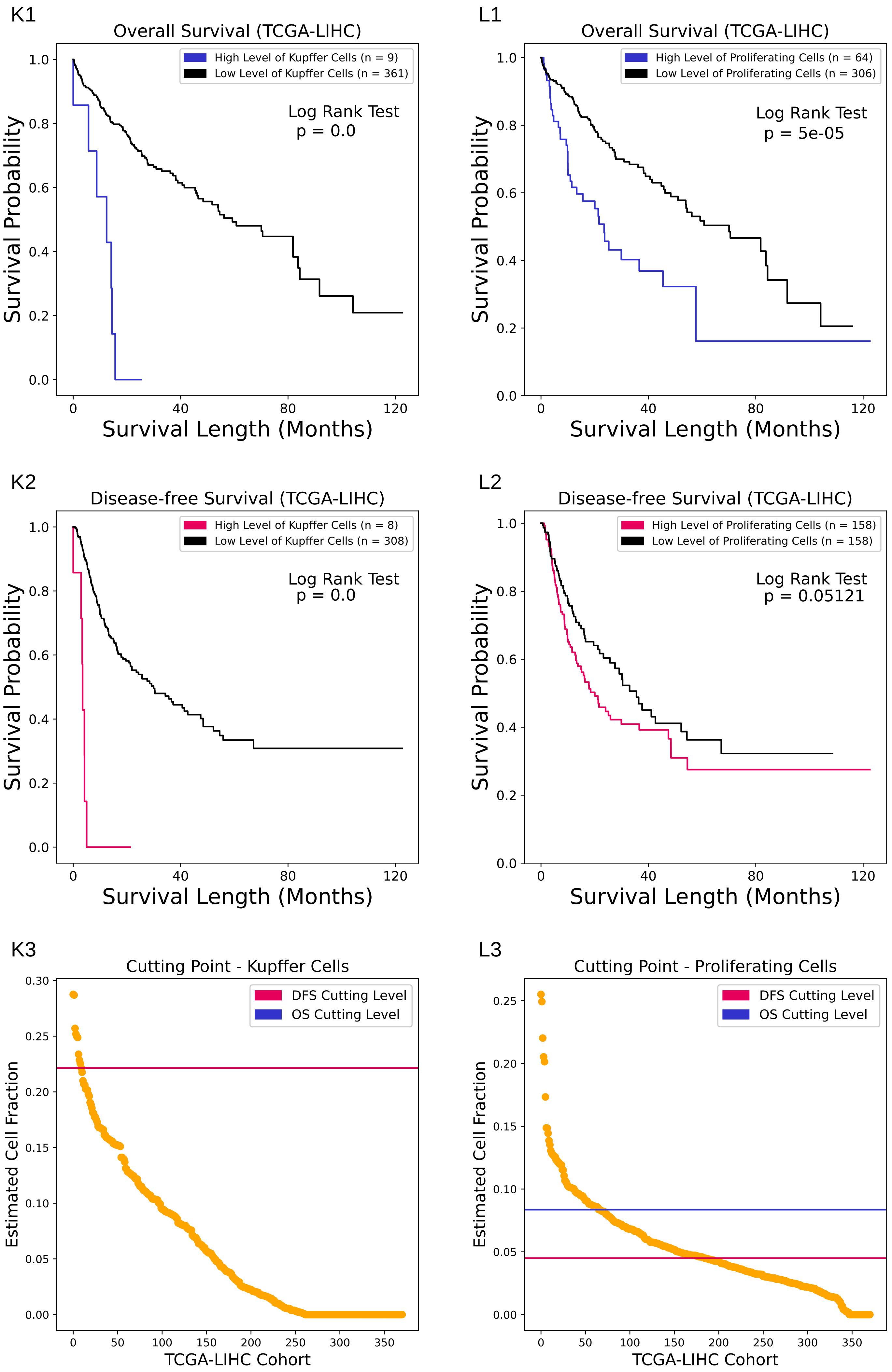

K1
Overall Survival (TCGA-LIHC)
High Level of Kupffer Cells (n = 9)
Low Level of Kupffer Cells (n = 361)
Log Rank Test
p = 0.0
Survival Probability
Survival Length (Months)
L1
Overall Survival (TCGA-LIHC)
High Level of Proliferating Cells (n = 64)
Low Level of Proliferating Cells (n = 306)
Log Rank Test
p = 5e-05
Survival Probability
Survival Length (Months)
K2
Disease-free Survival (TCGA-LIHC)
High Level of Kupffer Cells (n = 8)
Low Level of Kupffer Cells (n = 308)
Log Rank Test
p = 0.0
Survival Probability
Survival Length (Months)
L2
Disease-free Survival (TCGA-LIHC)
High Level of Proliferating Cells (n = 158)
Low Level of Proliferating Cells (n = 158)
Log Rank Test
p = 0.05121
Survival Probability
Survival Length (Months)
K3
Cutting Point - Kupffer Cells
DFS Cutting Level
OS Cutting Level
Estimated Cell Fraction
TCGA-LIHC Cohort
L3
Cutting Point - Proliferating Cells
DFS Cutting Level
OS Cutting Level
Estimated Cell Fraction
TCGA-LIHC Cohort

Figure S8

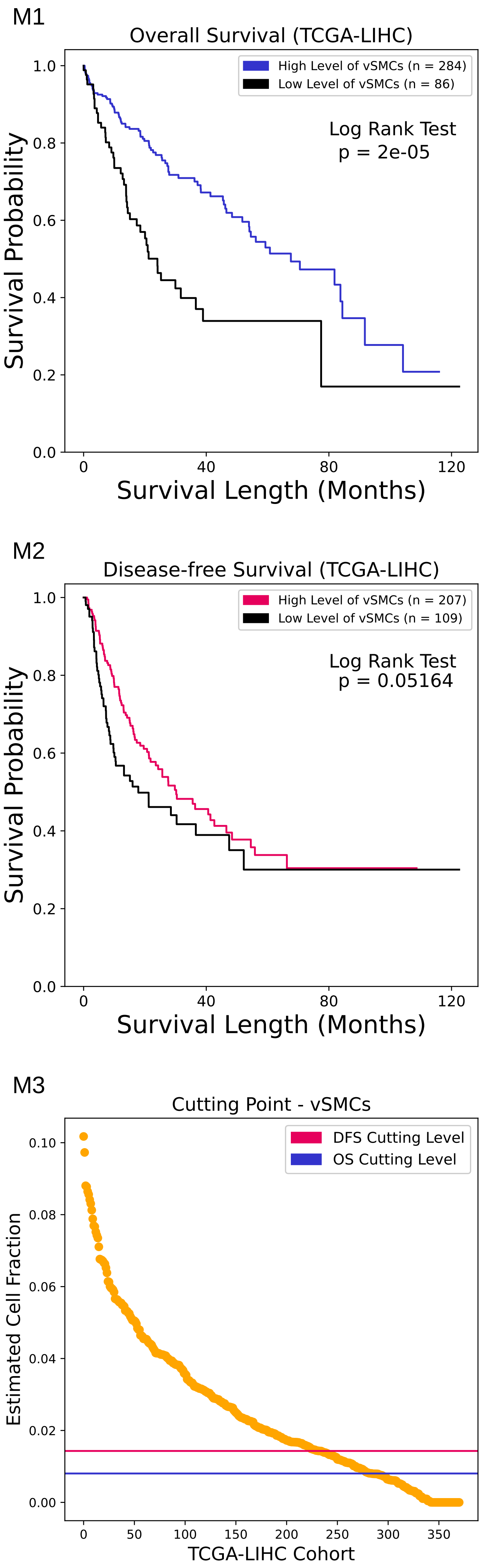
M1
Overall Survival (TCGA-LIHC)
High Level of vSMCs (n = 284)
Low Level of vSMCs (n = 86)
Log Rank Test
p = 2e-05
Survival Probability
1.0
0.8
0.6
0.4
0.2
0.0
0
40
80
120
Survival Length (Months)
M2
Disease-free Survival (TCGA-LIHC)
High Level of vSMCs (n = 207)
Low Level of vSMCs (n = 109)
Log Rank Test
p = 0.05164
Survival Probability
1.0
0.8
0.6
0.4
0.2
0.0
0
40
80
120
Survival Length (Months)
M3
Cutting Point - vSMCs
DFS Cutting Level
OS Cutting Level
Estimated Cell Fraction
0.10
0.08
0.06
0.04
0.02
0.00
0
50
100
150
200
250
300
350
TCGA-LIHC Cohort

Figure S9

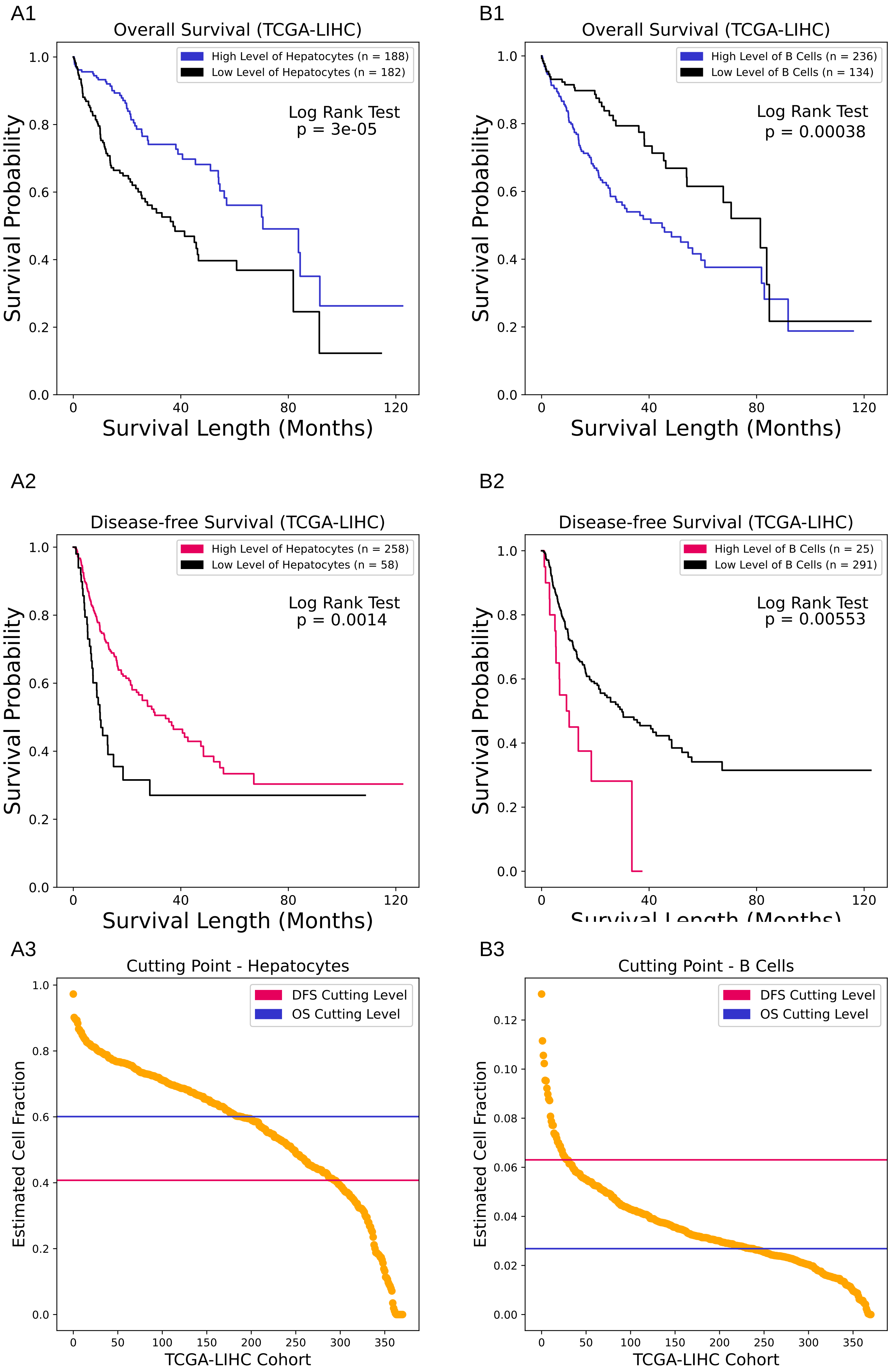
A1
Overall Survival (TCGA-LIHC)
High Level of Hepatocytes (n = 188)
Low Level of Hepatocytes (n = 182)
Log Rank Test
p = 3e-05
Survival Probability
Survival Length (Months)
B1
Overall Survival (TCGA-LIHC)
High Level of B Cells (n = 236)
Low Level of B Cells (n = 134)
Log Rank Test
p = 0.00038
Survival Probability
Survival Length (Months)
A2
Disease-free Survival (TCGA-LIHC)
High Level of Hepatocytes (n = 258)
Low Level of Hepatocytes (n = 58)
Log Rank Test
p = 0.0014
Survival Probability
Survival Length (Months)
B2
Disease-free Survival (TCGA-LIHC)
High Level of B Cells (n = 25)
Low Level of B Cells (n = 291)
Log Rank Test
p = 0.00553
Survival Probability
A3
Cutting Point - Hepatocytes
DFS Cutting Level
OS Cutting Level
Estimated Cell Fraction
TCGA-LIHC Cohort
B3
Cutting Point - B Cells
DFS Cutting Level
OS Cutting Level
Estimated Cell Fraction
TCGA-LIHC Cohort

Figure S9

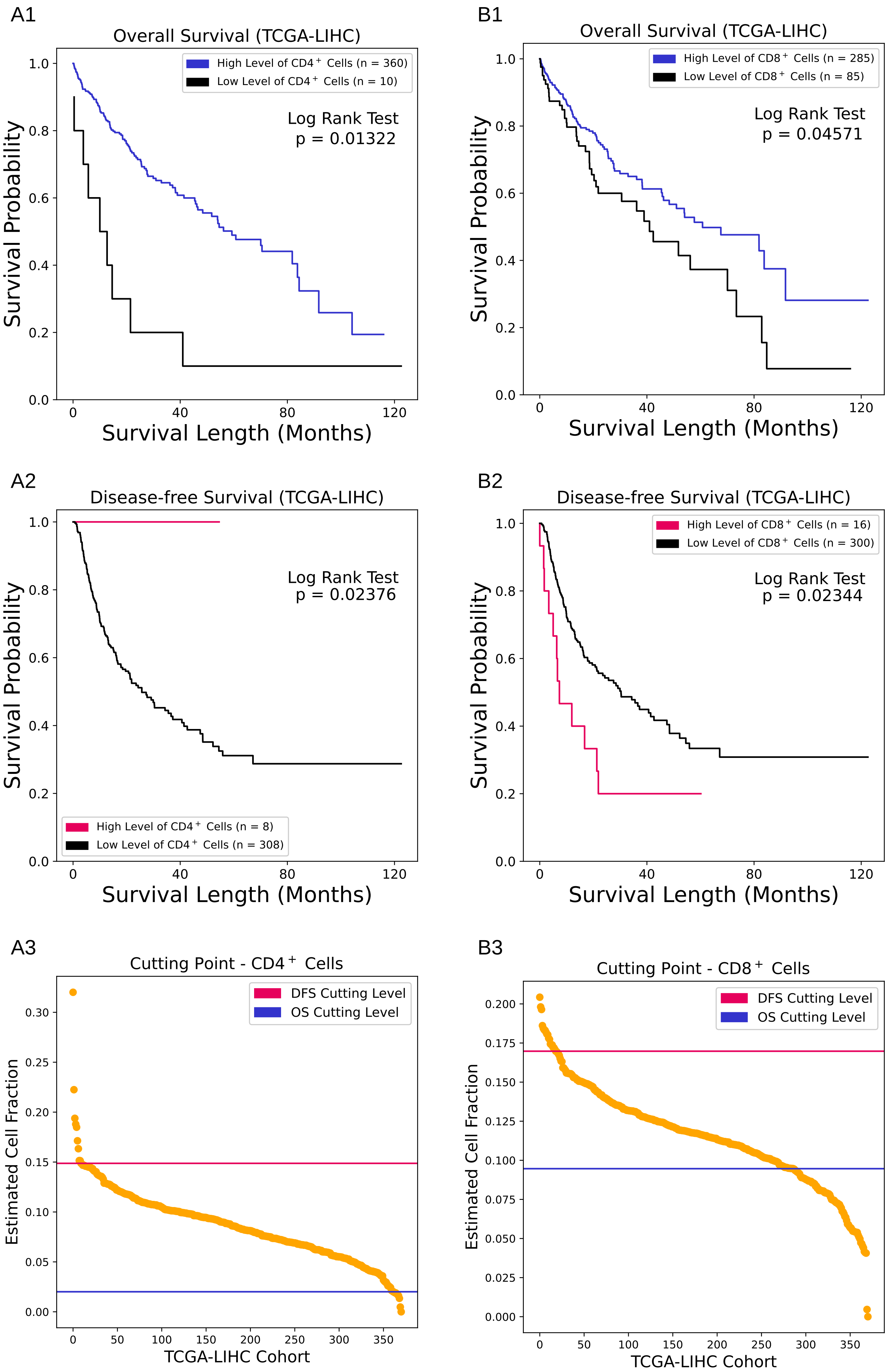
A1
Overall Survival (TCGA-LIHC)
High Level of CD4+ Cells (n = 360)
Low Level of CD4+ Cells (n = 10)
Log Rank Test
p = 0.01322
Survival Probability
Survival Length (Months)
B1
Overall Survival (TCGA-LIHC)
High Level of CD8+ Cells (n = 285)
Low Level of CD8+ Cells (n = 85)
Log Rank Test
p = 0.04571
Survival Probability
Survival Length (Months)
A2
Disease-free Survival (TCGA-LIHC)
Log Rank Test
p = 0.02376
High Level of CD4+ Cells (n = 8)
Low Level of CD4+ Cells (n = 308)
Survival Probability
Survival Length (Months)
B2
Disease-free Survival (TCGA-LIHC)
High Level of CD8+ Cells (n = 16)
Low Level of CD8+ Cells (n = 300)
Log Rank Test
p = 0.02344
Survival Probability
Survival Length (Months)
A3
Cutting Point - CD4+ Cells
DFS Cutting Level
OS Cutting Level
Estimated Cell Fraction
TCGA-LIHC Cohort
B3
Cutting Point - CD8+ Cells
DFS Cutting Level
OS Cutting Level
Estimated Cell Fraction
TCGA-LIHC Cohort

Figure S9

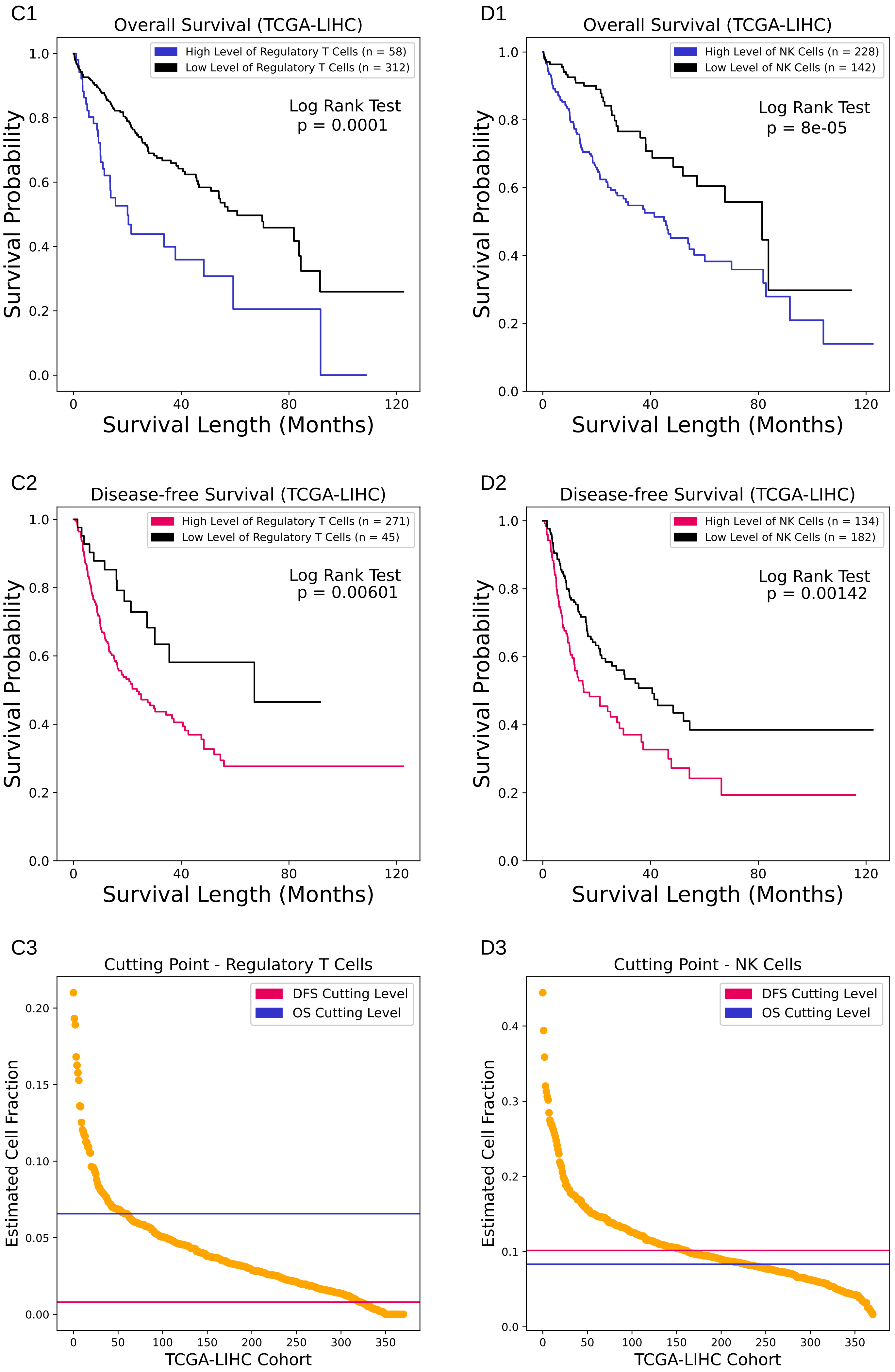
C1
Overall Survival (TCGA-LIHC)
High Level of Regulatory T Cells (n = 58)
Low Level of Regulatory T Cells (n = 312)
Log Rank Test
p = 0.0001
D1
Overall Survival (TCGA-LIHC)
High Level of NK Cells (n = 228)
Low Level of NK Cells (n = 142)
Log Rank Test
p = 8e-05
C2
Disease-free Survival (TCGA-LIHC)
High Level of Regulatory T Cells (n = 271)
Low Level of Regulatory T Cells (n = 45)
Log Rank Test
p = 0.00601
D2
Disease-free Survival (TCGA-LIHC)
High Level of NK Cells (n = 134)
Low Level of NK Cells (n = 182)
Log Rank Test
p = 0.00142
Survival Probability
Survival Length (Months)
C3
Cutting Point - Regulatory T Cells
D3
Cutting Point - NK Cells
DFS Cutting Level
OS Cutting Level
Estimated Cell Fraction
TCGA-LIHC Cohort

Figure S9

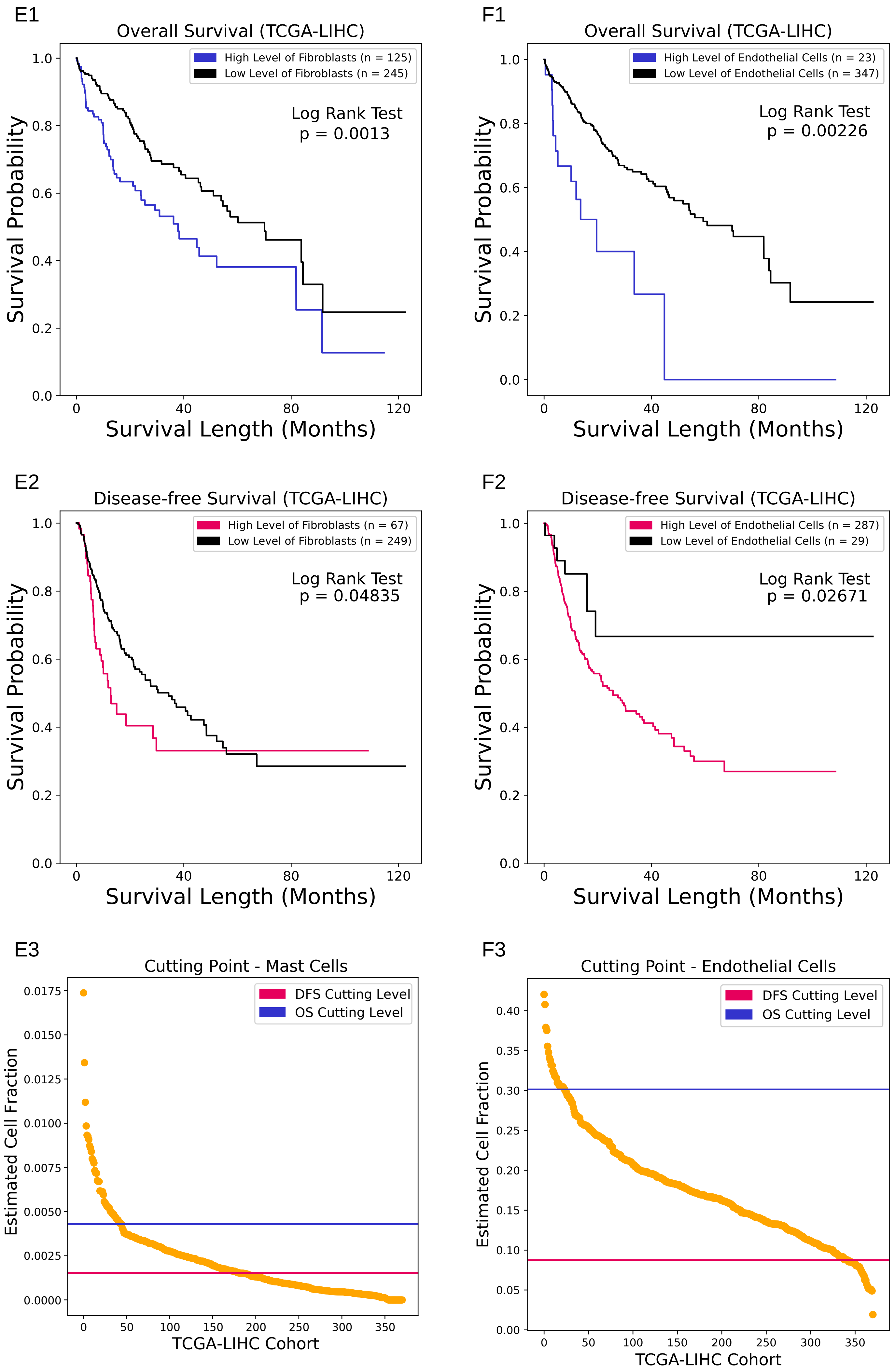
E1
Overall Survival (TCGA-LIHC)
High Level of Fibroblasts (n = 125)
Low Level of Fibroblasts (n = 245)
Log Rank Test
p = 0.0013
Survival Probability
Survival Length (Months)
F1
Overall Survival (TCGA-LIHC)
High Level of Endothelial Cells (n = 23)
Low Level of Endothelial Cells (n = 347)
Log Rank Test
p = 0.00226
Survival Probability
Survival Length (Months)
E2
Disease-free Survival (TCGA-LIHC)
High Level of Fibroblasts (n = 67)
Low Level of Fibroblasts (n = 249)
Log Rank Test
p = 0.04835
Survival Probability
Survival Length (Months)
F2
Disease-free Survival (TCGA-LIHC)
High Level of Endothelial Cells (n = 287)
Low Level of Endothelial Cells (n = 29)
Log Rank Test
p = 0.02671
Survival Probability
Survival Length (Months)
E3
Cutting Point - Mast Cells
DFS Cutting Level
OS Cutting Level
Estimated Cell Fraction
TCGA-LIHC Cohort
F3
Cutting Point - Endothelial Cells
DFS Cutting Level
OS Cutting Level
Estimated Cell Fraction
TCGA-LIHC Cohort

Figure S9

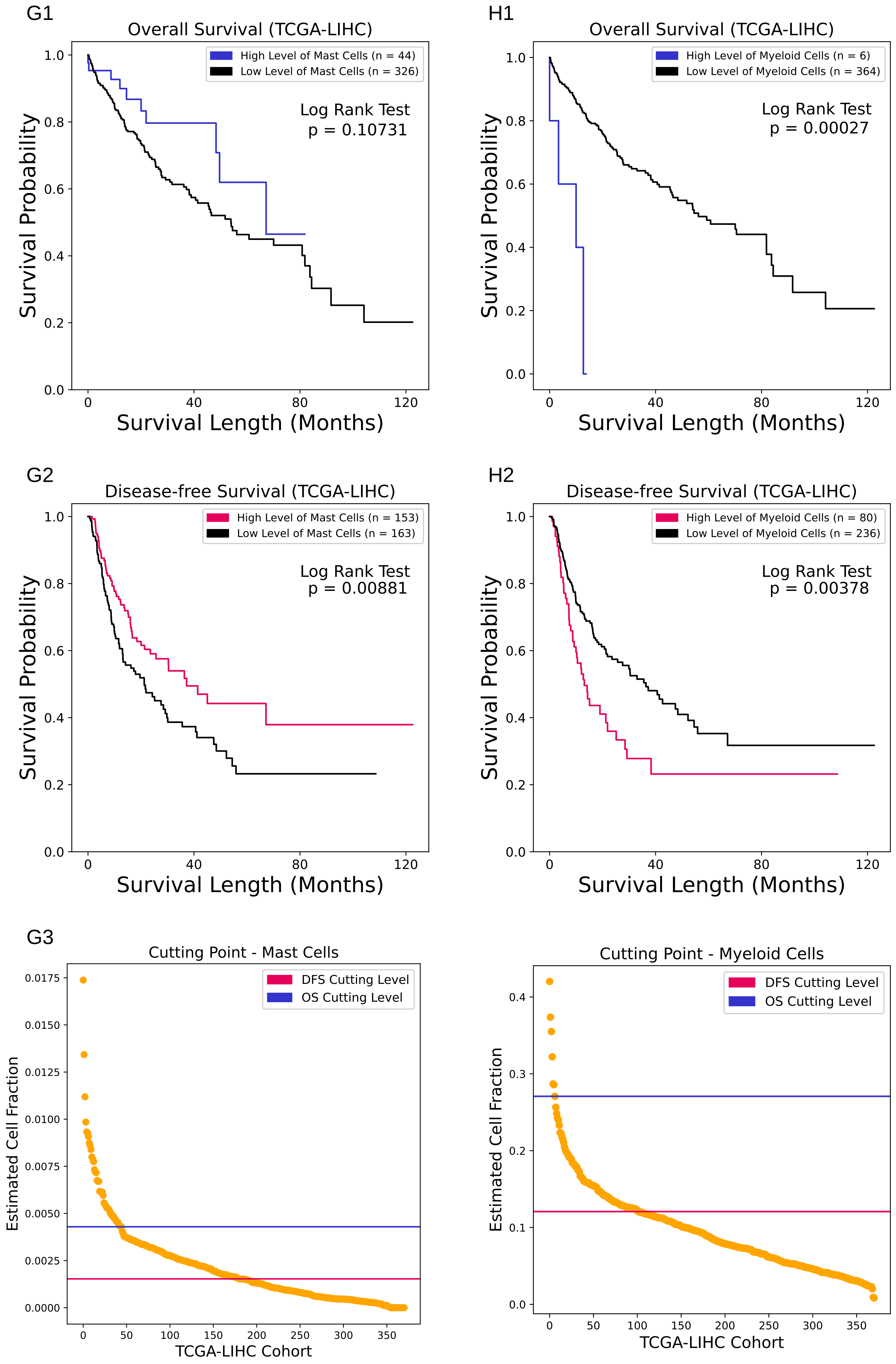
G1
Overall Survival (TCGA-LIHC)
High Level of Mast Cells (n = 44)
Low Level of Mast Cells (n = 326)
Log Rank Test
p = 0.10731
Survival Probability
Survival Length (Months)
H1
Overall Survival (TCGA-LIHC)
High Level of Myeloid Cells (n = 6)
Low Level of Myeloid Cells (n = 364)
Log Rank Test
p = 0.00027
Survival Probability
Survival Length (Months)
G2
Disease-free Survival (TCGA-LIHC)
High Level of Mast Cells (n = 153)
Low Level of Mast Cells (n = 163)
Log Rank Test
p = 0.00881
Survival Probability
Survival Length (Months)
H2
Disease-free Survival (TCGA-LIHC)
High Level of Myeloid Cells (n = 80)
Low Level of Myeloid Cells (n = 236)
Log Rank Test
p = 0.00378
Survival Probability
Survival Length (Months)
G3
Cutting Point - Mast Cells
DFS Cutting Level
OS Cutting Level
Estimated Cell Fraction
TCGA-LIHC Cohort
Cutting Point - Myeloid Cells
DFS Cutting Level
OS Cutting Level
Estimated Cell Fraction
TCGA-LIHC Cohort

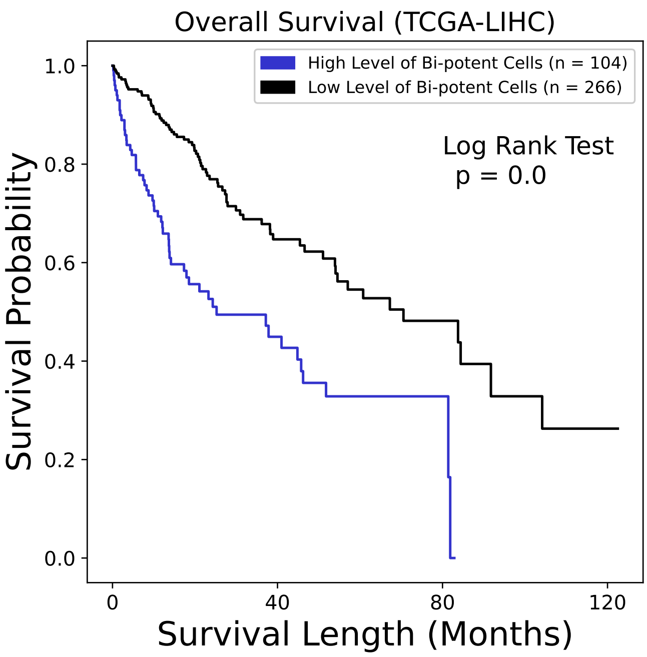
Overall Survival (TCGA-LIHC)
High Level of Bi-potent Cells (n = 104)
Low Level of Bi-potent Cells (n = 266)
Log Rank Test
p = 0.0
Survival Probability
Survival Length (Months)

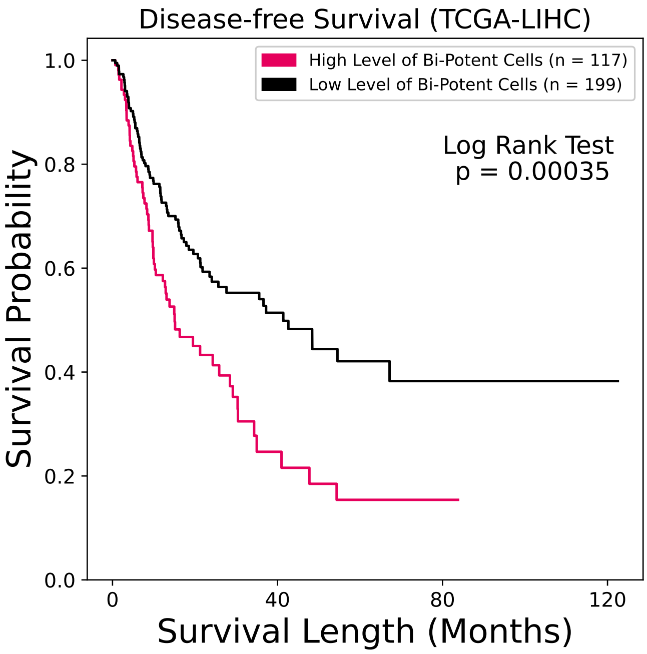
Disease-free Survival (TCGA-LIHC)
High Level of Bi-Potent Cells (n = 117)
Low Level of Bi-Potent Cells (n = 199)
Log Rank Test
p = 0.00035
Survival Probability
Survival Length (Months)

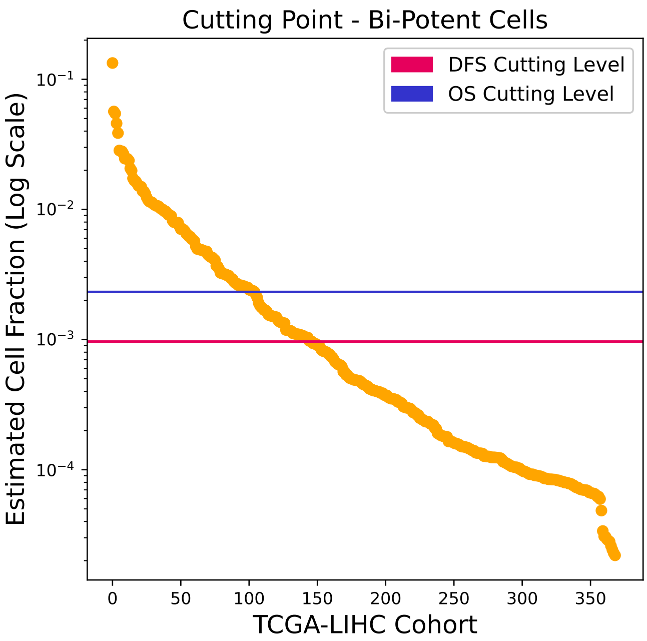
Cutting Point - Bi-Potent Cells
DFS Cutting Level
OS Cutting Level
Estimated Cell Fraction (Log Scale)
TCGA-LIHC Cohort
