# Supplementary material for: Deciphering Tumour Microenvironment of Liver Cancer through Deconvolution of Bulk RNA-Seq Data with Single-Cell Atlas
Source: Cancers (Basel). 2022 Dec 27;15(1):153. doi: 10.3390/cancers15010153 (PMC9818189; doi:10.3390/cancers15010153)

1

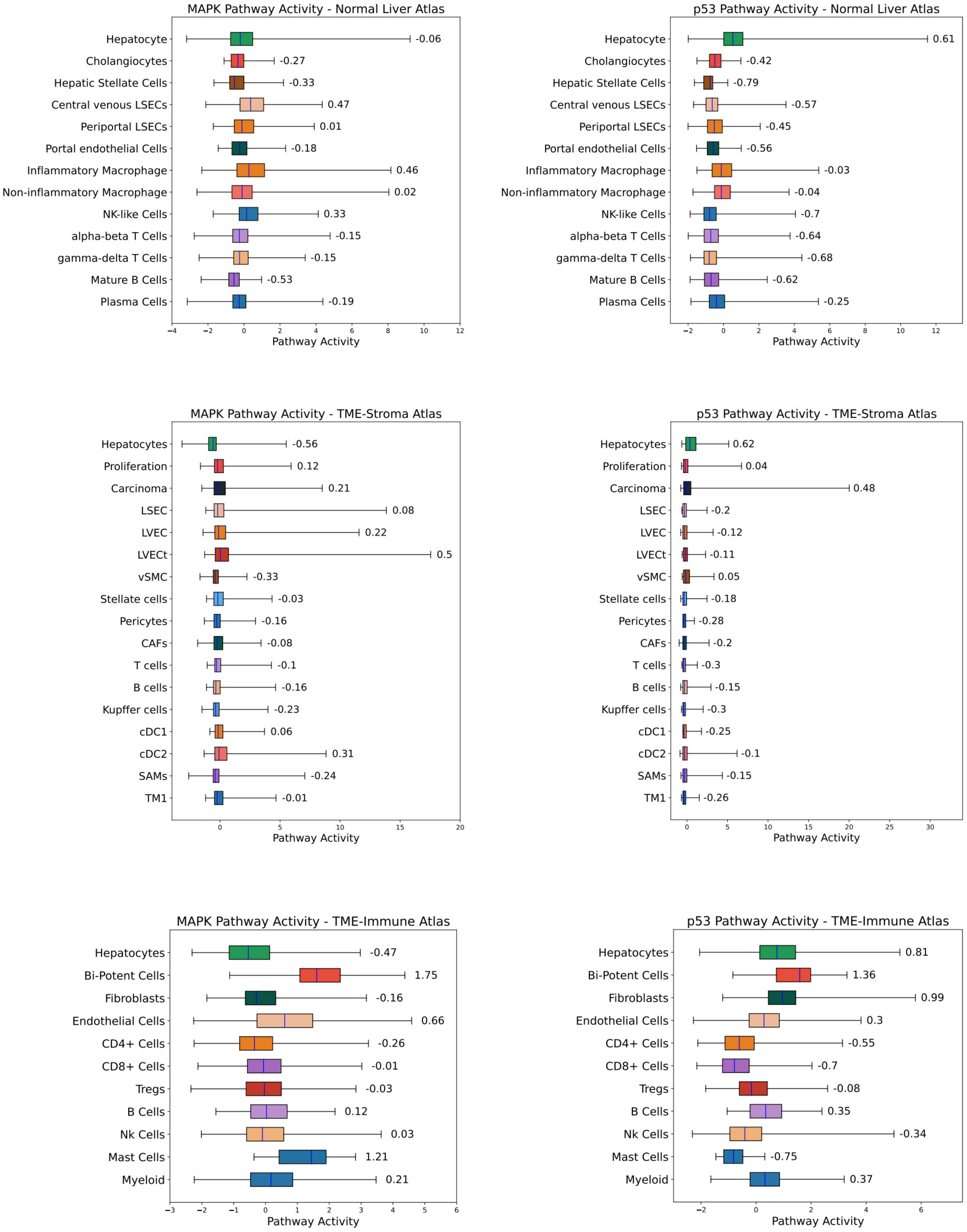
MAPK Pathway Activity - Normal Liver Atlas
Hepatocyte -0.06
Cholangiocytes -0.27
Hepatic Stellate Cells -0.33
Central venous LSECs 0.47
Periportal LSECs 0.01
Portal endothelial Cells -0.18
Inflammatory Macrophage 0.46
Non-inflammatory Macrophage 0.02
NK-like Cells 0.33
alpha-beta T Cells -0.15
gamma-delta T Cells -0.15
Mature B Cells -0.53
Plasma Cells -0.19
Pathway Activity
p53 Pathway Activity - Normal Liver Atlas
Hepatocyte 0.61
Cholangiocytes -0.42
Hepatic Stellate Cells -0.79
Central venous LSECs -0.57
Periportal LSECs -0.45
Portal endothelial Cells -0.56
Inflammatory Macrophage -0.03
Non-inflammatory Macrophage -0.04
NK-like Cells -0.7
alpha-beta T Cells -0.64
gamma-delta T Cells -0.68
Mature B Cells -0.62
Plasma Cells -0.25
Pathway Activity
MAPK Pathway Activity - TME-Stroma Atlas
Hepatocytes -0.56
Proliferation 0.12
Carcinoma 0.21
LSEC 0.08
LVEC 0.22
LVECt 0.5
vSMC -0.33
Stellate cells -0.03
Pericytes -0.16
CAFs -0.08
T cells -0.1
B cells -0.16
Kupffer cells -0.23
cDC1 0.06
cDC2 0.31
SAMs -0.24
TM1 -0.01
Pathway Activity
p53 Pathway Activity - TME-Stroma Atlas
Hepatocytes 0.62
Proliferation 0.04
Carcinoma 0.48
LSEC -0.2
LVEC -0.12
LVECt -0.11
vSMC 0.05
Stellate cells -0.18
Pericytes -0.28
CAFs -0.2
T cells -0.3
B cells -0.15
Kupffer cells -0.3
cDC1 -0.25
cDC2 -0.1
SAMs -0.15
TM1 -0.26
Pathway Activity
MAPK Pathway Activity - TME-Immune Atlas
Hepatocytes -0.47
Bi-Potent Cells 1.75
Fibroblasts -0.16
Endothelial Cells 0.66
CD4+ Cells -0.26
CD8+ Cells -0.01
Tregs -0.03
B Cells 0.12
Nk Cells 0.03
Mast Cells 1.21
Myeloid 0.21
Pathway Activity
p53 Pathway Activity - TME-Immune Atlas
Hepatocytes 0.81
Bi-Potent Cells 1.36
Fibroblasts 0.99
Endothelial Cells 0.3
CD4+ Cells -0.55
CD8+ Cells -0.7
Tregs -0.08
B Cells 0.35
Nk Cells -0.34
Mast Cells -0.75
Myeloid 0.37
Pathway Activity

2

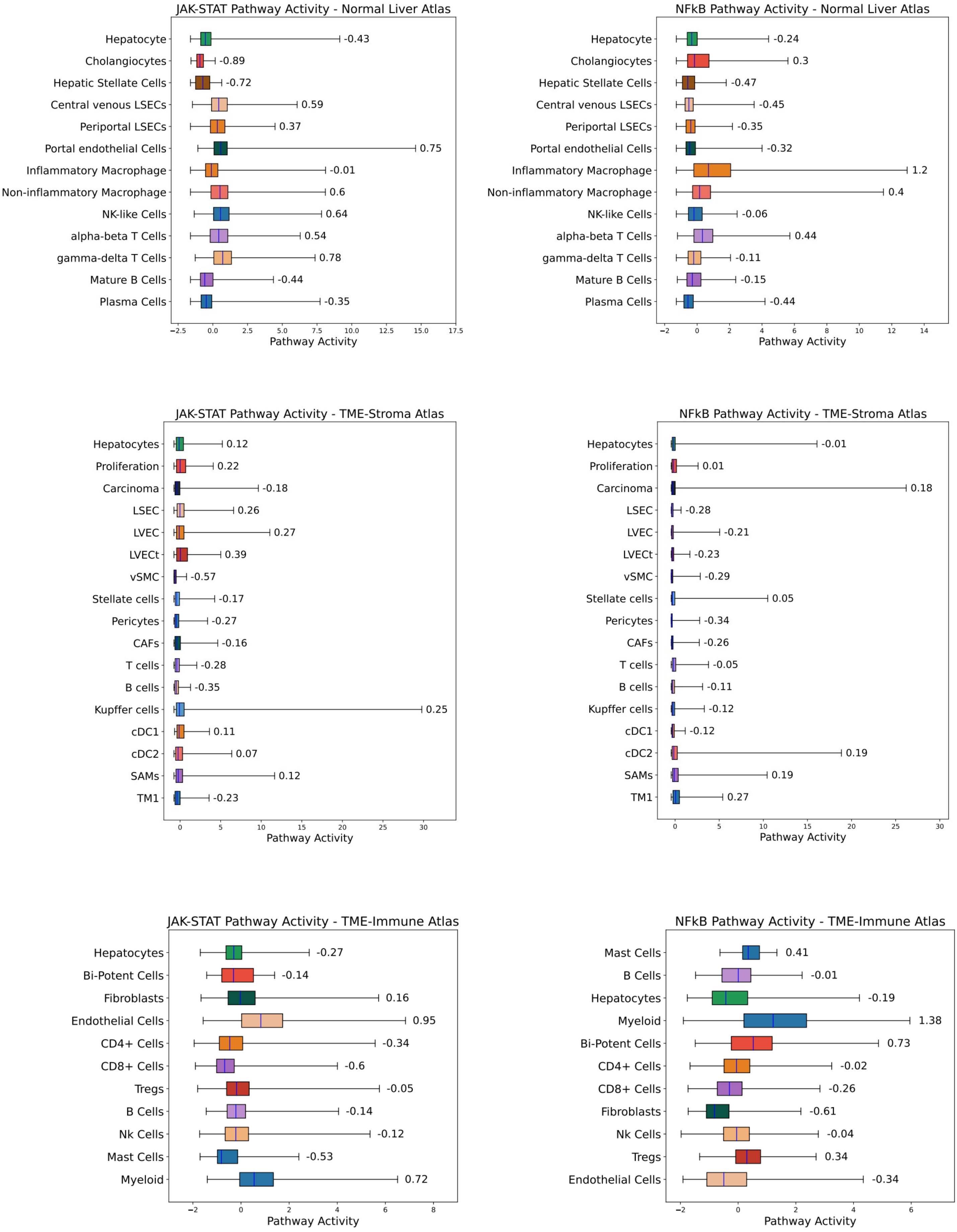
JAK-STAT Pathway Activity - Normal Liver Atlas
Hepatocyte -0.43
Cholangiocytes -0.89
Hepatic Stellate Cells -0.72
Central venous LSECs 0.59
Periportal LSECs 0.37
Portal endothelial Cells 0.75
Inflammatory Macrophage -0.01
Non-inflammatory Macrophage 0.6
NK-like Cells 0.64
alpha-beta T Cells 0.54
gamma-delta T Cells 0.78
Mature B Cells -0.44
Plasma Cells -0.35
Pathway Activity
NFkB Pathway Activity - Normal Liver Atlas
Hepatocyte -0.24
Cholangiocytes 0.3
Hepatic Stellate Cells -0.47
Central venous LSECs -0.45
Periportal LSECs -0.35
Portal endothelial Cells -0.32
Inflammatory Macrophage 1.2
Non-inflammatory Macrophage 0.4
NK-like Cells -0.06
alpha-beta T Cells 0.44
gamma-delta T Cells -0.11
Mature B Cells -0.15
Plasma Cells -0.44
Pathway Activity
JAK-STAT Pathway Activity - TME-Stroma Atlas
Hepatocytes 0.12
Proliferation 0.22
Carcinoma -0.18
LSEC 0.26
LVEC 0.27
LVECt 0.39
vSMC -0.57
Stellate cells -0.17
Pericytes -0.27
CAFs -0.16
T cells -0.28
B cells -0.35
Kupffer cells 0.25
cDC1 0.11
cDC2 0.07
SAMs 0.12
TM1 -0.23
Pathway Activity
NFkB Pathway Activity - TME-Stroma Atlas
Hepatocytes -0.01
Proliferation 0.01
Carcinoma 0.18
LSEC -0.28
LVEC -0.21
LVECt -0.23
vSMC -0.29
Stellate cells 0.05
Pericytes -0.34
CAFs -0.26
T cells -0.05
B cells -0.11
Kupffer cells -0.12
cDC1 -0.12
cDC2 0.19
SAMs 0.19
TM1 0.27
Pathway Activity
JAK-STAT Pathway Activity - TME-Immune Atlas
Hepatocytes -0.27
Bi-Potent Cells -0.14
Fibroblasts 0.16
Endothelial Cells 0.95
CD4+ Cells -0.34
CD8+ Cells -0.6
Tregs -0.05
B Cells -0.14
Nk Cells -0.12
Mast Cells -0.53
Myeloid 0.72
Pathway Activity
NFkB Pathway Activity - TME-Immune Atlas
Mast Cells 0.41
B Cells -0.01
Hepatocytes -0.19
Myeloid 1.38
Bi-Potent Cells 0.73
CD4+ Cells -0.02
CD8+ Cells -0.26
Fibroblasts -0.61
Nk Cells -0.04
Tregs 0.34
Endothelial Cells -0.34
Pathway Activity

3

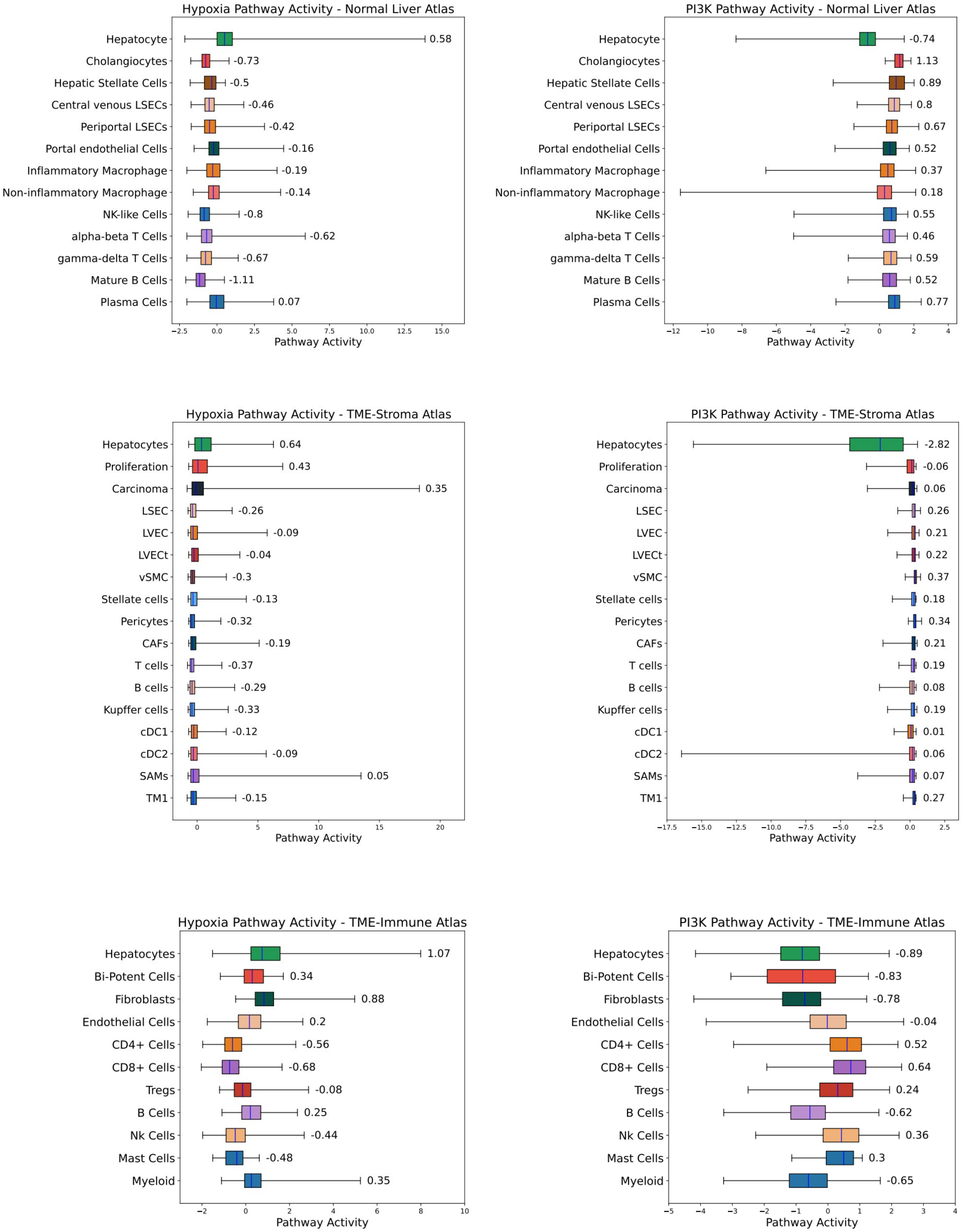
Hypoxia Pathway Activity - Normal Liver Atlas
Hepatocyte 0.58
Cholangiocytes -0.73
Hepatic Stellate Cells -0.5
Central venous LSECs -0.46
Periportal LSECs -0.42
Portal endothelial Cells -0.16
Inflammatory Macrophage -0.19
Non-inflammatory Macrophage -0.14
NK-like Cells -0.8
alpha-beta T Cells -0.62
gamma-delta T Cells -0.67
Mature B Cells -1.11
Plasma Cells 0.07
−2.5 0.0 2.5 5.0 7.5 10.0 12.5 15.0
Pathway Activity
PI3K Pathway Activity - Normal Liver Atlas
Hepatocyte -0.74
Cholangiocytes 1.13
Hepatic Stellate Cells 0.89
Central venous LSECs 0.8
Periportal LSECs 0.67
Portal endothelial Cells 0.52
Inflammatory Macrophage 0.37
Non-inflammatory Macrophage 0.18
NK-like Cells 0.55
alpha-beta T Cells 0.46
gamma-delta T Cells 0.59
Mature B Cells 0.52
Plasma Cells 0.77
−12 −10 −8 −6 −4 −2 0 2 4
Pathway Activity
Hypoxia Pathway Activity - TME-Stroma Atlas
Hepatocytes 0.64
Proliferation 0.43
Carcinoma 0.35
LSEC -0.26
LVEC -0.09
LVECt -0.04
vSMC -0.3
Stellate cells -0.13
Pericytes -0.32
CAFs -0.19
T cells -0.37
B cells -0.29
Kupffer cells -0.33
cDC1 -0.12
cDC2 -0.09
SAMs 0.05
TM1 -0.15
0 5 10 15 20
Pathway Activity
PI3K Pathway Activity - TME-Stroma Atlas
Hepatocytes -2.82
Proliferation -0.06
Carcinoma 0.06
LSEC 0.26
LVEC 0.21
LVECt 0.22
vSMC 0.37
Stellate cells 0.18
Pericytes 0.34
CAFs 0.21
T cells 0.19
B cells 0.08
Kupffer cells 0.19
cDC1 0.01
cDC2 0.06
SAMs 0.07
TM1 0.27
−17.5 −15.0 −12.5 −10.0 −7.5 −5.0 −2.5 0.0 2.5
Pathway Activity
Hypoxia Pathway Activity - TME-Immune Atlas
Hepatocytes 1.07
Bi-Potent Cells 0.34
Fibroblasts 0.88
Endothelial Cells 0.2
CD4+ Cells -0.56
CD8+ Cells -0.68
Tregs -0.08
B Cells 0.25
Nk Cells -0.44
Mast Cells -0.48
Myeloid 0.35
−2 0 2 4 6 8 10
Pathway Activity
PI3K Pathway Activity - TME-Immune Atlas
Hepatocytes -0.89
Bi-Potent Cells -0.83
Fibroblasts -0.78
Endothelial Cells -0.04
CD4+ Cells 0.52
CD8+ Cells 0.64
Tregs 0.24
B Cells -0.62
Nk Cells 0.36
Mast Cells 0.3
Myeloid -0.65
−5 −4 −3 −2 −1 0 1 2 3 4
Pathway Activity

4

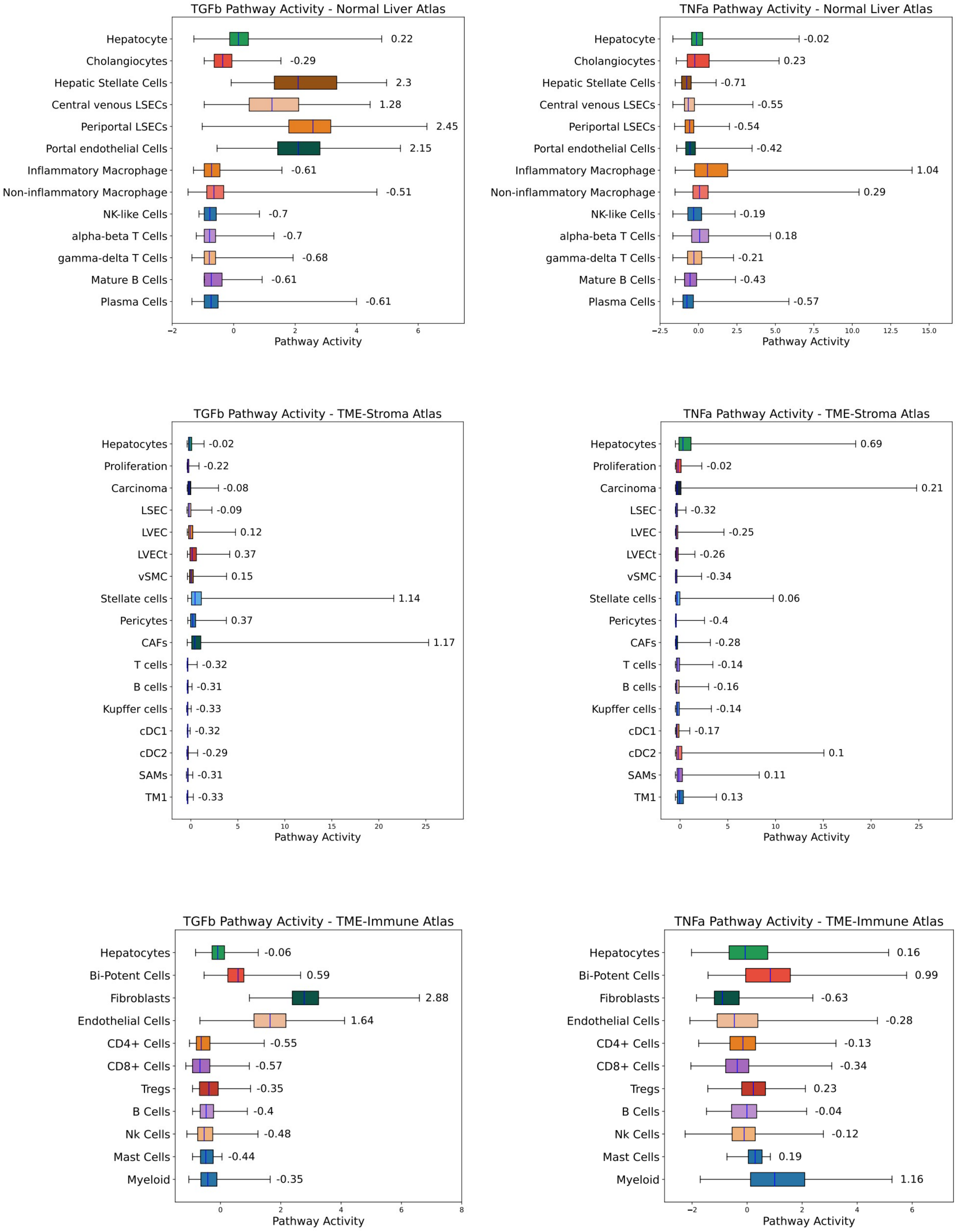

TGFb Pathway Activity - Normal Liver Atlas
Hepatocyte
Cholangiocytes
Hepatic Stellate Cells
Central venous LSECs
Periportal LSECs
Portal endothelial Cells
Inflammatory Macrophage
Non-inflammatory Macrophage
NK-like Cells
alpha-beta T Cells
gamma-delta T Cells
Mature B Cells
Plasma Cells
0.22
-0.29
2.3
1.28
2.45
2.15
-0.61
-0.51
-0.7
-0.7
-0.68
-0.61
-0.61
−2
0
2
4
6
Pathway Activity
TNFa Pathway Activity - Normal Liver Atlas
Hepatocyte
Cholangiocytes
Hepatic Stellate Cells
Central venous LSECs
Periportal LSECs
Portal endothelial Cells
Inflammatory Macrophage
Non-inflammatory Macrophage
NK-like Cells
alpha-beta T Cells
gamma-delta T Cells
Mature B Cells
Plasma Cells
-0.02
0.23
-0.71
-0.55
-0.54
-0.42
1.04
0.29
-0.19
0.18
-0.21
-0.43
-0.57
−2.5
0.0
2.5
5.0
7.5
10.0
12.5
15.0
Pathway Activity
TGFb Pathway Activity - TME-Stroma Atlas
Hepatocytes
Proliferation
Carcinoma
LSEC
LVEC
LVECt
vSMC
Stellate cells
Pericytes
CAFs
T cells
B cells
Kupffer cells
cDC1
cDC2
SAMs
TM1
-0.02
-0.22
-0.08
-0.09
0.12
0.37
0.15
1.14
0.37
1.17
-0.32
-0.31
-0.33
-0.32
-0.29
-0.31
-0.33
0
5
10
15
20
25
Pathway Activity
TNFa Pathway Activity - TME-Stroma Atlas
Hepatocytes
Proliferation
Carcinoma
LSEC
LVEC
LVECt
vSMC
Stellate cells
Pericytes
CAFs
T cells
B cells
Kupffer cells
cDC1
cDC2
SAMs
TM1
0.69
-0.02
0.21
-0.32
-0.25
-0.26
-0.34
0.06
-0.4
-0.28
-0.14
-0.16
-0.14
-0.17
0.1
0.11
0.13
0
5
10
15
20
25
Pathway Activity
TGFb Pathway Activity - TME-Immune Atlas
Hepatocytes
Bi-Potent Cells
Fibroblasts
Endothelial Cells
CD4+ Cells
CD8+ Cells
Tregs
B Cells
Nk Cells
Mast Cells
Myeloid
-0.06
0.59
2.88
1.64
-0.55
-0.57
-0.35
-0.4
-0.48
-0.44
-0.35
0
2
4
6
8
Pathway Activity
TNFa Pathway Activity - TME-Immune Atlas
Hepatocytes
Bi-Potent Cells
Fibroblasts
Endothelial Cells
CD4+ Cells
CD8+ Cells
Tregs
B Cells
Nk Cells
Mast Cells
Myeloid
0.16
0.99
-0.63
-0.28
-0.13
-0.34
0.23
-0.04
-0.12
0.19
1.16
−2
0
2
4
6
Pathway Activity

5

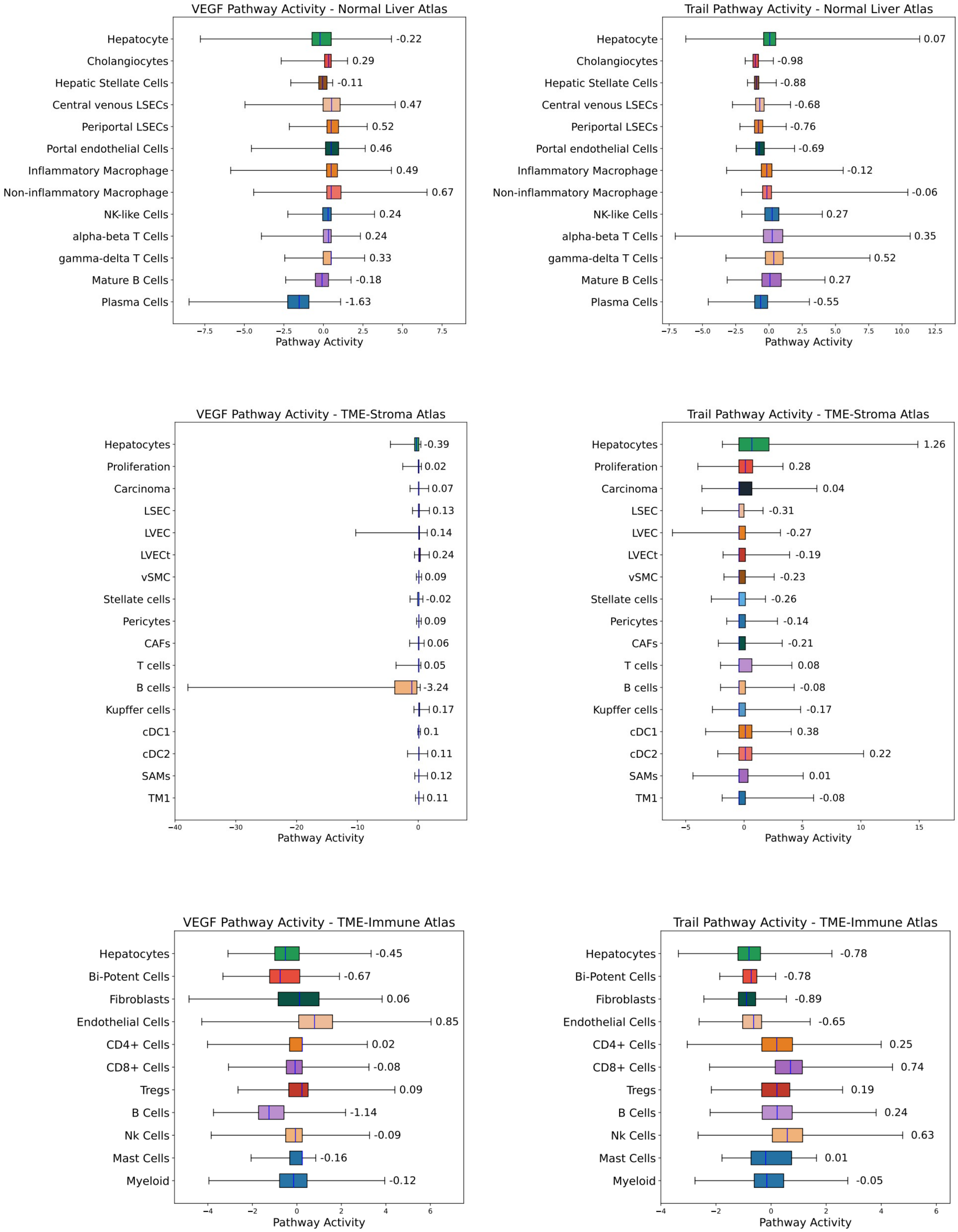

VEGF Pathway Activity - Normal Liver Atlas
Hepatocyte -0.22
Cholangiocytes 0.29
Hepatic Stellate Cells -0.11
Central venous LSECs 0.47
Periportal LSECs 0.52
Portal endothelial Cells 0.46
Inflammatory Macrophage 0.49
Non-inflammatory Macrophage 0.67
NK-like Cells 0.24
alpha-beta T Cells 0.24
gamma-delta T Cells 0.33
Mature B Cells -0.18
Plasma Cells -1.63
−7.5 −5.0 −2.5 0.0 2.5 5.0 7.5
Pathway Activity
Trail Pathway Activity - Normal Liver Atlas
Hepatocyte 0.07
Cholangiocytes -0.98
Hepatic Stellate Cells -0.88
Central venous LSECs -0.68
Periportal LSECs -0.76
Portal endothelial Cells -0.69
Inflammatory Macrophage -0.12
Non-inflammatory Macrophage -0.06
NK-like Cells 0.27
alpha-beta T Cells 0.35
gamma-delta T Cells 0.52
Mature B Cells 0.27
Plasma Cells -0.55
−7.5 −5.0 −2.5 0.0 2.5 5.0 7.5 10.0 12.5
Pathway Activity
VEGF Pathway Activity - TME-Stroma Atlas
Hepatocytes -0.39
Proliferation 0.02
Carcinoma 0.07
LSEC 0.13
LVEC 0.14
LVECt 0.24
vSMC 0.09
Stellate cells -0.02
Pericytes 0.09
CAFs 0.06
T cells 0.05
B cells -3.24
Kupffer cells 0.17
cDC1 0.1
cDC2 0.11
SAMs 0.12
TM1 0.11
−40 −30 −20 −10 0
Pathway Activity
Trail Pathway Activity - TME-Stroma Atlas
Hepatocytes 1.26
Proliferation 0.28
Carcinoma 0.04
LSEC -0.31
LVEC -0.27
LVECt -0.19
vSMC -0.23
Stellate cells -0.26
Pericytes -0.14
CAFs -0.21
T cells 0.08
B cells -0.08
Kupffer cells -0.17
cDC1 0.38
cDC2 0.22
SAMs 0.01
TM1 -0.08
−5 0 5 10 15
Pathway Activity
VEGF Pathway Activity - TME-Immune Atlas
Hepatocytes -0.45
Bi-Potent Cells -0.67
Fibroblasts 0.06
Endothelial Cells 0.85
CD4+ Cells 0.02
CD8+ Cells -0.08
Tregs 0.09
B Cells -1.14
Nk Cells -0.09
Mast Cells -0.16
Myeloid -0.12
−4 −2 0 2 4 6
Pathway Activity
Trail Pathway Activity - TME-Immune Atlas
Hepatocytes -0.78
Bi-Potent Cells -0.78
Fibroblasts -0.89
Endothelial Cells -0.65
CD4+ Cells 0.25
CD8+ Cells 0.74
Tregs 0.19
B Cells 0.24
Nk Cells 0.63
Mast Cells 0.01
Myeloid -0.05
−4 −2 0 2 4 6
Pathway Activity

6

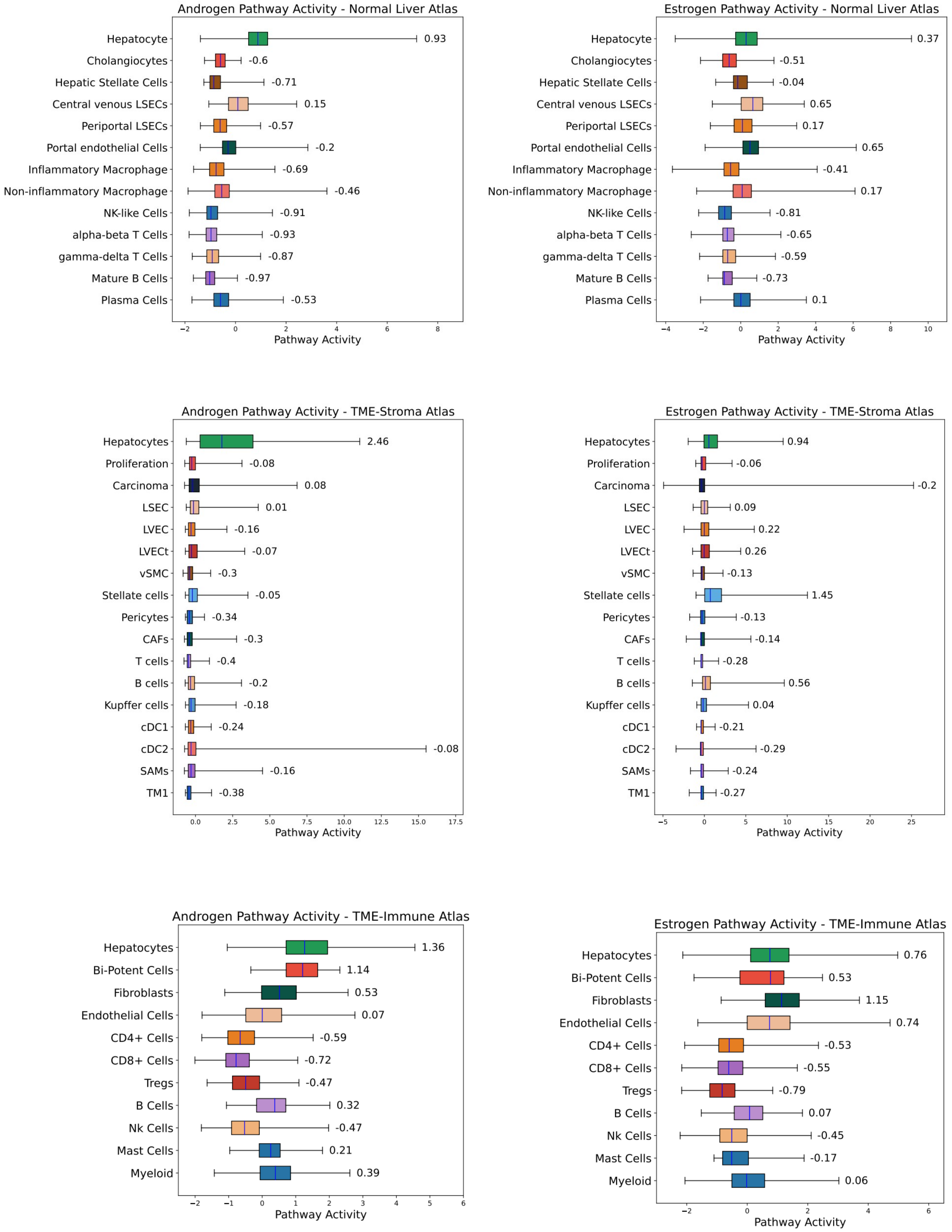

Androgen Pathway Activity - Normal Liver Atlas
Hepatocyte 0.93
Cholangiocytes -0.6
Hepatic Stellate Cells -0.71
Central venous LSECs 0.15
Periportal LSECs -0.57
Portal endothelial Cells -0.2
Inflammatory Macrophage -0.69
Non-inflammatory Macrophage -0.46
NK-like Cells -0.91
alpha-beta T Cells -0.93
gamma-delta T Cells -0.87
Mature B Cells -0.97
Plasma Cells -0.53
Pathway Activity
Estrogen Pathway Activity - Normal Liver Atlas
Hepatocyte 0.37
Cholangiocytes -0.51
Hepatic Stellate Cells -0.04
Central venous LSECs 0.65
Periportal LSECs 0.17
Portal endothelial Cells 0.65
Inflammatory Macrophage -0.41
Non-inflammatory Macrophage 0.17
NK-like Cells -0.81
alpha-beta T Cells -0.65
gamma-delta T Cells -0.59
Mature B Cells -0.73
Plasma Cells 0.1
Pathway Activity
Androgen Pathway Activity - TME-Stroma Atlas
Hepatocytes 2.46
Proliferation -0.08
Carcinoma 0.08
LSEC 0.01
LVEC -0.16
LVECt -0.07
vSMC -0.3
Stellate cells -0.05
Pericytes -0.34
CAFs -0.3
T cells -0.4
B cells -0.2
Kupffer cells -0.18
cDC1 -0.24
cDC2 -0.08
SAMs -0.16
TM1 -0.38
Pathway Activity
Estrogen Pathway Activity - TME-Stroma Atlas
Hepatocytes 0.94
Proliferation -0.06
Carcinoma -0.2
LSEC 0.09
LVEC 0.22
LVECt 0.26
vSMC -0.13
Stellate cells 1.45
Pericytes -0.13
CAFs -0.14
T cells -0.28
B cells 0.56
Kupffer cells 0.04
cDC1 -0.21
cDC2 -0.29
SAMs -0.24
TM1 -0.27
Pathway Activity
Androgen Pathway Activity - TME-Immune Atlas
Hepatocytes 1.36
Bi-Potent Cells 1.14
Fibroblasts 0.53
Endothelial Cells 0.07
CD4+ Cells -0.59
CD8+ Cells -0.72
Tregs -0.47
B Cells 0.32
Nk Cells -0.47
Mast Cells 0.21
Myeloid 0.39
Pathway Activity
Estrogen Pathway Activity - TME-Immune Atlas
Hepatocytes 0.76
Bi-Potent Cells 0.53
Fibroblasts 1.15
Endothelial Cells 0.74
CD4+ Cells -0.53
CD8+ Cells -0.55
Tregs -0.79
B Cells 0.07
Nk Cells -0.45
Mast Cells -0.17
Myeloid 0.06
Pathway Activity

Alpha-beta T cell (Normal Liver atlas)

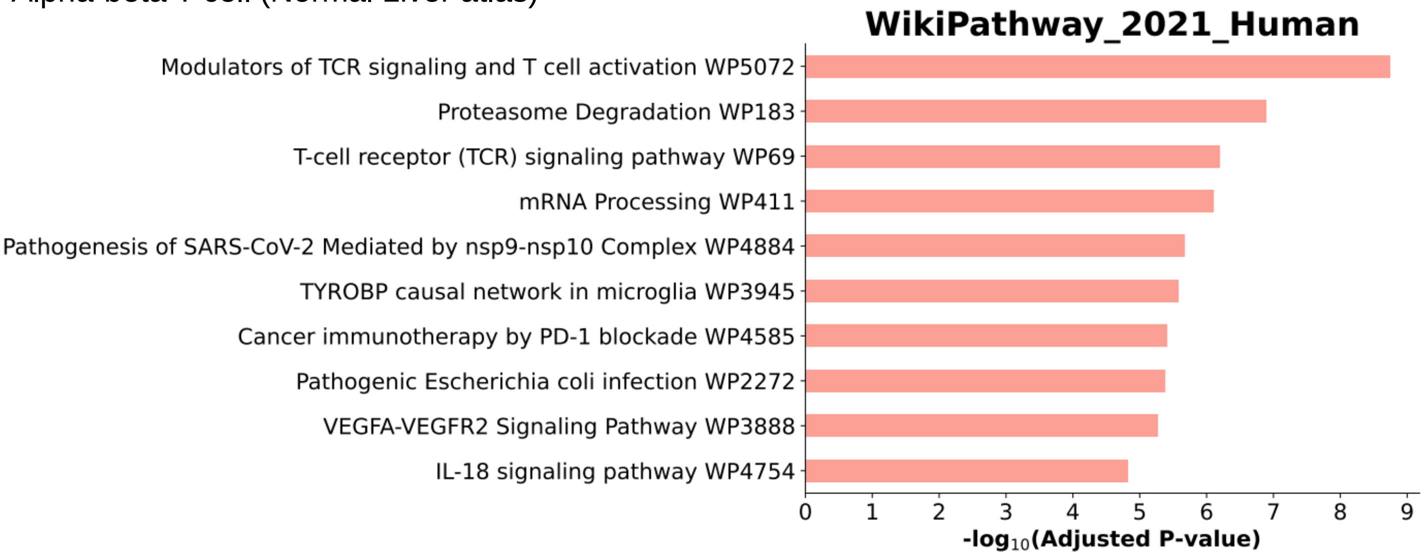

Central Venous LSEC (Normal Liver atlas)

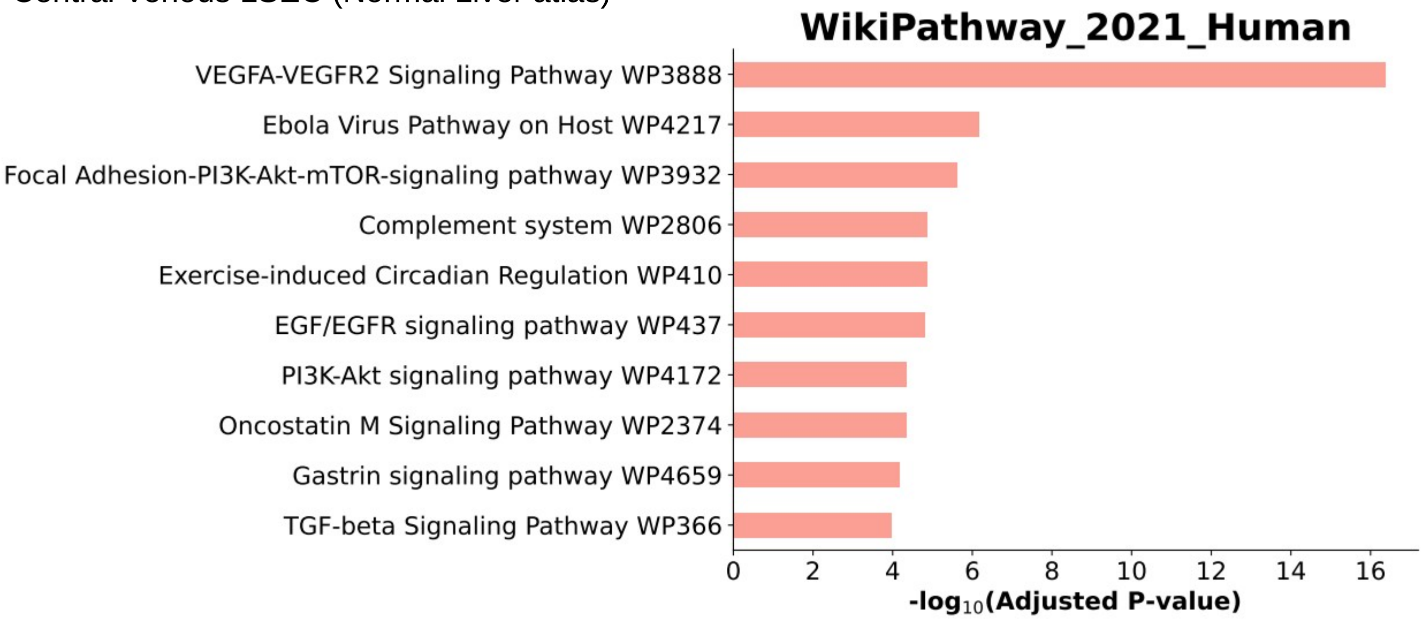

Cholangiocyte (Normal Liver atlas)

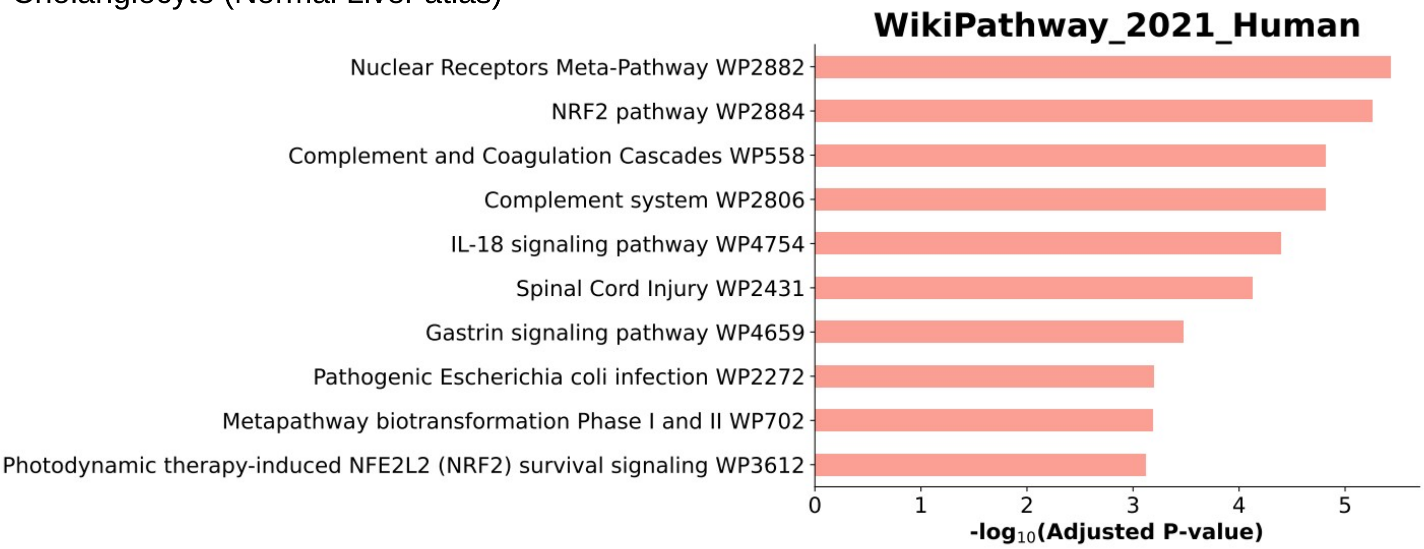

Erythroid cell (Normal Liver atlas)

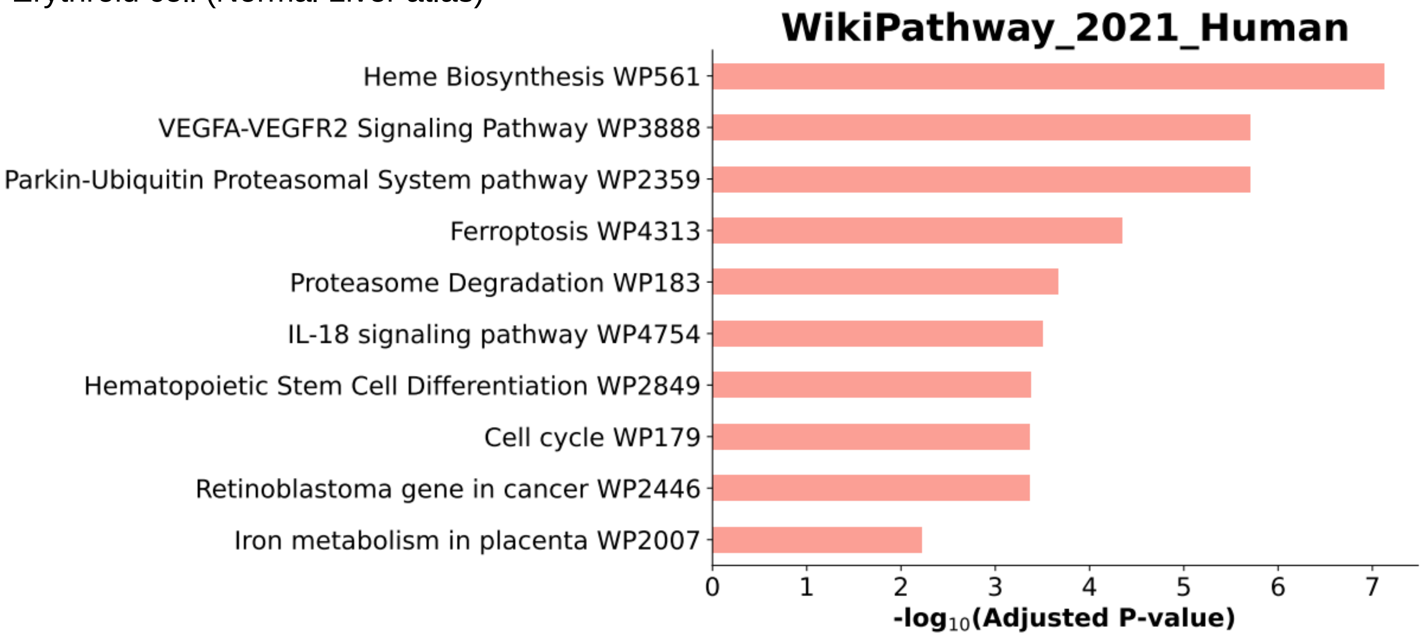

Gamma-delta T cell (Normal Liver atlas)

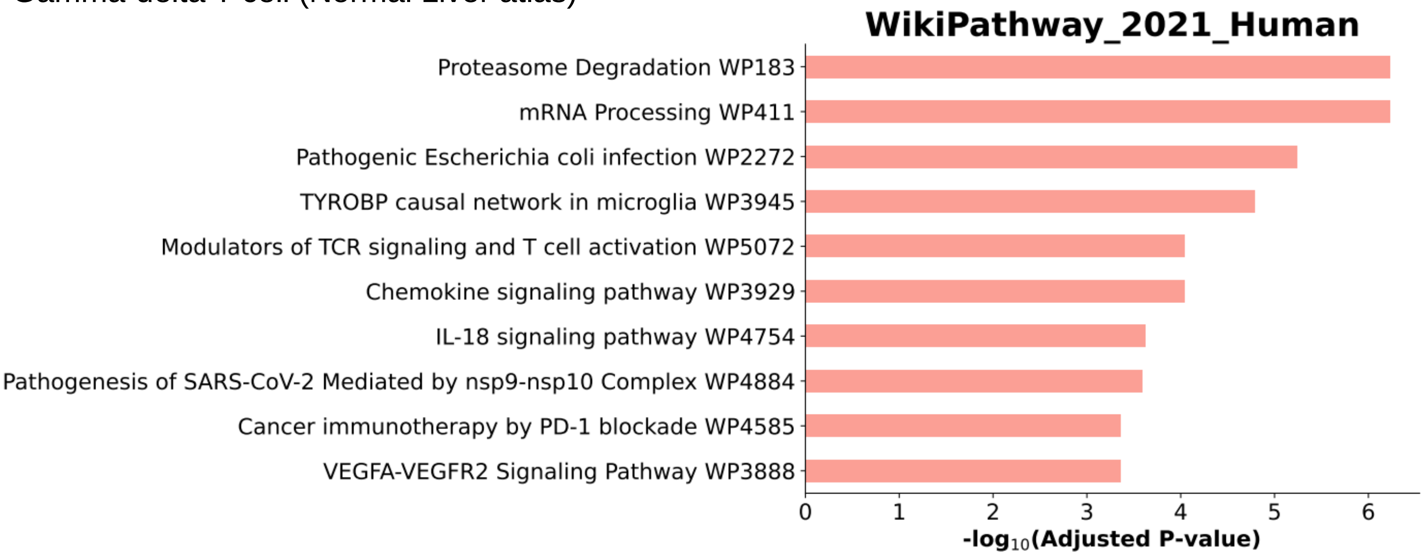

Hepatic stellate cell (Normal Liver atlas)

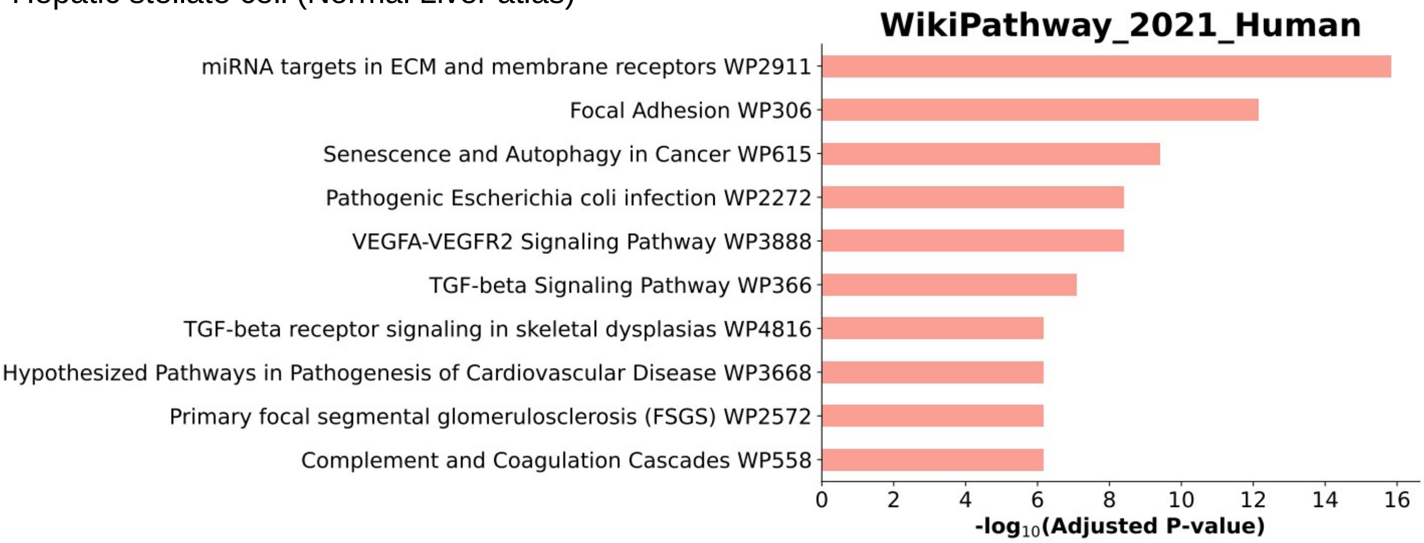

Hepatocyte (Normal Liver atlas)

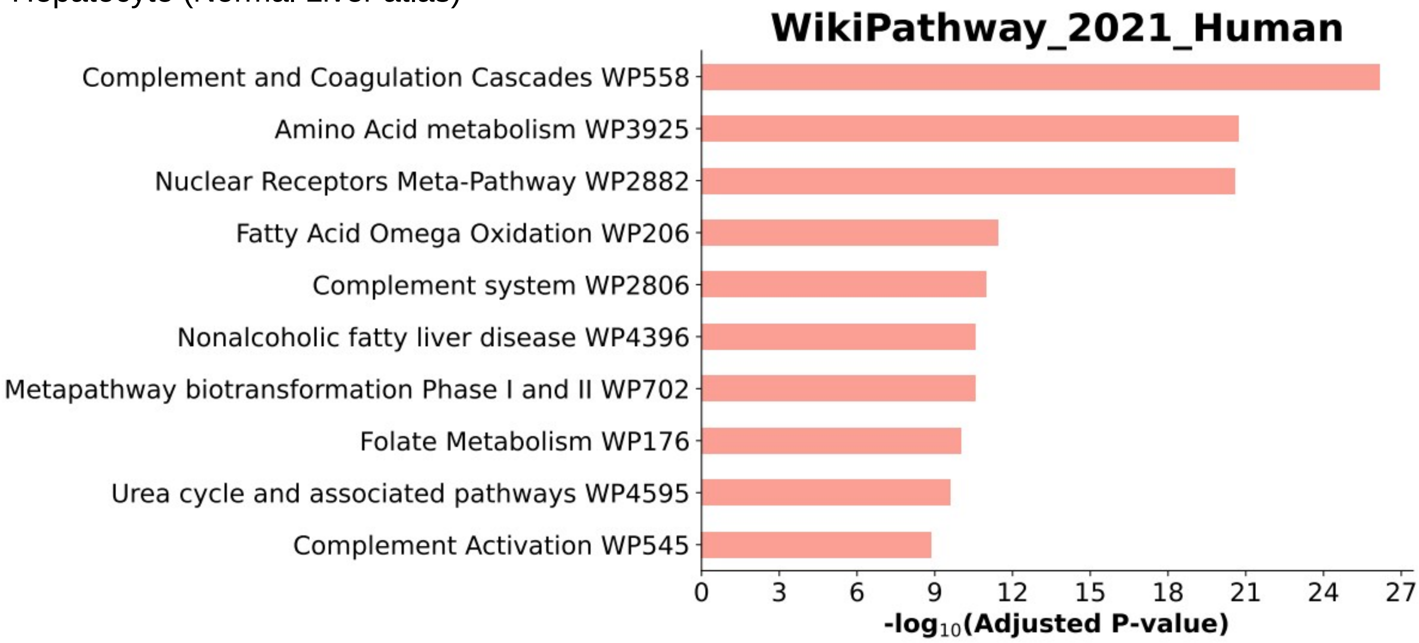

Inflammatory macrophage (Normal Liver atlas)

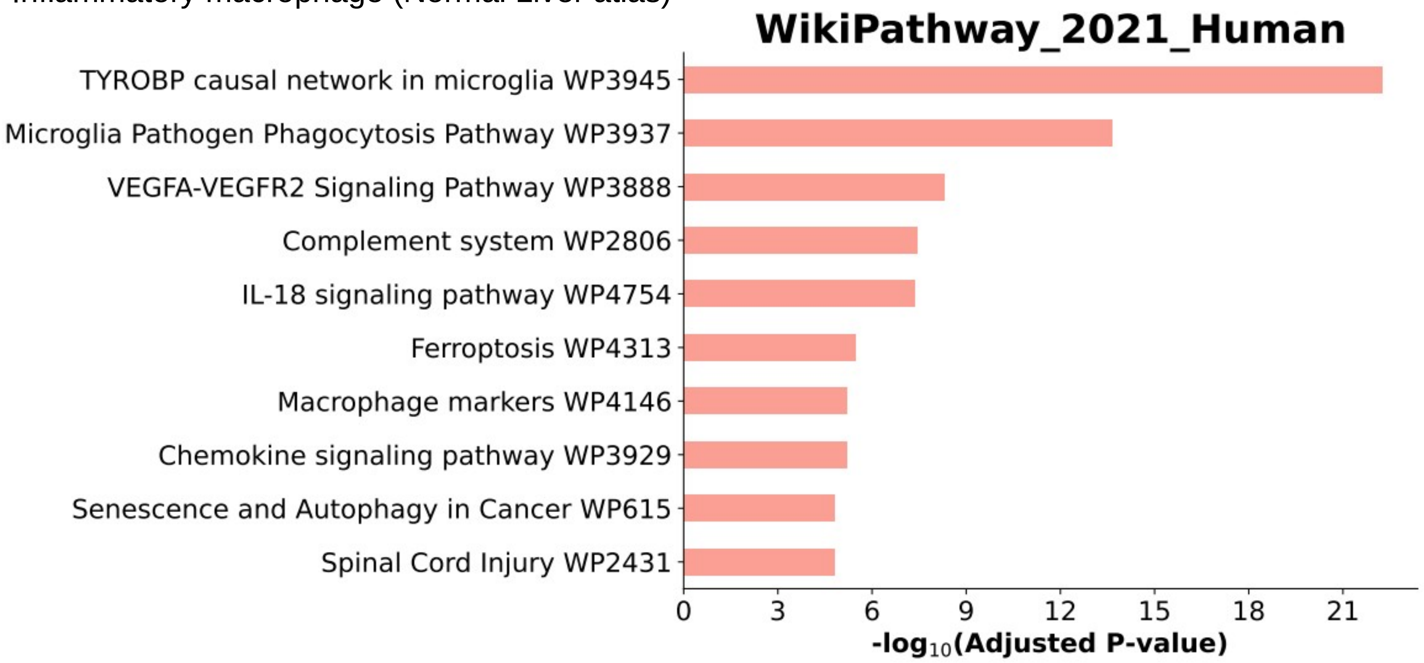

Mature B cell (Normal Liver atlas)

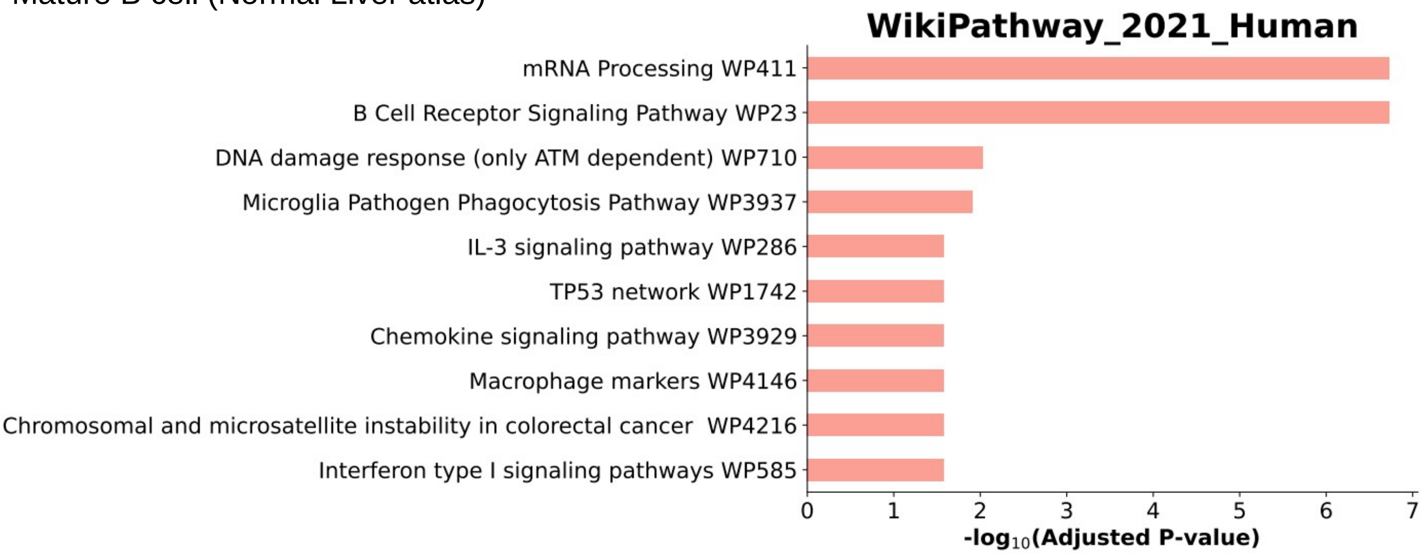

NK-like cell (Normal Liver atlas)

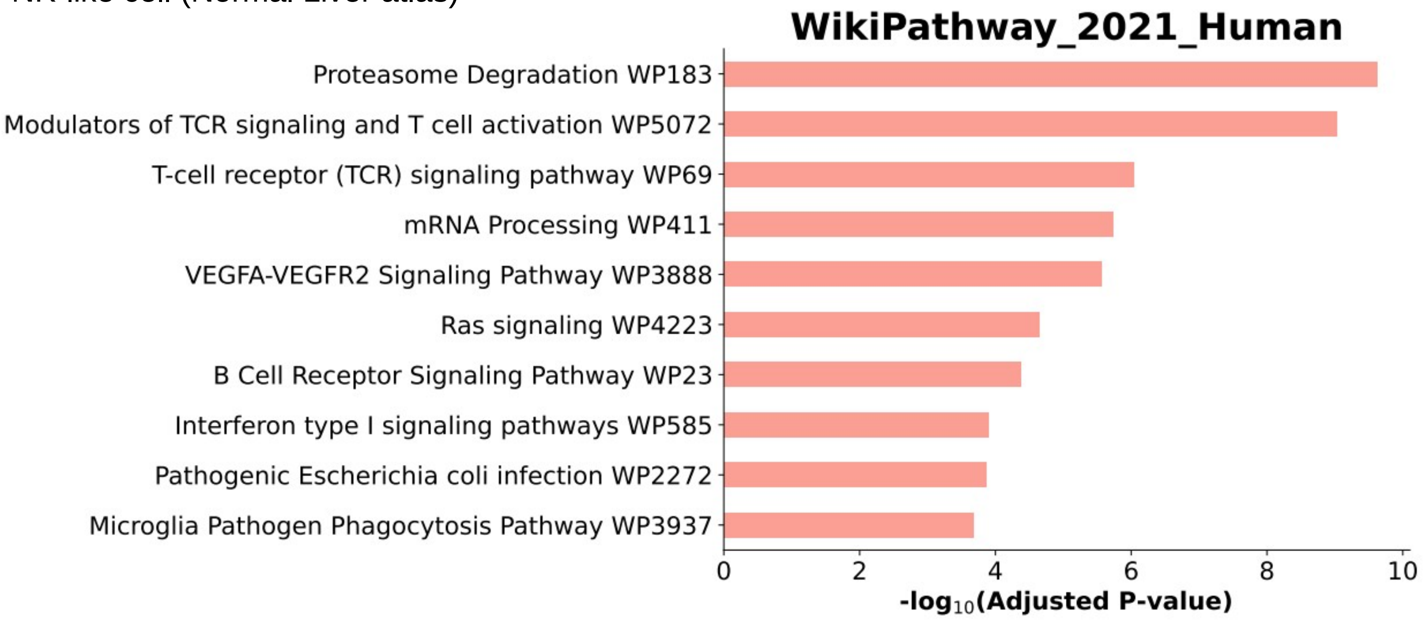

Non-inflammatory macrophage (Normal Liver atlas)

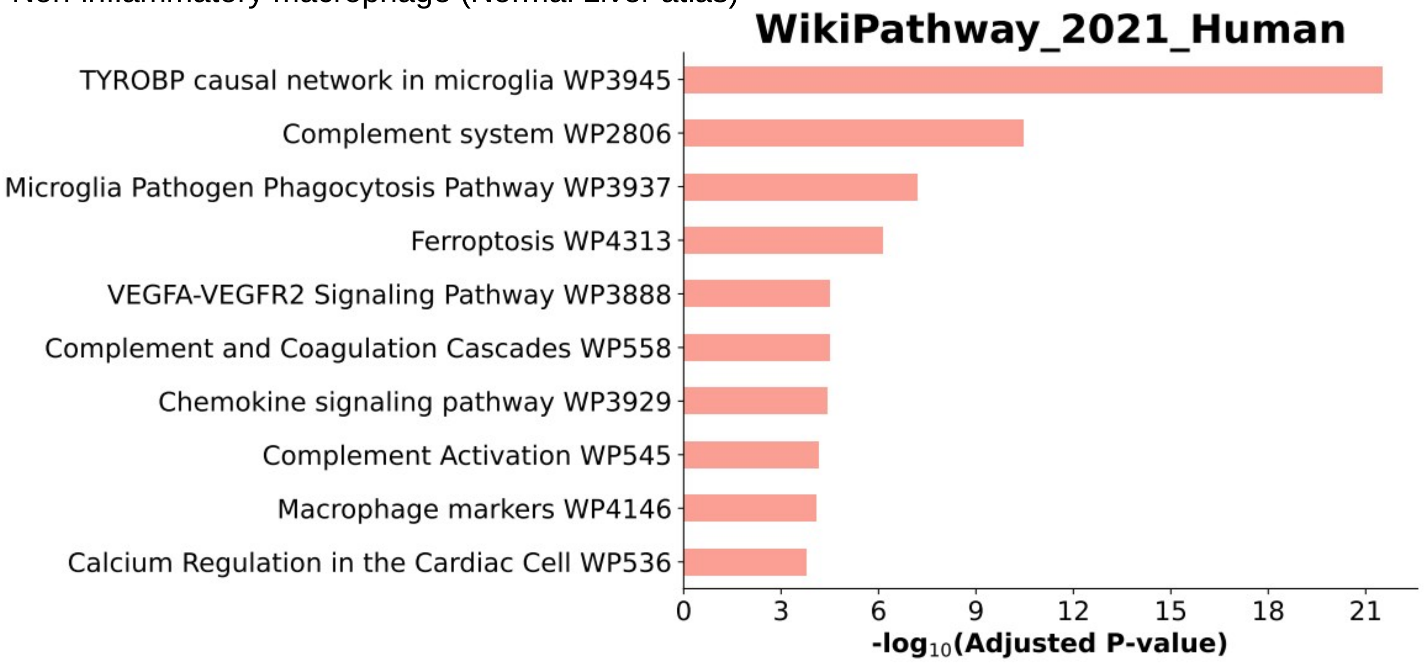

Periportal liver sinusoidal endothelial cell(Normal Liver atlas)

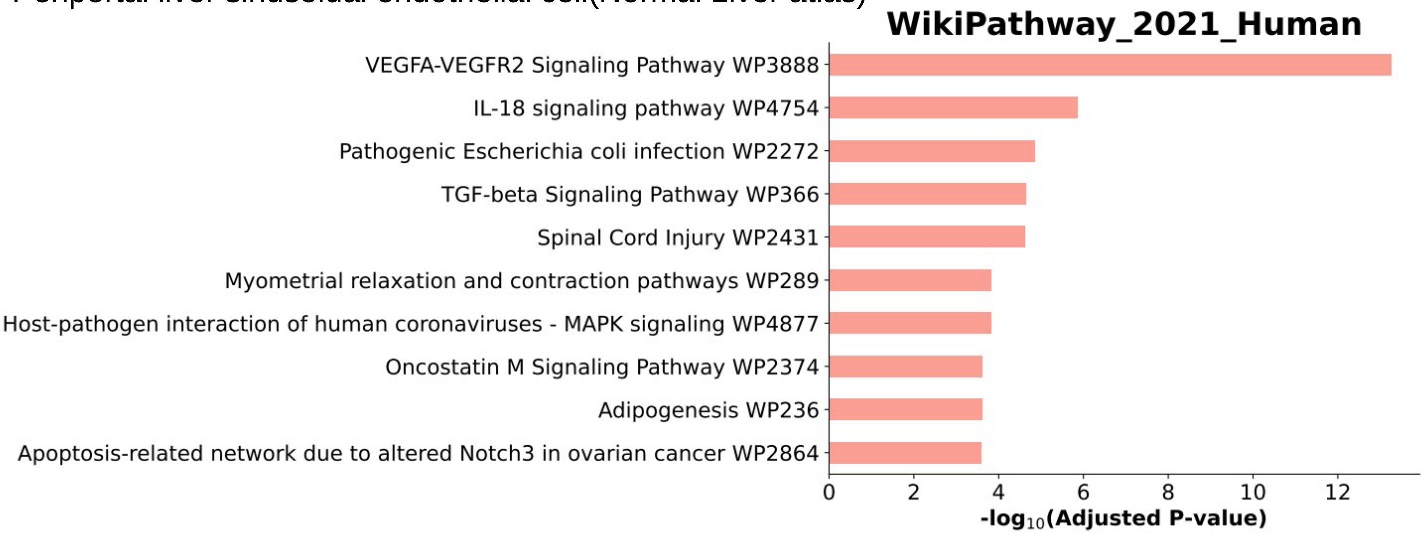

Plasma cell (Normal Liver atlas)

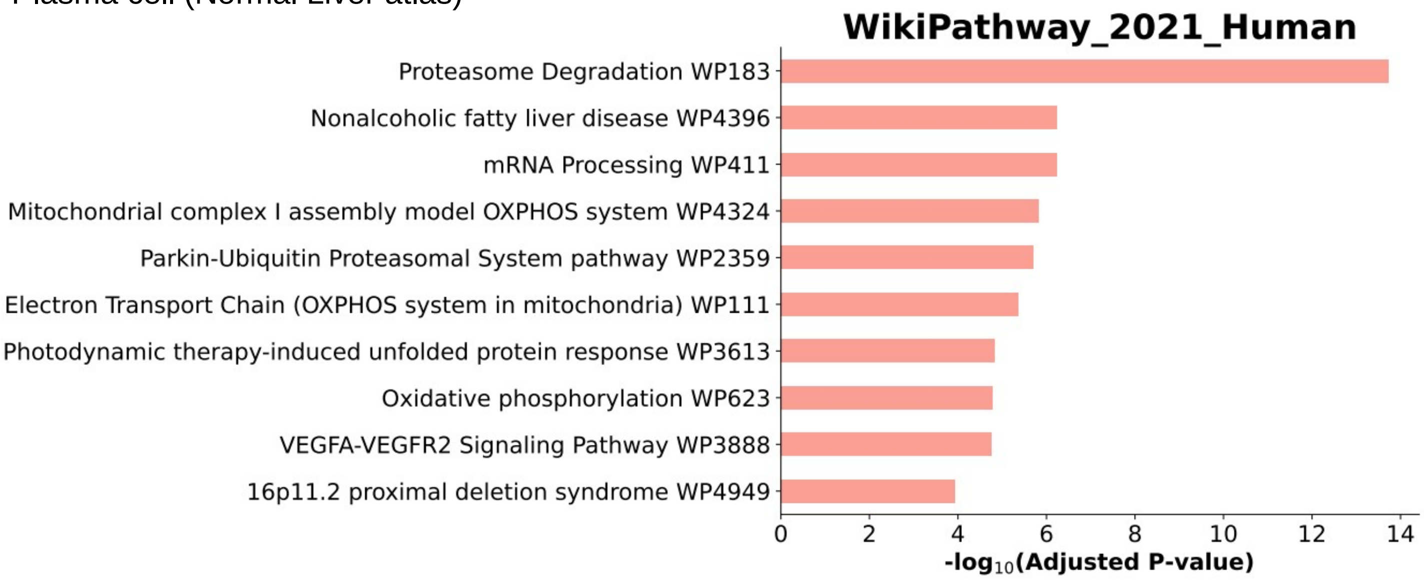

Portal endothelial cell (Normal Liver atlas)

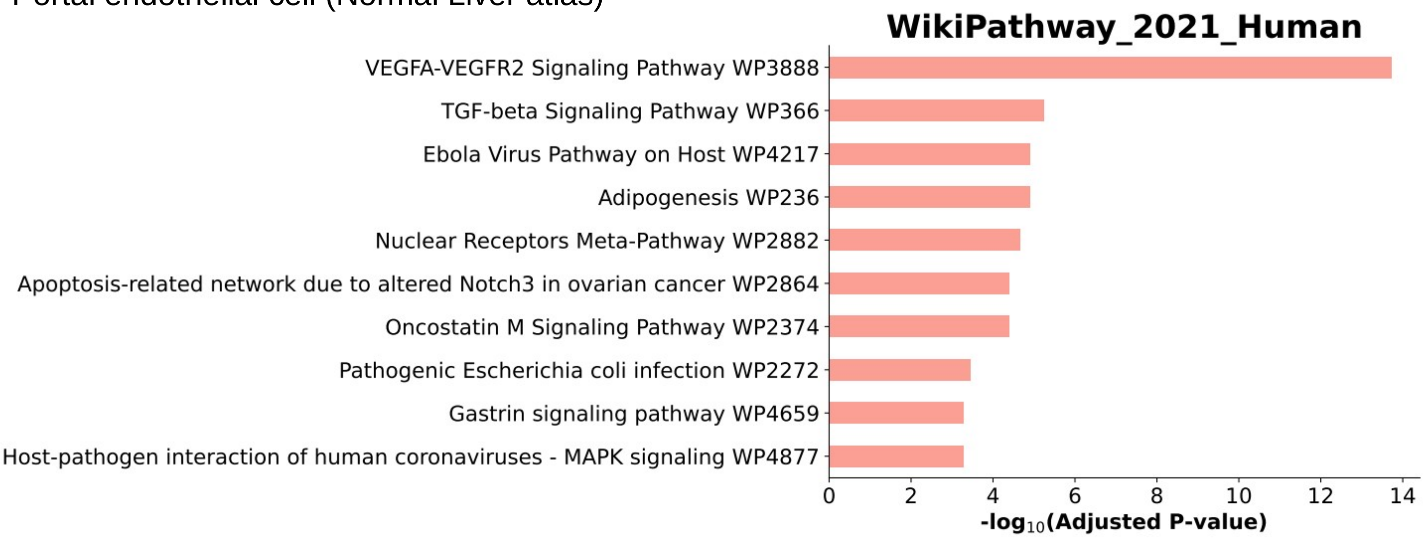

B cell (TME-Stroma atlas)

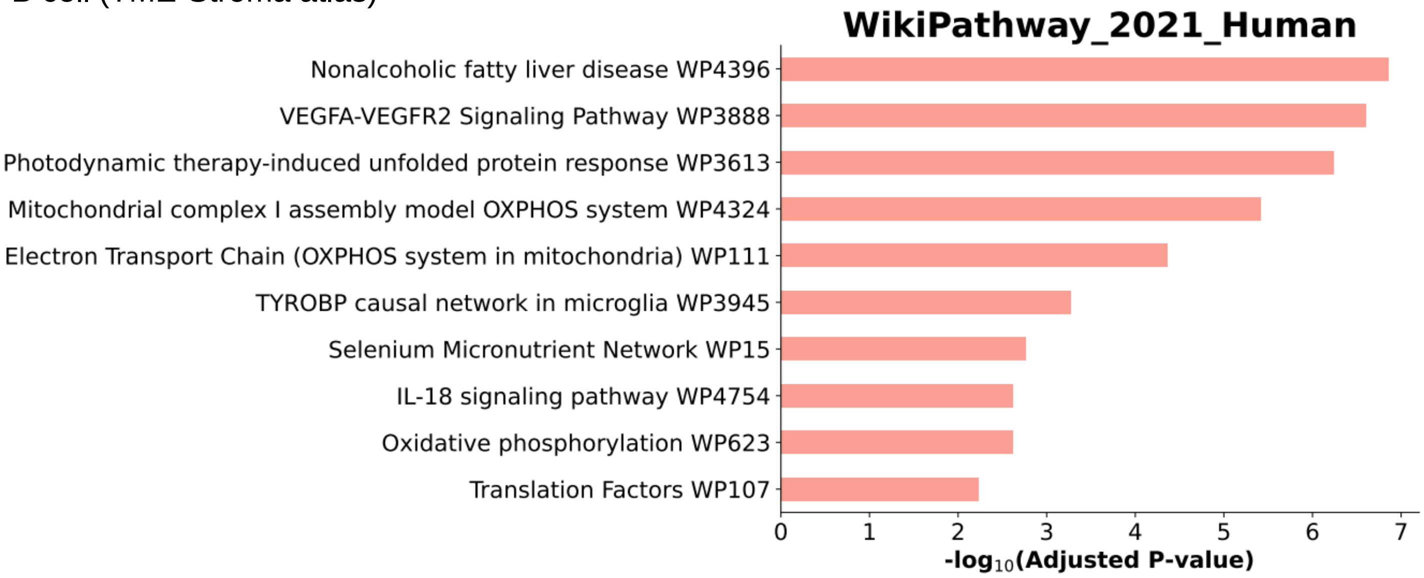

Cancer-associated fibroblast (TME-Stroma atlas)

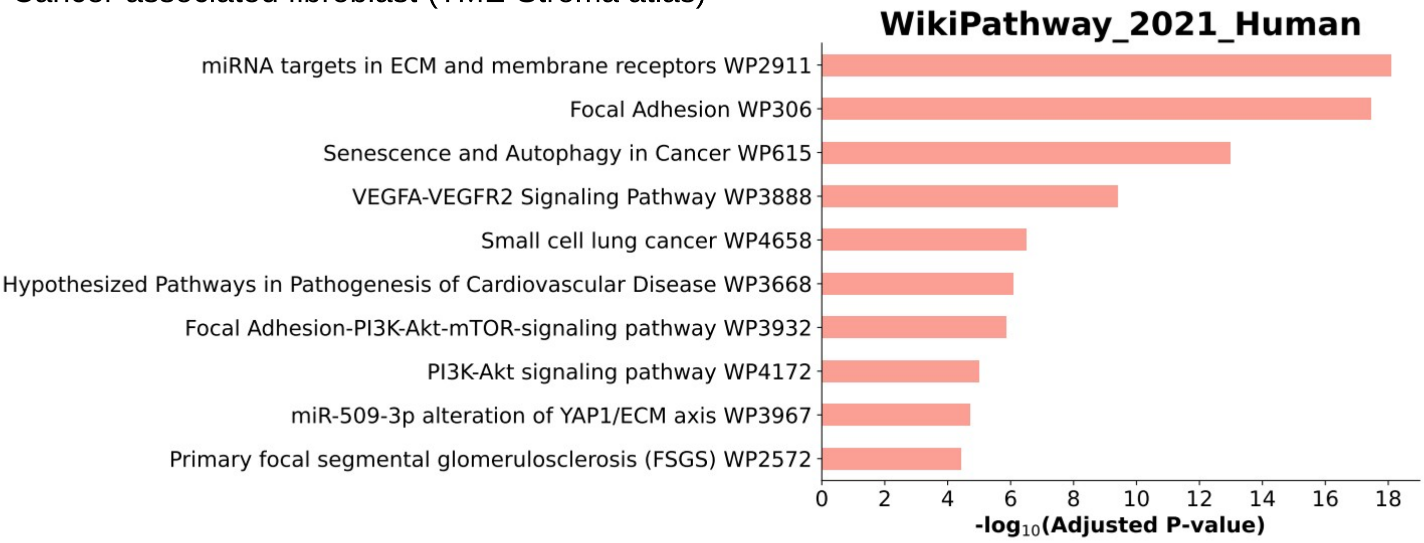

Convential dendritic cell 1 (TME-Stroma atlas)

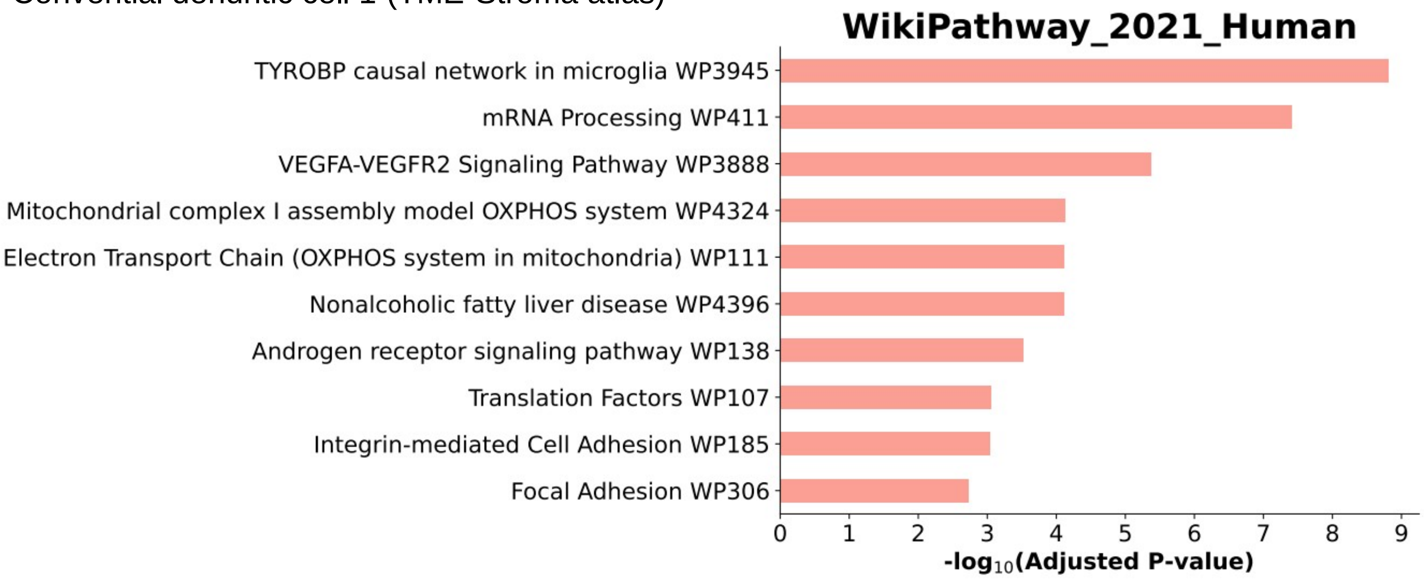

Conventional dendritic cell 2 (TME-Stroma atlas)

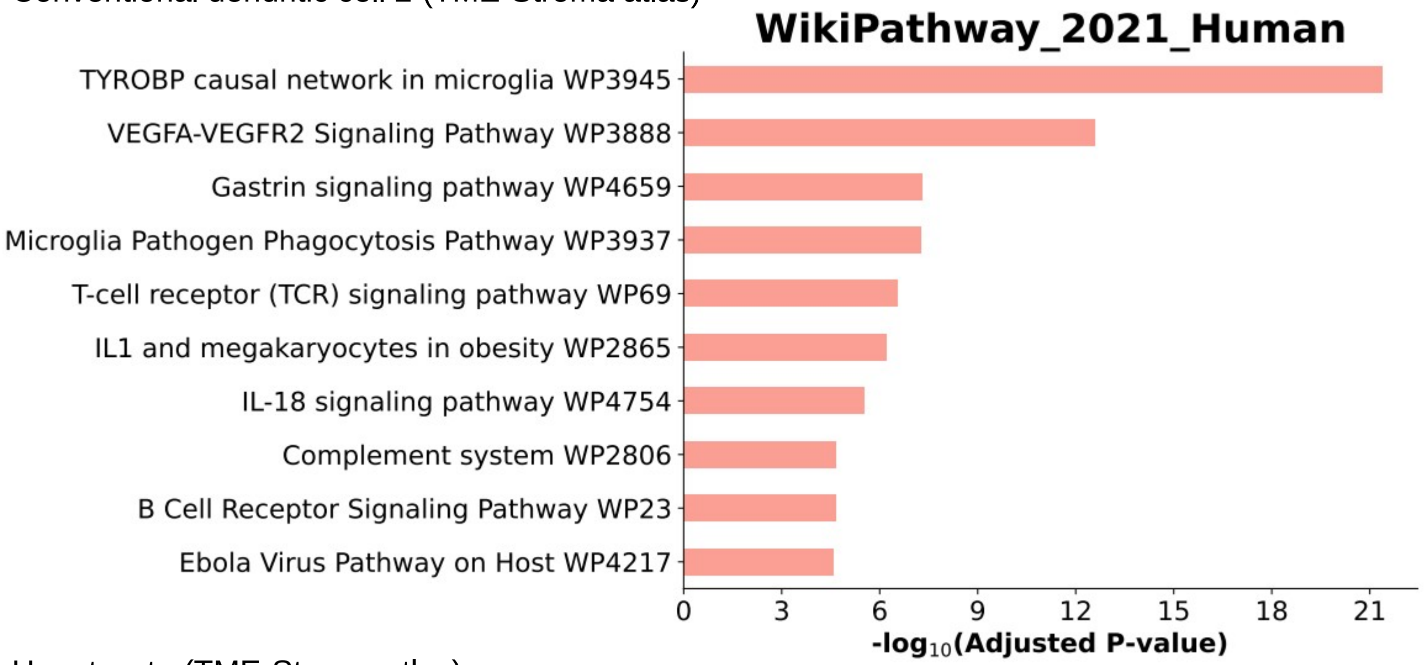

Hepatocyte (TME-Stroma atlas)

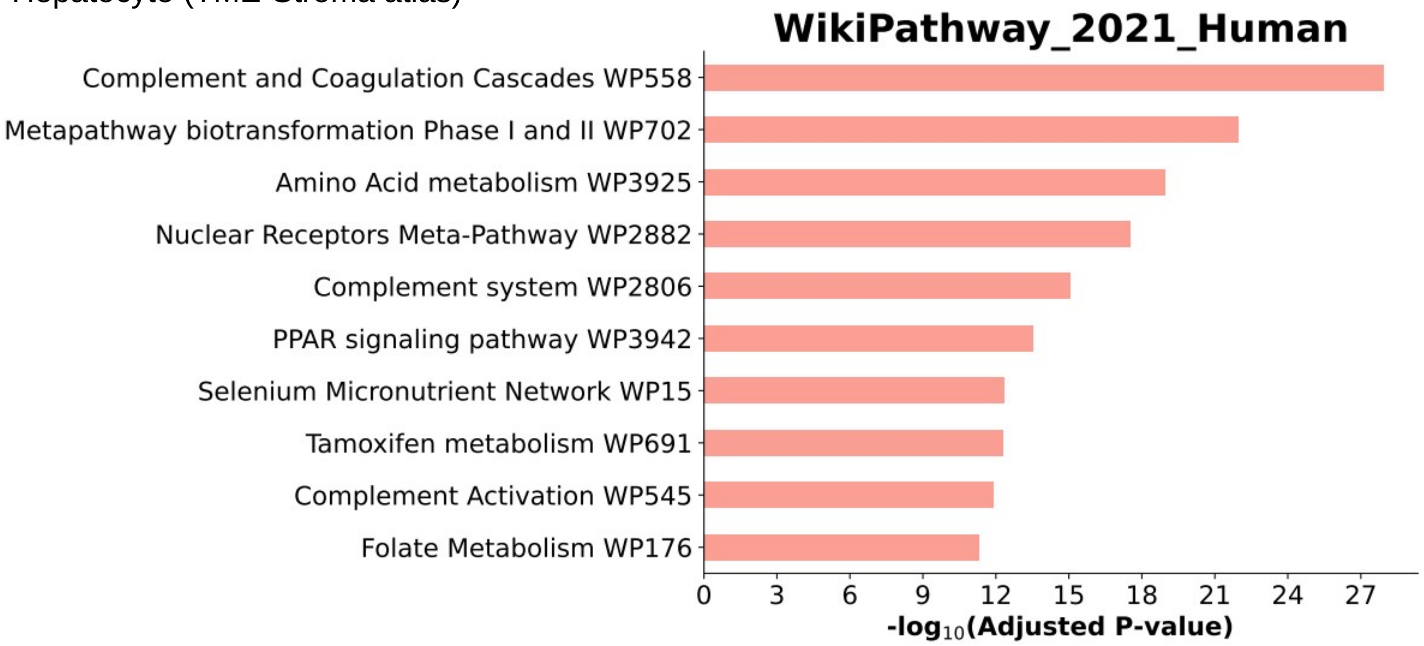

Kupffer cell (TME-Stroma atlas)

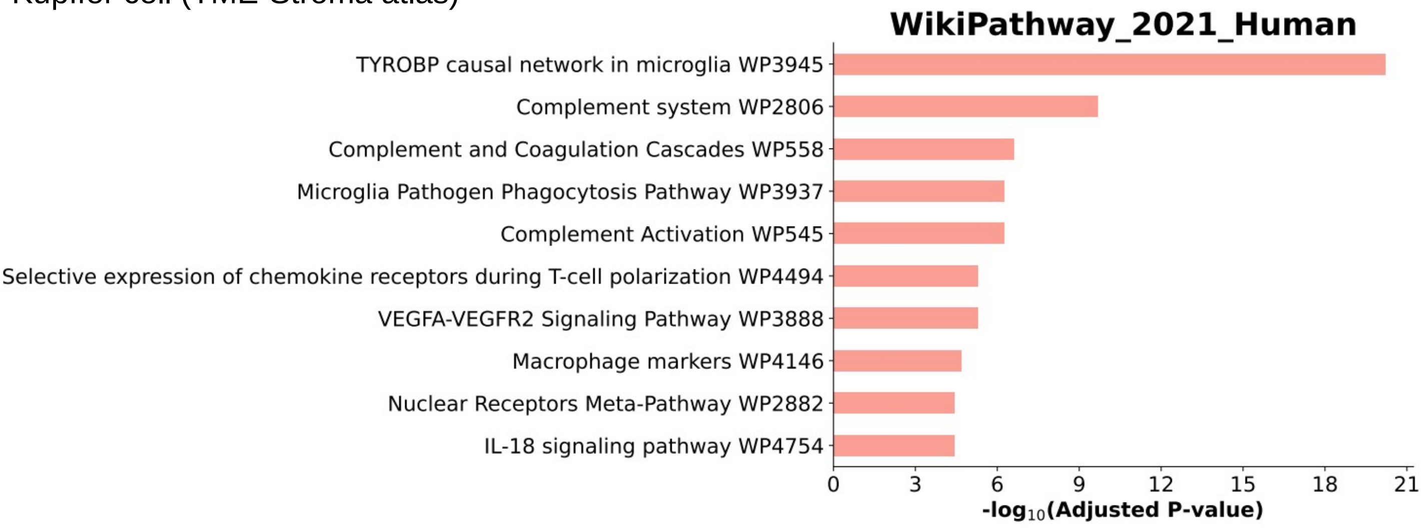

Liver sinusoidal endothelial cell (TME-Stroma atlas)

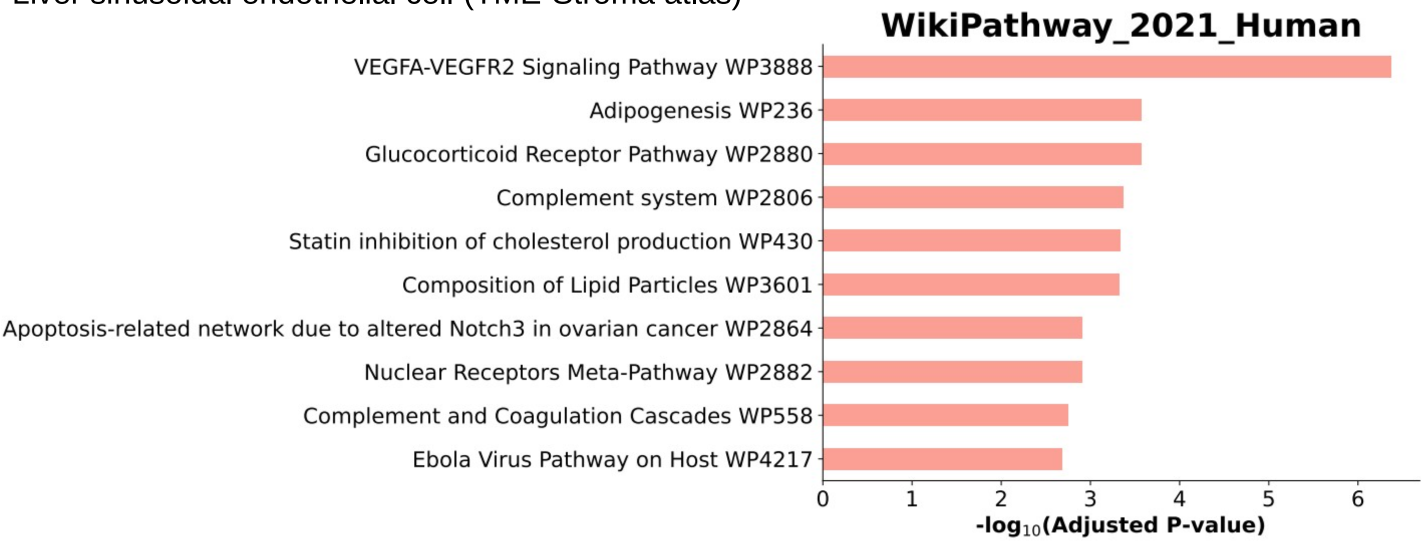

Liver vascular endothelial cell (TME-Stroma atlas)

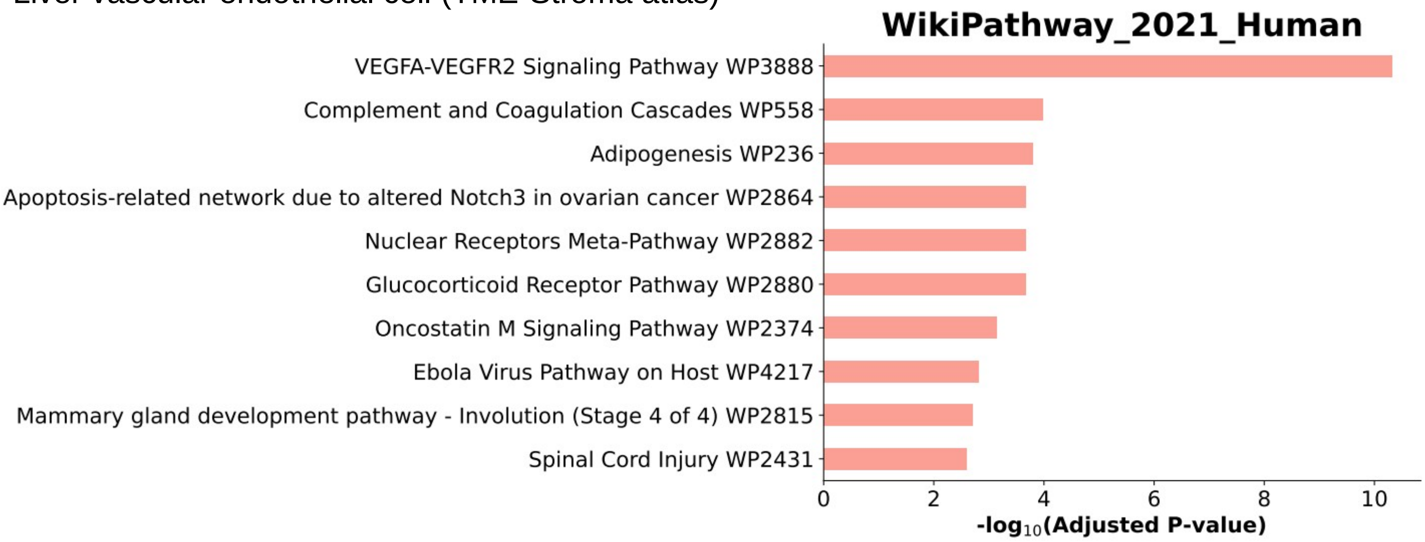

Tumour liver vascular endothelial cell (TME-Stroma atlas)

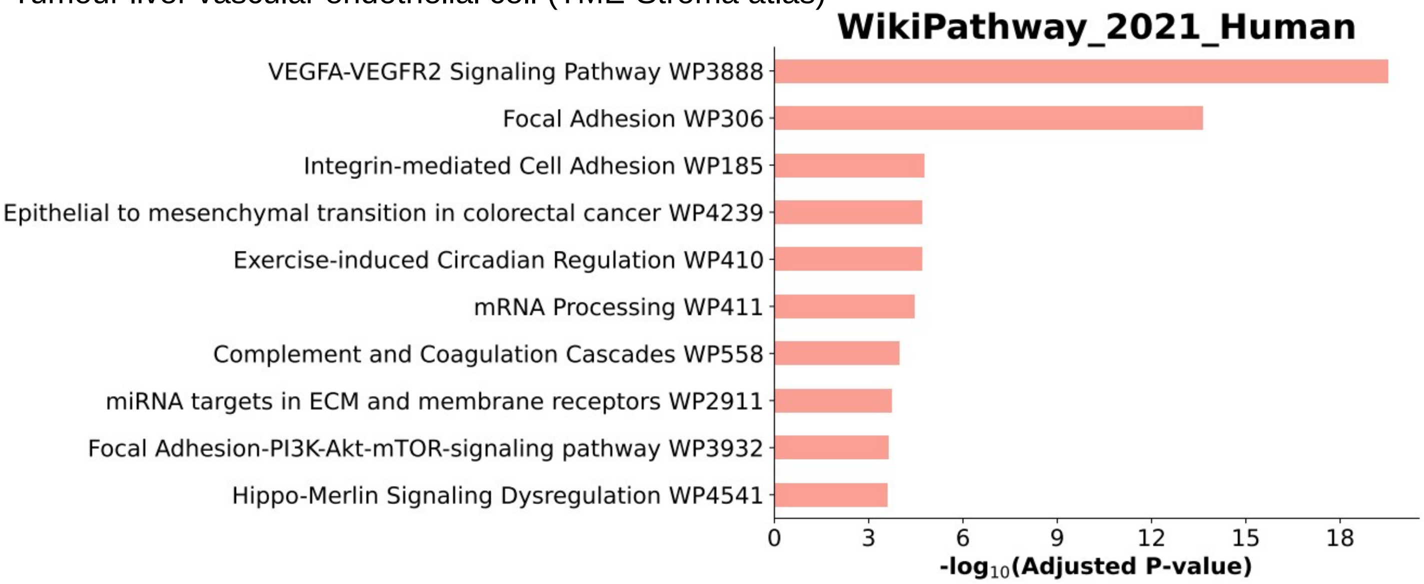

Pericyte (TME-Stroma atlas)

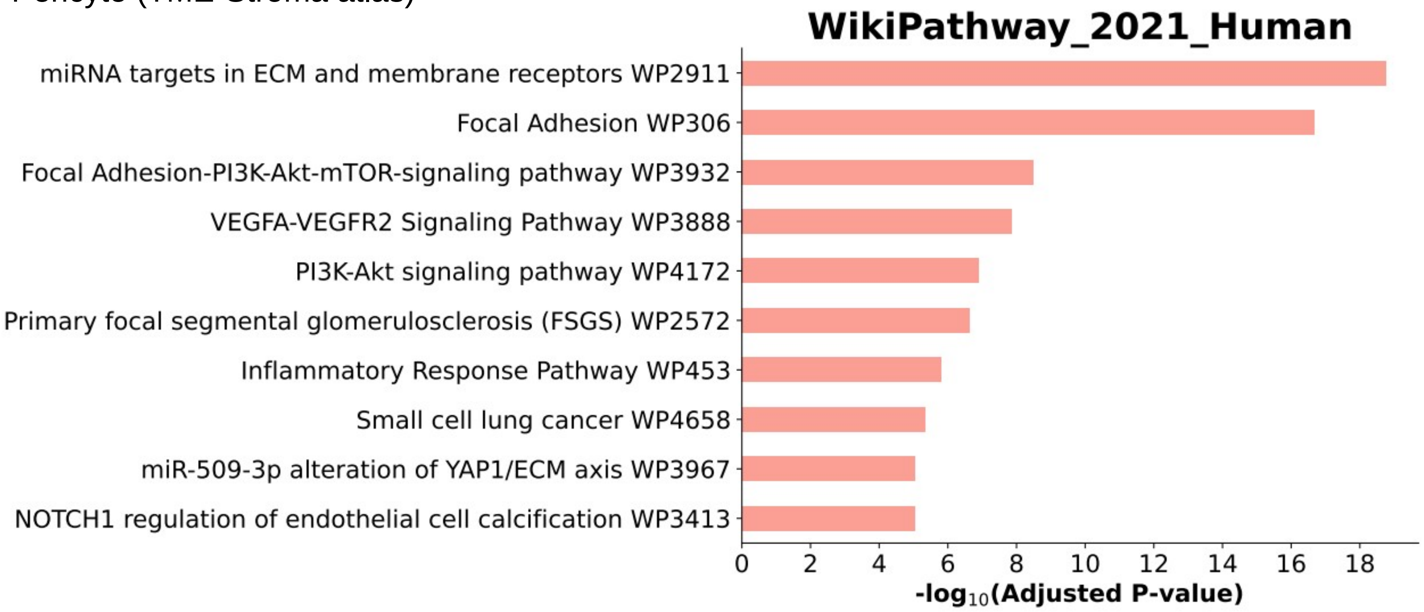

Proliferation (TME-Stroma atlas)

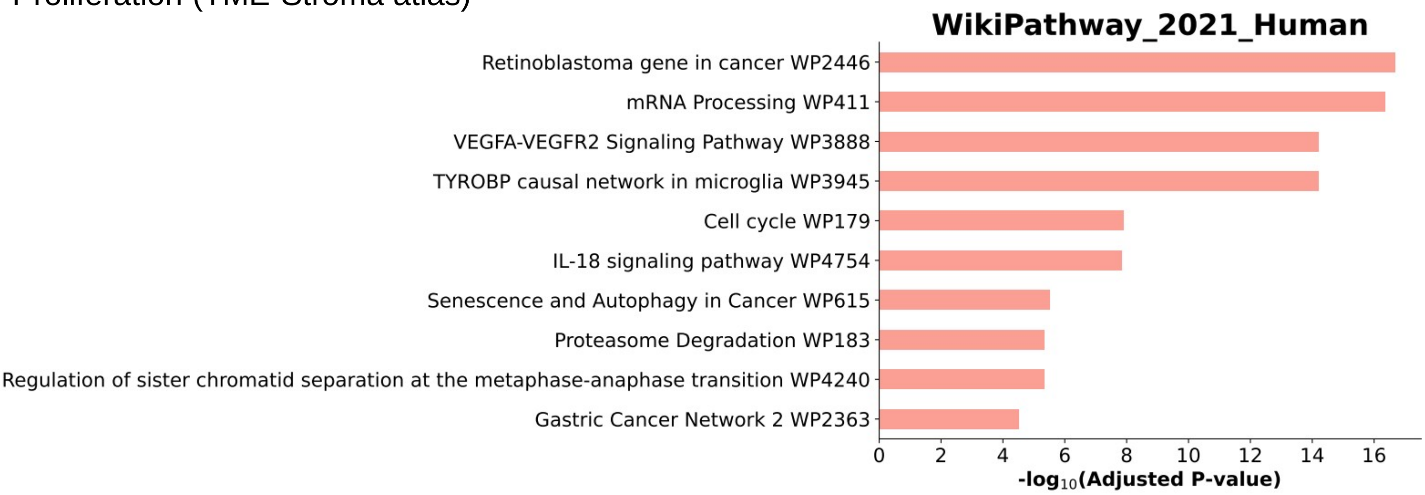

Scar-associated macrophage (TME-Stroma atlas)

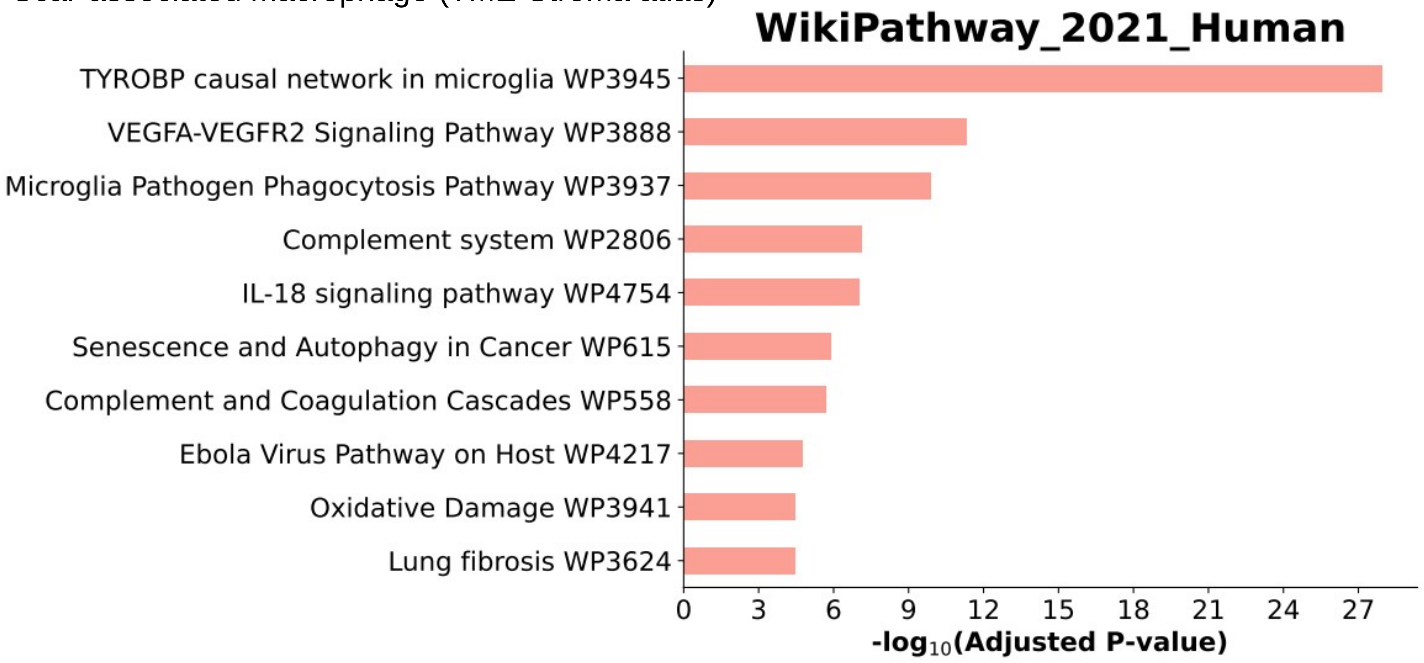

Stellate cell (TME-Stroma atlas)

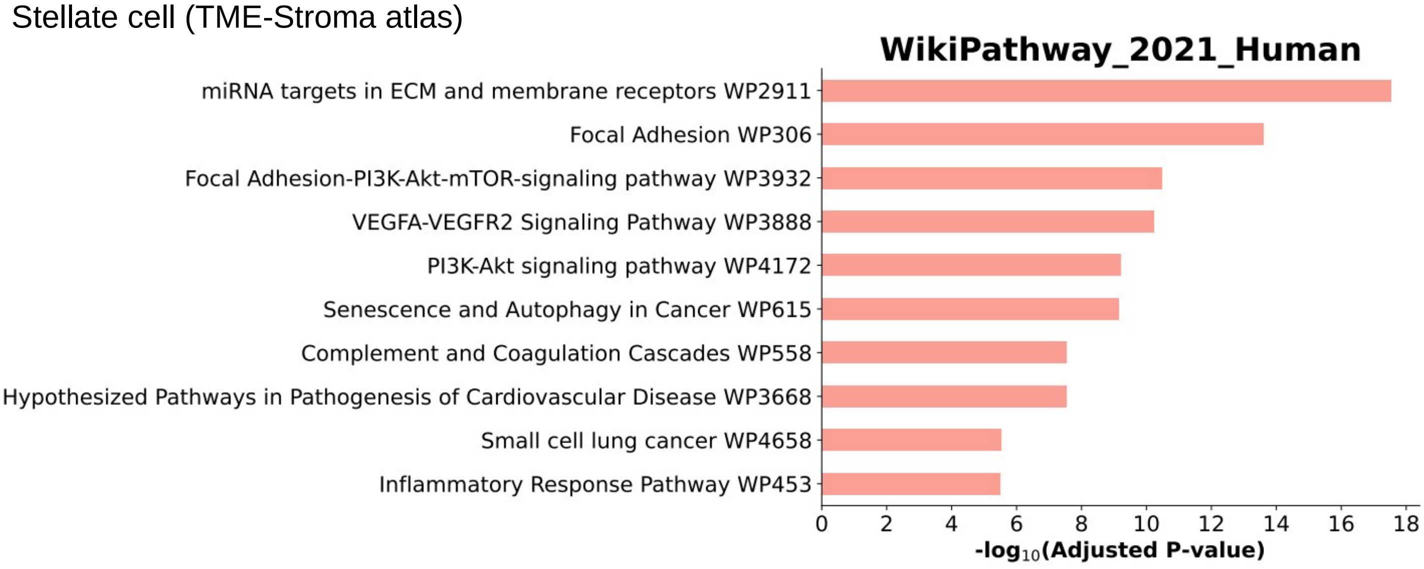

T cell (TME-Stroma atlas)

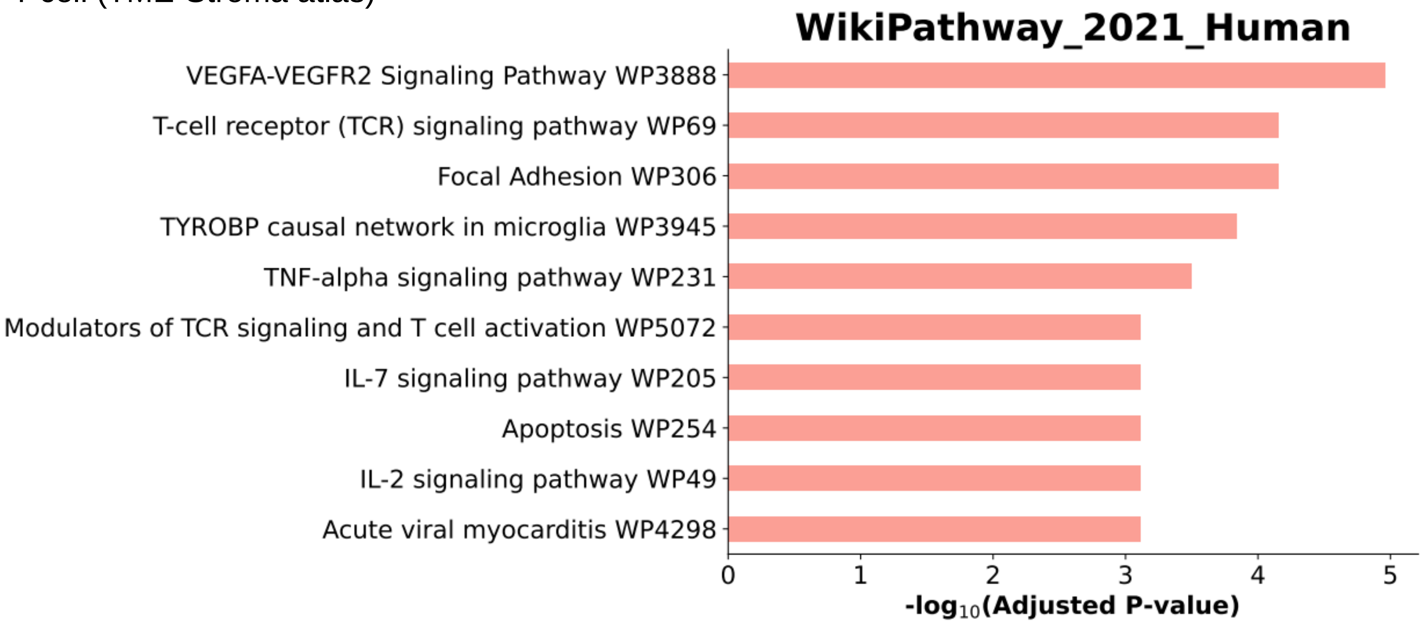

Tissue macrophage (TME-Stroma atlas)

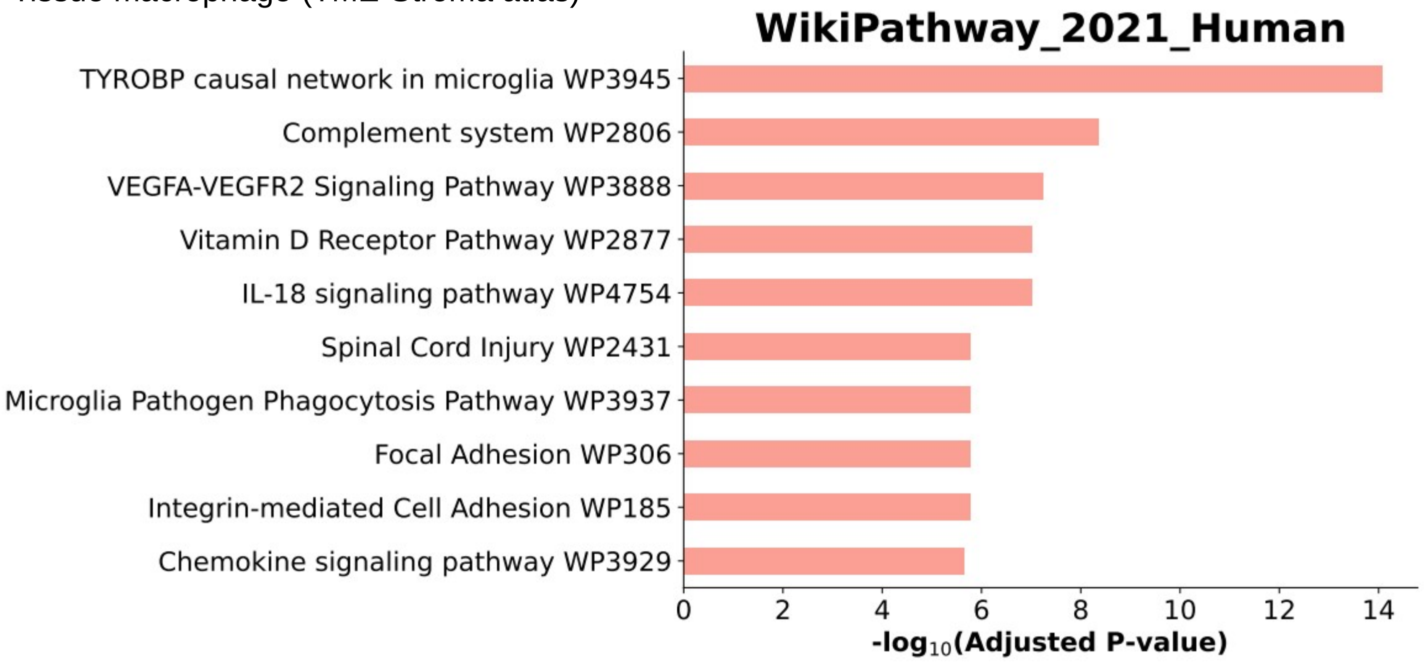

Vascular smooth muscle cell (TME-Stroma atlas)

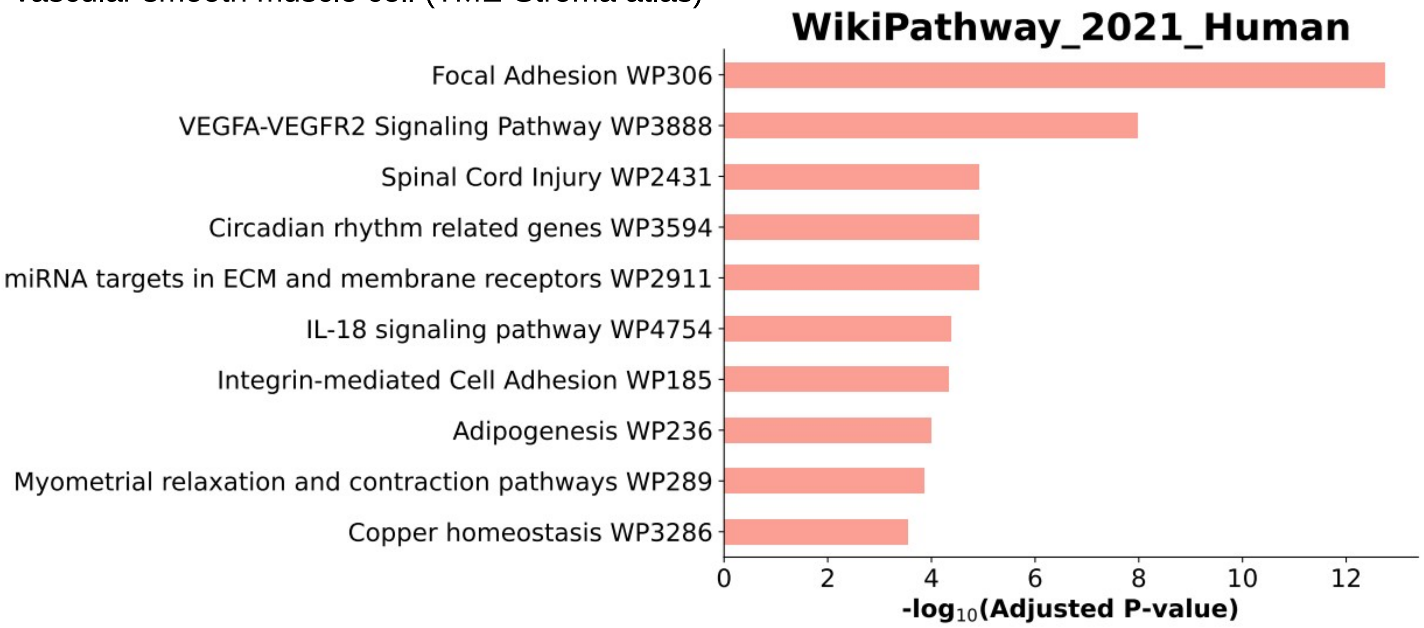

B cell (TME-Immune atlas)

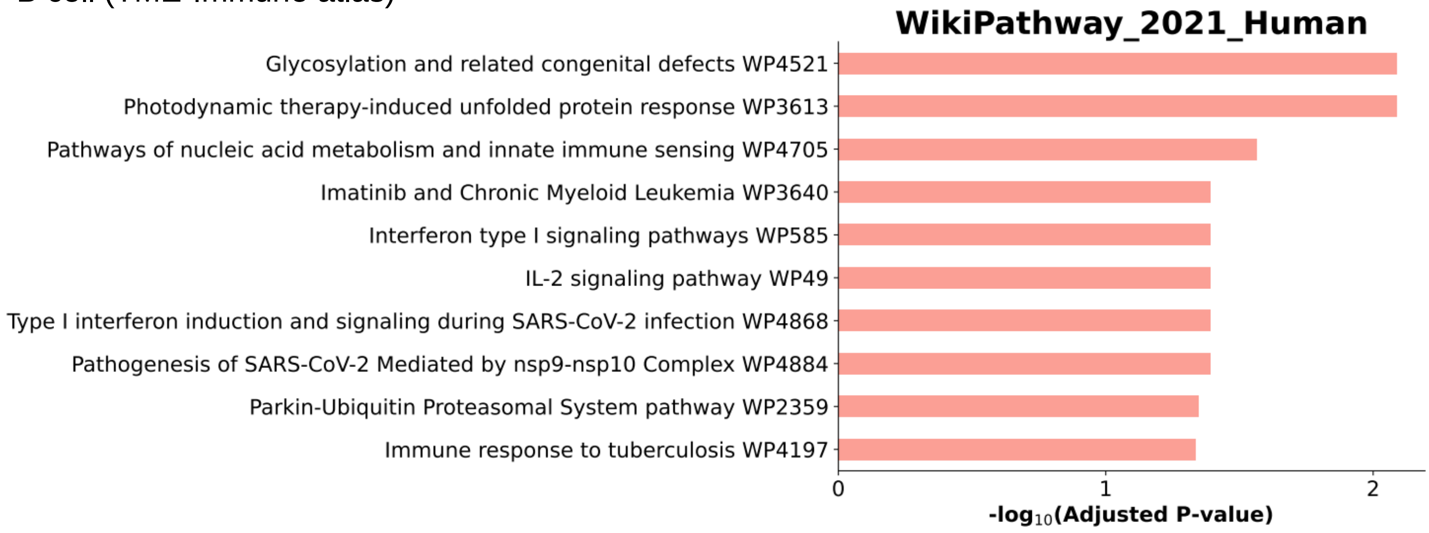

Bi-potent cell (TME-Immune atlas)

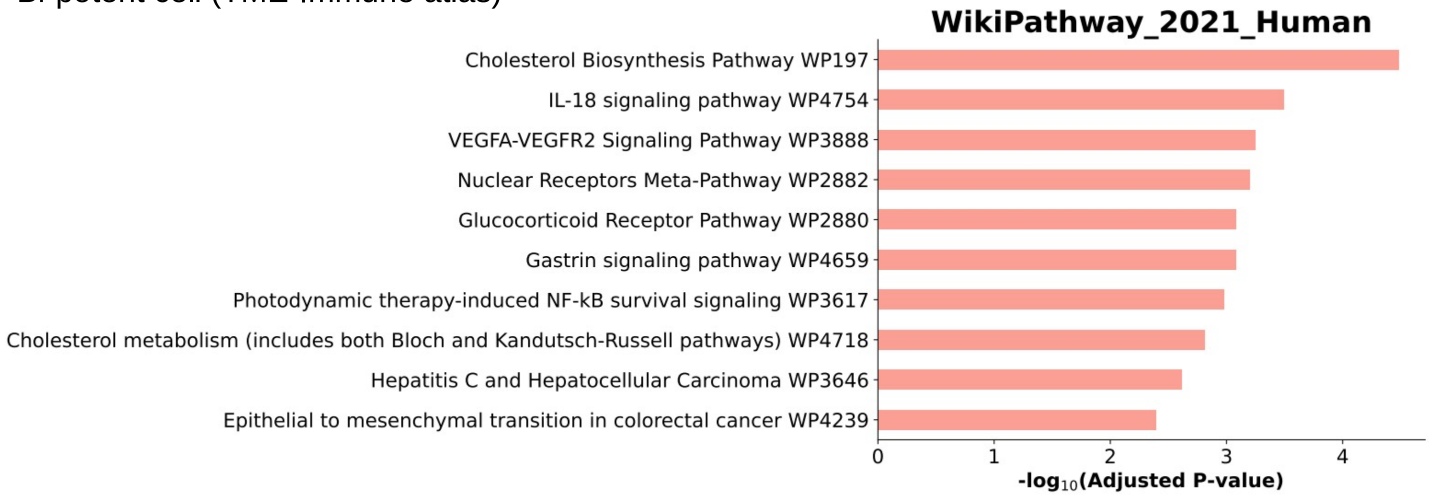

CD4 positive T cell (TME-Immune atlas)

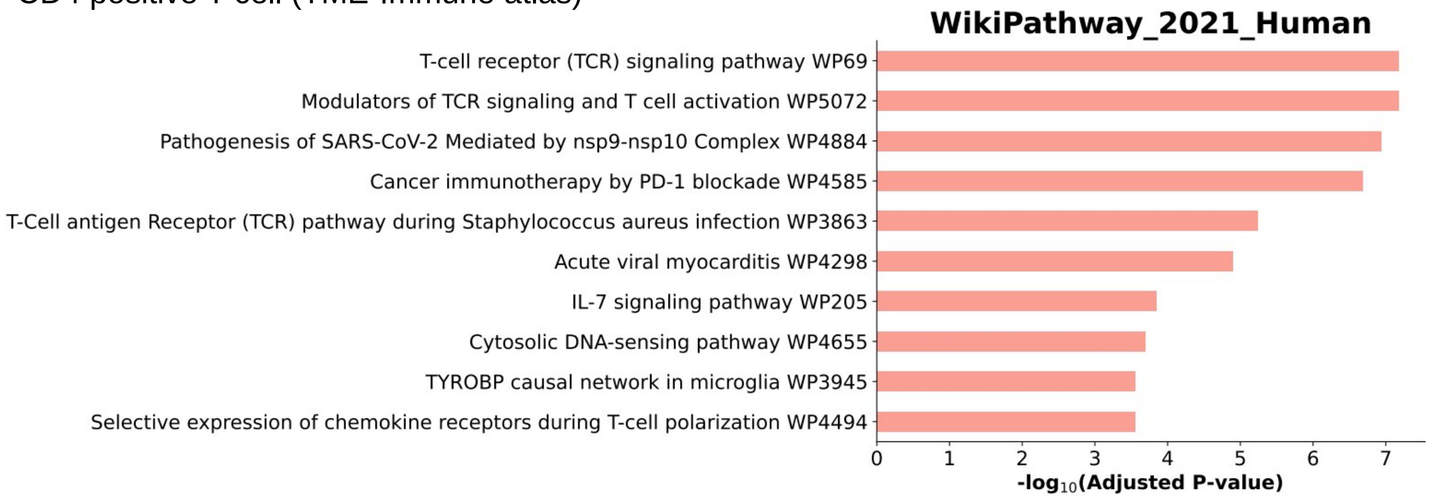

CD8 positive T cell (TME-Immune atlas)

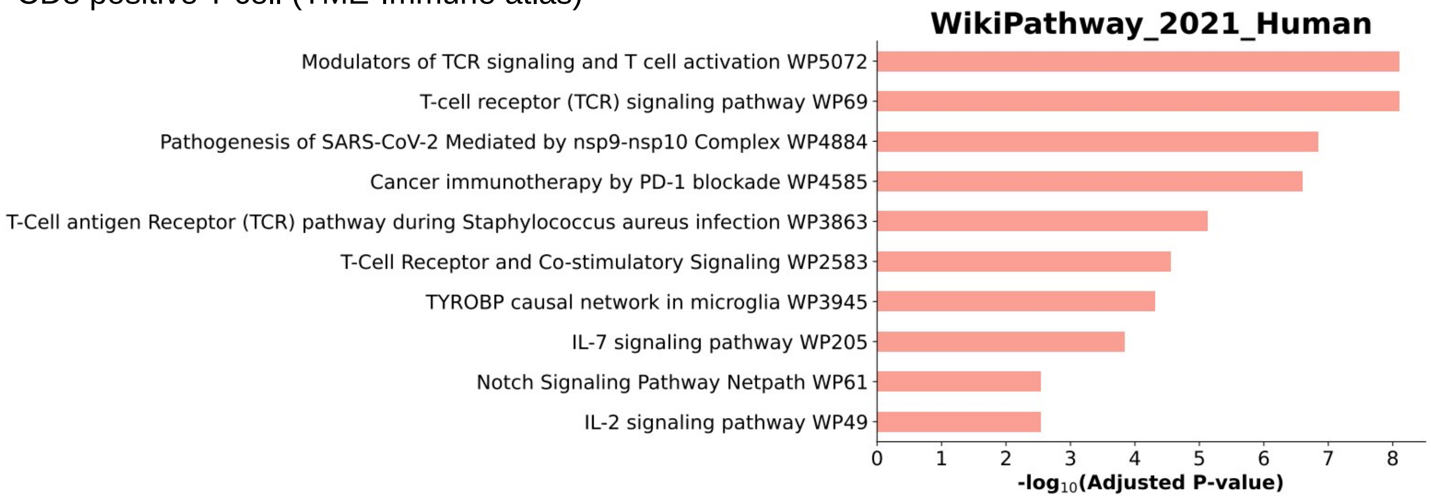

Endothelial cell (TME-Immune atlas)

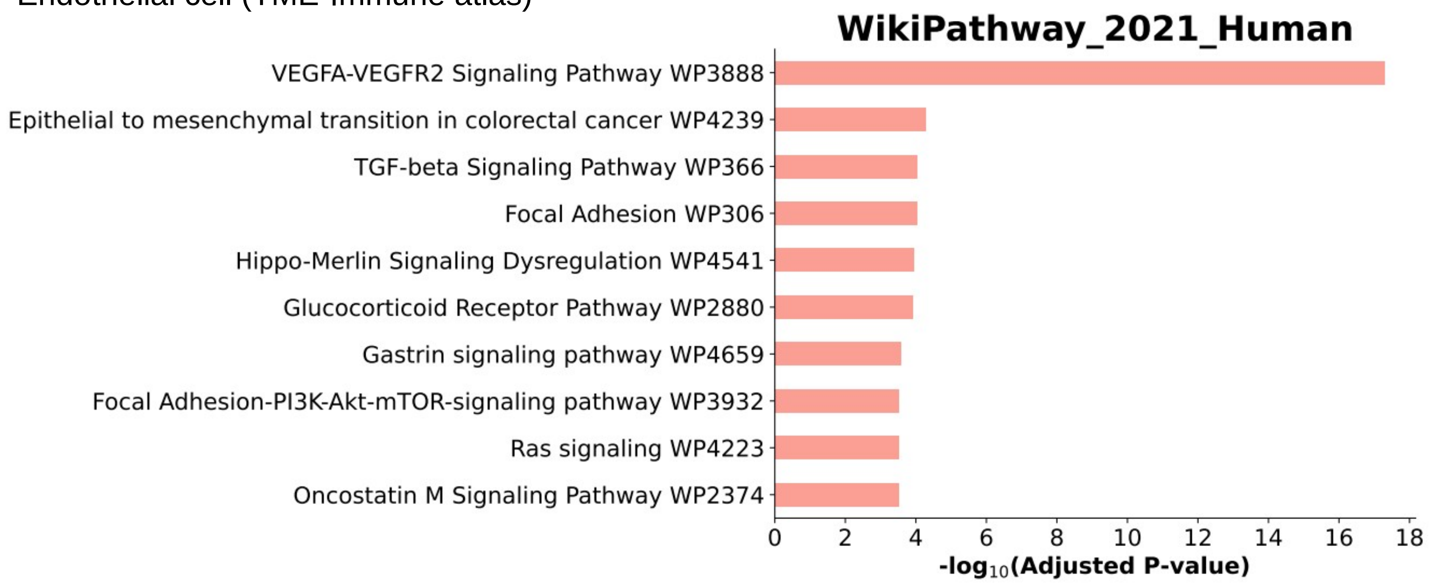

Fibroblast (TME-Immune atlas)

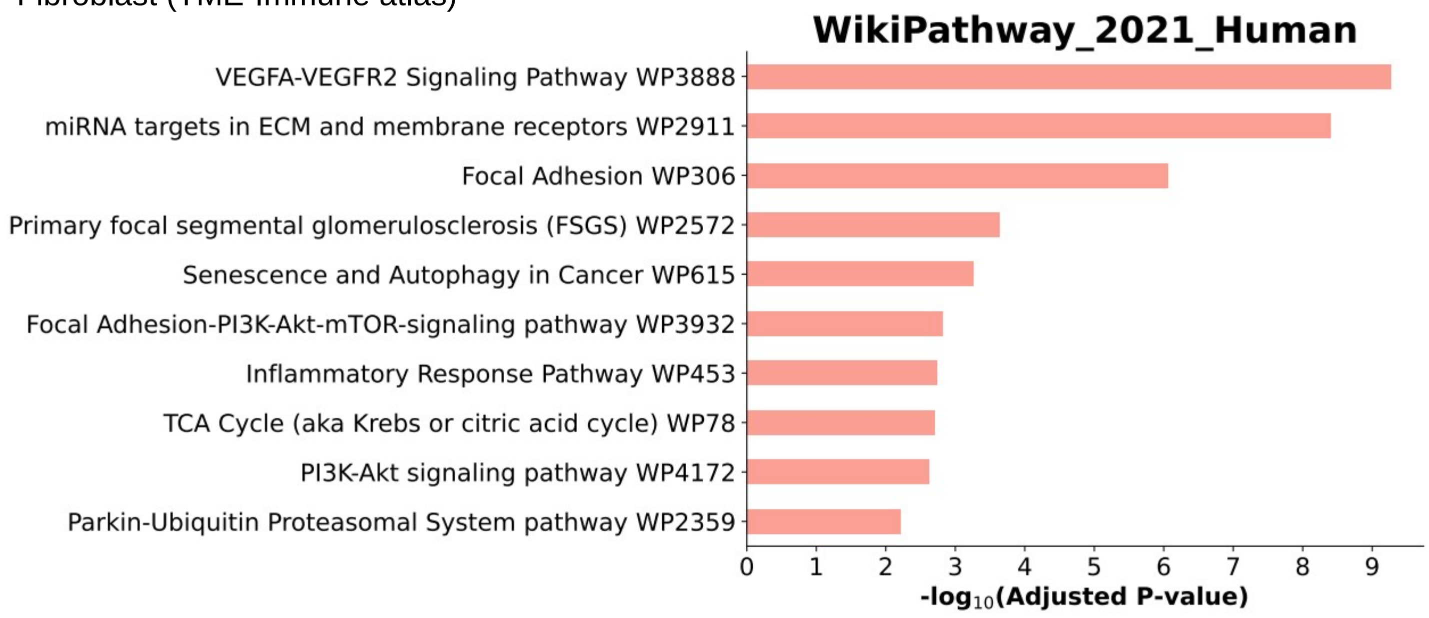

Hepatocyte (TME-Immune atlas)

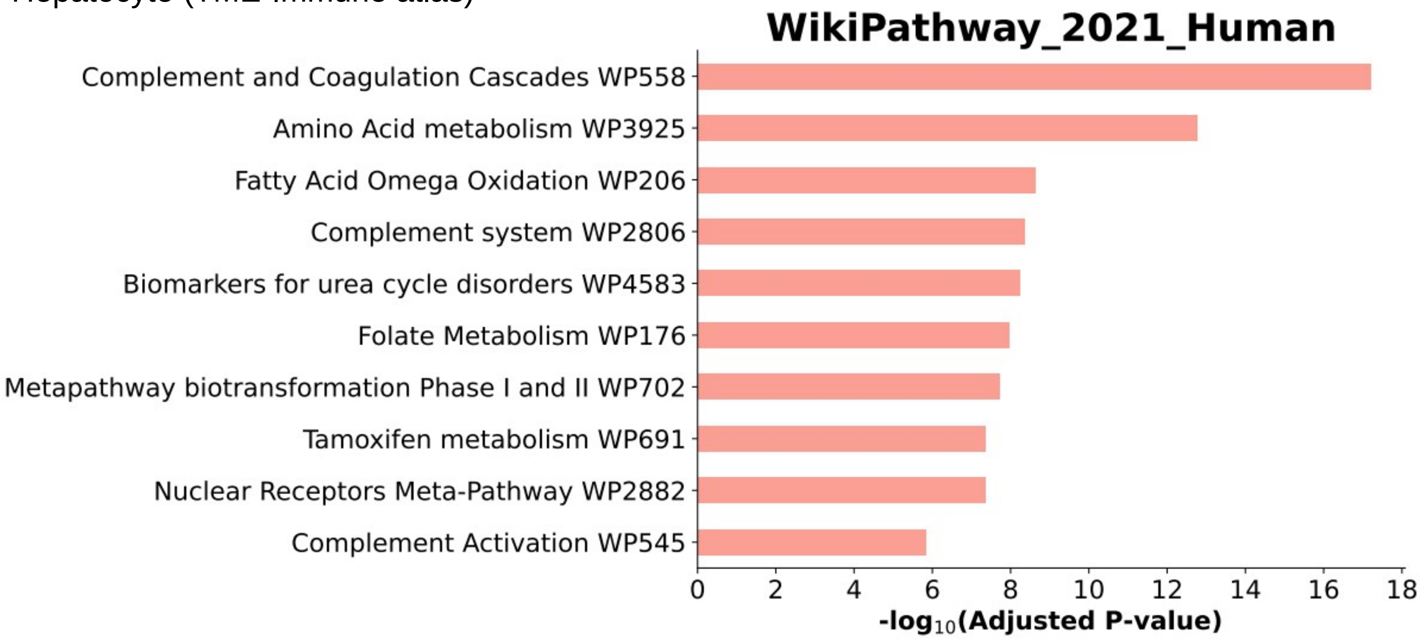

Mast cell (TME-Immune atlas)

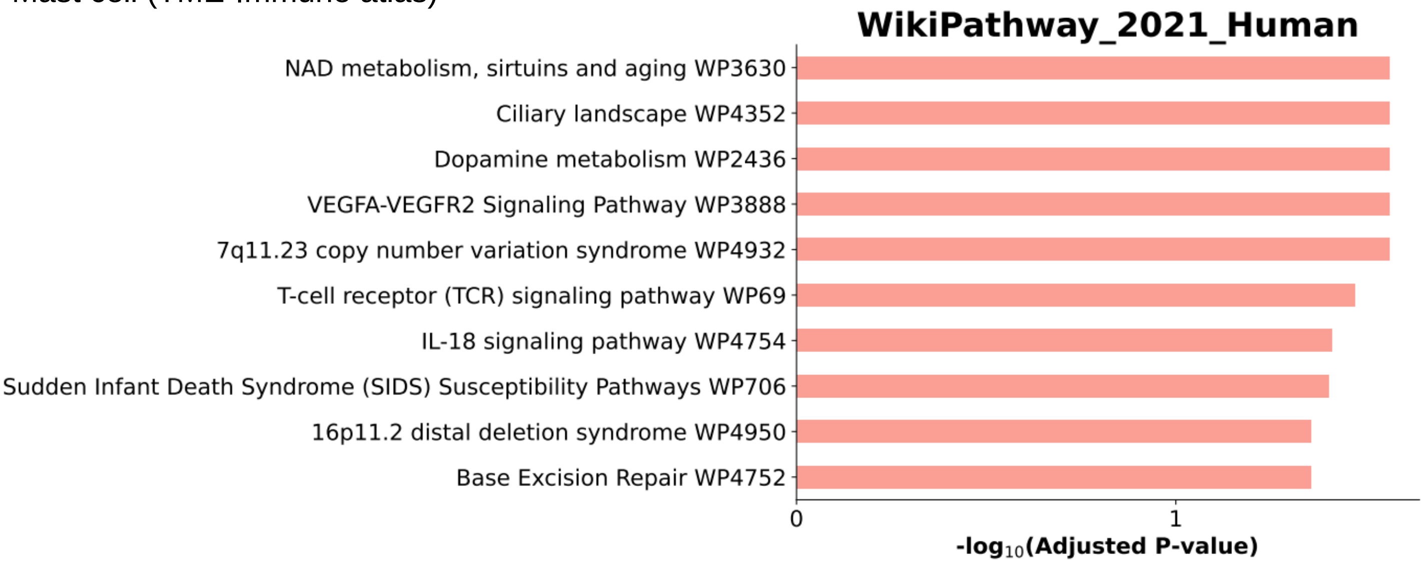

Myeloid cell (TME-Immune atlas)

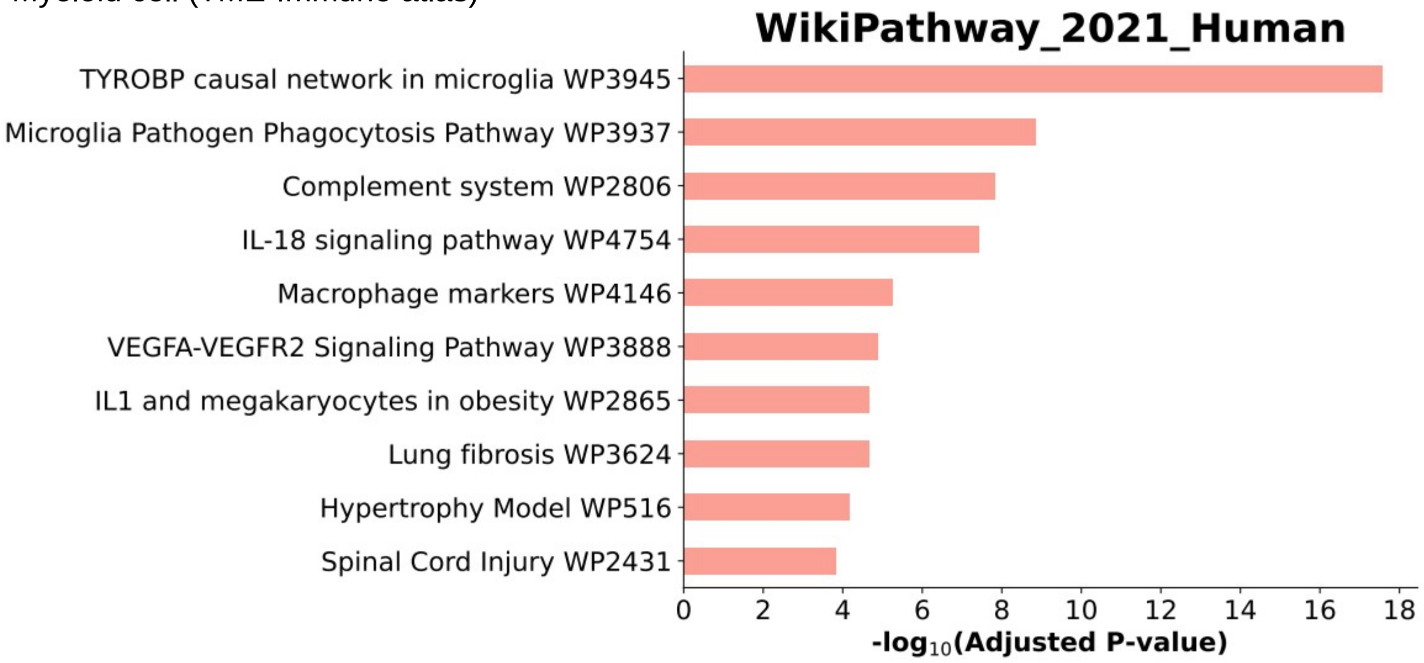

NK cell (TME-Immune atlas)

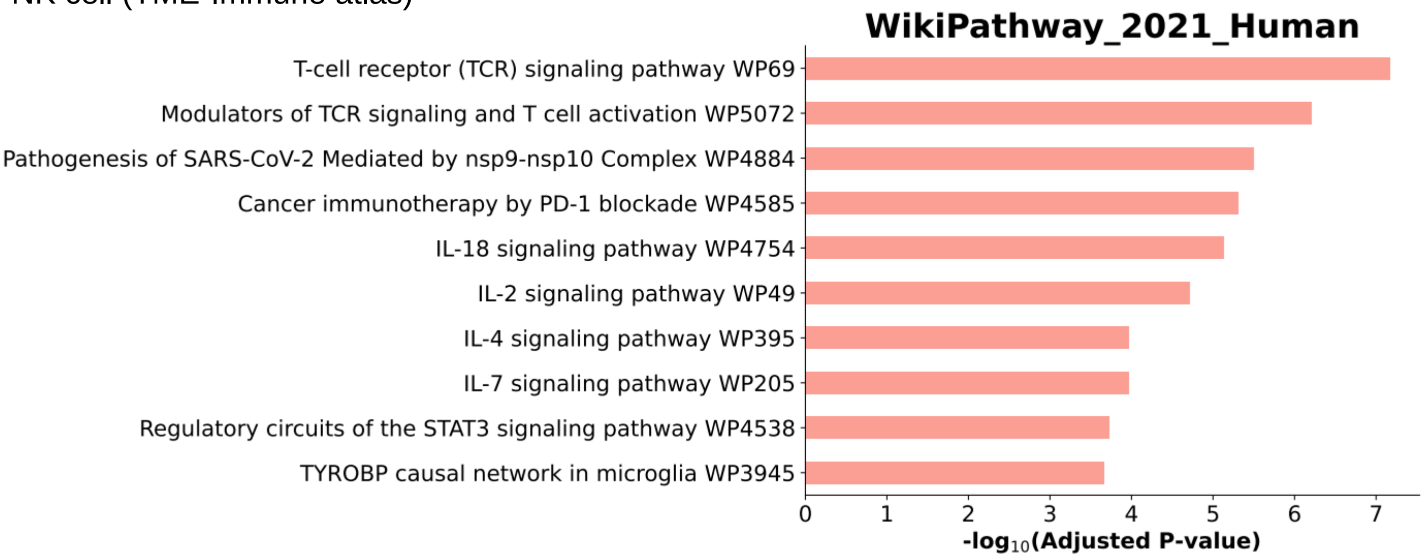

Regulatory T cell (TME-Immune atlas)

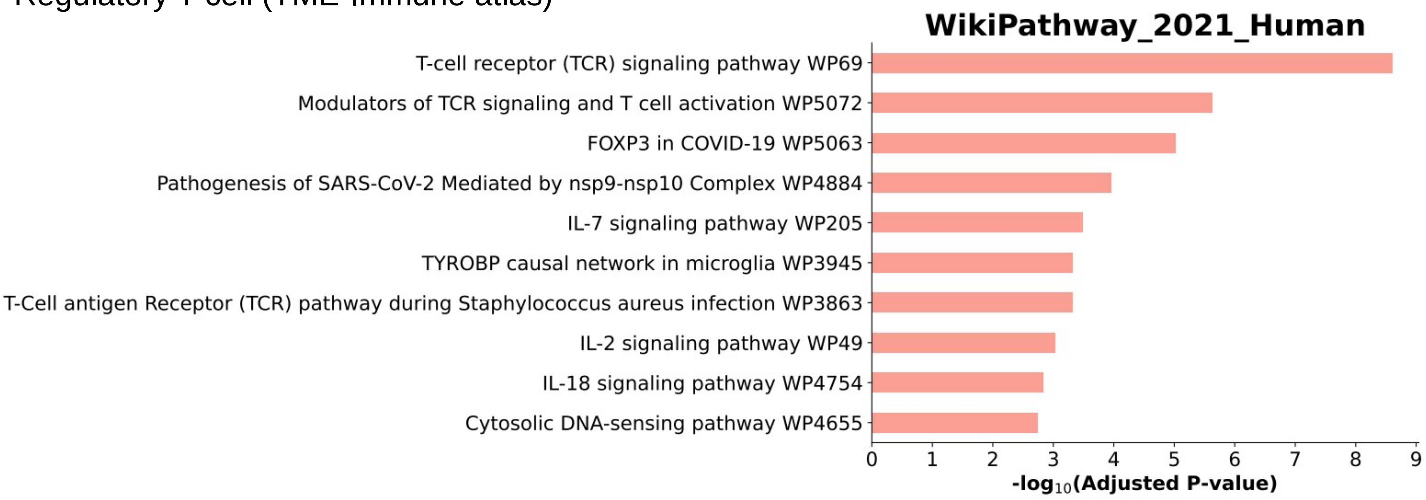

Supplement: Supplementary file 1 [file cancers-15-00153-s001.zip › cancers-2059594-supplementary/Supplements/S7_Pathway_Analysis.pdf]
