# Supplementary material for: Deciphering Tumour Microenvironment of Liver Cancer through Deconvolution of Bulk RNA-Seq Data with Single-Cell Atlas
Source: Cancers (Basel). 2022 Dec 27;15(1):153. doi: 10.3390/cancers15010153 (PMC9818189; doi:10.3390/cancers15010153)
Supplement: Supplementary file 1 [file cancers-15-00153-s001.zip › cancers-2059594-supplementary/Supplements/Supporting_Information .pdf]

Supporting Information

Estimation of Cell Abundance through Support Vector Regression

We first tested the performance of ε-support vector regression(ε-SVR). E-SVR was constructed with the Scikit-Learn toolkit and trained with pseudobulk datasets. Pseudobulk generation was in the same principle in the main text. Training data, testing data, and expression matrix for prediction were merged and normalised by the Trimmed Mean of M-values (TMM) method with library sizes as 1×10^6 [1,2]. Then, features were filtered by marker genes. Finally, features were divided by the max value of each gene and re-scaled into values between 0 and 1.

In the test of hyperparameters, three parameters of ε-SVR were tuned by in silico experiments. Among the four available kernel functions (linear, polynomial, radial basis function and sigmoid), the radial basis function performs the best. The experiments were with a gradient (e.g., 1,0.9,0.8,0.5) of hyperparameter c (regularisation). We did not observe any improvement by adjusting hyperparameter c. Then c=1 was used in subsequent calculations. After testing a gradient (0, 0.02, 0.05, 0.1, 0.2) of hyperparameter ε (penalty), we found the model performs best with 0, thus guiding the subsequent experiments.

Feature selection based on marker gene calculation by Seurat and optimisation experiments. Differential gene expression (DGE) for each cell type was first calculated by Seurat [3]. The best feature number varies between cell types. Seurat returned two parameters to evaluate marker genes – average logarithm of fold change (avg_log2fc) and percentage of occurrence (pct). We defined the pct ratio as pct.1/pct.2. Through tests with a gradient (1, 1.5, 2, 3, 5, 10, 20, 30, 50) of pct ratios, we selected the cutting point with which ε-SVR showed the best performance.

Using the ε-SVR model with the best configuration, we predicted cell abundance for liver cancer samples. Pseudobulk was generated with scRNA-seq atlases and used as training data for ε-SVR. The training data and expression matrices of bulk RNA-seq were merged and normalised with the TMM method. Then the features were filtered with optimised marker genes and rescaled into values between 0 and 1. The visualisation of results is identical to those in the main text.

Comparison of Results by Cibersortx and Support Vector Regression

The estimated results by Cibersortx and ε-SVR achieved the same conclusions for most cell types with some exceptions.

Normal Atlas, Hepatocytes, consistent.

TME-Immune Atlas, Hepatocytes, consistent.

Normal Atlas, Cholangiocytes, consistent.

TME-Stroma Atlas, HSCs, consistent with some marginal exceptions.

TME-Stroma Atlas, Pericytes, consistent.

TME-Stroma Atlas, CAFs, consistent.

TME-Stroma Atlas, LSECs, consistent.

TME-Stroma Atlas, vSMCs, inconsistent. Cibersortx predicted significantly lower fractions in tumours but no conclusion from SVR estimation.

TME-Stroma Atlas, LVECs, consistent.

TME-Stroma Atlas, LVECts, consistent with some marginal exceptions.

Normal Atlas, alpha-beta T cells, consistent with some marginal exceptions.

Normal Atlas, gamma-delta T cells, inconsistent. No conclusion from Cibersortx prediction but SVR predicted significantly higher fractions in tumours.

TME-Immune Atlas, CD4+ cells, partially consistent. Both predicted significantly higher fractions in CCA and broad marginal elevations in HCC. Inconsistency exists in two datasets.

TME-Immune Atlas, CD8+ cells, inconsistent. Cibersortx predicted broad moderate elevations but no conclusion can be drawn from SVR estimation.

TME-Immune Atlas, Regulatory T cells, consistent with some marginal exceptions.

Normal Atlas, Mature B cells. No conclusion from both predictions.

Normal Atlas, Plasma cells, consistent.

Normal Atlas, Inflammatory macrophages, consistent.

Normal Atlas, Non-inflammatory macrophages, inconsistent. No conclusion can be drawn from Cibersortx estimation but SVR predicted significance higher fractions in tumours.

TME-Stroma Atlas, Kupffer cells, partially consistent. Both predicted significantly lower fractions in tumours. Inconsistency exists in three datasets.

TME-Stroma Atlas, SAMs, partially consistent. Both predicted significantly higher fractions in tumours. Inconsistency exists in predictions of two datasets.

TME-Stroma Atlas, cDC1, consistent.

TME-Stroma Atlas, cDC2. No conclusion from both predictions.

TME-Immune Atlas, Bi-potent cells, inconsistent. Cibersortx predicted prominently higher fractions in CCA but SVR predicted broad moderate lower fractions in tumours.

TME-Immune Atlas, NK cells, consistent with some marginal exceptions.

TME-Immune Atlas, Myeloid cells. No conclusion from both predictions.

Reference

1. Robinson, M.D.; Oshlack, A. A Scaling Normalization Method for Differential Expression Analysis of RNA-Seq Data. *Genome Biol.* **2010**, *11*, R25, doi:10.1186/gb-2010-11-3-r25.

2. Avila Cobos, F.; Alquicira-Hernandez, J.; Powell, J.E.; Mestdagh, P.; De Preter, K. Benchmarking of Cell Type Deconvolution Pipelines for Transcriptomics Data. *Nat. Commun.* **2020**, *11*, 5650, doi:10.1038/s41467-020-19015-1.

3. Stuart, T.; Butler, A.; Hoffman, P.; Hafemeister, C.; Papalexi, E.; Mauck, W.M.; Hao, Y.; Stoeckius, M.; Smibert, P.; Satija, R. Comprehensive Integration of Single-Cell Data. *Cell* **2019**, *177*, 1888-1902.e21, doi:10.1016/j.cell.2019.05.031.

Supplmentary Files

S1 – Estimation by Support Vector Regression

Estimation of Cell Abundance by Support Vector Regression

Pages 1 – 5.

Pages 1 – 5 are the counterparts of Figures 2-6 in the main text. Estimation by support vector regression (SVR) achieved similar results for most cell types. Of note, SVR estimates only one cell type while the Cibersorts returns the fractions summed together equal to 1. This caution is warranted when interpreting the difference between results by Cibersortx and SVR.

S2 – Cell Type Hierarchy

Sometimes, atlases with complicated cell-type tree may overdrive Cibersortx. This group of figures show how the cell types were collapsed. Dash lines separate the cell-type tree into branches. When estimating the components in one branch, collapsing other branches may smooth the calculation of Cibersortx.

S3 – Estimation by Cibersortx and Support Vector Regression – Other Cell Types

Pages 1. Cibersortx Estimation – Normal Atlas. Hepatic stellate cells, NK-like cells, portal endothelial cells.

Pages 2. Cibersortx Estimation – TME-Stroma Atlas. Hepatocytes, TM1 cells, T cells, B cells.

Pages 3. Cibersortx Estimation – TME-Immune Atlas. B cells, fibroblasts.

Pages 4. SVR Estimation – Normal Atlas. Hepatic stellate cells, NK-like cells, portal endothelial cells.

Pages 5. SVR Estimation – TME-Stroma Atlas. Hepatocytes, TM1 cells, T cells, B cells.

Pages 6. SVR Estimation – TME-Immune Atlas. B cells, fibroblasts.

S4 – In Silico Validation of Cibersortx and Support Vector Regression

Intra-study Validation of Deconvolution – Normal Atlas

Page 1 – 5.

The table on page 1 summarises this group of experiments.

This group includes intra-study experiments. Cibersortx was trained with the Normal Atlas. Pseudobulk data was also generated from the Normal Atlas. Cell labelling was according to the authors' instructions. The horizontal axis represents the predefined proportion while the vertical axis shows the predicted value by Cibersortx.

Intra-study Validation of Deconvolution – Normal Atlas (with Collapsed Branches)

Page 6 – 9.

The table on page 6 summarises this group of experiments.

In this group of experiments, some subtypes of the Normal Atlas were collapsed into major cell types: alpha-beta + gamma-delta T cells = T cells, inflammatory + non-inflammatory macrophages = macrophages, periportal + central venous LSEC = LSEC, mature + plasma B cells = B cells. All the remaining conditions of the experiments were identical to those described in the above section.

Intra-study Validation of Deconvolution – TME-Stroma Atlas

Page 10 – 14.

The table on page 10 summarises this group of experiments.

In this group of experiments, Cibersortx was trained with the TME-Stroma atlas and pseudobulk data were also generated from the TME-Stroma atlas.

Intra-study Validation of Deconvolution – TME-Iummne Atlas

Page 15 – 18.

The table on page 15 summarises this group of experiments.

In this group of experiments, Cibersortx was trained with the TME-Immune atlas and pseudobulk data were also generated from the TME-Immune atlas.

Intra-study Validation of Deconvolution – Cross-study validation

Page 19 – 29.

The table on pages 19-22 summarises this group of experiments.

The column "Cell type (Reference atlas)" denotes the atlas with which Cibersortx was trained. The column "Pseudobulk (Atlas)" denotes the atlas with which benchmarking pseudobulk data were generated.

In Silico Validation of Support Vector Regression – Normal Atlas

Page 30 – 33.

SVR was trained with pseudobulk generated from the Normal Atlas. Pseudobulk data for testing were also generated from the Normal Atlas. Cell labelling was according to the authors' instructions. The horizontal axis represents the predefined proportion while the vertical axis shows the predicted value by SVR.

In Silico Validation of Support Vector Regression – TME-Stroma Atlas

Page 34 – 37.

SVR was trained with pseudobulk generated from the TME-Stroma Atlas. Pseudobulk data for testing were also generated from the TME-Stroma Atlas.

In Silico Validation of Support Vector Regression – TME-Immune Atlas

Page 38 – 40.

SVR was trained with pseudobulk generated from the TME-Immune Atlas. Pseudobulk data for testing were also generated from the TME-Immune Atlas.

In Silico Validation of Support Vector Regression – Other Cells

Page 41 – 43.

These three pages include the corresponding validation experiments as the counterparts of S2 – Estimation by Cibersortx and Support Vector Regression – Other Cell Types, Pages 4 – 6.

S5 – Survival Impacts of Cell Fractions Estimated by Cibersortx – TCGA-LIHC

The figures were laid vertically. If one cell type has three panels, the survival analysis was in optimised strategy. Otherwise, it was in median-point strategy with two panels. The third panel shows the distribution of Cibersortx-estimated values. Horizontal lines indicate the cutting thresholds in optimised strategy.

Page 1. alpha-beta T Cells (Normal Atlas), gamma-delta T Cells (Normal Atlas).

Page 2. Cholangiocytes (Normal Atlas), Hepatic Stellate Cells (Normal Atlas).

Page 3. Central Venous LSECs (Normal Atlas), Periportal LSECs (Normal Atlas).

Page 4. Inflammatory Macrophage (Normal Atlas), Non-inflammatory Macrophage (Normal Atlas).

Page 5. Mature B Cells (Normal Atlas), Plasma Cells (Normal Atlas).

Page 6. Portal Endothelial Cells (Normal Atlas), NK-like Cells (Normal Atlas).

Page 7. Hepatocytes (Normal Atlas).

Page 8. cDC1 (TME-Stroma Atlas), cDC2 (TME-Stroma Atlas).

Page 9. LSECs (TME-Stroma Atlas), cDC2 (TME-Stroma Atlas).

Page 10. Stellate Cells (TME-Stroma Atlas), CAFs (TME-Stroma Atlas).

Page 11. LVECs (TME-Stroma Atlas), LVECts (TME-Stroma Atlas).

Page 12. SAMs (TME-Stroma Atlas), TM1 (TME-Stroma Atlas).

Page 13. Kupffer Cells (TME-Stroma Atlas), Proliferating Cells (TME-Stroma Atlas).

Page 14. vSMCs (TME-Stroma Atlas).

Page 15. Hepatocytes (TME-Immune Atlas), B Cells (TME-Immune Atlas).

Page 16. $CD4^+$ Cells (TME-Immune Atlas), $CD8^+$ Cells (TME-Immune Atlas).

Page 17. Regulatory T Cells (TME-Immune Atlas), NK Cells (TME-Immune Atlas).

Page 18. Fibroblasts (TME-Immune Atlas), Endothelial Cells (TME-Immune Atlas).

Page 19. Mast Cells (TME-Immune Atlas), Myeloid Cells (TME-Immune Atlas).

Page 20. Bi-potent Cells (TME-Immune Atlas).

S6 – Survival Impacts of Cell Fractions Estimated by Cibersortx – GSE14520

Page 1. alpha-beta T Cells (Normal Atlas), gamma-delta T Cells (Normal Atlas), Cholangiocytes (Normal Atlas).

Page 2. Hepatic Stellate Cells (Normal Atlas), Central Venous LSECs (Normal Atlas), Periportal LSECs (Normal Atlas).

Page 3. Inflammatory Macrophage (Normal Atlas), Non-inflammatory Macrophage (Normal Atlas), Mature B Cells (Normal Atlas).

Page 4. Plasma Cells (Normal Atlas), Portal Endothelial Cells (Normal Atlas), NK-like Cells (Normal Atlas).

Page 5. cDC1 (TME-Stroma Atlas), cDC2 (TME-Stroma Atlas), LSECs (TME-Stroma Atlas).

Page 6. Pericytes (TME-Stroma Atlas), Stellate Cells (TME-Stroma Atlas), CAFs (TME-Stroma Atlas).

Page 7. LVECs (TME-Stroma Atlas), LVECts (TME-Stroma Atlas), SAMs (TME-Stroma Atlas).

Page 8. TM1 (TME-Stroma Atlas), Kupffer Cells (TME-Stroma Atlas), Proliferating Cells (TME-Stroma Atlas).

Page 9. vSMCs (TME-Stroma Atlas).

Page 10. Hepatocytes (TME-Immune Atlas), B Cells (TME-Immune Atlas), $CD4^{+}$ Cells (TME-Immune Atlas).

Page 11. $CD8^{+}$ Cells (TME-Immune Atlas), Regulatory T Cells (TME-Immune Atlas), Fibroblasts (TME-Immune Atlas).

Page 12. NK Cells (TME-Immune Atlas), Mast Cells (TME-Immune Atlas), Myeloid Cells (TME-Immune Atlas).

Page 13. Bi-Potent Cell (TME-Immune Atlas).

S7 – Pathway Analysis

Page 1. PROGENy estimation. MAPK, p53.

Page 2. PROGENy estimation. JAK-STAT, NFkB.

Page 3. PROGENy estimation. NFkB, PI3K.

Page 4. PROGENy estimation. NGFb, TNFa.

Page 5. PROGENy estimation. VEGF, Trail.

Page 6. PROGENy estimation. Androgen, Estrogen.

Page 7-21. GSEA analysis for the cell type marker genes selected by Cibersortx.

Table S1 – Summary of the Datasets Used in This Study

This table describes the general information of the datasets recruited in this study.

Table S2 – Summary of Survival Impact

This table includes the summary for S4 – Survival Impacts of Cell Fractions Estimated by Cibersortx – TCGA-LIHC, and S5 – Survival Impacts of Cell Fractions Estimated by Cibersortx – GSE14520.
