# Supplementary figures and images for: Deciphering Tumour Microenvironment of Liver Cancer through Deconvolution of Bulk RNA-Seq Data with Single-Cell Atlas
Source: Cancers (Basel). 2022 Dec 27;15(1):153. doi: 10.3390/cancers15010153 (PMC9818189; doi:10.3390/cancers15010153)

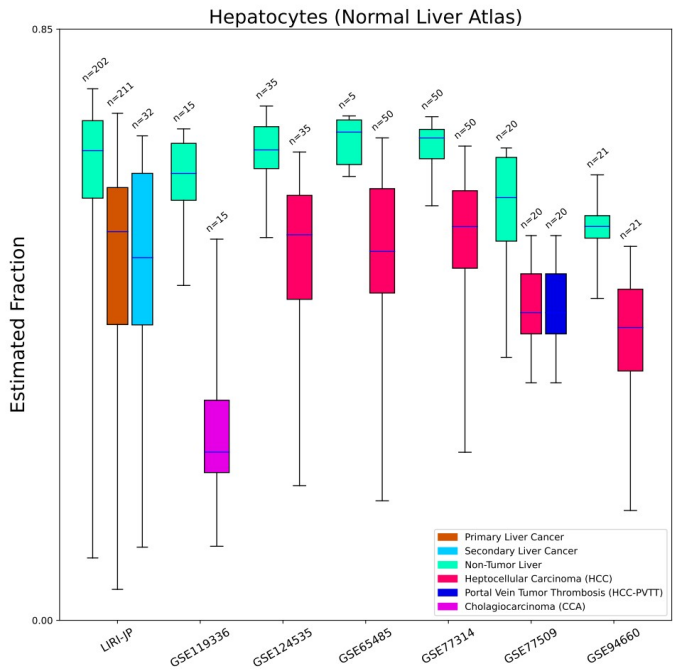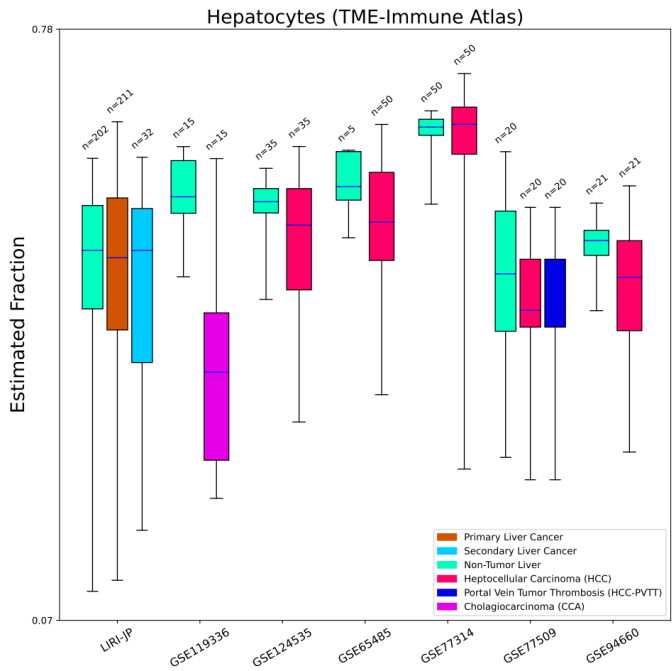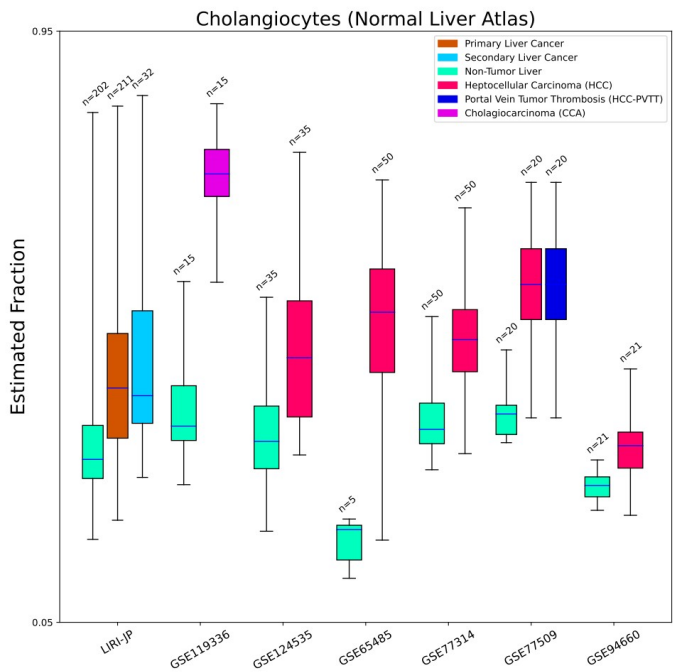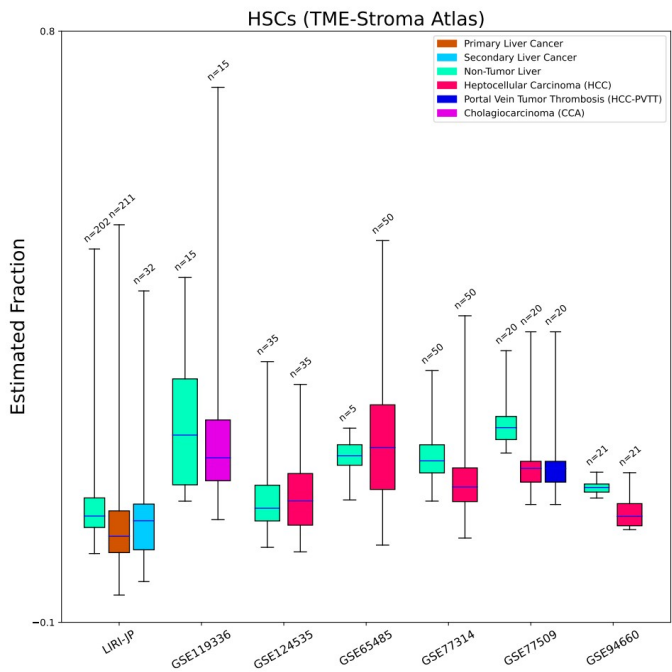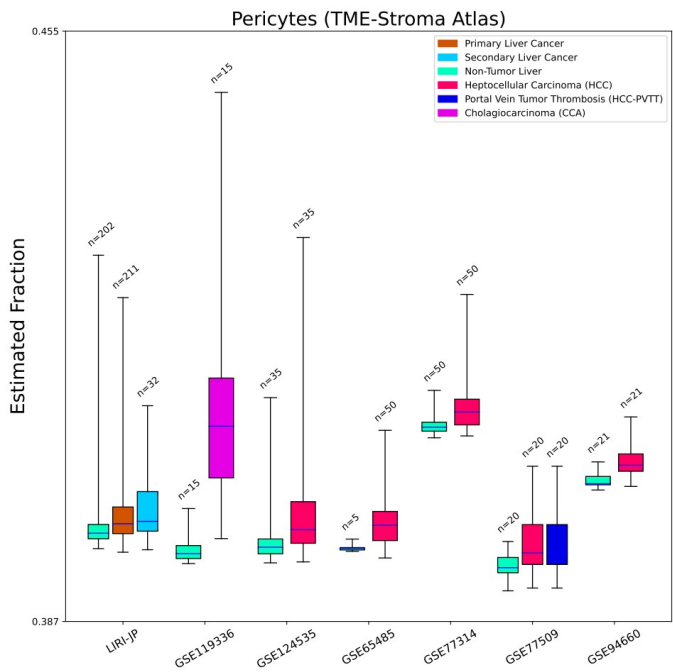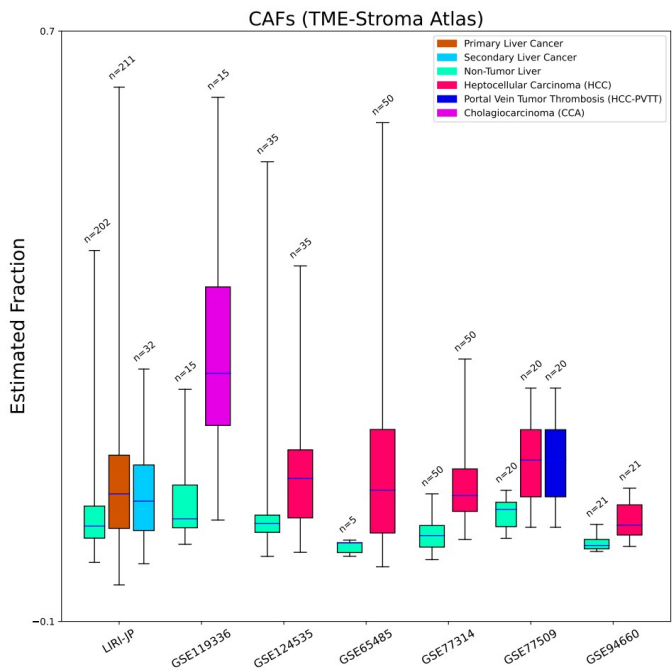

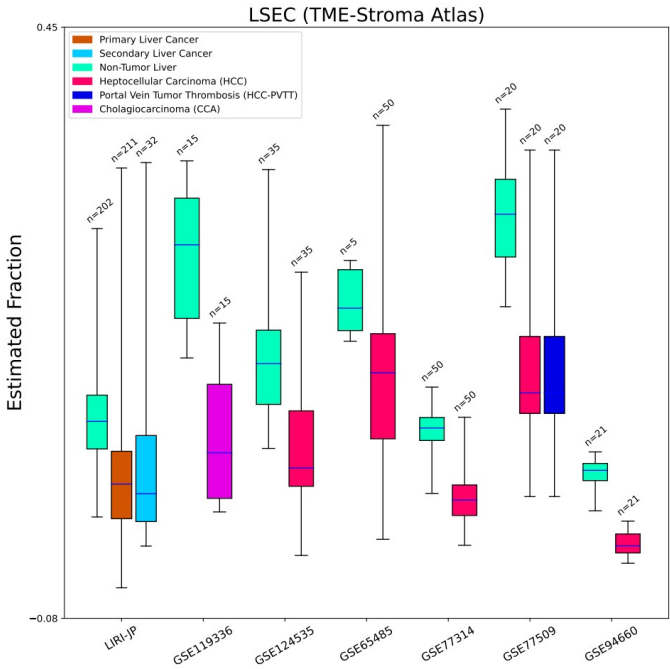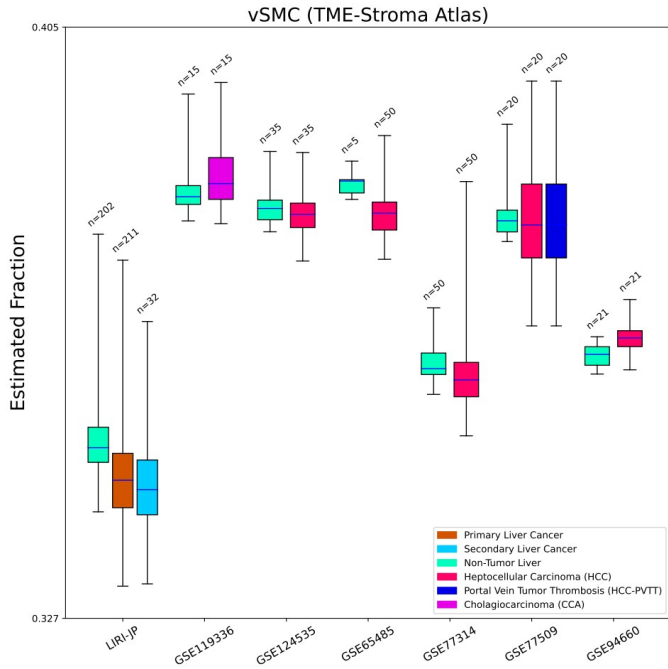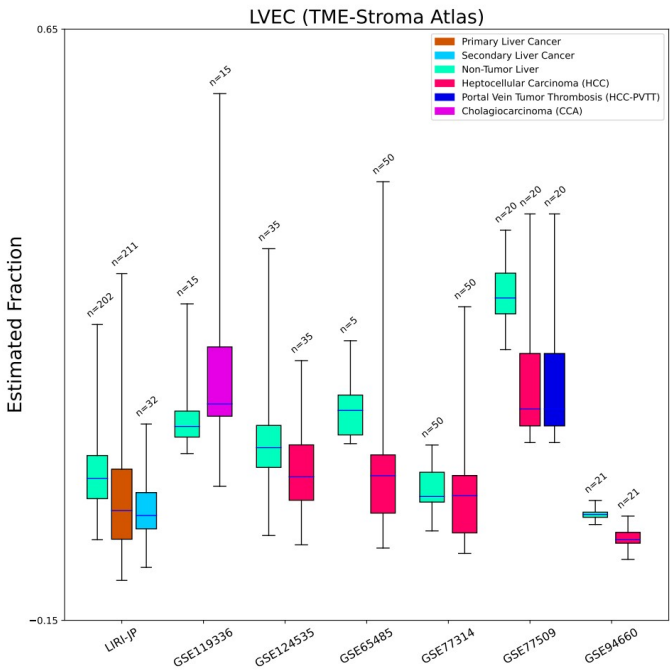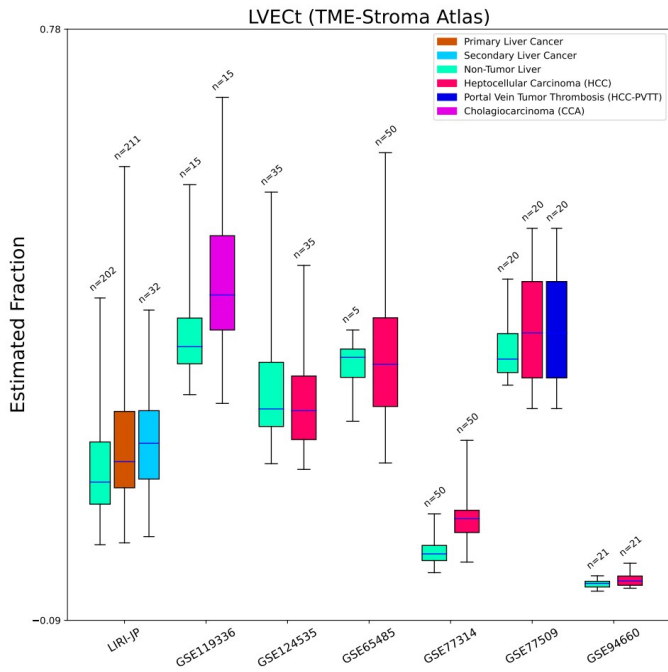

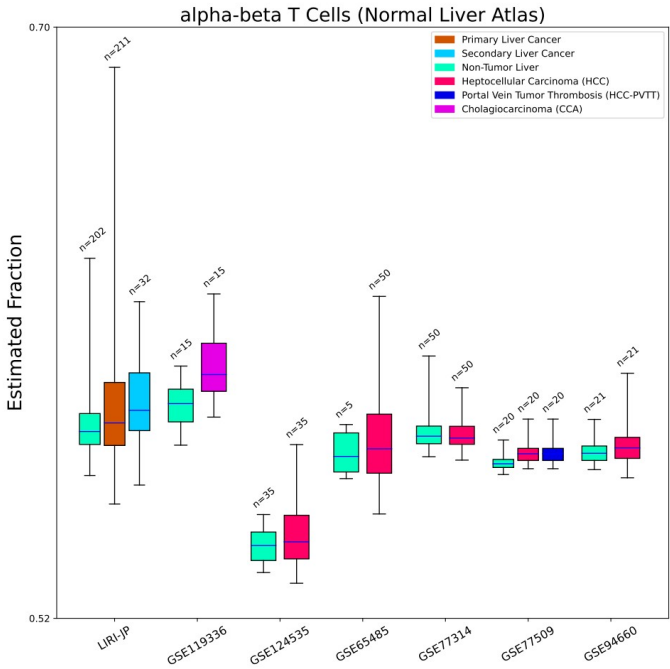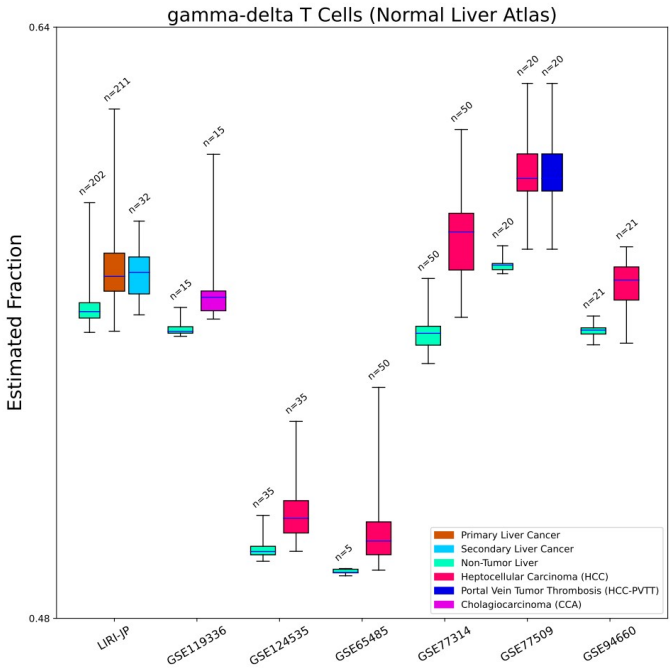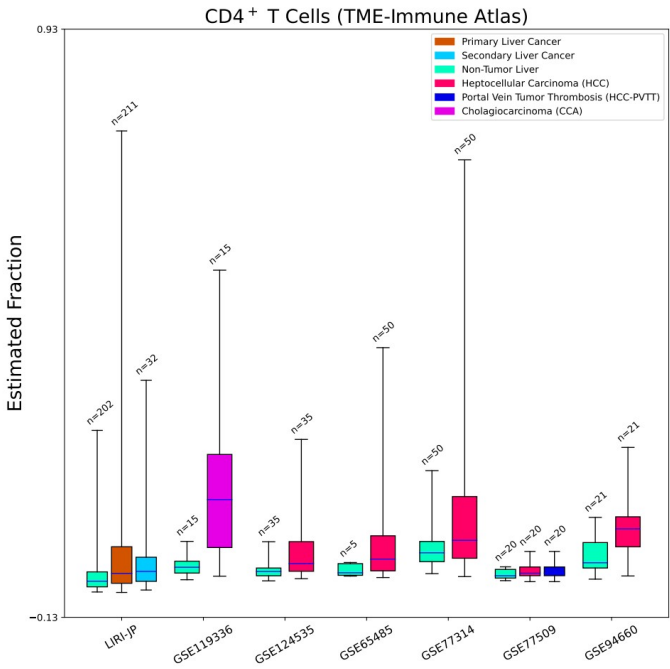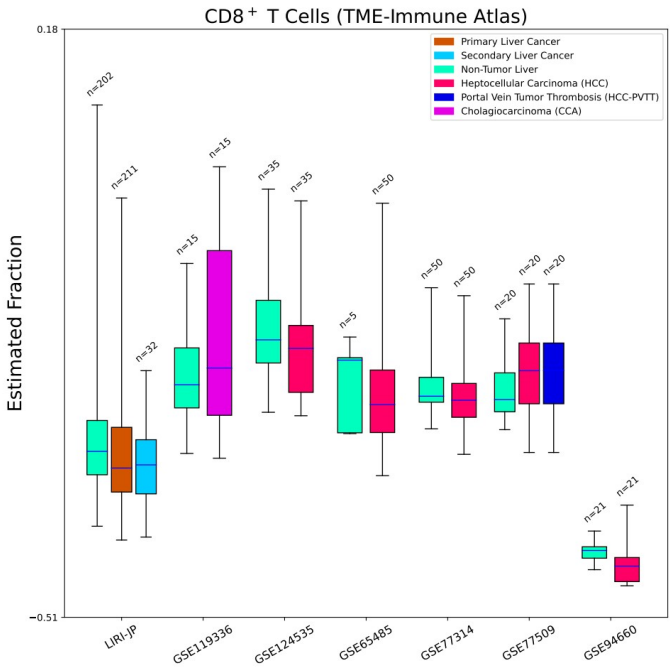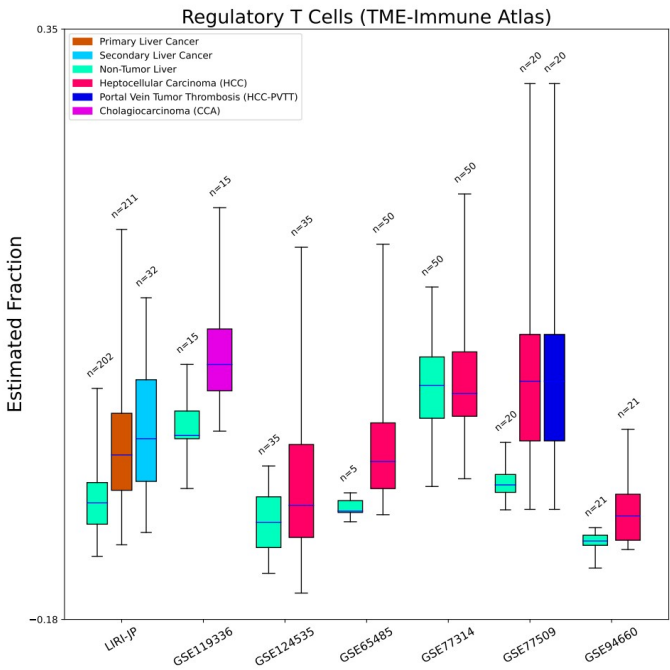

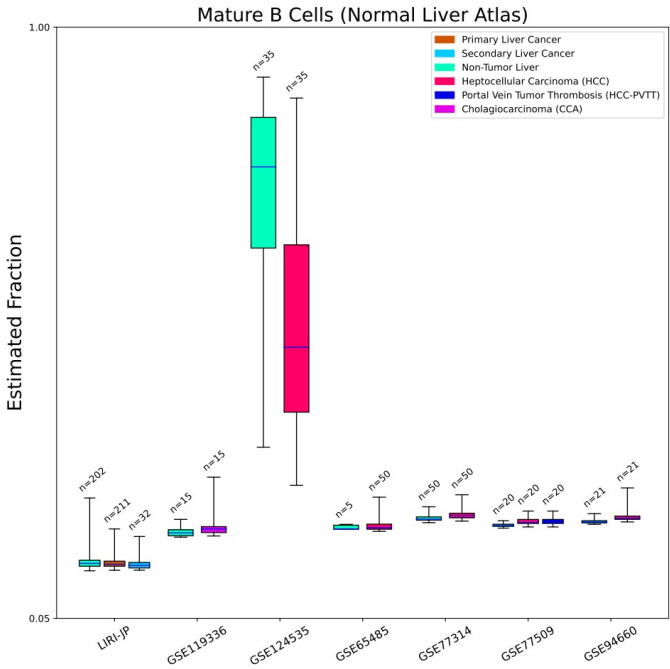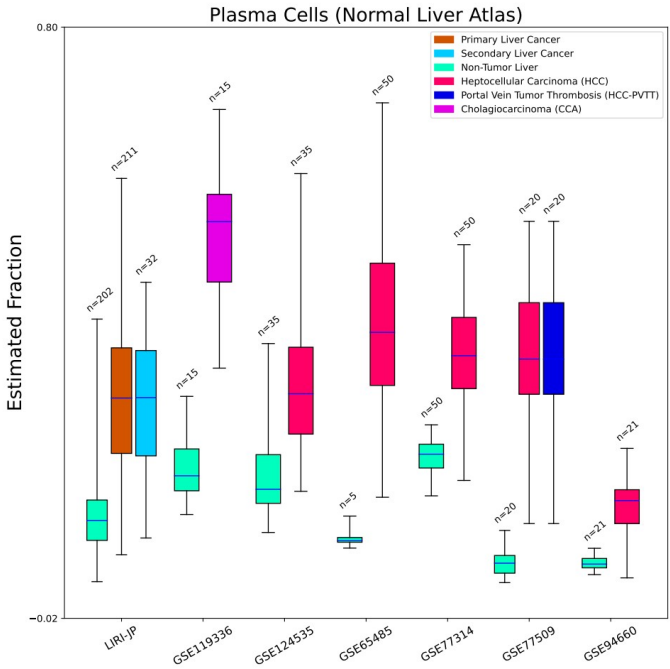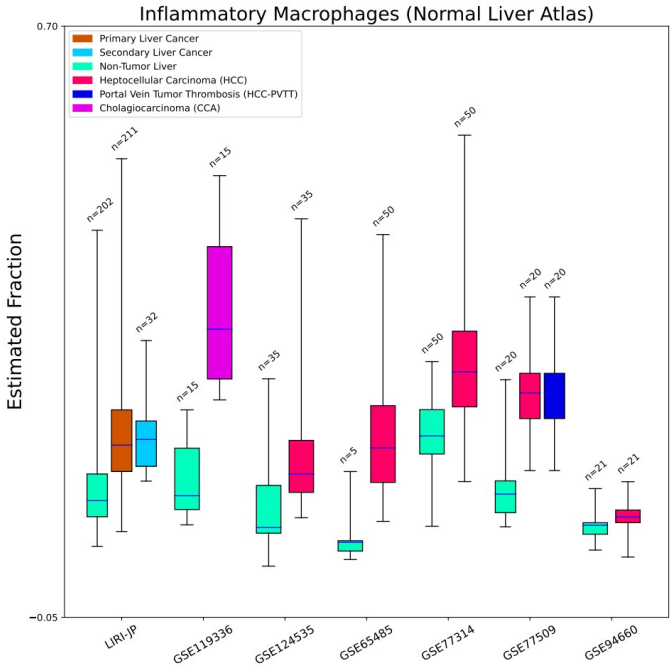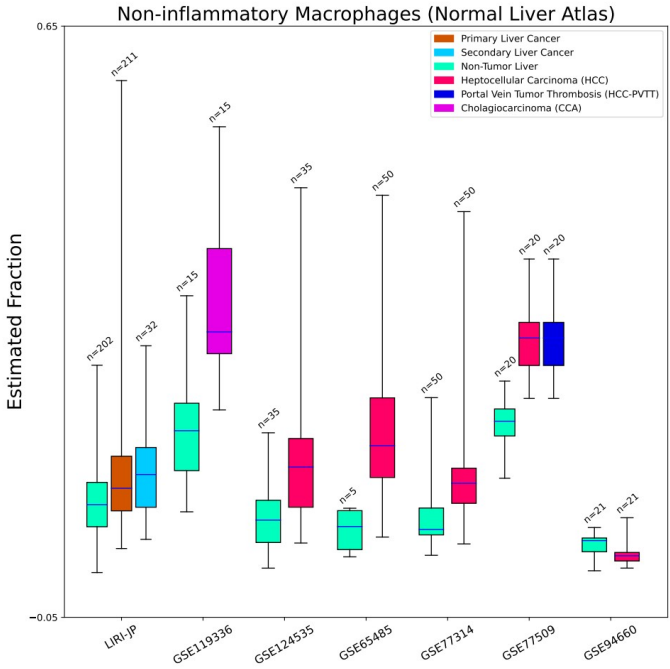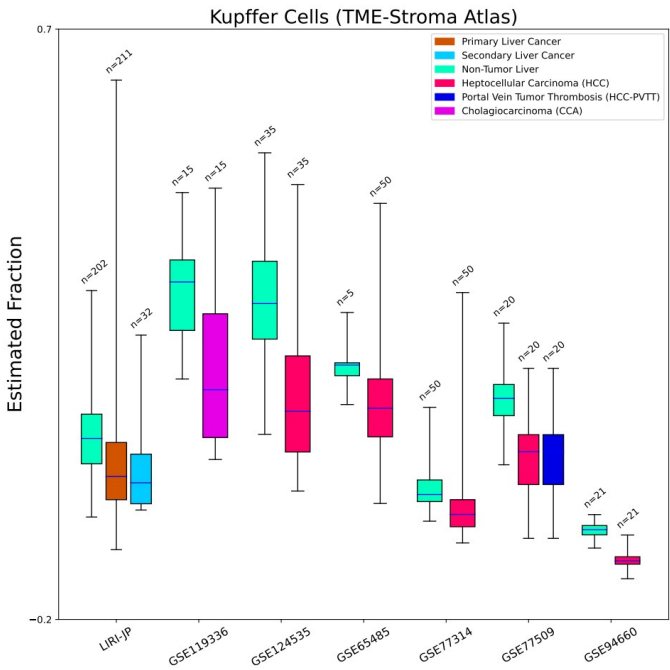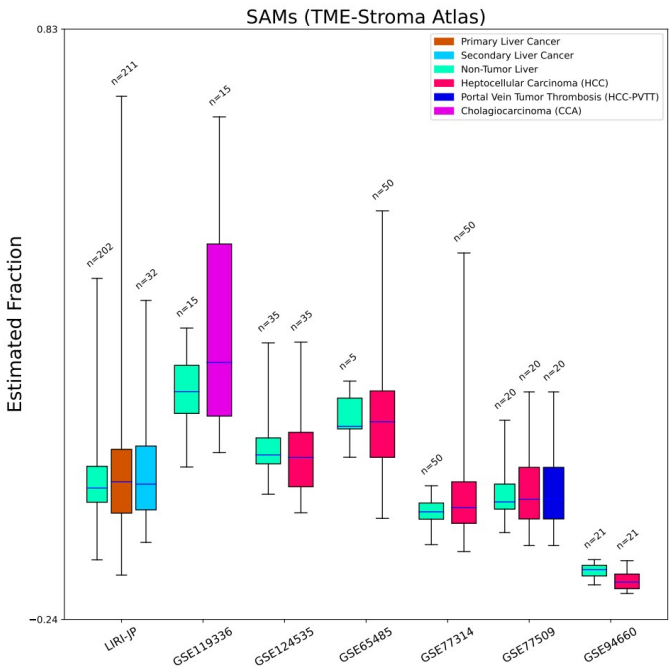

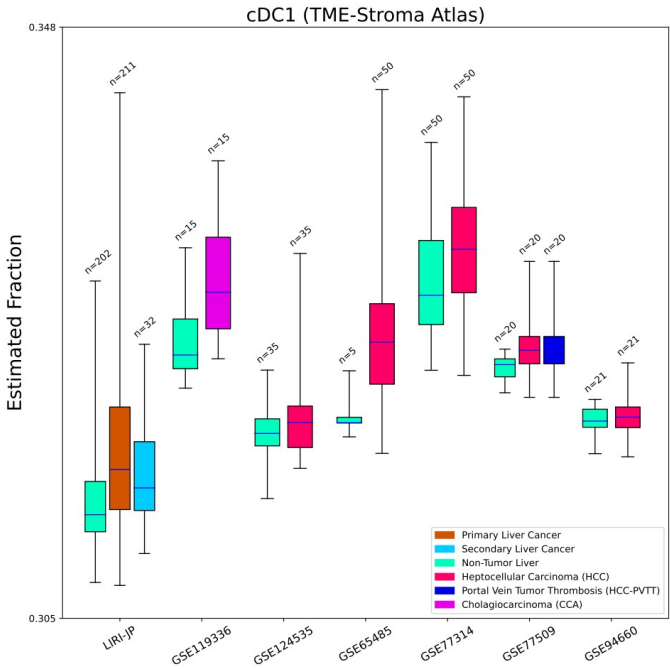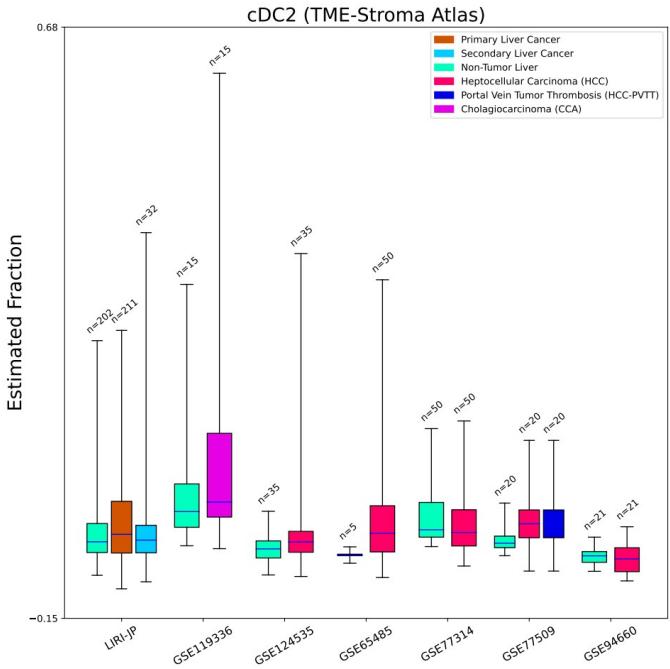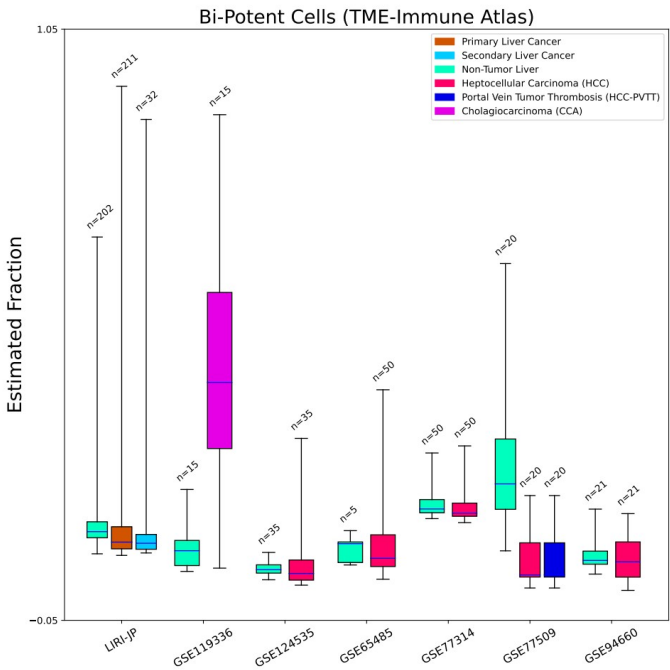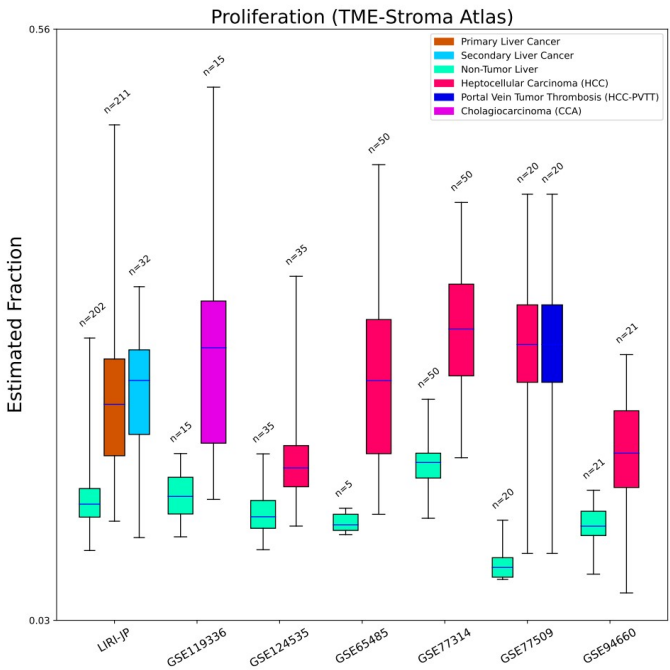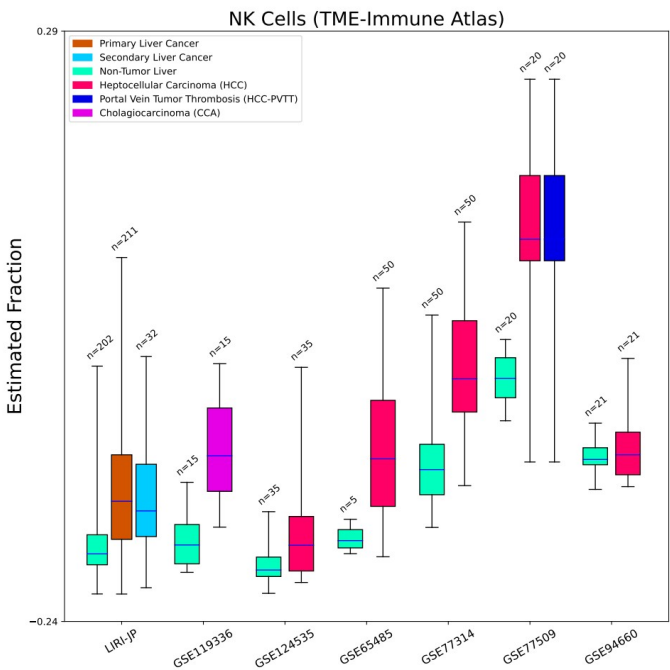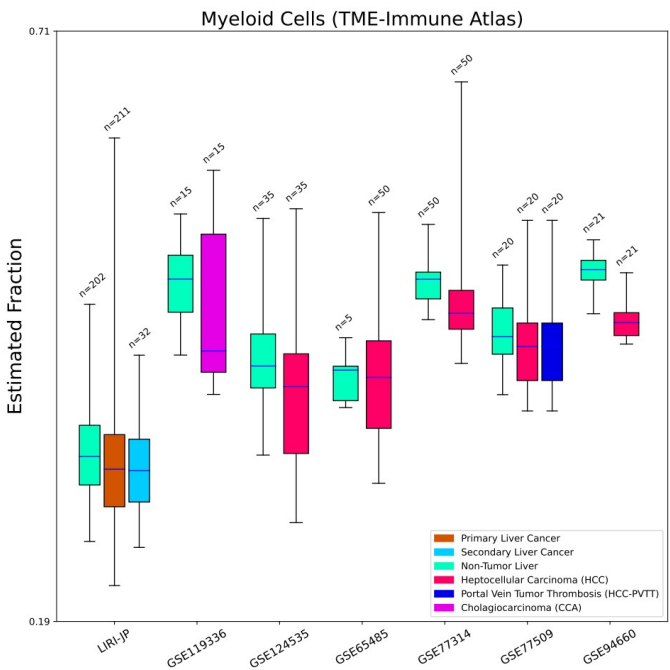

Supplement: Supplementary file 1 [file cancers-15-00153-s001.zip › cancers-2059594-supplementary/Supplements/S1_SVR_Estimation.pdf]

A

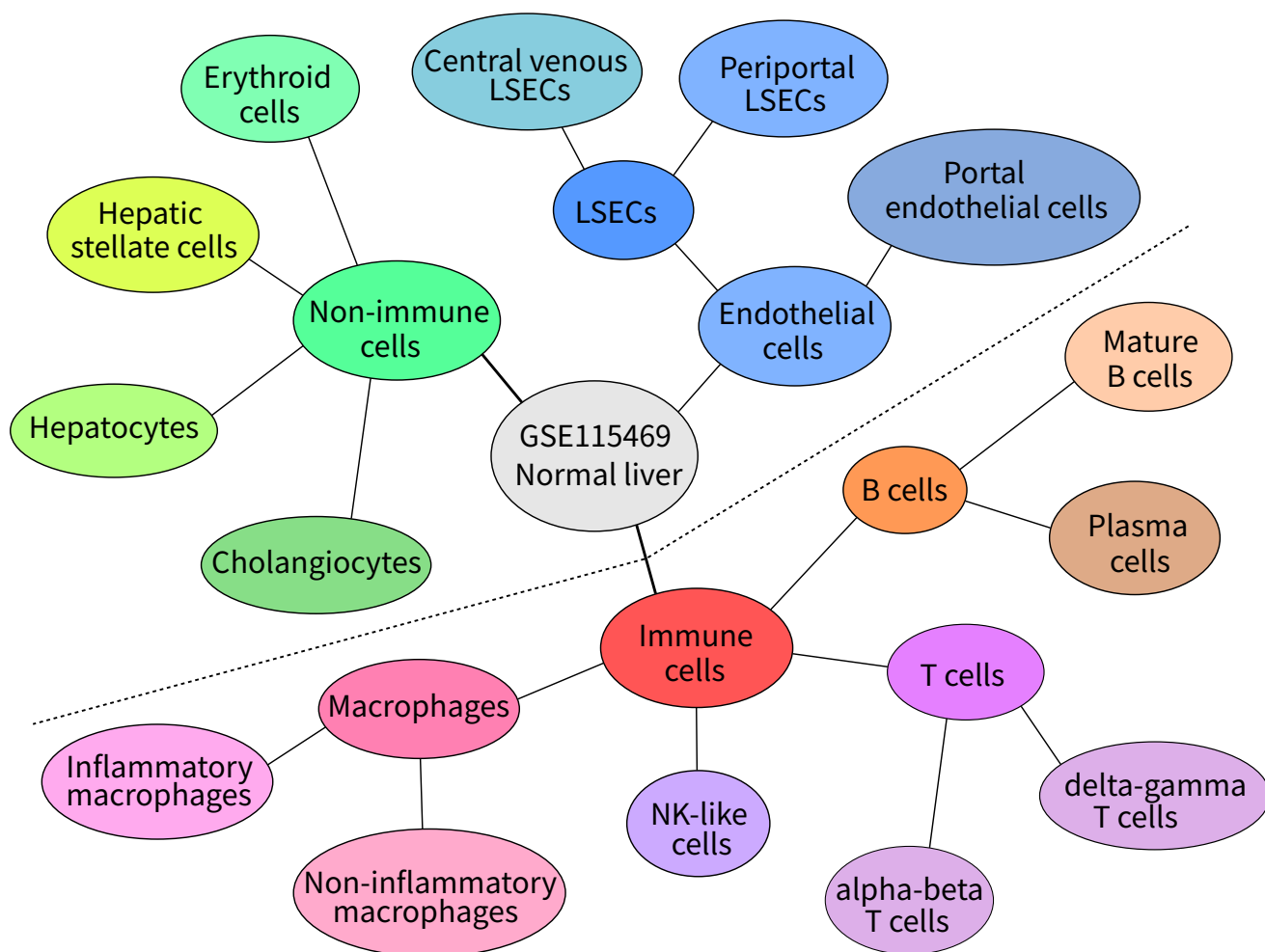

B

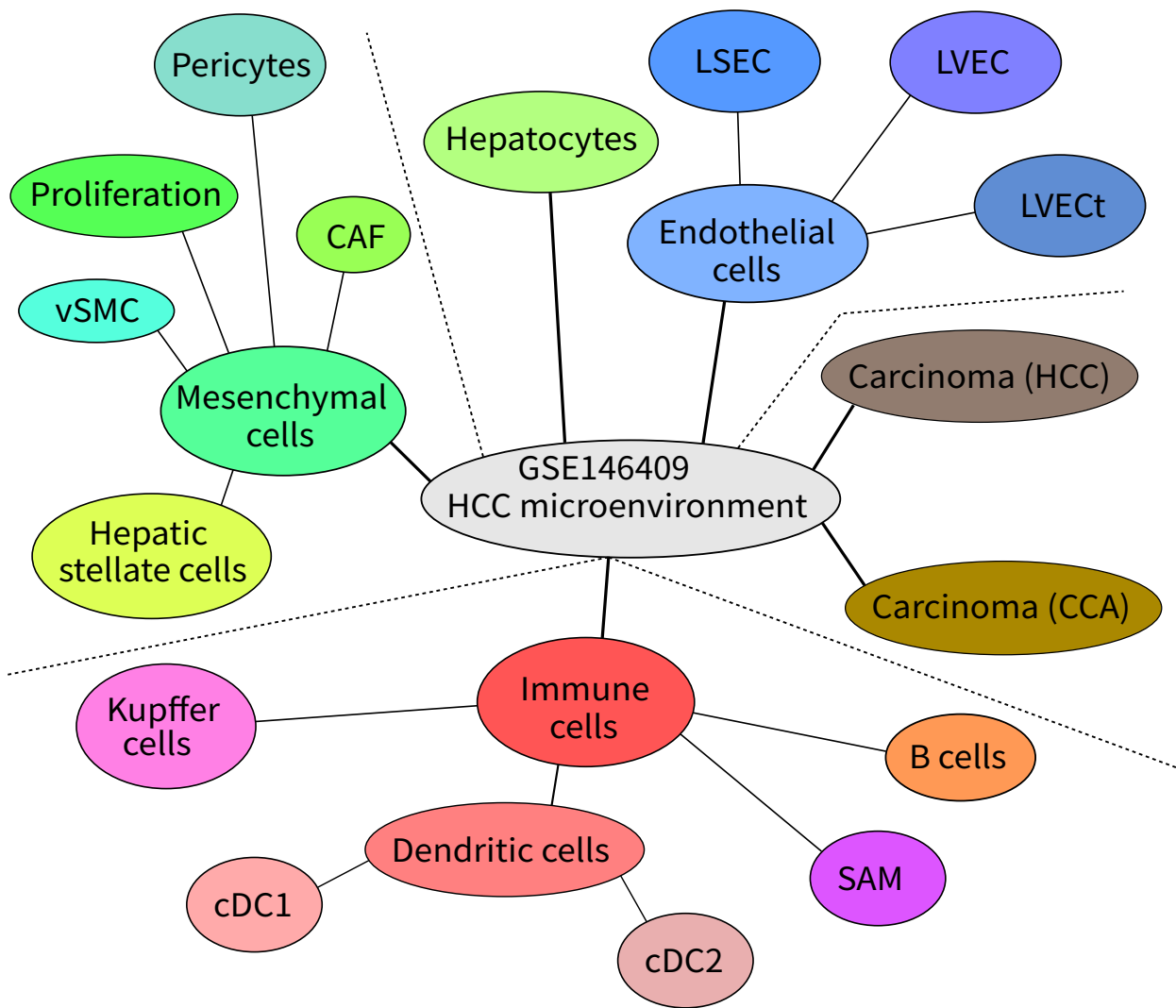

C

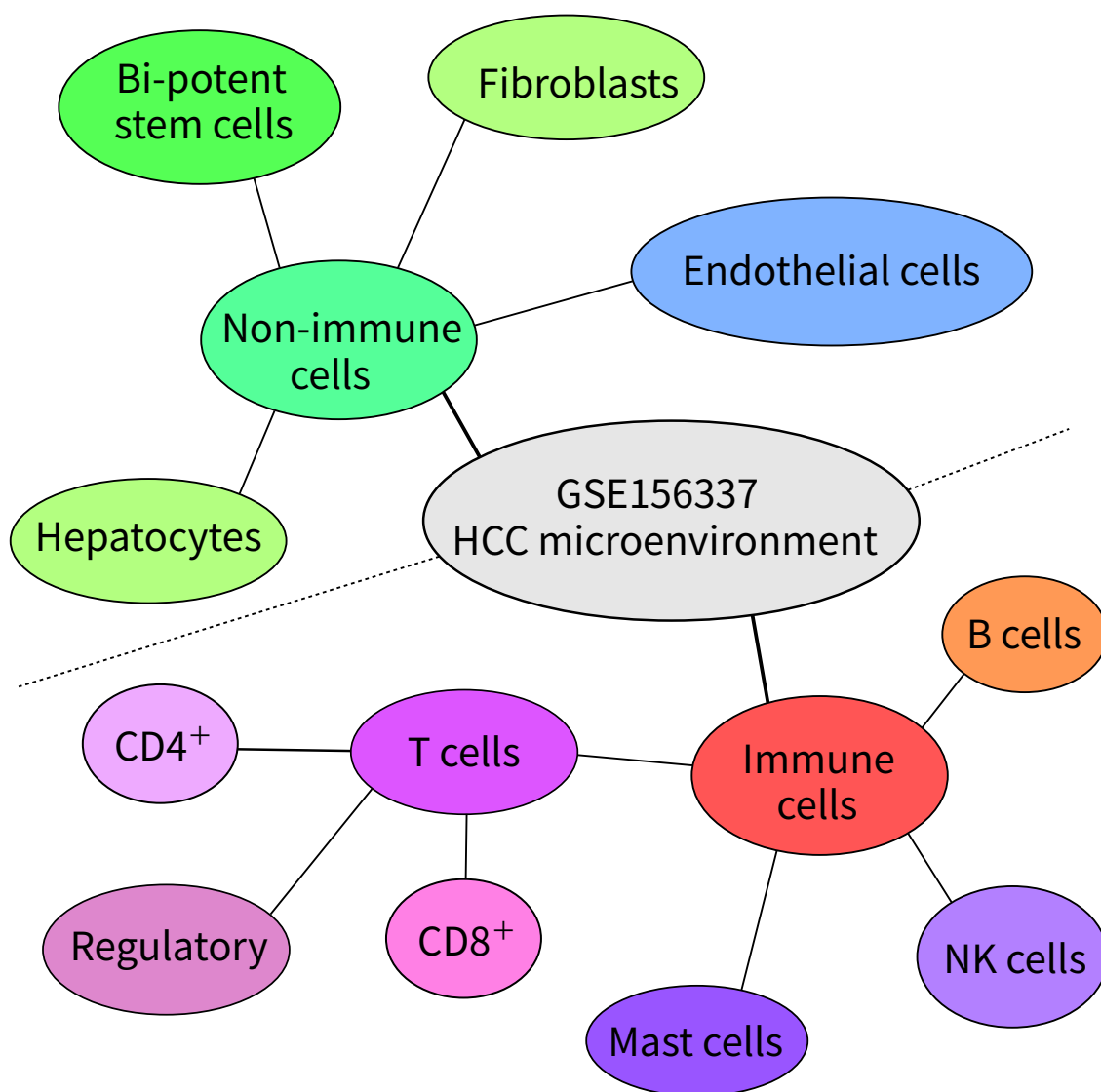

Supplement: Supplementary file 1 [file cancers-15-00153-s001.zip › cancers-2059594-supplementary/Supplements/S2_Cell_Type_Hierarchy.pdf]

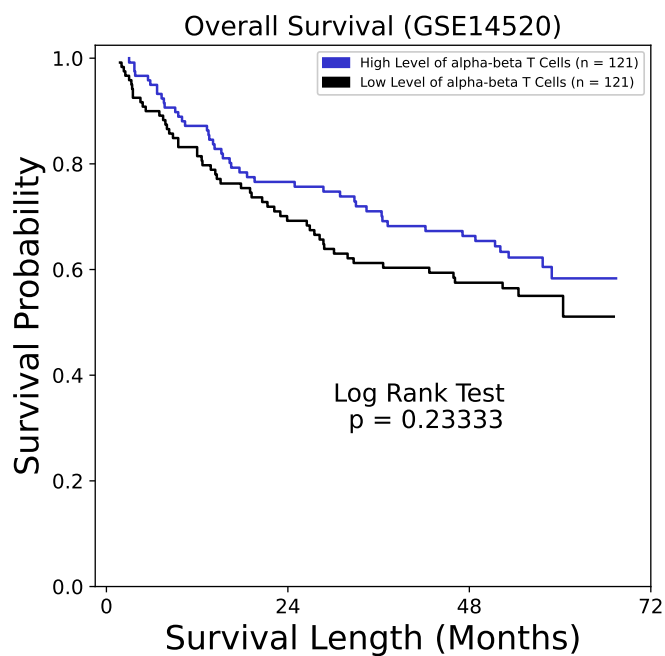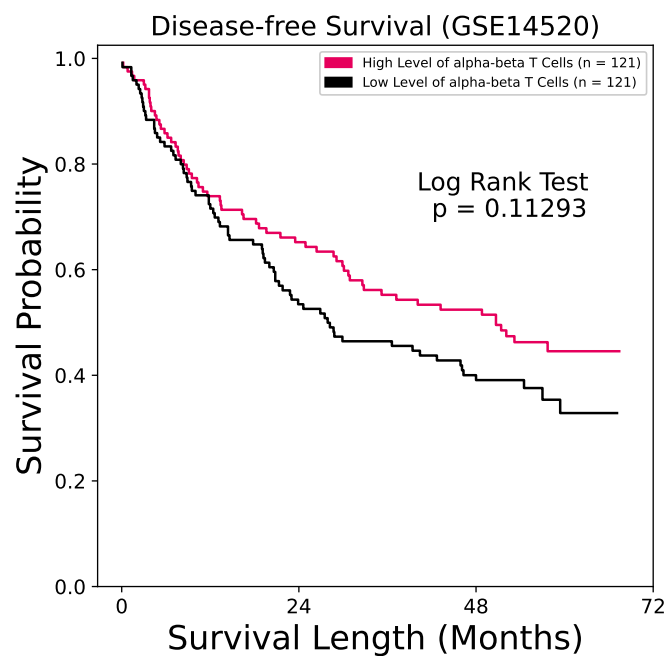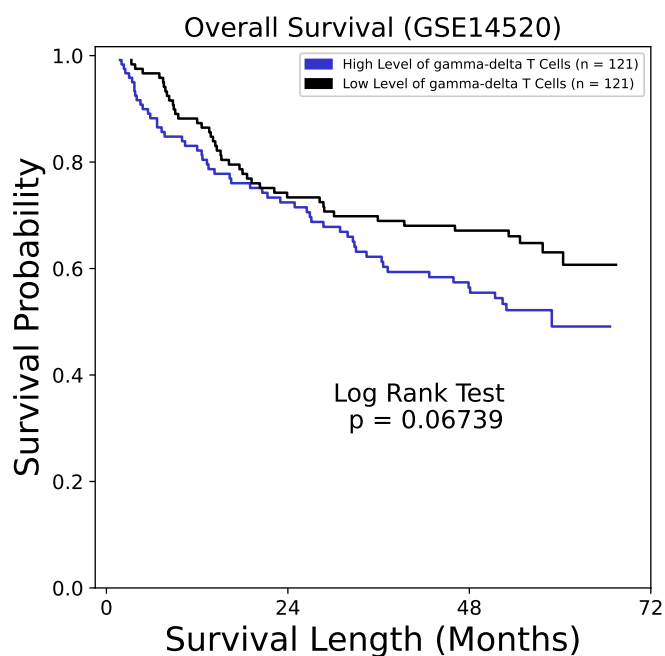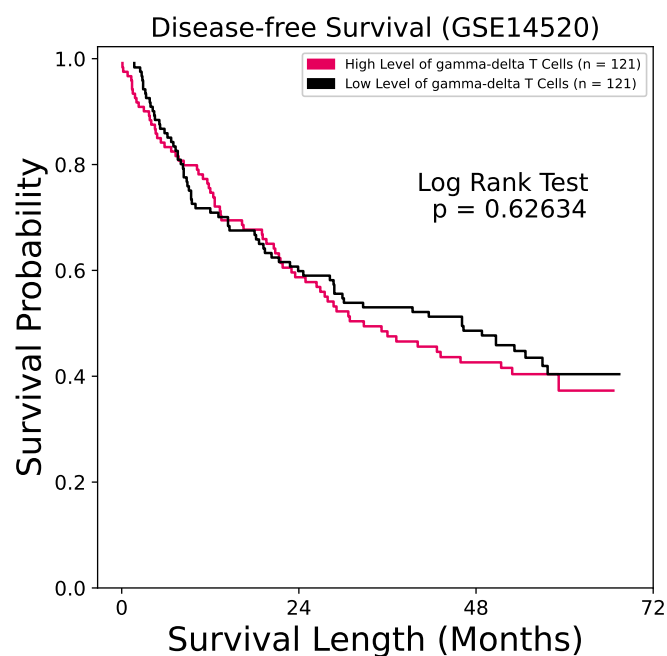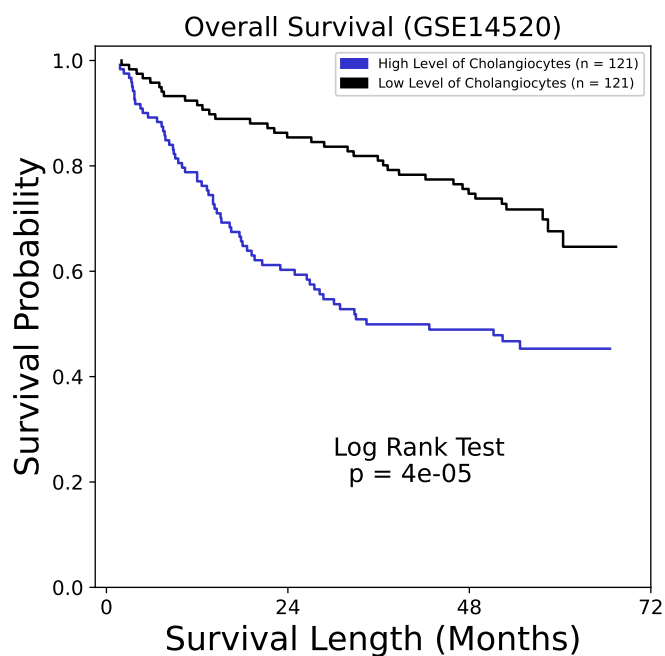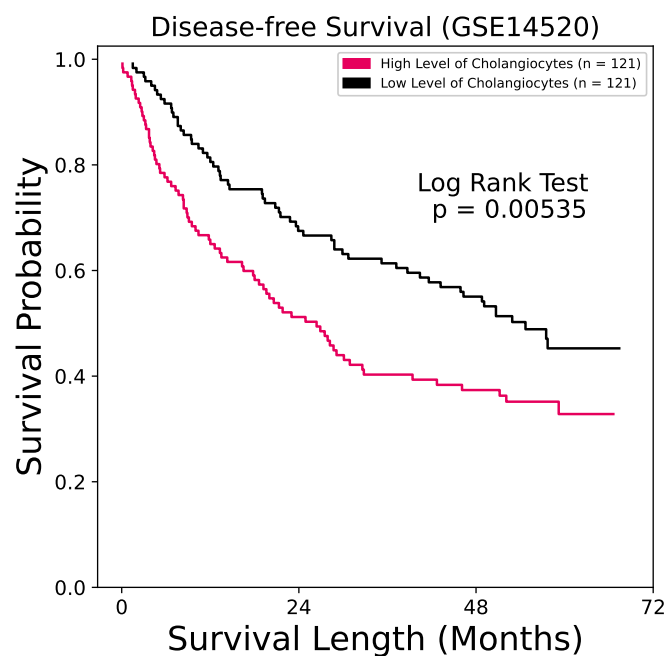

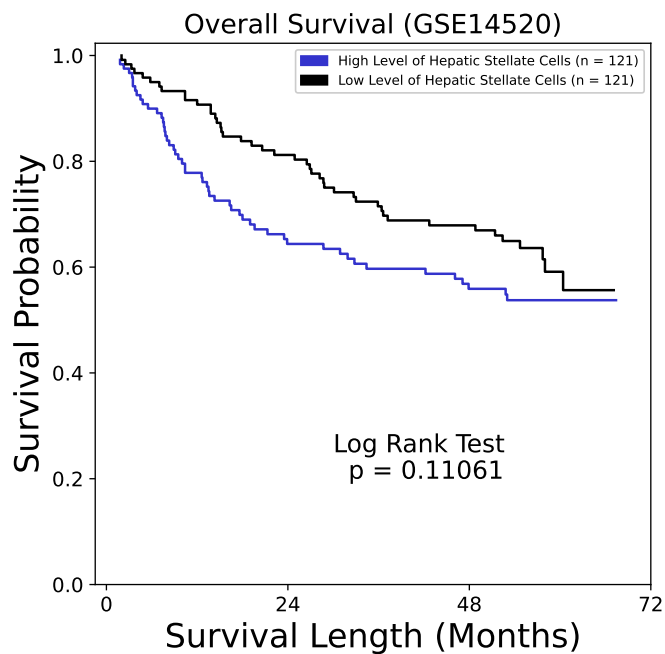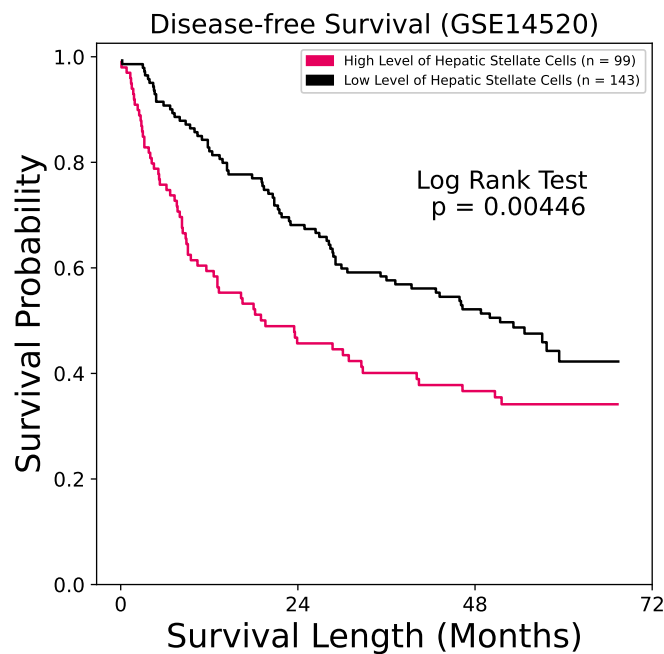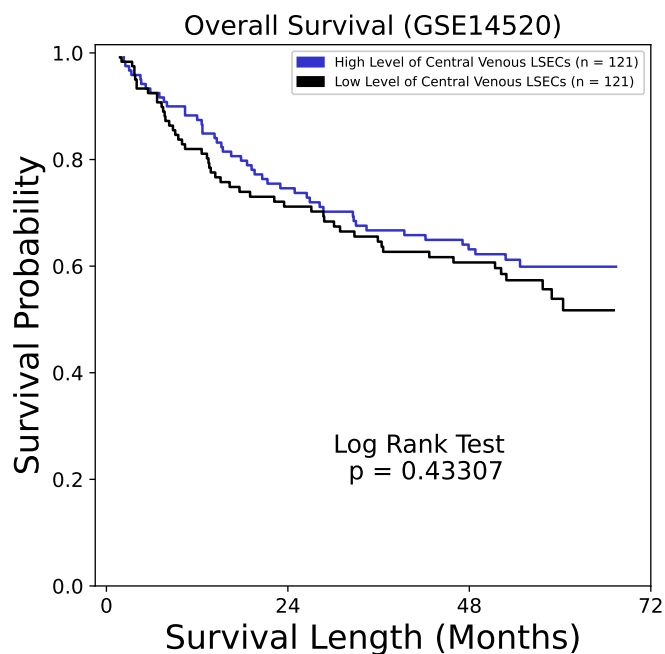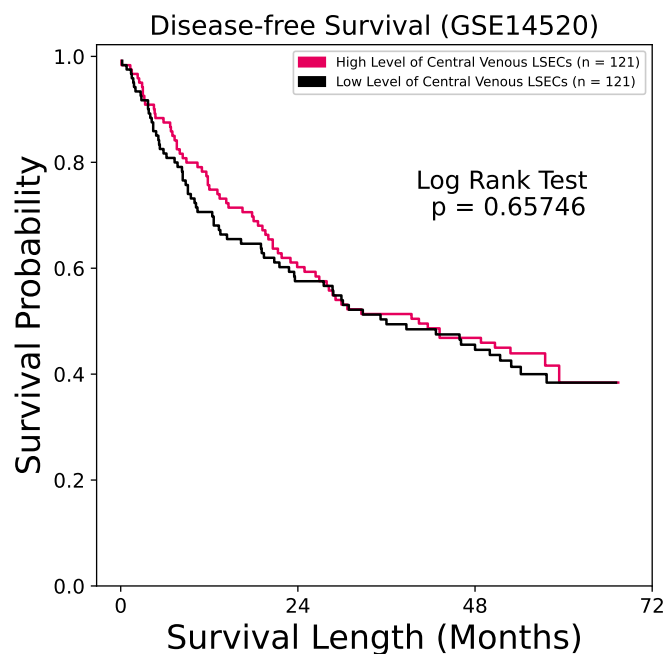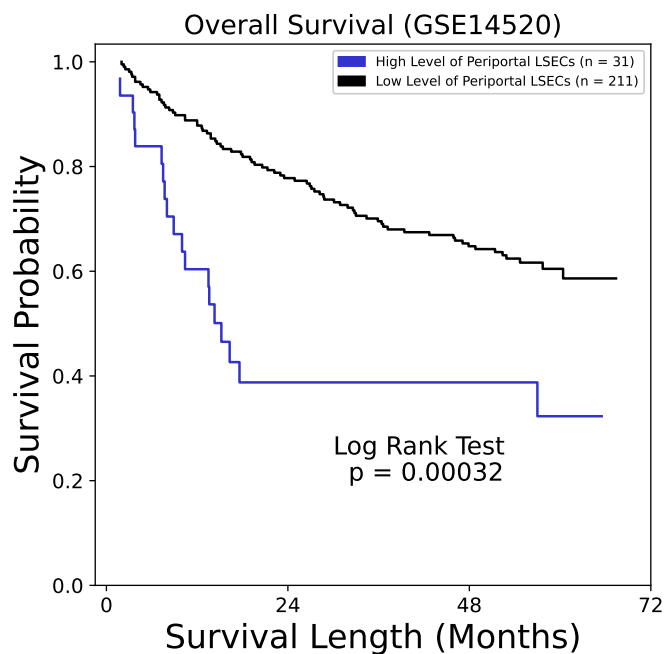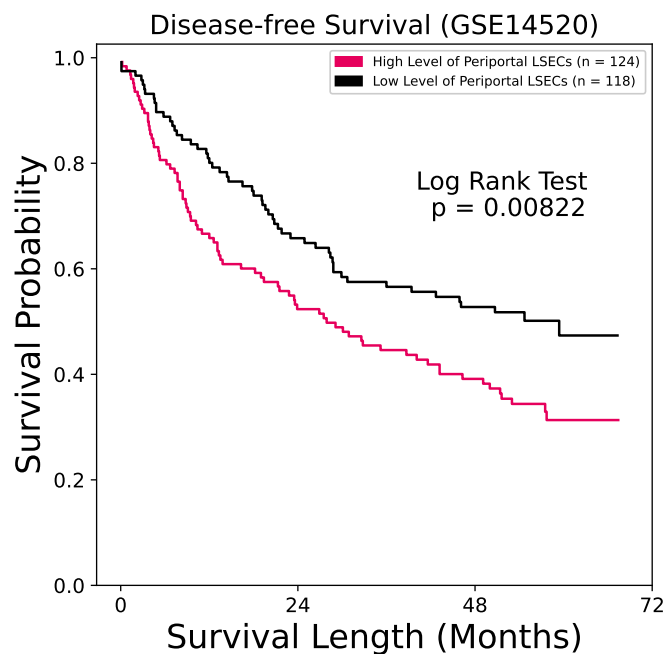

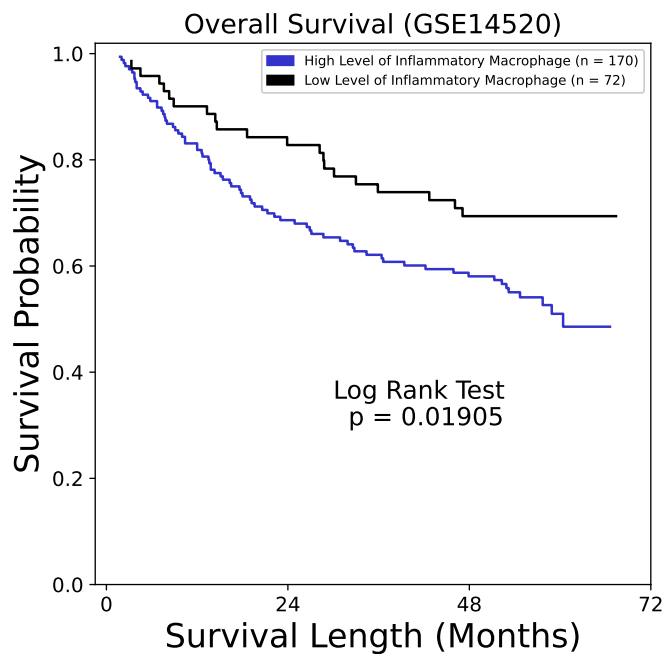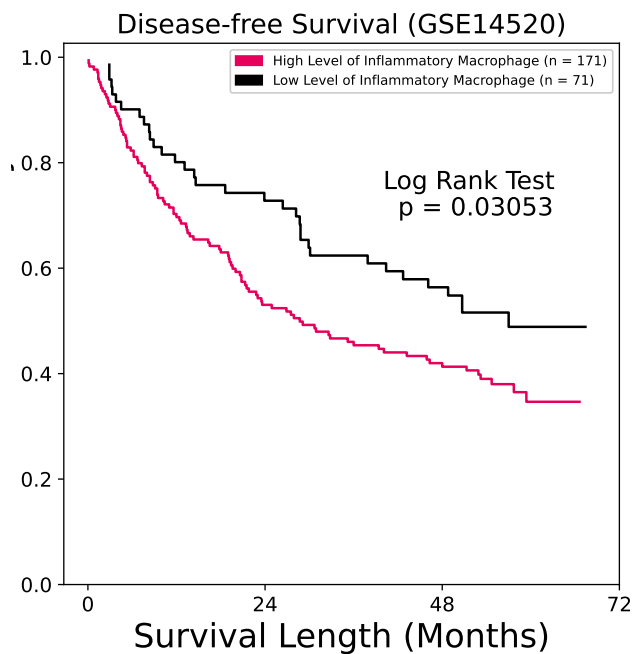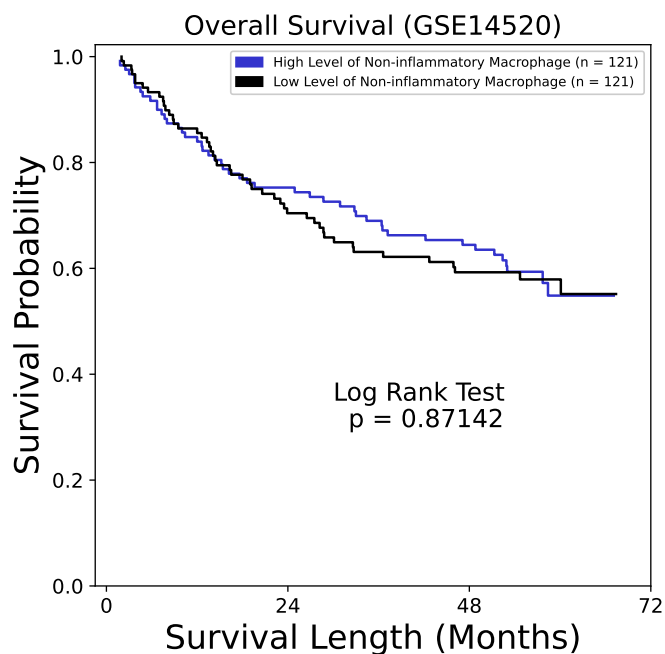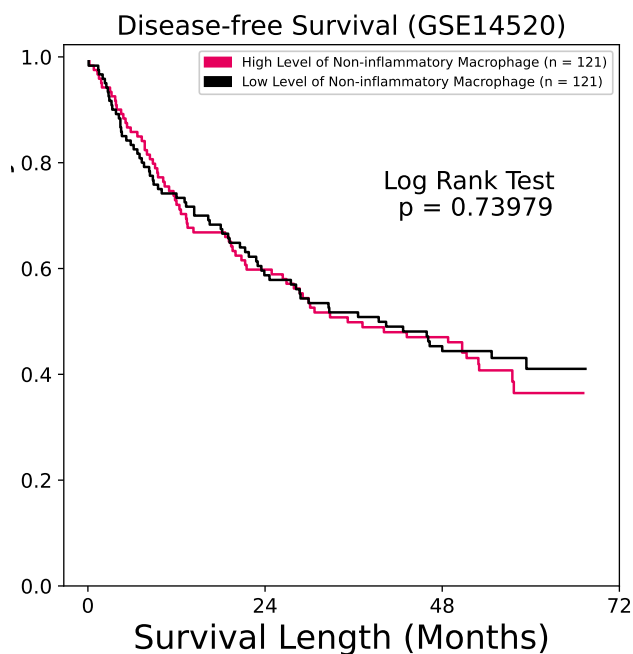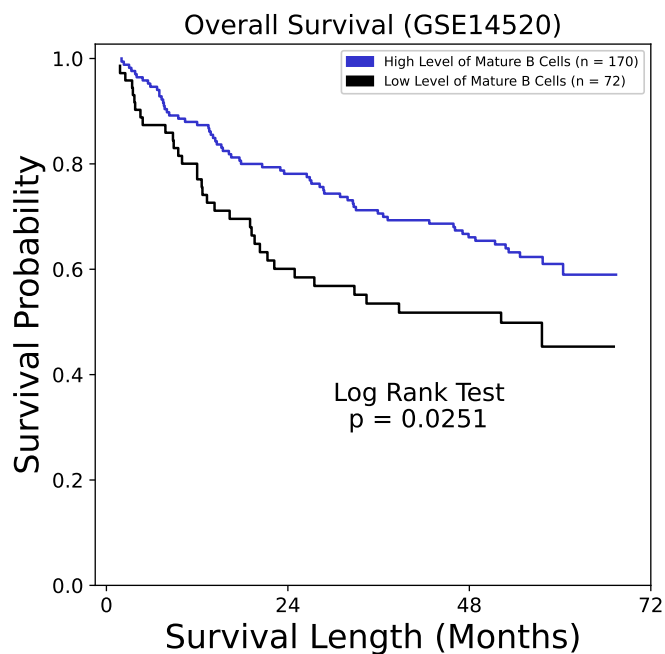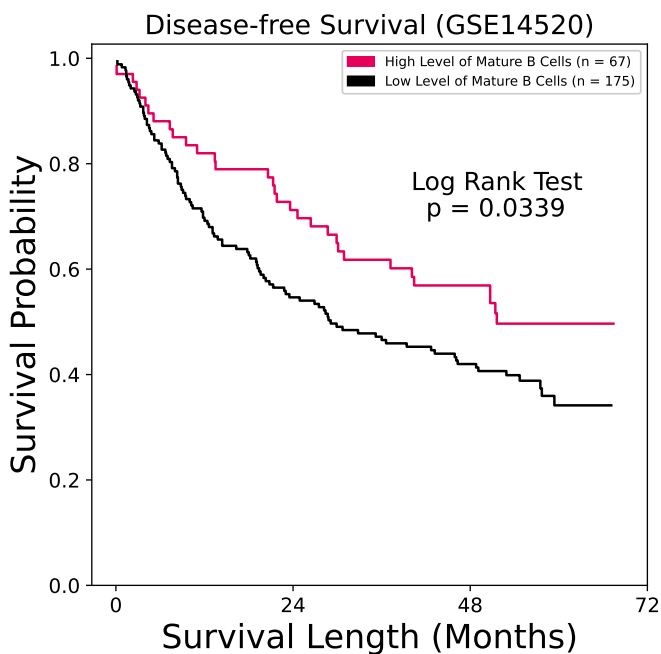

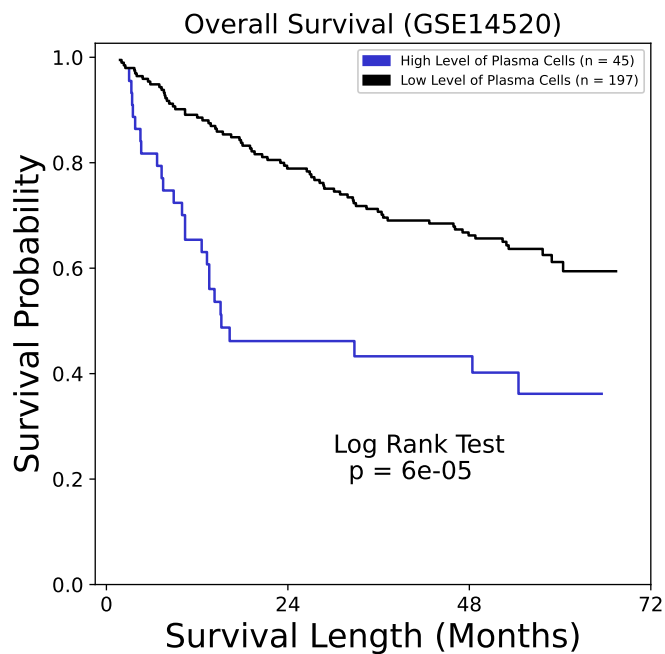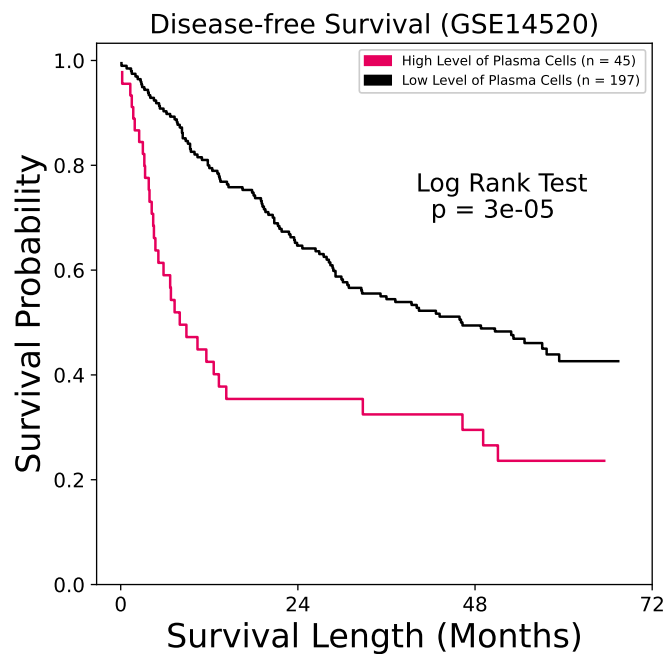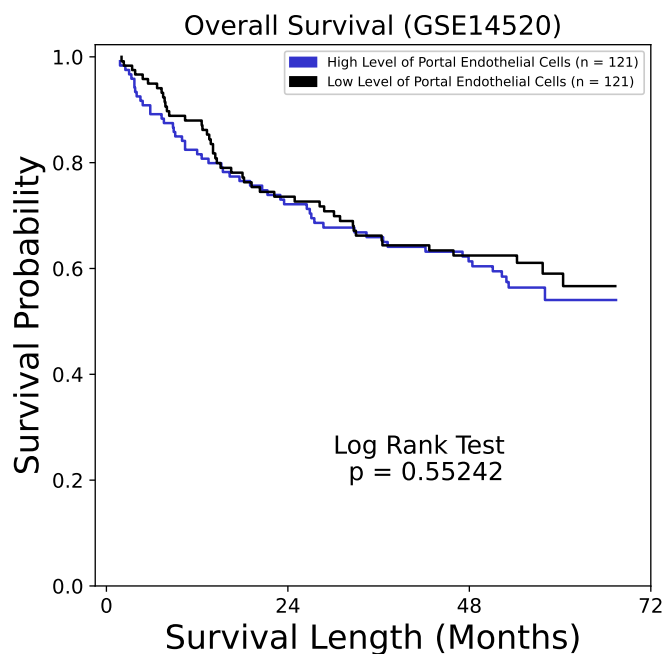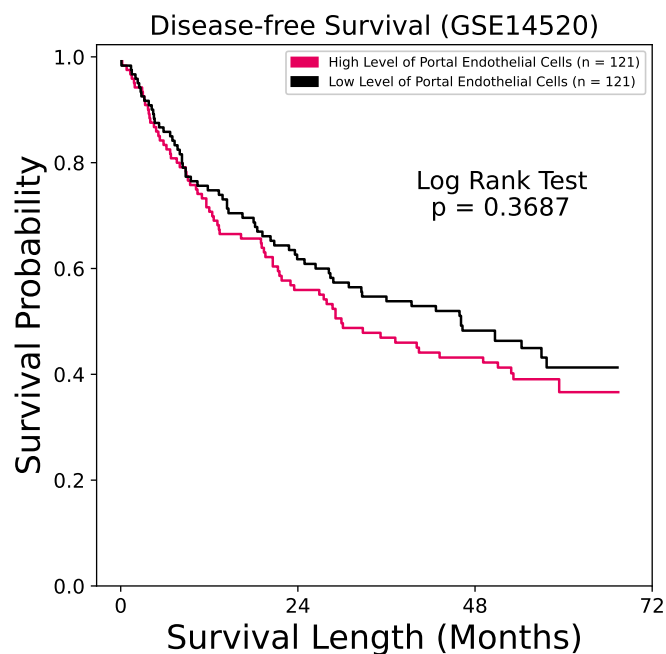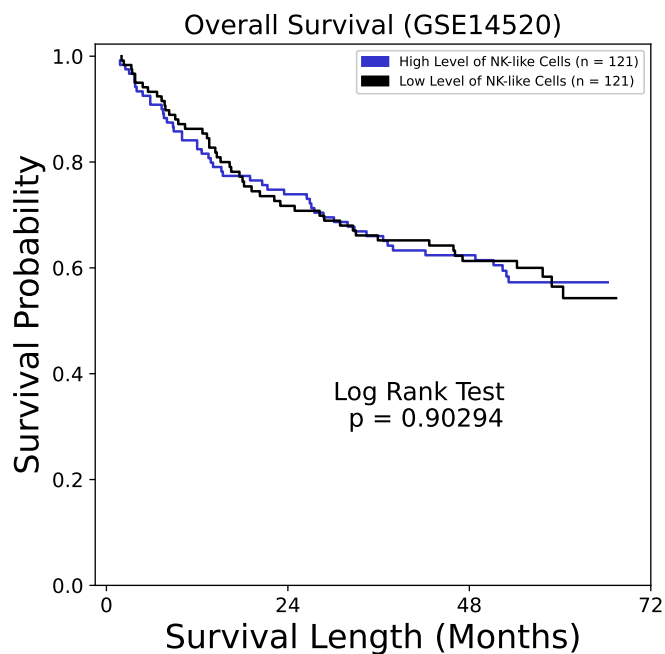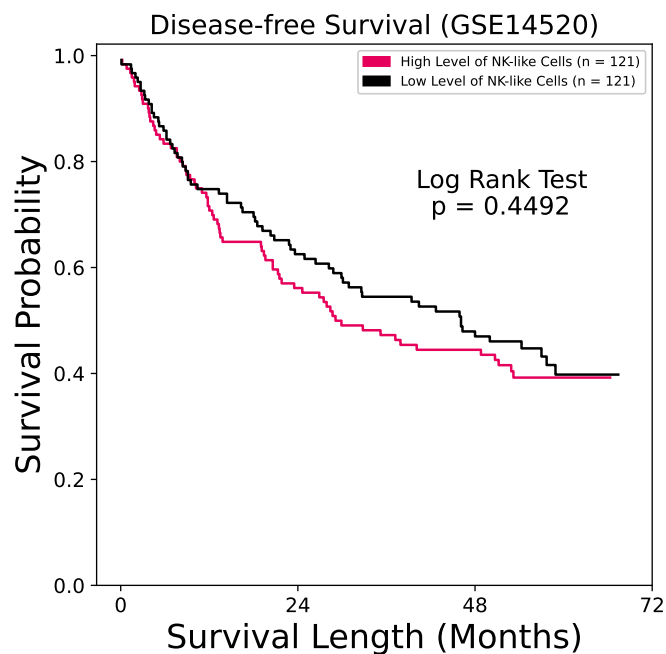

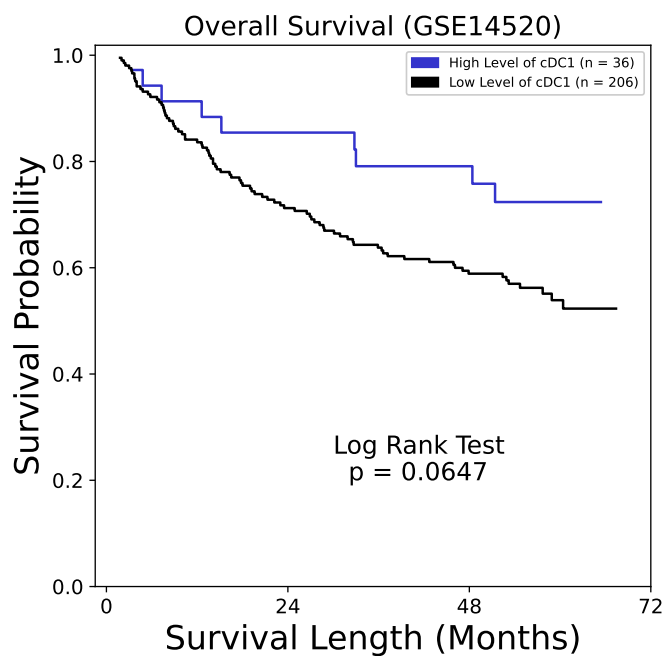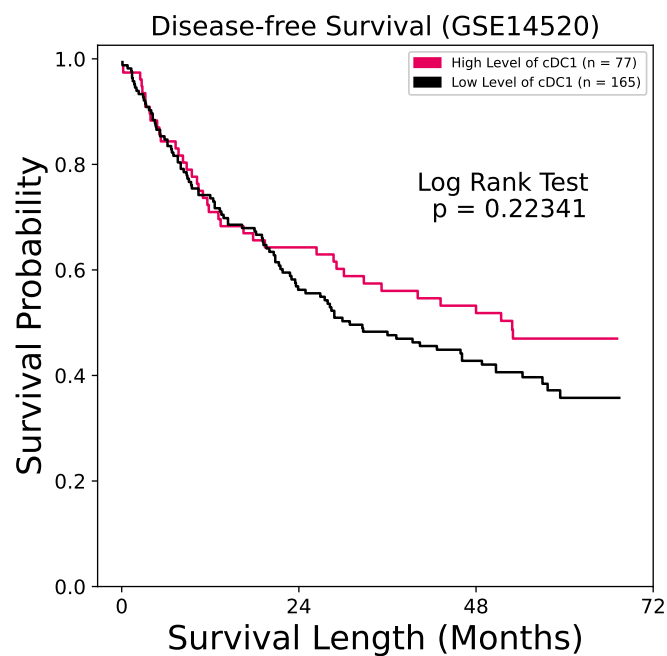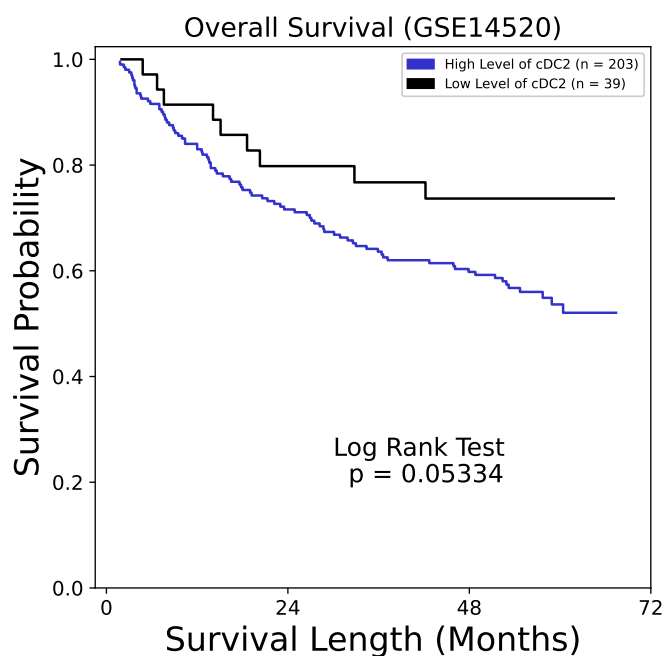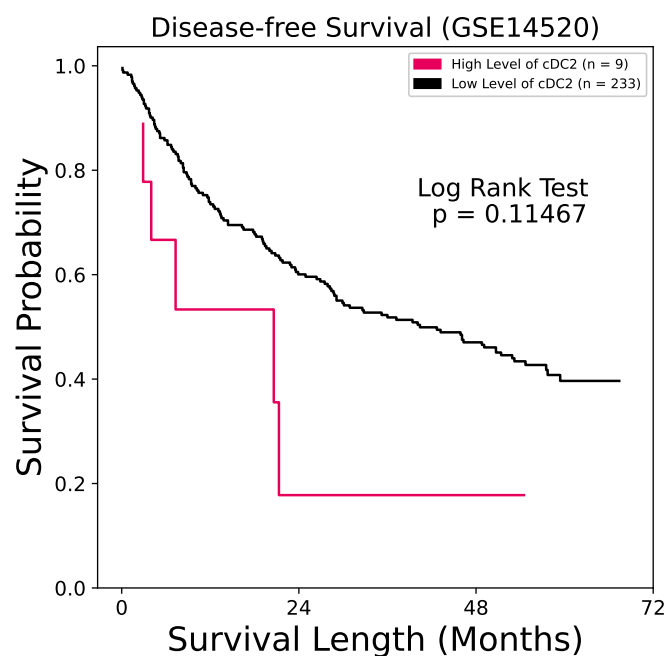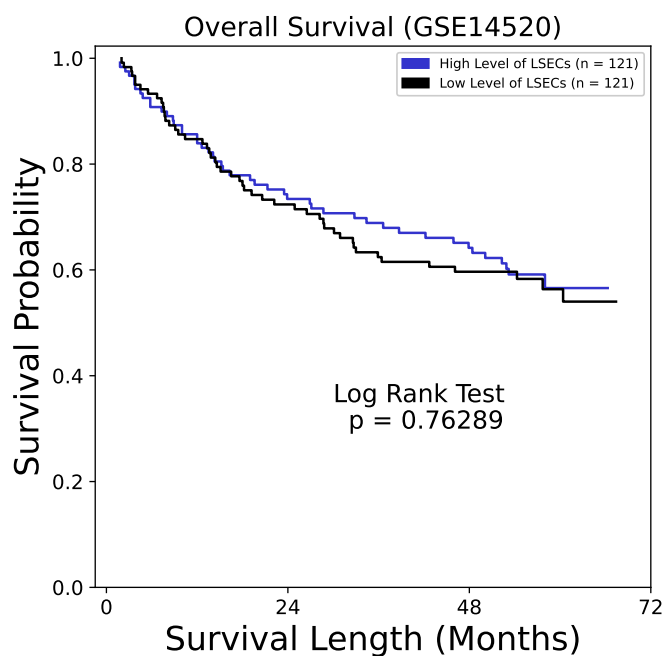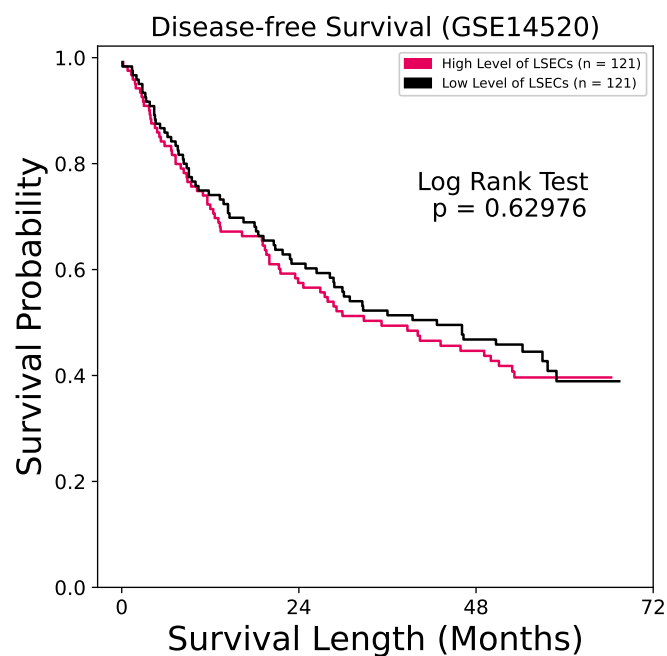

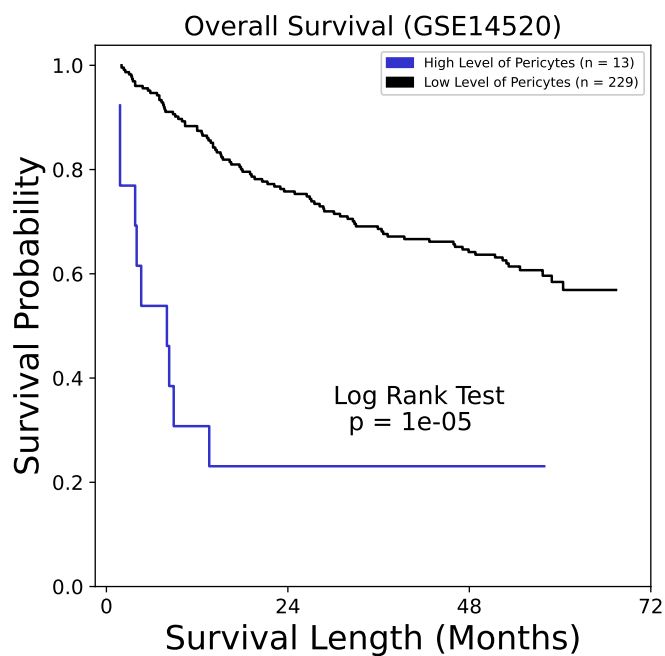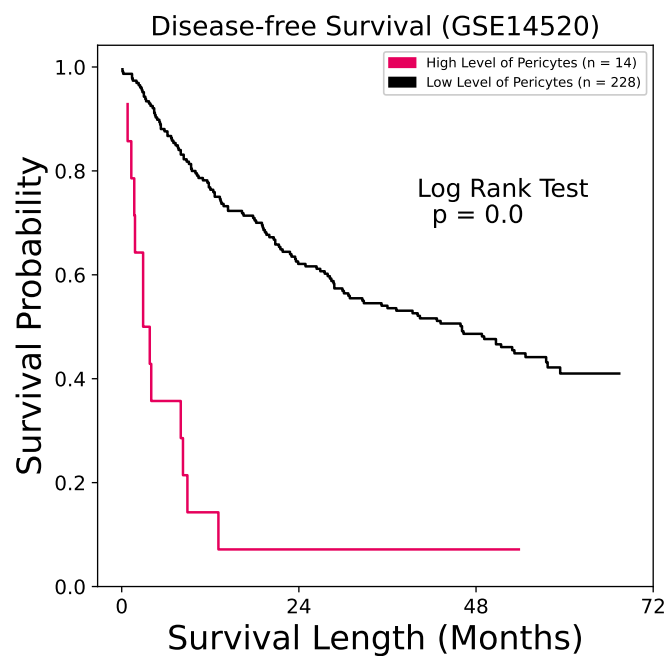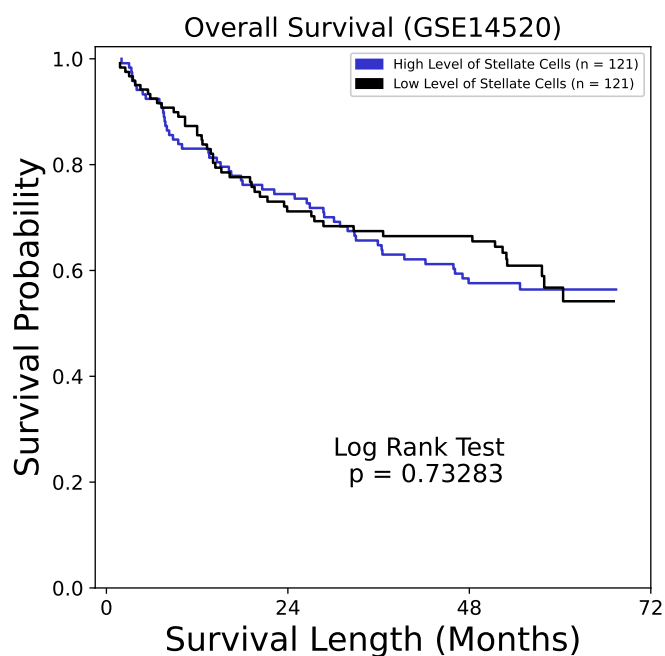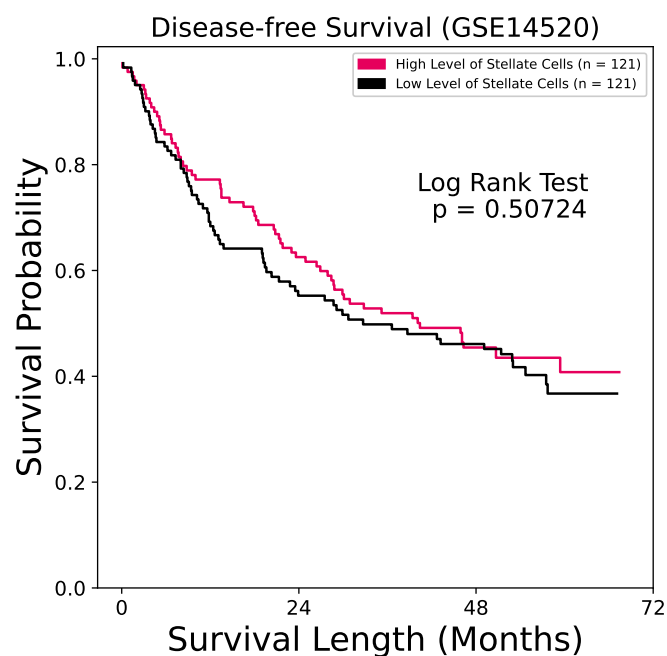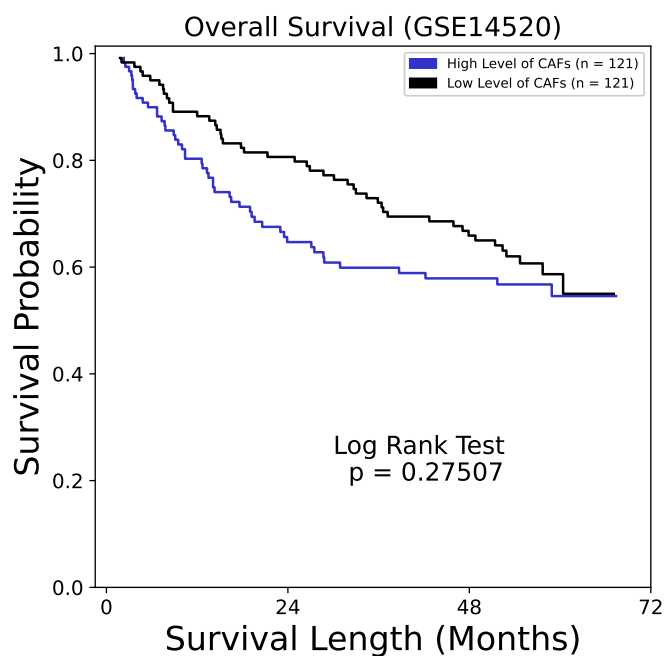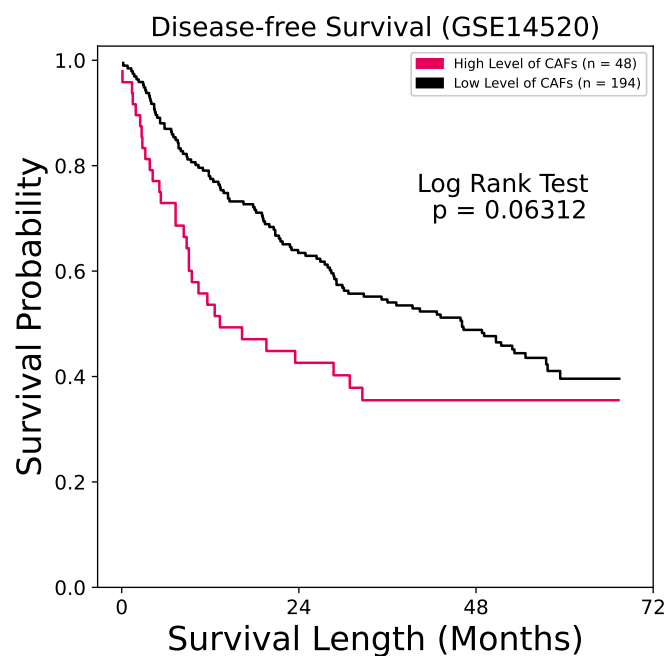

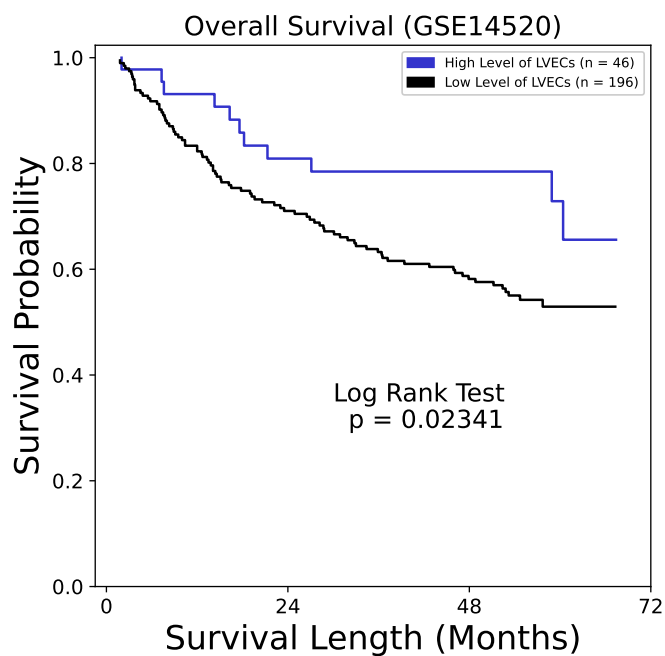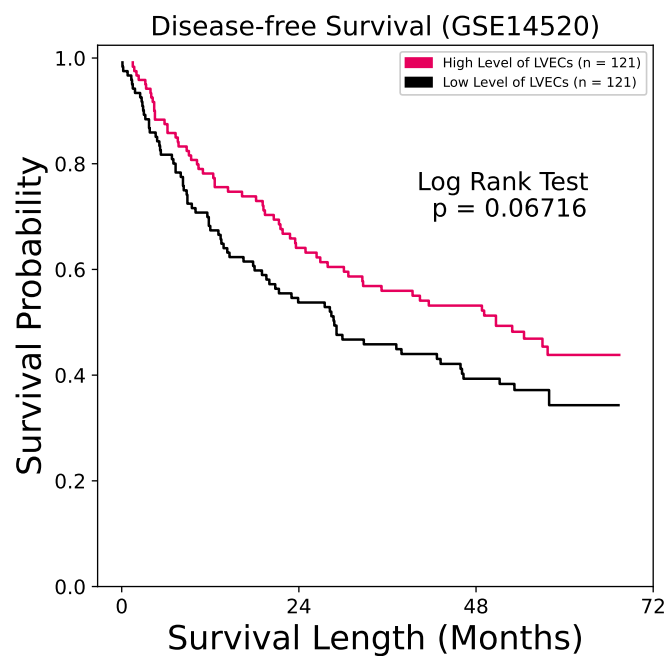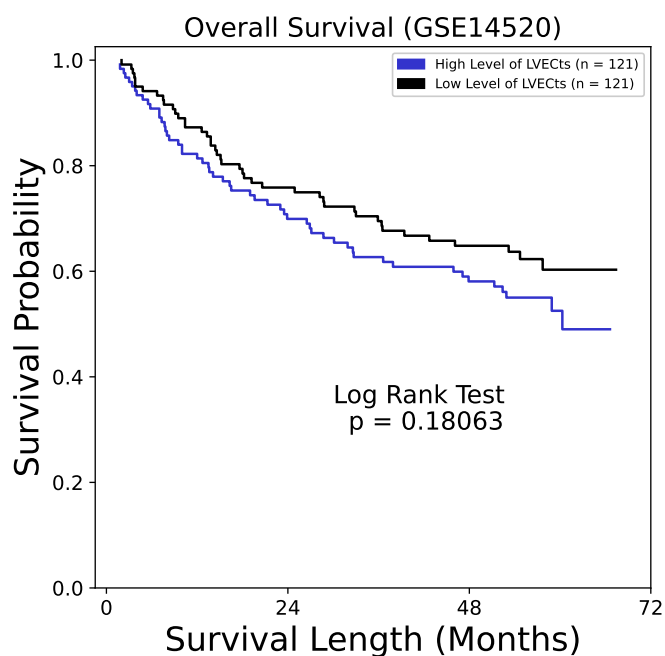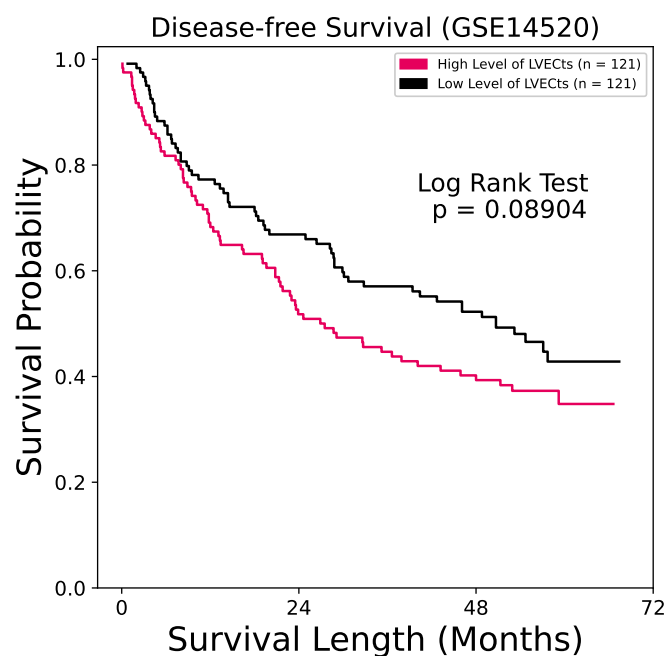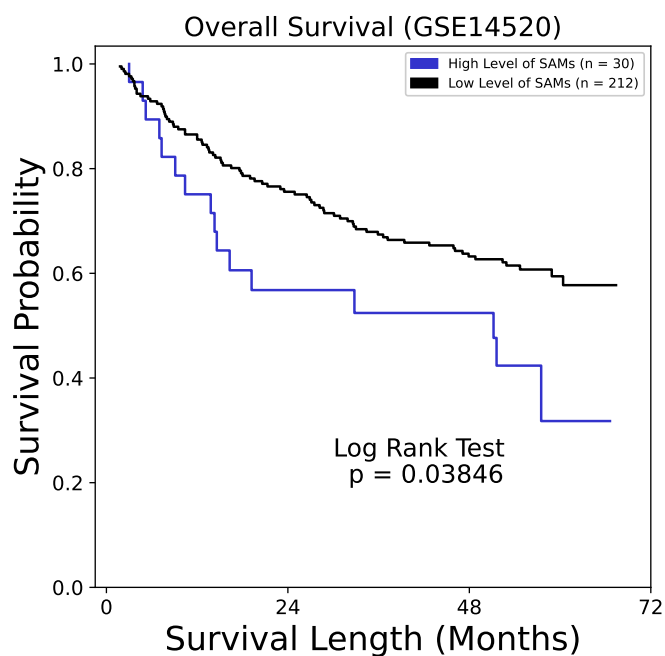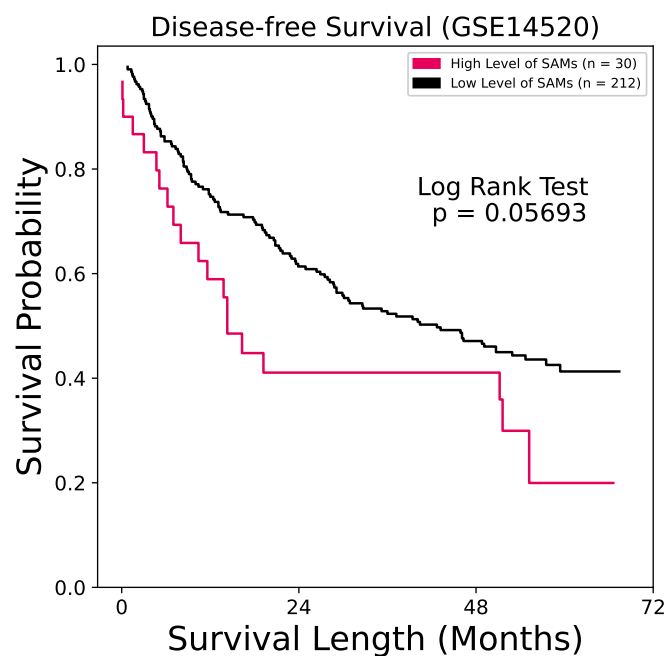

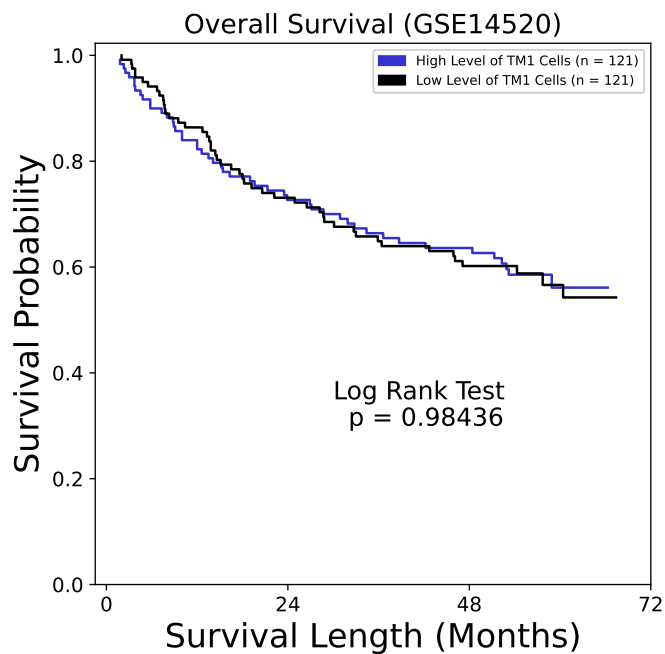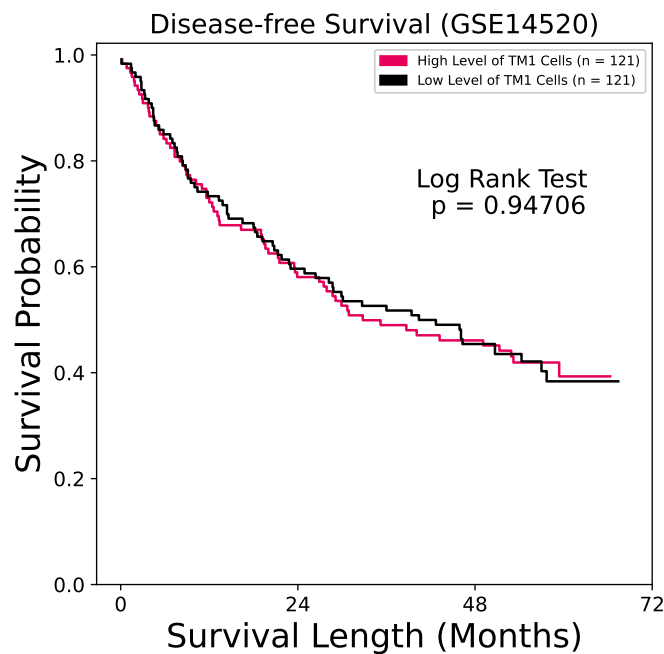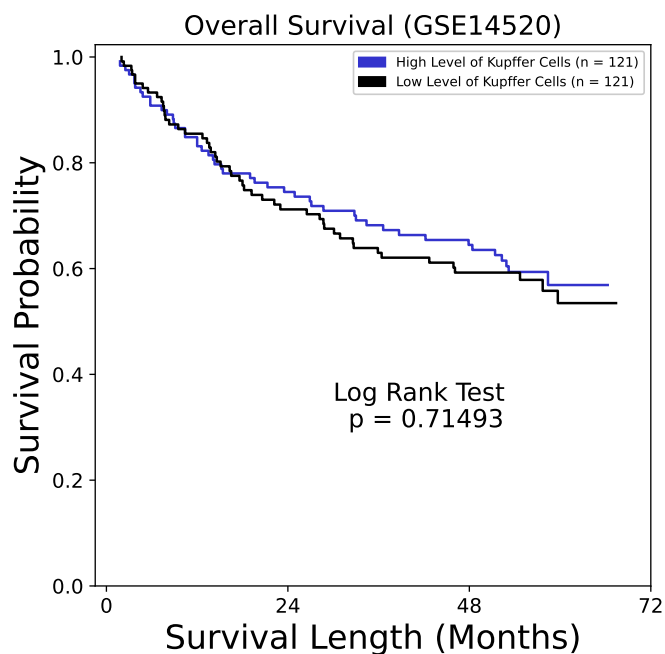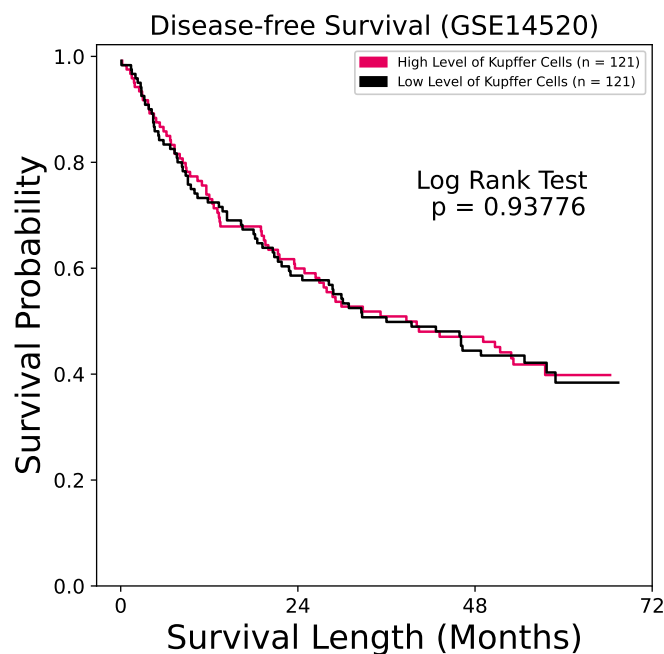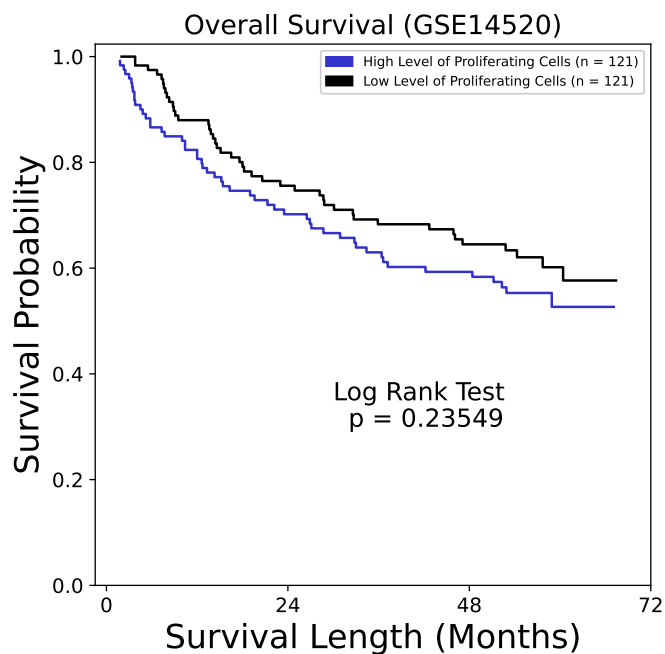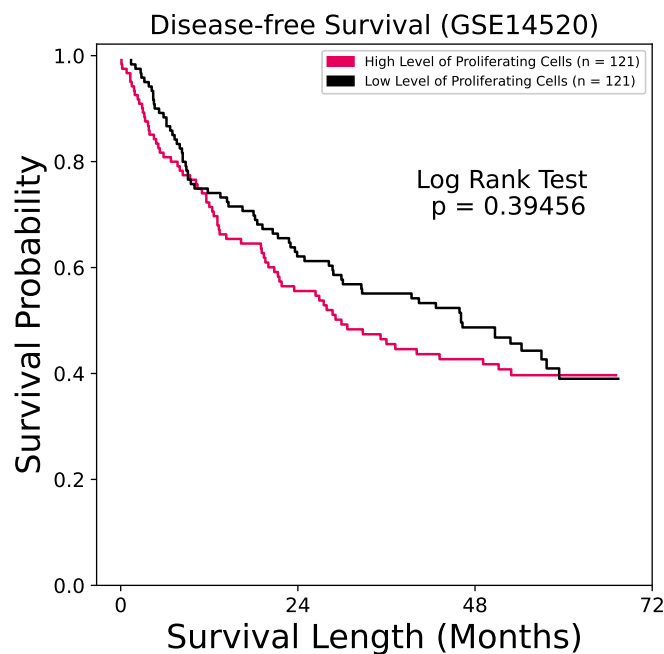

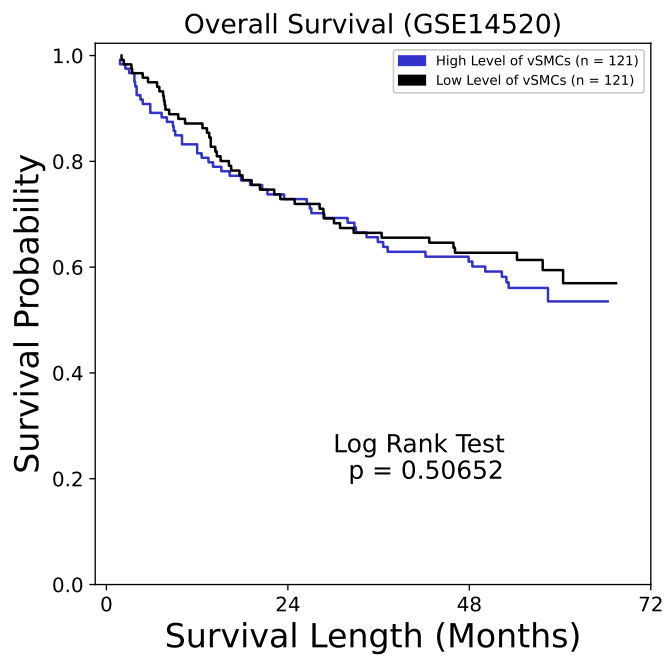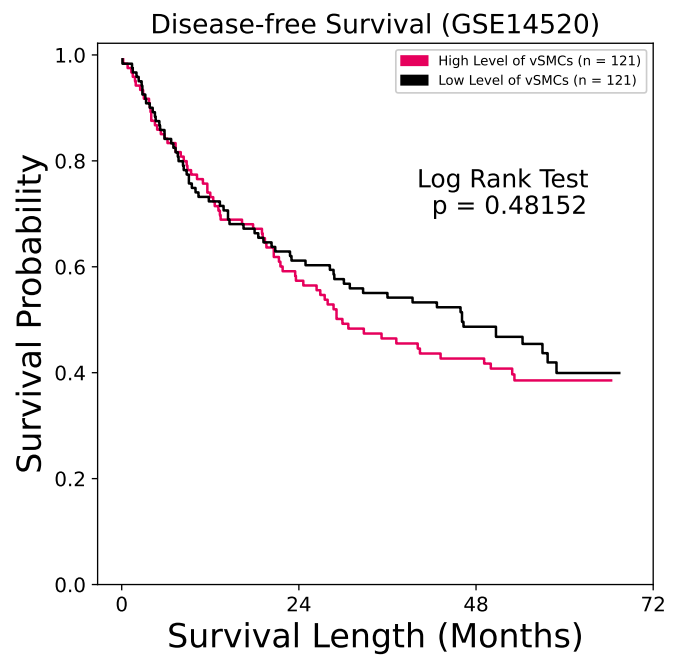

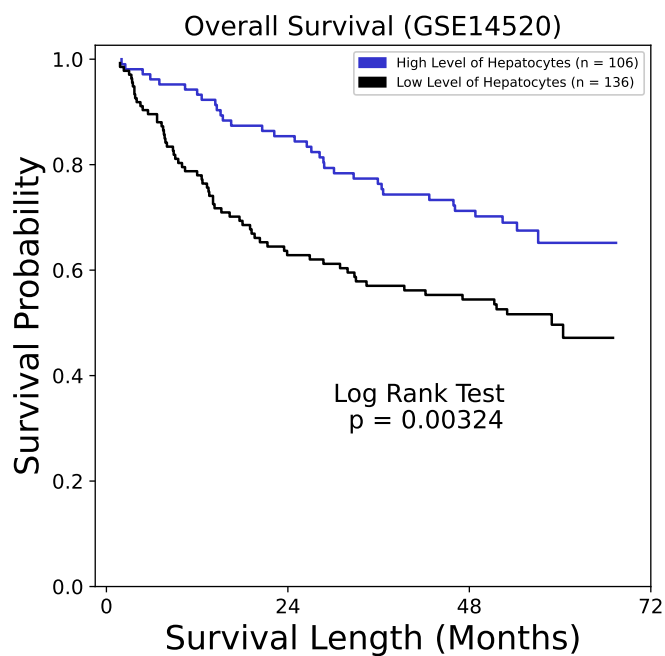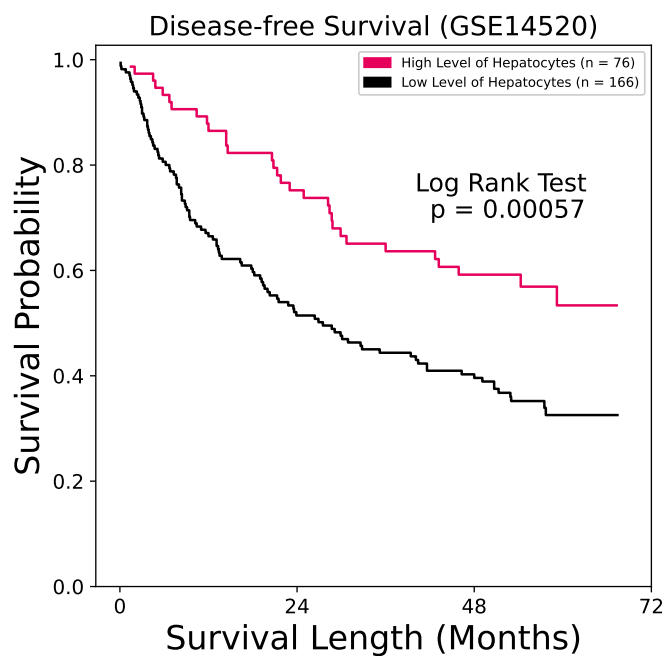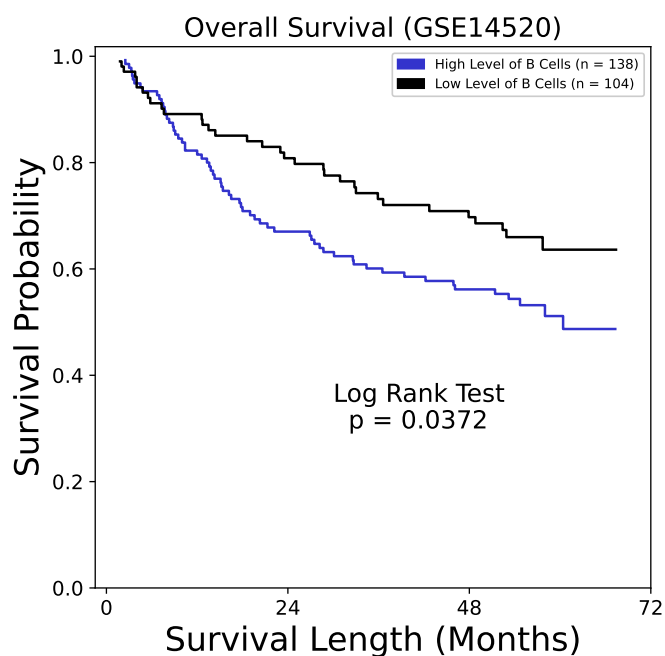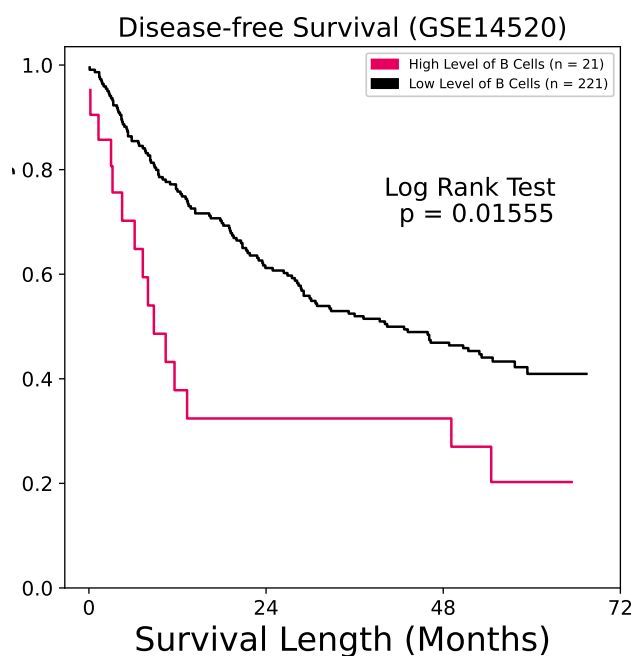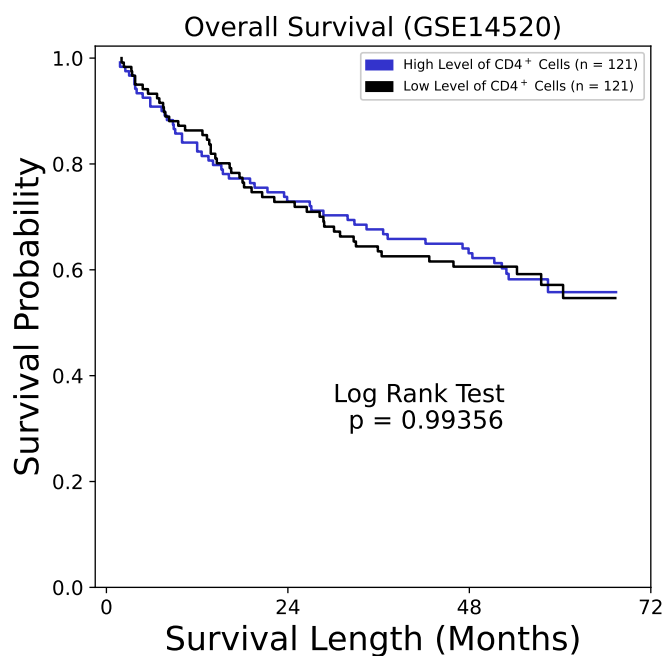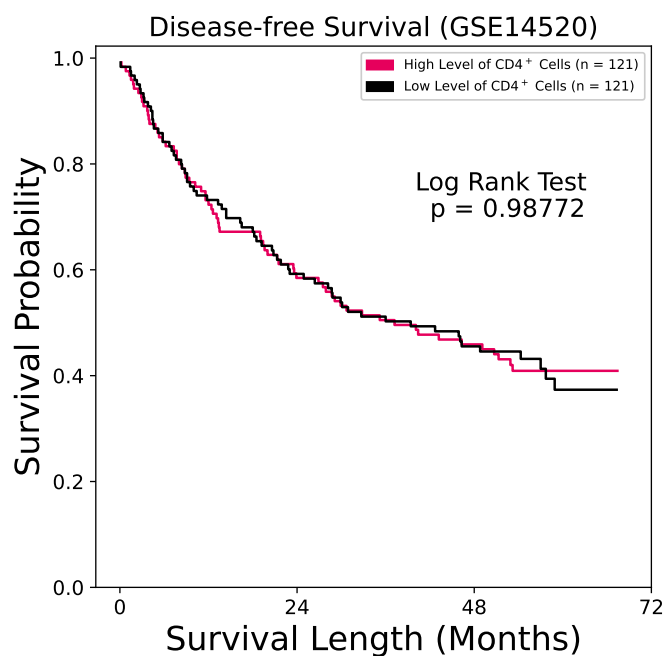

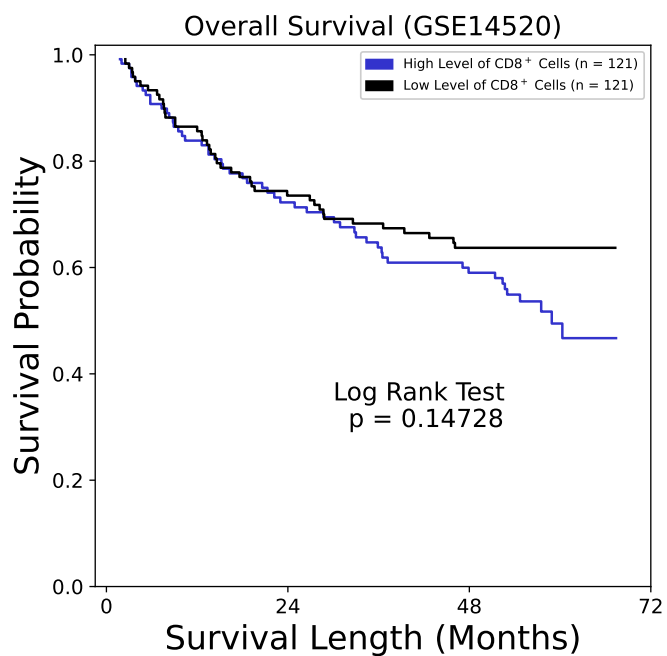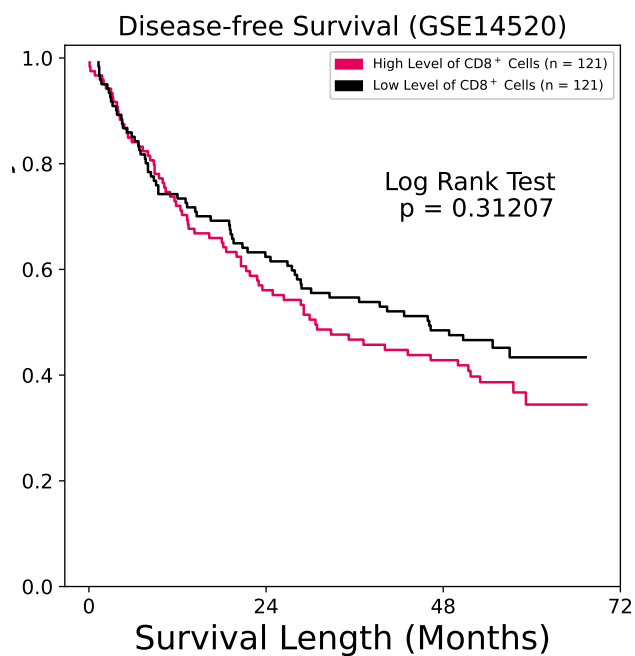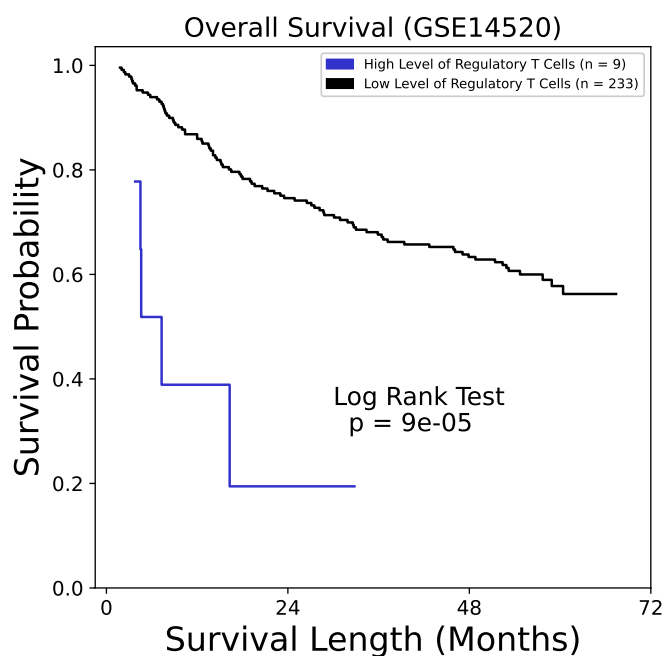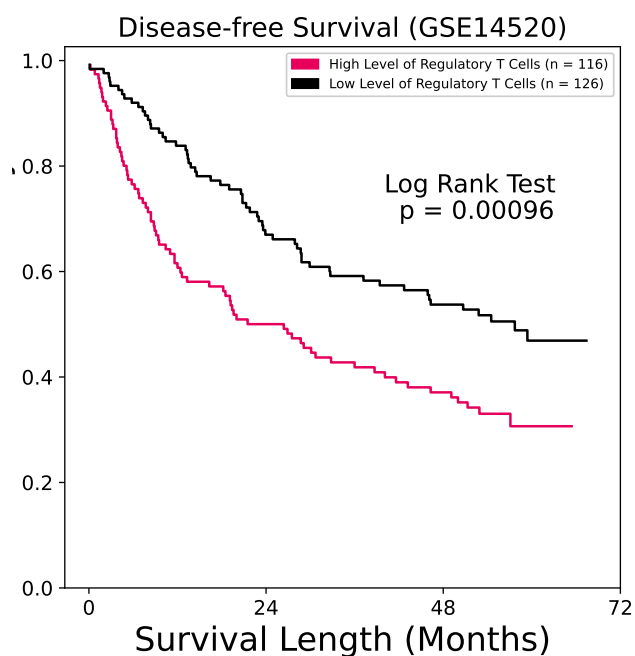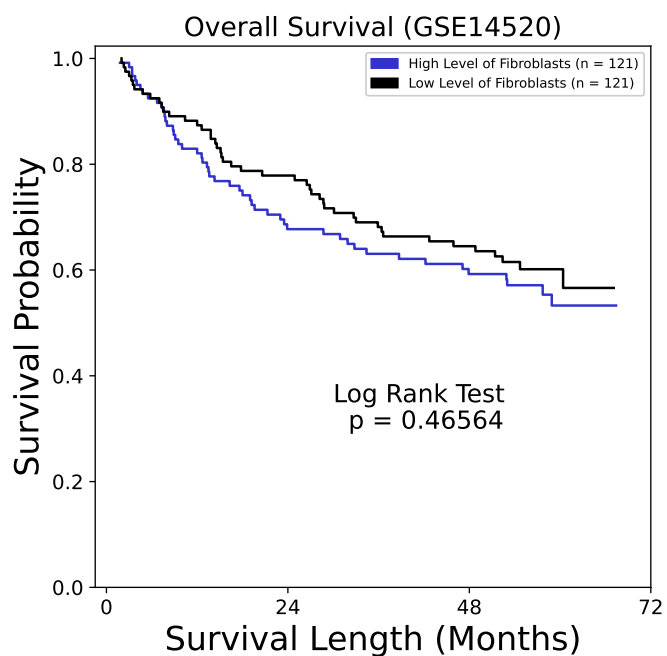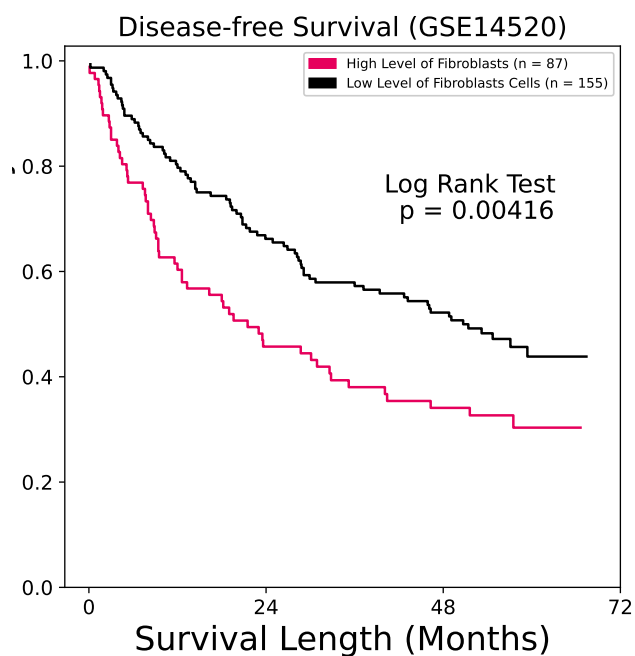

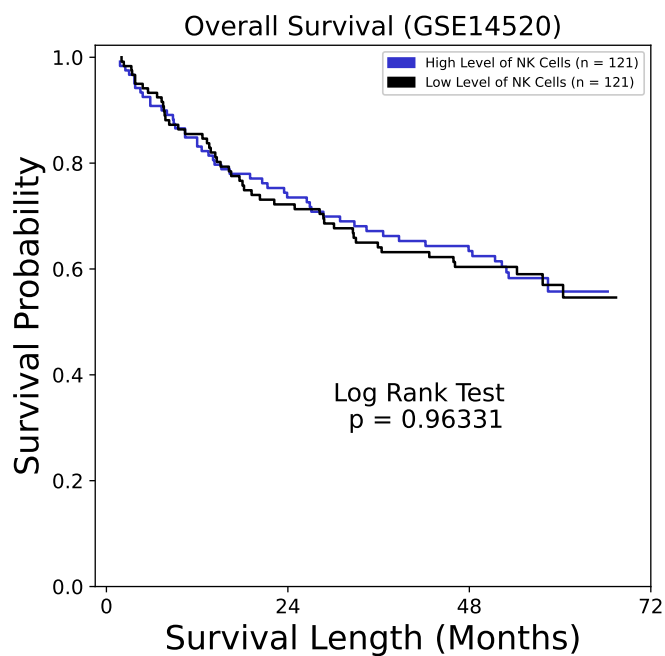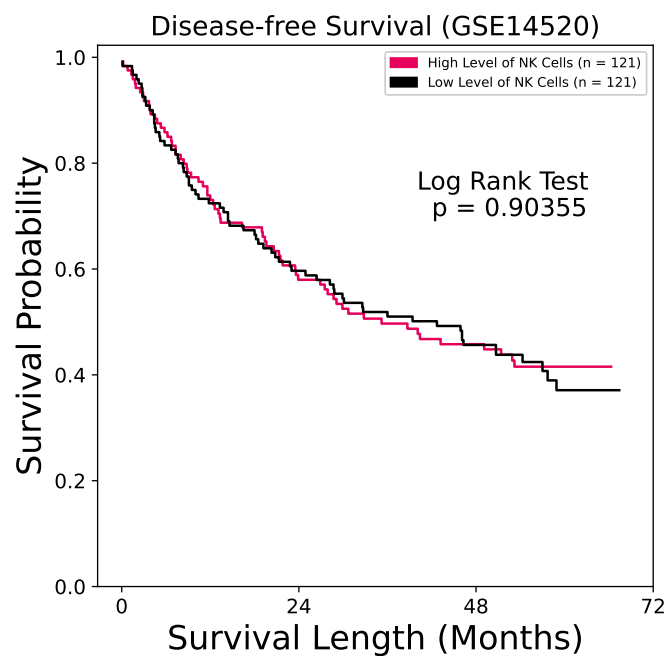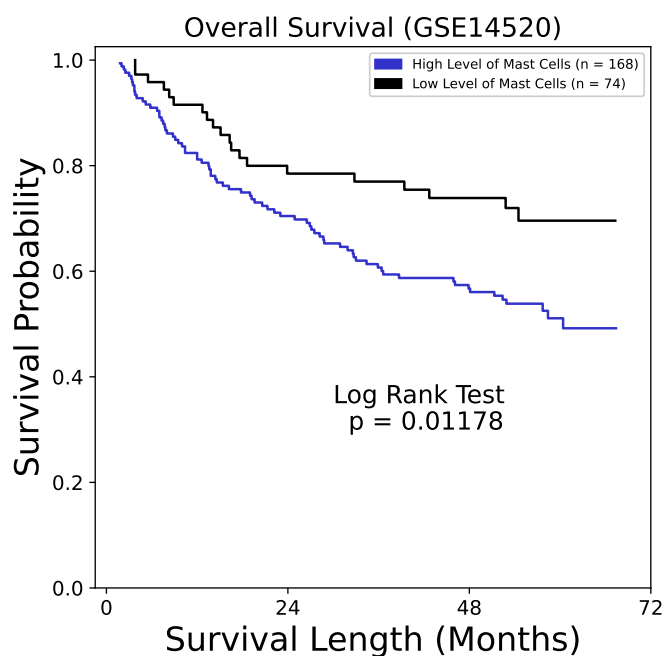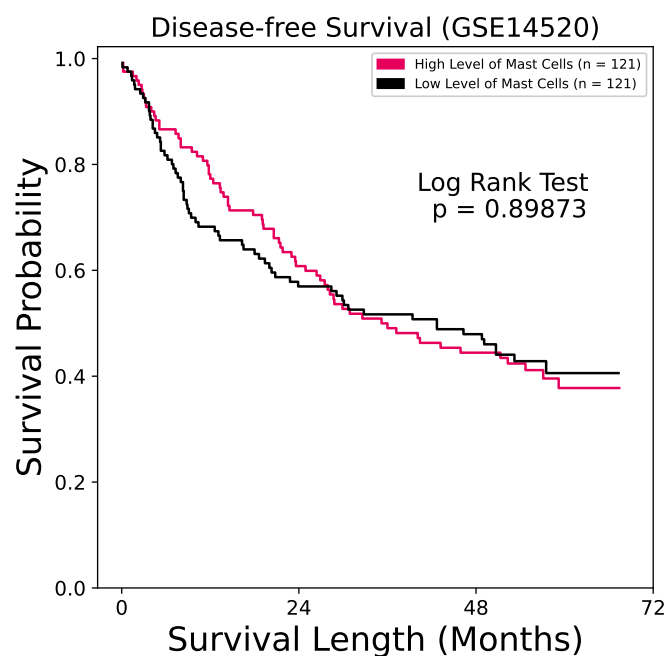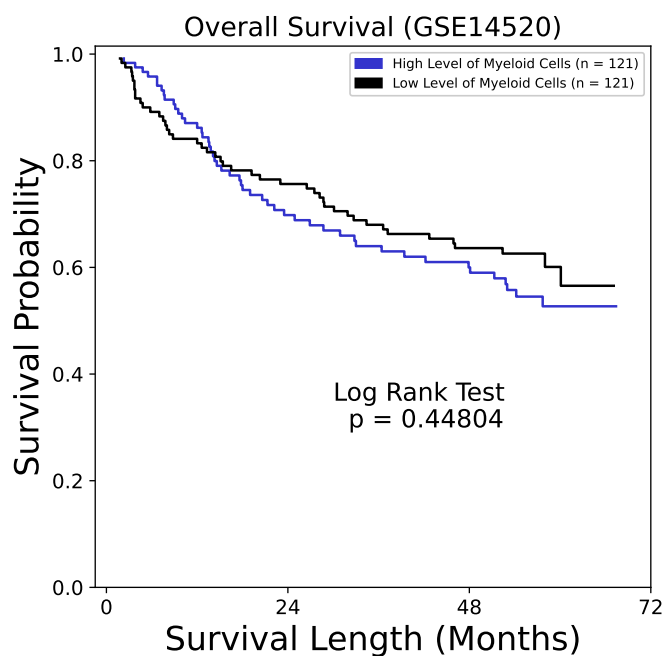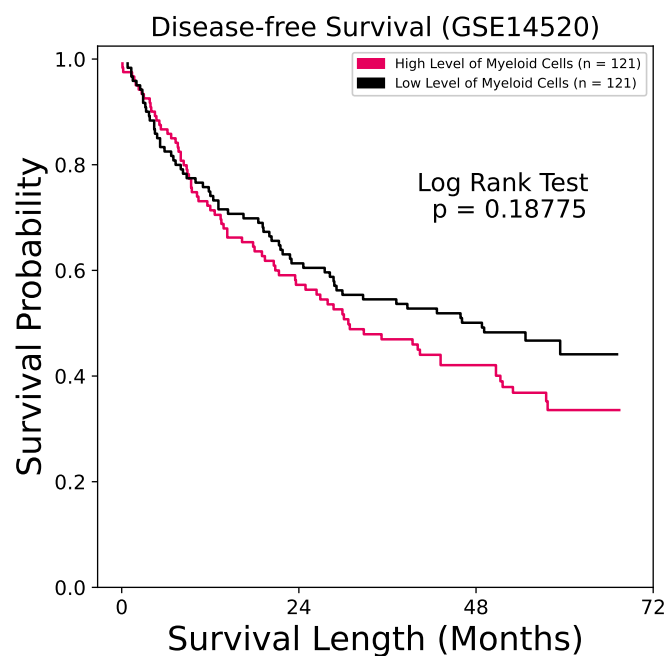

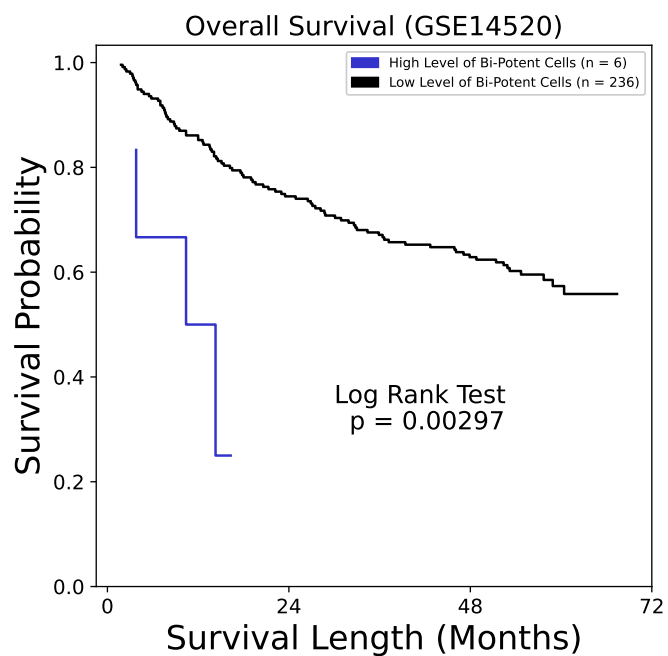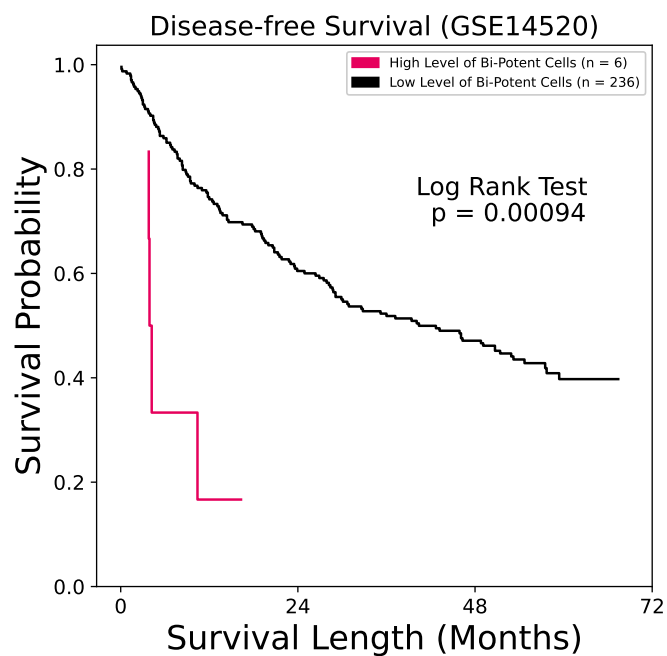

Supplement: Supplementary file 1 [file cancers-15-00153-s001.zip › cancers-2059594-supplementary/Supplements/S6_Survival_Impacts_of_Cell_Fractions_Estimated_by_Cibersortx_GSE14520.pdf]
